# Extensive Adaptive Changes Occur in the Transcriptome of *Streptococcus agalactiae* (Group B Streptococcus) in Response to Incubation with Human Blood

**DOI:** 10.1371/journal.pone.0003143

**Published:** 2008-09-04

**Authors:** Laurent Mereghetti, Izabela Sitkiewicz, Nicole M. Green, James M. Musser

**Affiliations:** 1 Center for Molecular and Translational Human Infectious Diseases Research, Department of Pathology, The Methodist Hospital Research Institute, Houston, Texas, United States of America; 2 Université François-Rabelais, Faculté de Médecine, EA3854 “Bactéries et risque materno-foetal”, et Centre Hospitalier Universitaire, Tours, France; Centre for DNA Fingerprinting and Diagnostics, India

## Abstract

To enhance understanding of how *Streptococcus agalactiae* (group B streptococcus, GBS) adapts during invasive infection, we performed a whole-genome transcriptome analysis after incubation with whole human blood. Global changes occurred in the GBS transcriptome rapidly in response to blood contact following shift from growth in a rich laboratory medium. Most (83%) of the significantly altered transcripts were down-regulated after 30 minutes of incubation in blood, and all functional categories of genes were abundantly represented. We observed complex dynamic changes in the expression of transcriptional regulators and stress response genes that allow GBS to rapidly adapt to blood. The transcripts of relatively few proven virulence genes were up-regulated during the first 90 minutes. However, a key discovery was that genes encoding proteins involved in interaction with the host coagulation/fibrinolysis system and bacterial-host interactions were rapidly up-regulated. Extensive transcript changes also occurred for genes involved in carbohydrate metabolism, including multi-functional proteins and regulators putatively involved in pathogenesis. Finally, we discovered that an incubation temperature closer to that occurring in patients with severe infection and high fever (40°C) induced additional differences in the GBS transcriptome relative to normal body temperature (37°C). Taken together, the data provide extensive new information about transcriptional adaptation of GBS exposed to human blood, a crucial step during GBS pathogenesis in invasive diseases, and identify many new leads for molecular pathogenesis research.

## Introduction

*Streptococcus agalactiae*, also known as group B streptococcus (GBS), is a commensal inhabitant of the human gastrointestinal and genitourinary tract. Pregnant women who carry GBS asymptomatically can transmit the bacterium to their newborns, sometimes resulting in devastating neonatal infection. Although adherence to recommendations designed to prevent perinatal GBS disease has greatly decreased the frequency of early-onset infections [1], the bacterium remains a major cause of late-onset neonatal infections such as bacteremia and meningitis. In addition, GBS has emerged in the last two decades as an important cause of serious infections in elderly patients [1], [2]. Thus, whether considering neonatal or adult infections, in most cases transient passage or prolonged exposure of the bacterium to blood is a crucial step in pathogenesis.

Despite its importance in disease, relatively little is known about how GBS adapts to permit survival and growth in human blood. Many proven or putative GBS virulence factors have been identified, including such well-studied molecules as polysaccharide capsule and extracellular hemolysin. Proven virulence factors also include surface-exposed or secreted proteins such as the Alp protein family, C5a peptidase, Lmb protein, fibrinogen-binding protein FbsA, CspA protein, hyaluronate lyase, and pilins (for extensive review on virulence factors see references [3], [4], [5], [6], [7]. More recently, other proteins involved in adherence, metabolism, or regulation have been shown to contribute to GBS virulence and survival in blood [8], [9], [10]. It is important to stress that GBS pathogenesis is a complex process that likely involves whole-transcriptome remodeling, not simply up-regulation of a few virulence genes [11]. Many factors that allow the bacterium to persist and thrive in humans remain to be identified. Thus, studies performed under conditions that approximate human physiological parameters may contribute new information about pathogenesis [12].

To enhance our understanding of the capacity of GBS to adaptively respond to contact with human blood, we conducted a whole-genome transcriptome analysis during GBS incubation with blood obtained from healthy volunteers. The study was modeled on analyses conducted with group A streptococcus (GAS) grown *ex vivo* in human saliva and blood [13], [14], and GBS grown in human amniotic fluid [15]. In the present study, blood samples were mixed with GBS and incubated at 37°C, the physiological temperature of the human body, and 40°C, a temperature closer to that occurring in patients with severe infection and high fever. Bacterial transcriptome analysis was performed before mixing bacteria with blood, and after 30 and 90 minutes of incubation. We observed extensive remodeling of the GBS transcriptome during adaptive culture in human blood. A very large number of genes was down-regulated, whereas transcription was enhanced of genes encoding proteins important for successful adaptation and establishment of the bacterium in the blood.

## Results

### Global expression microarray analysis

**Figure 1:**
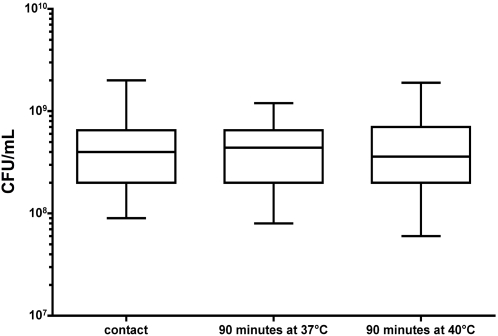
Number of bacterial cells during during incubation with human blood. The average of the CFU/mL for the seven blood samples after 90 min of incubation at 37°C and 40°C was not statistically different from the average of the CFU/mL for the seven blood samples at time 0.

Blood obtained from each of the seven donors was manipulated and processed separately. We confirmed by CFU counting that there was no significant difference in the number of GBS inoculated into each blood sample (data not shown). We also documented that there was no significant difference between donors in the change in number of bacteria during the 90-minute incubation in blood (Fig. 1).

**Figure 2:**
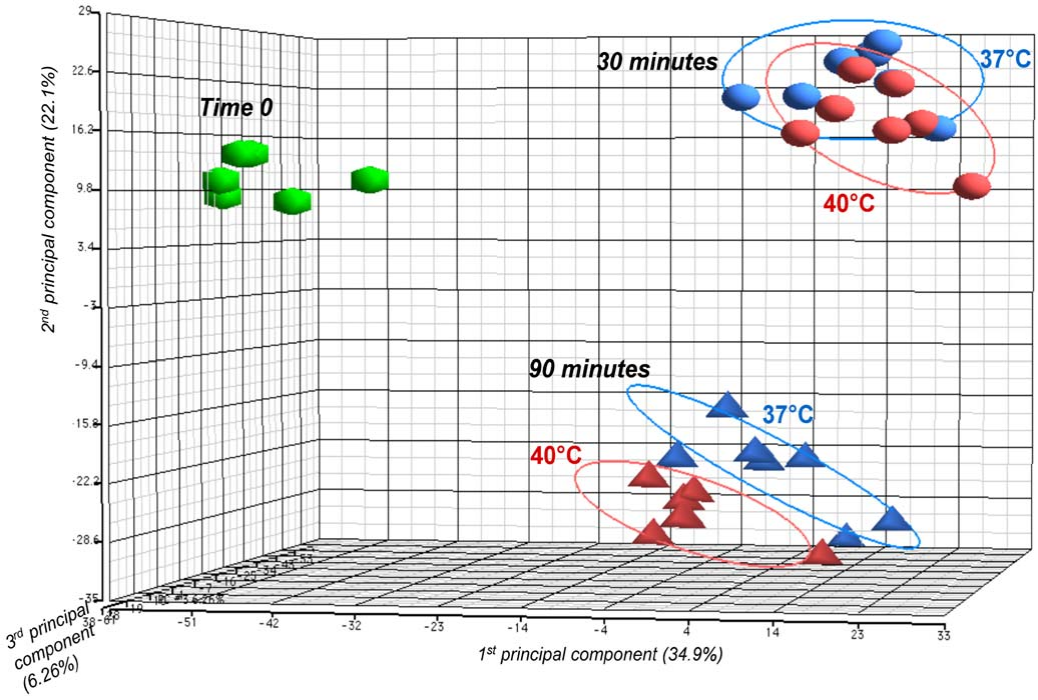
Principal component analysis (PCA) plot showing transcriptome differences between expression microarray data of GBS strain NEM316 strain incubated in human blood at 37°C and 40°C. Samples were analyzed at time 0, and after 30 min and 90 min of incubation. The PCA plot captures the variance in a dataset in terms of principal components and displays the most significant of these on the x, y, and z axes. The percentages of the total variation that are accounted for by the 1^st^, 2^nd^, and 3^rd^ principal components are shown on the x-, y- and z-axes labels. Plots are colored by temperature of incubation and shaped by time point. Elipses circled plot each time point.

For each blood sample, five different transcript data sets were obtained at the following points: immediately after mixing GBS with blood (time 0), after 30 min (time 1) of incubation with blood at 37°C and 40°C, and after 90 min (time 2) of incubation with blood at 37°C and 40°C. Principal component analysis (PCA) showed extensive clustering of the transcriptome data among the seven samples at time 0, and 30 min and 90 min incubation (Fig. 2). Not unexpectedly, there was a slight difference in the transcriptome clustering at 90 min between samples incubated at 37°C and those incubated at 40°C, suggesting an influence on the transcriptome of the temperature of incubation after a longer period of contact with blood (Fig. 2, and see text below). Taken together, these results indicated that the transcriptome profile data were of sufficient quality to permit robust statistical analysis and interpretation.

Using data obtained from the seven donors, we calculated the average transcript level for each gene at each time point for the 1,995 ORFs present on the chip. Differences in gene transcripts were determined by comparing the average values from each time point or temperature to average values obtained at another time point or temperature (extensive results for all ORFs are presented as supplementary table S1).

### Changes in the GBS transcriptome induced by human blood

**Figure 3:**
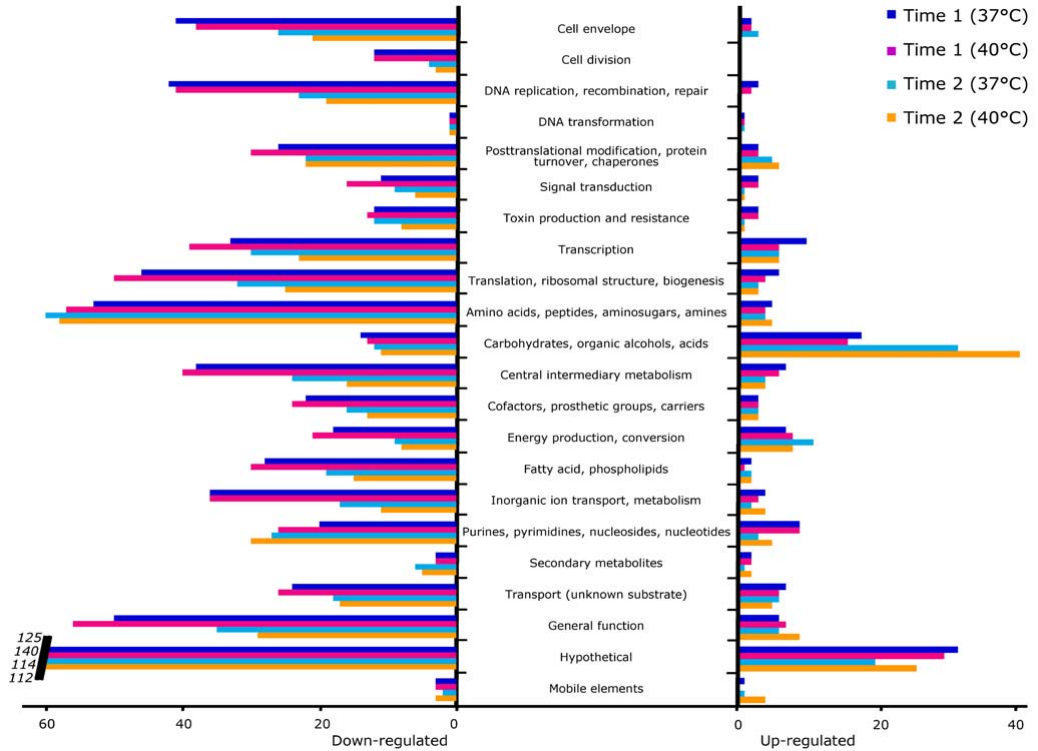
Differential regulation of transcript expression in GBS strain NEM316 after incubation with human blood. Genes were classified into 22 main functional categories. Bars indicate the numbers of genes whose expression was modified at one time-point (time 1 at 37°C, time 1 at 40°C, time 2 at 37°C, or time 2 at 40°C) relative to time 0. On the left, genes that were down-regulated relative to time 0. After 30 min of incubation, 658 transcripts were down-regulated at 37°C and 715 transcripts were down-regulated at 40°C. After 90 min of incubation, 518 transcripts were down-regulated at 37°C and 456 transcripts were down-regulated at 40°C. On the right, genes that were up-regulated relative to time 0. After 30 min of incubation, 134 transcripts were up-regulated at 37°C and 119 transcripts were up-regulated at 40°C. After 90 min of incubation, 115 transcripts were up-regulated at 37°C and 135 transcripts were up-regulated at 40°C. Carbohydrate metabolism genes were the most numerous up-regulated genes (apart from the hypothetical gene category).

The data revealed extensive remodelling of the GBS transcriptome after the shift from THY broth into human blood. For example, more genes were expressed in THY (at time 0) than in blood, regardless of the time or temperature of incubation considered. After 30 min at 37°C, 134 transcripts were up-regulated and 658 were down-regulated compared to time 0, whereas 119 transcripts were up-regulated and 715 were down-regulated at 40°C compared to time 0 (Table 1 and Fig. 3). Similarly, 115 transcripts were up-regulated and 518 were down-regulated after 90 min at 37°C compared to time 0, and 135 transcripts were up-regulated and 456 were down-regulated at 40°C compared to time 0 (Table 1 and Fig. 3).

**Table 1:** Number (and percentage) of GBS transcripts significantly up- and down-regulated according to the temperature and duration of incubation in human blood.

	37°C		40°C	
	After 30 minutes (time 1)	After 90 minutes (time 2)	After 30 minutes (time 1)	After 90 minutes (time 2)
Up-regulation	134 (6.7%)	115 (5.7%)	119 (6.0%)	135 (6.8%)
Down-regulation	658 (33.0%)	518 (26.0%)	715 (35.8%)	456 (22.8%)
Total	792 (39.7%)	633 (31.7%)	834 (41.8%)	591 (29.6%)

An ORF was considered to be differentially expressed if there was a “present” signal, if there was a significant (*P*<0.05, T-test) change in expression greater than 2-fold at one time point relative to another and if these criteria were met for at least six of the seven donor samples. Up- and down-regulation are expressed relatively to time 0.

Percentage are expressed relative to the 1,995 ORFs present on the chip.

Most of the transcript differences observed between 37°C and 40°C were due either to a ratio between levels of expression slightly above 0.5 or below 2, or to a *P* value slightly above 0.05 (Table S1). Thus, there was extensive overlap of the GBS transcriptome at the two temperatures studied. Consequently, all the results presented subsequently are based on comparison with the transcript data obtained at 37°C, except the last paragraph of the Results section which describes key substantive differences between the 40°C and 37°C data.

We identified drastic down-regulation of the genes of most of the functional subcategories (83% of the genes whose expression was modified were down-regulated at 30 min relative to time 0) (Fig. 3). In contrast, genes encoding proteins involved in transcriptional regulation, carbohydrate and purine/pyrimidine metabolism, and in contact with the host cells were up-regulated (Fig. 3).

### Stress response of GBS during incubation with blood

Inasmuch as the shift from rich THY medium to blood involves a considerable change in the environment, we anticipated that GBS would experience a substantial stress response. However, this was not the case. For example, after the first 30 min of incubation with human blood there was no up-regulation of the GBS genes involved in heat/cold stress conditions. The most notable changes concerned the transcripts for gbs1721, encoding a universal stress protein, and gbs0808 (*sodA*), a gene that confers protection against oxidative stress and is required for maintaining a high level of bacteremia in mice after experimental inoculation [16]. Gbs1721 and gbs0808 were 8.4- and 2.4-fold up-regulated, respectively, at 30 min post-inoculation into blood. After 90 min, additional stress response genes were up-regulated (from 2.3- to 9-fold), including genes encoding several stress proteins (gbs1202, gbs1204, gbs1721), a chaperone (gbs0625), ClpL protease (gbs 1376), and a stress response regulator (gbs0756) (Table S1). Interestingly, gbs1202 and gbs1204 are homologs of the enterococcal general stress protein Gls24 implicated in the stress response and *Enterococcus faecalis* virulence [17].

The switch from growth in THY to blood resulted in a large decrease in the number of transcripts of genes in all functional categories, especially genes involved in cell division and cell envelope processes. Indeed, we observed down-regulation of most of the genes involved in cell wall metabolism (Fig. 3) after 30 min in blood (ranging from 2- to 10-fold reduction). In addition, the entire polysaccharide capsule (gbs1233–1247) and group B antigen (gbs1480–1494) clusters were down-regulated (Table S1). Conversely, gbs0182 and gbs0183, genes encoding two proteins similar to *Staphylococcus aureus* LrgAB, were 17.8- and 15.3-fold up-regulated, respectively, at 90 min relative to 30 min. Interestingly, LrgAB in *S. aureus* function together to inhibit murein hydrolase activity, and their transcription is responsive to carbohydrate metabolism [18].

### Many transcriptional regulators are altered when GBS is grown in human blood

After 30 min of incubation with blood, 18 transcriptional regulators were down-regulated and 10 were up-regulated (Table S1). For example, gbs0833, gbs1344, gbs1793, gbs1807 and gbs1958, which have homology with the Cro/CI or MerR families of transcriptional regulators, were more than 4-fold up-regulated. Although little is known about the function of these proteins in GBS, transcriptional regulators of the MerR family, which usually act as activators, respond to the presence of essential and toxic metals [19]. Of note, it was recently shown that a MerR-like regulator in *Streptococcus pneumoniae* is required for survival in blood [20].

After 90 min of incubation, more complex transcriptome changes were observed. Some regulators that were up-regulated at 30 min relative to time 0 were down-regulated at 90 min relative to 30 min, whereas other regulators initially down-regulated were subsequently up-regulated. Thus, gbs0267, a putative trans-acting positive regulator with some similarity to the *mga* positive regulator of virulence in GAS, was 3-fold down-regulated after 30 min in blood, but 9.6-fold up-regulated at 90 min relative to 30 min. We observed similar transcript fluctuations, although of lower magnitude, for gbs1051 (*lytR*), gbs1671 (*covS*), and gbs1719 (*codY*) which were initially down-regulated, and subsequently up-regulated. A homologue of LytSR has been implicated in controlling the rate of autolysis by affecting the *S. aureus* murein hydrolase activity [21], and in *S. pneumoniae* this two component system (TCS) is important for *in vivo* adaptation and pathogenesis [22]. CovS is part of another TCS extensively studied in GBS that regulates more than 100 genes, including virulence genes [23], [24], [25]. Taken together, the extensive changes we observed in the regulation of GBS transcriptional regulators illustrate the complex response used by GBS to adapt to human blood.

### Major changes in transcription of genes involved in host-cell interaction and coagulation/fibrinolysis

**Figure 4:**
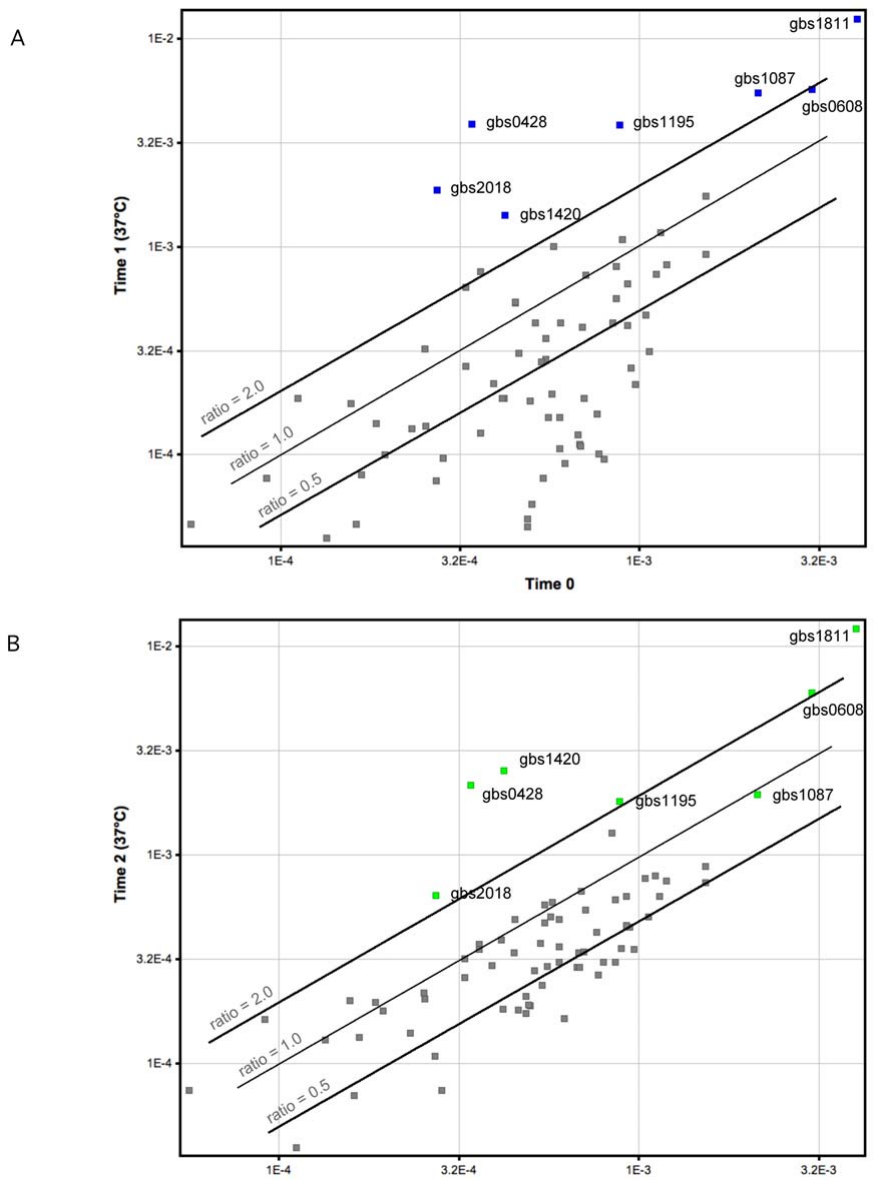
Differential regulation of virulence gene expression in GBS strain NEM316 during incubation with human blood. The scatter diagram displays normalized spot intensities of the microarray analysis from proven and putative GBS virulence genes. Genes of interest are in blue or green; other proven and putative GBS virulence factors are in gray. (a) After 30 min of incubation with blood at 37°C (time 1 relative to time 0). Higher level of transcription is shown for genes encoding proteins with LPXTG motifs: gbs0428, gbs1087 (*fbsA*), gbs1420 (*bsp*), and gbs2018 (*bibA*); and genes encoding proteins implicated in binding/activation of plasminogen: gbs0608 (*eno*), gbs1811 (*gapC*), and gbs1195 (*ska*). Numerous other proven and putative virulence genes were either down-regulated or their transcription was not modified. (b) After 90 min of incubation with blood at 37°C (time 2 relative to time 0). Six of the previous seven genes (except gbs1087-*fbsA*) remained up-regulated after 90 min of incubation with blood. Levels of transcription of the other proven and putative virulence genes were higher relative to incubation of 30 min.

Relatively few proven virulence genes were significantly up-regulated in response to culturing in human blood. Most known and putative virulence genes were either down-regulated or their transcript level was not altered, although after 90 min of incubation in blood the level of many transcripts was increased (Fig. 4). Importantly, we observed up-regulation of four genes encoding LPXTG proteins after 30 min of incubation in blood, including gbs0428, gbs1087 (*fbsA*) encoding a major receptor for fibrinogen, gbs1420 (*bsp*) which encodes a putative choline-binding protein, and gbs2018 (*bibA*) which encodes a protein that binds to human C4-binding protein and contributes to survival in human blood [10] (Fig. 4A and Table 2). Interestingly, the level of transcripts of three genes encoding proteins implicated in binding or activation of plasminogen also were higher in human blood than in THY, including gbs0608 (*eno*) encoding enolase, gbs1811 (*gapC*) encoding glyceraldehyde 3-phosphate dehydrogenase (GAPDH), and gbs1195 (*ska*) encoding a secreted protein similar to streptokinase [26] (Fig. 4A and Table 2). After 90 minutes of incubation, six of these seven genes (except gbs1087-*fbsA*) remained up-regulated relative to time 0 (Fig. 4B and Table 2).

**Table 2:** GBS genes involved in virulence significantly up-regulated in human blood.

ORF	Gene	Function/Characteristic	Ratio time 1 vs time 0	Ratio time 2 vs time 0
gbs0428	*-*	LPXTG motif	11.0	6.4
gbs0608	*eno*	enolase	1.9	2.0
gbs1087	*fbsA*	LPXTG motif	2.6	0.9
gbs1195	*ska*	streptokinase-like	4.4	2.1
gbs1420	*bsp*	LPXTG motif	3.4	6.0
gbs1811	*gapC*	GAPDH^a^	3.1	3.0
gbs2018	*bibA*	LPXTG motif	6.9	2.4

Seven genes involved in cell-to-cell contact, adhesion to fibrinogen, and coagulation/fibrinolysis processes were up-regulated after 30 min of incubation with blood (time 1 relative to time 0). Six of these genes were also up-regulated after 90 minutes of incubation (time 2 relative to time 0).

a GAPDH: glyceraldehyde 3-phosphate dehydrogenase.

### Extensive changes in the transcripts of genes encoding proteins involved in GBS adaptive metabolism

The expression of a large proportion of genes involved in various metabolic pathways was influenced by incubation with human blood, reflecting rapid adjustment of the bacterial cellular metabolism to nutrients and conditions present in the new environment. We observed that major changes occurred in genes involved in carbohydrate metabolism or transport, as transcripts of 55 genes were significantly altered at 30 min or 90 min relative to time 0. Most of these altered transcripts were up-regulated (Fig. 3 and Table S1). For example, genes encoding phosphoenolpyruvate-dependent phosphotransferase systems (PTS) involved in transport of 3-keto-L-gulonate, cellobiose, and glucose, were at least 3-fold up-regulated after 30 min of incubation with blood. After 90 min, more genes or operons were up-regulated relative to time 0 (Table S1), sometimes at high levels (for example, see gbs1936-gbs1939,encoding a mannose/fructose transport system). Of note, genes involved in complex carbohydrate metabolism also were up-regulated, including the maltose-maltodextrin region gbs1507-gbs1508 and gbs1510-gbs1512, gbs1911 (*dexB*) encoding a glucan 1,6-alpha-glucosidase, and gbs1912 (*msmK*) encoding a multiple sugar transport ATP-binding protein. Interestingly, in GAS, homologues of all of these genes are controlled by the same negative regulator (MalR) [27], which is consistent with our findings of a 2.5-fold down-regulation of *malR* (gbs1509). We also found that *fba* (gbs0125), *gapC* (gbs1811) and *eno* (gbs0608), encoding the fructose-bisphosphate aldolase, GAPDH, and enolase, respectively, were up-regulated within the first 30 min of contact with blood. These enzymes are not only involved in glycolysis, but also in virulence. Such a link between complex carbohydrate metabolism and virulence was recently documented in GAS [28], consistent with the fact that we observed up-regulation of the GBS gene encoding catabolite control protein CcpA (3-fold up-regulated at 90 min relative to 30 min).

We identified a significant modification in transcripts of genes encoding proteins involved in purine/pyrimidine metabolism. One set of genes (gbs0023–gbs0027, gbs0029, gbs0042–gbs0044, gbs0047), all encoding enzymes involved in the initial steps of purine metabolism (i.e. transformation of phosphoribosyl pyrophosphate to inosine monophosphate), was down-regulated (Fig. S1). Conversely, the pyrimidine metabolism genes *carA/carB* and *pyrB/pyrC/pyrE/pyrF* (gbs1077–gbs1082), encoding enzymes involved in transformation of glutamine to uracil monophosphate, were up-regulated during the first 30 min of incubation with blood, and then down-regulated (Fig. S1).

Genes encoding amino acid metabolism proteins also were significantly altered, with transcripts of 53 and 60 genes down-regulated after 30 min and 90 min of contact with blood, respectively. This group included genes involved in aspartate, histidine, glutamine, serine, and glycine metabolism. Conversely, very few genes in this category were up-regulated, exceptions being gbs2122–gbs2126, encoding arginine catabolic enzymes, that were up-regulated from 5.5- to 8.6-fold at 90 min relative to time 0 (Table S1).

Genes or operons involved in transport of various ions were down-regulated after contact with blood. For example, *fhuA* and *fhuG* (gbs1462 and gbs1465), *mtsC* and *mtsB* (gbs1587 and gbs1588), and another putative operon (gbs1043–gbs1045), all involved in iron metabolism, were down-regulated from 2.2- to 5.8-fold. Similarly, the genes *adcC* and *adcB* (gbs0151 and gbs0152) involved in zinc uptake, and gbs1679–gbs1680 encoding cobalt transport proteins, were also down-regulated. However, *mts*, *pts,* and gbs1043–gbs1045 subsequently were up-regulated at 90 min relative to 30 min.

### Differential GBS transcription between contact with blood at 40°C and 37°C

In an effort to identify gene transcript changes occurring in conditions that may be especially relevant to human illness, we compared the bacterial transcriptome at a temperature observed in severe infections with high fever (40°C) to the physiological temperature of the human body (37°C). After 30 min of incubation with blood, the GBS transcriptomes obtained from cells grown at 40°C and 37°C were very similar. More transcriptome differences were observed between these two temperatures after 90 min. However, in most cases, the ratio between the transcript levels at 40°C and 37°C was less than 2.

Of interest, the analysis identified up-regulation of several transcripts that may enhance GBS virulence. For example, the genes in the hemolysin operon *cyl* (except gbs0645) were all modestly up-regulated at 40°C relative to 37°C (Table 3). Another proven virulence gene (gbs2000) which encodes the CAMP factor (an extracellular cytolytic protein involved in GBS pathogenesis), and gbs1288 and gbs1420 (*bsp*), each encoding proteins with LPXTG motifs, were also up-regulated at 40°C, respectively (Table 3). Similarly, we observed slightly higher transcript levels of genes implicated in stress response, including four chaperones or heat-shock proteins (gbs0095-*grpE*, gbs0096-*dnaK*, gbs0097-*dnaJ* and gbs0625) and a heat-inducible transcription regulator (gbs0094-*hrcA*). Another gene (gbs1376-*clpL*) encoding another protein involved in stress response was clearly over-expressed at 40°C (Table 3).

**Table 3:** GBS genes of interest that were slightly up-regulated at 40°C relative to 37°C in human blood.

ORF	Gene	Function	Ratio 40°C vs 37°C
gbs0644	*cylX*	hypothetical	1.12
gbs0645	*cylD*	fatty acid biosynthesis enzyme	0.95
gbs0646	*cylG*	fatty acid biosynthesis enzyme	1.18
gbs0647	*acpC*	acyl carrier protein homologue	1.26
gbs0648	*cylZ*	fatty acid biosynthesis enzyme	1.20
gbs0649	*cylA*	ABC transporter	1.36
gbs0650	*cylB*	ABC transporter	1.54
gbs0651	*cylE*	putative hemolysin	1.49
gbs0652	*cylF*	putative aminomethyltransferase	1.64
gbs0653	*cylI*	fatty acid biosynthesis enzyme	1.40
gbs0654	*cylJ*	putative glycosyltransferase	1.71
gbs0655	*cylK*	hypothetical	1.75
gbs2000	*cfb*	CAMP factor	1.74
gbs1288	*-*	LPXTG motif	1.40
gbs1420	*bsp*	LPXTG motif	1.57
gbs0094	*hrcA*	heat-inducible transcription regulator	1.32
gbs0095	*grpE*	heat-shock protein	1.34
gbs0096	*dnaK*	chaperone	1.35
gbs0097	*dnaJ*	chaperone	1.38
gbs0625	*-*	chaperone	1.39
gbs1376	*clpL*	Clp proteinase	4.53

The *cyl* operon, the *cfb* gene encoding the CAMP factor, and two genes encoding proteins with LPXTG motifs may provide advantage to the bacterium during invasive process. Genes encoding proteins involved in stress response were also slightly up-regulated at 40°C suggesting that the adaptive stress response was engaged in GBS.

## Discussion

**Figure 5:**
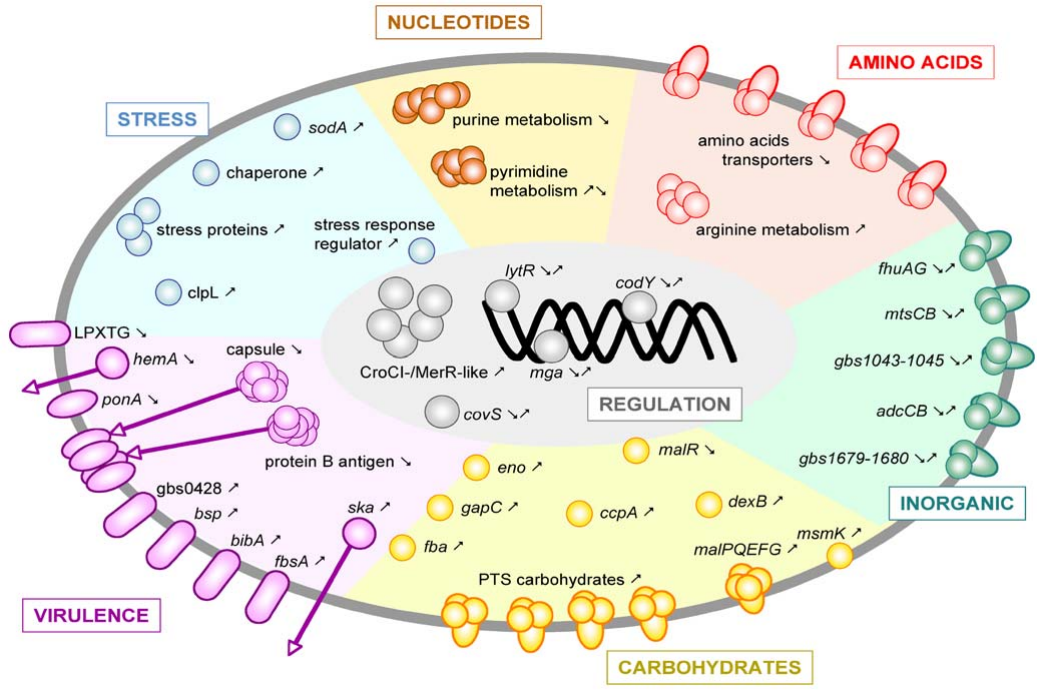
Schematic summarizing the transcriptional response of GBS to human blood. Depicted schematically is a proposed model of GBS gene expression involved in adaptation of the bacterium during *ex vivo* incubation with human blood at 37°C. ORFs of interest are colored according to main functional categories. Arrows indicate up-or down-regulation relative to time 0. When opposite transcription occurs during the first 30 min and the next 60 min, two arrows are shown.

Although studies using animal infection models have provided considerable information about GBS virulence factors and pathogenesis, some processes observed in animals may not occur in humans [29] because human strains may express components that specifically interact with the human host [30]. Ideally, the most relevant data would be provided by analyzing bacterial gene expression during the progress of infections in patients, but this kind of study is extremely difficult to conduct. To circumvent this limitation, and as a first step to better understand the host-bacterium interaction in blood that is a crucial step in most GBS invasive infections, we performed a bacterial transcriptome analysis during GBS incubation *ex vivo* with human blood. The *ex vivo* culture system we used does not perfectly mimic the highly complex environment encountered by the bacterium *in vivo* in human infections. For example, the putative starvation of some nutrients and the lack of continuous oxygenation of the blood, a parameter that contributes to GBS invasiveness and virulence [31], render the duration of the experiment limited in time. In addition, the amount of GBS incubated with each blood sample (∼5×10^8^ CFU/mL, or ∼50 CFU per white blood cell) is higher than commonly present during bacteremia [32], but necessary to obtain a sufficient amount of RNA for the arrays. However, our *ex vivo* experiments mirror numerous conditions existing within infected humans, in particular temperature and the presence of the majority of nutrients and immune system factors. Thus, for the first time, the present investigation provides new data about how GBS remodels its transcriptome in response to human blood. Our data point to strategies implemented by GBS to adapt immediatly after invading the blood (during the first 30 min), and subsequently to establish infection (during the next 60 min) (Fig. 5).

Interestingly, although GAS multiplies rapidly in human blood [13], [33], [34], the number of GBS CFUs did not increase or decrease after 90 min of incubation. The lack of growth could be linked either to an autoregulation phenomenon (for example by a quorum sensing system) due to the high number of bacterial cells already present at time 0 or to an insufficient amount of nutrients (see below). However, this observation can also suggest that GBS needs a longer time than GAS to adapt to blood, an idea consistent with transcriptome data reported from an analogous GAS-blood contact study [13]. Indeed, we observed that most (83%) differentially regulated GBS transcripts were down-regulated in blood (Fig. 3), whereas in striking contrast, in GAS most (62.7%) transcripts were up-regulated [13]. Moreover, in GAS a large number of proven virulence factors were up-regulated [13], but in GBS relatively few virulence genes were up-regulated in blood (Table 2). One hypothesis to explain these observations is that up-regulation of virulence genes in GBS may occur later than in GAS, consistent with the fact that virulence factors are not constitutively expressed but often are activated in the stationary phase of growth [14]. Another explanation is that GAS has more efficient virulence activity in blood (regardless of growth phase), a hypothesis that could bear on the fact that GAS has a higher mortality rate in elderly adults than GBS [2], [35].

Derangements of coagulation and fibrinolysis are common features in patients with severe sepsis [36]. One notable finding of our work was the discovery of up-regulation in human blood of GBS transcripts for proteins involved in interaction with hemostasis system molecules. For example, the gene (*fbsA*) encoding the major GBS receptor for fibrinogen was significantly up-regulated in blood. FbsA potentiates thrombin-catalyzed fibrin clot formation [37]. Notably, a *fbsA* mutant grows poorly in human blood and loses its ability to induce platelet aggregation [38], [39]. Importantly, one-fifth of GBS causing human invasive infections lack the *fbsA* gene, but it is present in all organisms belonging to the hyperinvasive neonatal clone MLST-17 [40]. We also observed up-regulation of three other genes encoding proteins involved in fibrinolysis in other pathogenic streptococcal species (*ska*, *eno,* and *gapC*) [41], [42], [43]. GAS can directly bind plasminogen through enolase and GAPDH, whereas indirect binding requires the formation of a complex with streptokinase, fibrinogen, and plasminogen [44]. Enolase and GAPDH are major GBS outer surface proteins [45], but to date only GAPDH was been shown to interact with human plasminogen [46]. Regardless of the route of activation, plasminogen is converted to plasmin, which in turn, degrades fibrin resulting in release of fibrin degradation products and enhanced dissemination of GBS. Of note, plasmin proteolytic activity cannot be inhibited by α2-antiplasmin, the main physiological inhibitor of plasmin, suggesting that once this enzyme is bound to the bacterial surface it is impervious to at least one normal regulatory mechanism [46]. Thus, our data provide evidence that on exposure to blood GBS enhances transcription of factors that contribute to survival in the blood and dissemination to other anatomic sites.

The transcriptome analysis revealed a large dynamic metabolic adaptation of the bacterium, as more than half of the carbohydrate metabolism genes were up- or down-regulated during incubation with human blood. Although it can be argued that these significant transcript changes occur because GBS is glucose-starved in human blood, which could be the consequence of the high number of bacterial cells present in the sample, several lines of evidence support another hypothesis. First, extensive modifications in carbohydrate gene expression occurred rapidly in the first 30 min of GBS-blood interaction, a time too short to be explained by significant reduction of the blood glucose concentration. Second, numerous expression changes in carbohydrate metabolism genes occurred during a similar experiment with GAS and human blood [13], and also under *in vivo* conditions in humans and non-human primates [47], [48], when nutrients are maintained at physiological levels by the host. Thirdly, regulation of carbohydrate metabolism is crucial during the initial growth and colonization of the bacterium [48]. Several investigators have recently reported that carbohydrate metabolism and virulence are linked in other pathogenic streptococcal species [28], [49], [50], [51]. Thus, we favor the hypothesis that the significant alterations in carbohydrate metabolism genes we observed occur as a fundamental response to the overall host physiologic milieu, rather than glucose starvation. Additional studies are needed to investigate this hypothesis.

We found that only a limited number of transcripts were up-regulated at 40° C relative to 37° C, and most were of a relatively small magnitude. Most transcripts observed to be up-regulated were at 90 min rather than 30 min after blood exposure. Six of the genes up-regulated at 40°C belong either to the chaperone or to class I or class III heat shock families [52], suggesting that GBS is undergoing an adaptive response to the increased temperature of incubation. A homologue of ClpL was the highest up-regulated. In *S. pneumoniae*, ClpL is required for survival after heat shock and it also plays a role in virulence [53], [54]. Our results confirmed previous observations made for GAS that genes implicated in virulence (such as hemolysin) were up-regulated at 40°C [55].

In summary, our study provides the first genome-wide view of the early steps of GBS genetic adaptation to human blood, a crucial step of pathogenesis during invasive disease. We identified an extensive remodeling of the bacterial transcriptome, notable for a down-regulation of numerous genes belonging to all functional categories. It also provides evidence for a rapid and complex response in expression of stress response genes and transcriptional regulators, and underscores the extensive carbohydrate metabolism changes and the high expression of genes involved in contact/activation with coagulation/fibrinolysis networks during blood invasion. Taken together, the data presented here reveal new leads for future studies designed to understand pathogenesis, and to identify additional putative targets that would be helpful to develop new diagnostic and therapeutic strategies.

## Materials and Methods

### Bacterial strain, human blood and growth conditions

The serotype III GBS reference strain NEM316 isolated from a fatal case of invasive infection was used in this study. The genome of strain NEM316 has been sequenced and annotated [52]. Strain NEM316 was grown in Todd Hewitt broth supplemented with 0.2% yeast extract (THY) in 5% CO_2_ at 37°C until the OD_600_ reached 0.75, a value corresponding to mid-logarithmic phase. The bacteria were harvested by centrifugation for 8 min at 4000×g at 37°C, and the cell pellet was suspended in phosphate-buffered saline (PBS).

Fresh heparinized human blood was donated by seven healthy volunteers (four males and three females) in accordance with a protocol approved by the Institutional Review Board of The Methodist Hospital Research Institute. Bacteria were mixed with 80 mL of blood from each donor (final GBS concentration of ∼5×10^8^ CFU/mL of blood) and rotated slightly to avoid sedimentation of blood and bacterial cells. Samples were removed immediately after adding bacteria, and after 30 and 90 min of incubation at 37°C and 40°C.

### RNA isolation

Each blood-bacteria sample was treated with 2 volumes of RNAprotect Bacteria Reagent (Qiagen, Valencia, CA) immediately after collection. The mixture was centrifuged and the pellets were stored at −80°C until processing. The pellets were incubated with 5 volumes of the Erythocyte Lysis (EL) buffer (Qiagen) for 20 min on ice, centrifuged, and rinsed with 2 volumes of EL buffer. RNA was extracted using Lysing Matrix B microtubes containing 0.1 mm silica spheres (Qbiogene, Carlsbad, CA) and 1 mL Trizol (Invitrogen) with the FastPrep FP120 cell disrupter (Qbiogene). RNA extraction was completed with the RNeasy Mini kit (Qiagen) according to the manufacturer's instructions. Samples were treated with DNAFree (Ambion, Austin, TX) to remove trace DNA, and 40 cycles of PCR were performed with RNA templates to document the absence of contaminating genomic DNA. A second treatment with DNAFree was performed if necessary. The RNA concentration was determined by measuring absorbance at 260 and 280 nm, and RNA quality was evaluated by electrophoretic analysis with an Agilent 2100 Bioanalyzer (Agilent Technologies Inc., Palo Alto, CA).

### cDNA synthesis, fragmentation and labeling

The methods used for cDNA synthesis, fragmentation, and labelling have been described extensively elsewhere [56].

### Expression microarray hybridization and analysis

Expression microarray analysis was performed with a custom-made Affymetrix chip formulated based on the genome sequence of strain NEM316 [52]. The chip contains 1,995 probe sets corresponding to the annotated opening reading frames (ORFs) in this genome. Briefly, end-labeled cDNA was hybridized overnight at 40°C using the Affymetrix hybridization and staining modules according to the manufacturer's instructions. Chip hybridization data were acquired and normalized using Affymetrix GeneChip Operating Software (GCOS). Hybridization intensity values were normalized to the mean intensity of all GBS genes present on the chip using GCOS version 1.0 to permit comparison of data obtained from multiple experimental conditions. An ORF was considered to be differentially expressed if there was a “present” signal, if there was a significant (*P*<0.05, T-test) change in expression greater than 2-fold at one time point relative to another and if these criteria were met for at least six of the seven donor samples.

The microarray data have been deposited in the Gene Expression Omnibus database (GSE11705).

### Bioinformatic analyses

Analyses, statistics, and graphics were performed with Partek Pro Genomics Suite 6.0 (Partek, St. Louis, MO), ArrayAssist software v5.5 (Stratagene, La Jolla, CA), and GraphPad Prism v4 (GraphPad Software Inc., San Diego, CA).

## Supporting Information

Figure S1 Kinetics of genes involved in purine/pyrimidine metabolism during incubation with human blood at 37°C. The ‘purine’ genes gbs0023–gbs0027, gbs0029, gbs0042–gbs0044, and gbs0047 (in blue), encoding all the enzymes involved in the first steps of purine metabolism (i.e. transformation of the phosphoribosyl pyrophosphate to the inosine monophasphate), were down-regulated from 2.2- to 7.7-fold. The ‘pyrimidine’ genes gbs1077–gbs1082 (in red), encoding the enzymes involved in the transformation of the glutamine to the uracyl-monophosphate, were up-regulated from 2.2- to 2.5-fold during the first 30 min of contact with blood, and then down-regulated.
(2.65 MB TIF)

Table S1 Microarray expression data from GBS strain NEM316 during incubation with human blood at 37°C and 40°C. Up- and down-regulation after 30 and 90 min of incubation are expressed relatively to time 0. Ratios greater than 2 and less than 0.5 (with *P* value less than 0.05) are highlighted in blue and green, respectively.
(0.50 MB PDF)
