# Supplementary figures and images for: Extensive Adaptive Changes Occur in the Transcriptome of Streptococcus agalactiae (Group B Streptococcus) in Response to Incubation with Human Blood
Source: PLoS One. 2008 Sep 4;3(9):e3143. doi: 10.1371/journal.pone.0003143 (PMC2519835; doi:10.1371/journal.pone.0003143)

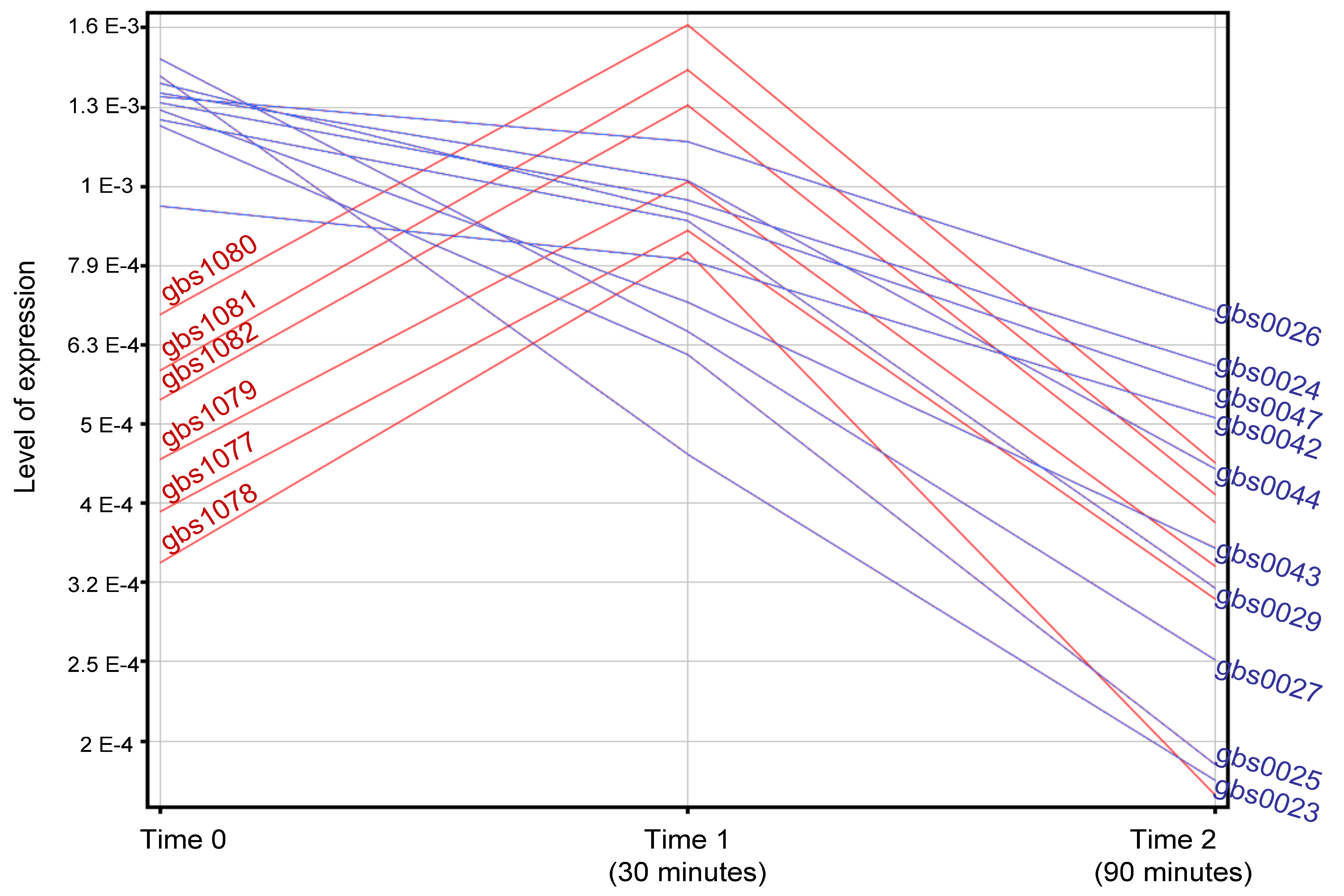

Supplement: Figure S1 — Kinetics of genes involved in purine/pyrimidine metabolism during incubation with human blood at 37°C. The ‘purine’ genes gbs0023–gbs0027, gbs0029, gbs0042–gbs0044, and gbs0047 (in blue), encoding all the enzymes involved in the first steps of purine metabolism (i.e. transformation of the phosphoribosyl pyrophosphate to the inosine monophasphate), were down-regulated from 2.2- to 7.7-fold. The ‘pyrimidine’ genes gbs1077–gbs1082 (in red), encoding the enzymes involved in the transformation of the glutamine to the uracyl-monophosphate, were up-regulated from 2.2- to 2.5-fold during the first 30 min of contact with blood, and then down-regulated. (2.65 MB TIF) [file pone.0003143.s001.tif]
