# Supplementary material for: Extensive Adaptive Changes Occur in the Transcriptome of Streptococcus agalactiae (Group B Streptococcus) in Response to Incubation with Human Blood
Source: PLoS One. 2008 Sep 4;3(9):e3143. doi: 10.1371/journal.pone.0003143 (PMC2519835; doi:10.1371/journal.pone.0003143)
Supplement: Table S1 — Microarray expression data from GBS strain NEM316 during incubation with human blood at 37°C and 40°C. Up- and down-regulation after 30 and 90 min of incubation are expressed relatively to time 0. Ratios greater than 2 and less than 0.5 (with P value less than 0.05) are highlighted in blue and green, respectively. (0.50 MB PDF) [file pone.0003143.s002.pdf]

NCBI ID	COG(s)	Locus	Gene	Functional category	Subcategory	Putative function	37 T1/T0	p T1/T0	37 T2/T0	p T2/T0	40 T1/T0	p T1/T0	40 T2/T0	p T2/T0
1029804	COG0593L	gbs0001	dnaA	Cellular processess	DNA replication, recombination and repair	Chromosomal replication initiator protein dnaA	0,441	0,000	0,673	0,000	0,475	0,000	0,817	0,018
1029808	COG0592L	gbs0002	dnaN	Cellular processess	DNA replication, recombination and repair	DNA polymerase III, beta chain (EC 2.7.7.7)	0,538	0,000	0,818	0,014	0,550	0,000	0,899	0,182
1029799	COG1597IR	gbs0003		Hypothetical		Hypothetical protein	0,614	0,000	0,948	0,760	0,550	0,000	0,794	0,146
1029797	-	gbs0004		Hypothetical		Hypothetical cytosolic protein	0,723	0,033	0,801	0,288	0,620	0,010	0,564	0,005
1029795	-	gbs0005		Hypothetical		Hypothetical protein	0,621	0,002	0,537	0,000	0,587	0,001	0,472	0,000
1030675	COG0012J	gbs0006		Cellular processess	Translation, ribosomal structure and biogenesis	GTP-binding protein, probable translation factor	0,344	0,000	0,630	0,000	0,366	0,000	0,611	0,000
1030987	-	gbs0007	pth	Cellular processess	Translation, ribosomal structure and biogenesis	Peptidyl-tRNA hydrolase (EC 3.1.1.29)	0,057	0,000	0,277	0,003	0,104	0,000	0,109	0,000
1029794	COG1197LK	gbs0008	trcF	Cellular processess	DNA replication, recombination and repair	Transcription-repair coupling factor	0,097	0,001	0,315	0,002	0,165	0,001	0,294	0,002
1029790	COG1188J	gbs0009		Cellular processess	Translation, ribosomal structure and biogenesis	Heat shock protein 15	0,579	0,002	0,216	0,000	0,443	0,000	0,296	0,000
1029787	COG2919D	gbs0010	divIC	Cellular processess	Cell division	Cell division protein DIVIC	0,381	0,000	0,404	0,000	0,373	0,000	0,516	0,001
1030424	-	gbs0011		Hypothetical		Hypothetical protein	0,216	0,000	0,168	0,000	0,163	0,000	0,231	0,000
1029782	COG2367V	gbs0012		Cellular processess	Toxin production and resistance	Beta-lactamase (EC 3.5.2.6)	0,305	0,000	0,387	0,000	0,266	0,000	0,417	0,000
1029802	COG0037D	gbs0013	tilS	Cellular processess	Translation, ribosomal structure and biogenesis	tRNA(Ile)-lysidine synthetase TilS	0,230	0,000	0,468	0,002	0,248	0,000	0,425	0,001
1030991	COG0634F	gbs0014	hpt	Metabolism and transport	Purines, pyrimidines, nucleosides, and nucleotides	Hypoxanthine-guanine phosphoribosyltransferase (EC 2.4.2.8)	0,362	0,000	0,458	0,000	0,361	0,000	0,463	0,000
1030977	COG0465O	gbs0015	ftsH	Cellular processess	Posttranslational modification, protein turnover, chaperones	Cell division protein ftsH (EC 3.4.24.-)	0,475	0,000	0,458	0,000	0,411	0,000	0,438	0,000
1031481	COG3883S, C	gbs0016	pcsB	Cell Envelope	Other	Glucan-binding protein B	0,494	0,000	0,623	0,000	0,518	0,000	0,902	0,088
1031120	COG0462FE	gbs0017	prsA.2	Metabolism and transport	Purines, pyrimidines, nucleosides, and nucleotides	Ribose-phosphate pyrophosphokinase (EC 2.7.6.1)	0,507	0,000	0,682	0,000	0,476	0,000	0,781	0,001
1031047	COG0436E	gbs0018	aspB	Metabolism and transport	Amino acids, peptides, aminosugars and amines	Aromatic amino acid aminotransferase (EC 2.6.1.57) Acetyldiaminopimelate amino	0,621	0,000	0,353	0,000	0,653	0,000	0,388	0,000
1030999	COG1381L	gbs0019	recO	Cellular processess	DNA replication, recombination and repair	DNA repair protein recO	0,315	0,000	0,344	0,000	0,324	0,000	0,380	0,000
1031000	-	gbs0020		Cellular processess	Posttranslational modification, protein turnover, chaperones	CAAX amino terminal protease family	2,765	0,017	9,644	0,016	4,798	0,021	5,225	0,022
1031476	COG0416I	gbs0021	plsX	Metabolism and transport	Fatty acid and phospholipids	Fatty acid phospholipid synthesis protein plsX	0,321	0,000	0,293	0,000	0,294	0,000	0,391	0,000
1031477	COG0236IQ	gbs0022	acpP.2	Metabolism and transport	Fatty acid and phospholipids	Acyl carrier protein	0,260	0,000	0,341	0,000	0,239	0,000	0,376	0,000
1031217	-	gbs0023	purC	Metabolism and transport	Purines, pyrimidines, nucleosides, and nucleotides	Phosphoribosylaminoimidazole-succinocarboxamide synthase (EC 6.3.2.6)	0,333	0,004	0,129	0,001	0,341	0,004	0,071	0,001
1030849	COG0046F, C	gbs0024	purL	Metabolism and transport	Purines, pyrimidines, nucleosides, and nucleotides	Phosphoribosylformylglycinamidine synthase (EC 6.3.5.3)	0,754	0,145	0,466	0,007	0,841	0,310	0,397	0,004
1031315	COG0034F	gbs0025	purF	Metabolism and transport	Purines, pyrimidines, nucleosides, and nucleotides	Amidophosphoribosyltransferase (EC 2.4.2.14)	0,514	0,015	0,156	0,001	0,453	0,009	0,145	0,001
1029830	COG0150F	gbs0026	purM	Metabolism and transport	Purines, pyrimidines, nucleosides, and nucleotides	Phosphoribosylformylglycinamidine cyclo-ligase (EC 6.3.3.1)	0,876	0,425	0,537	0,013	0,881	0,435	0,470	0,007
1030771	-	gbs0027	purN	Metabolism and transport	Purines, pyrimidines, nucleosides, and nucleotides	Phosphoribosylglycinamide formyltransferase (EC 2.1.2.2)	0,452	0,005	0,174	0,001	0,512	0,009	0,149	0,001
1031482	-	gbs0028		Hypothetical		Zwittermicin A resistance protein zmaR	0,472	0,008	0,133	0,001	0,432	0,005	0,077	0,000
1031413	-	gbs0029	purH	Metabolism and transport	Purines, pyrimidines, nucleosides, and nucleotides	Phosphoribosylaminoimidazolecarboxamide formyltransferase (EC 2.1.2.3) IMP cy	0,745	0,160	0,257	0,002	0,815	0,346	0,248	0,002
1031034	COG0739M	gbs0030		Cellular processess	Posttranslational modification, protein turnover, chaperones	Peptidoglycan-specific endopeptidase, M23 family	1,924	0,085	2,376	0,234	1,752	0,369	1,762	0,077
1031483	-	gbs0031	sip	Hypothetical		Surface antigen	0,607	0,000	0,581	0,000	0,634	0,000	0,610	0,000
1029831	COG3010G	gbs0032		Metabolism and transport	Carbohydrates, organic alcohols, and acids	N-acetylmannosamine-6-phosphate 2-epimerase (EC 5.1.3.9)	7,232	0,000	37,112	0,000	8,609	0,004	41,469	0,001
1030981	COG1653G	gbs0033		Metabolism and transport	Carbohydrates, organic alcohols, and acids	N-acetylneuraminate-binding protein	5,145	0,013	34,694	0,000	3,933	0,014	49,495	0,002
1029979	COG1175G	gbs0034		Metabolism and transport	Carbohydrates, organic alcohols, and acids	N-acetylneuraminate transport system permease protein	3,875	0,090	17,158	0,010	3,830	0,037	23,153	0,001
1031470	COG0395G	gbs0035		Metabolism and transport	Carbohydrates, organic alcohols, and acids	N-acetylneuraminate transport system permease protein	8,764	0,021	31,226	0,003	7,067	0,028	50,399	0,002
1031485	-	gbs0036		Hypothetical		Hypothetical cytosolic protein	2,320	0,068	38,130	0,000	2,469	0,158	46,177	0,001
1031486	-	gbs0037		Hypothetical		Hypothetical membrane spanning protein	1,161	0,772	13,539	0,002	1,901	0,293	14,786	0,000
1030355	COG0329EM	gbs0038	nanH	Cell Envelope	Biosynthesis and degradation of surface polysaccharides and lipopo	N-acetylneuraminate lyase (EC 4.1.3.3)	3,584	0,288	26,690	0,004	1,916	0,108	35,211	0,001
1030984	COG1940KG	gbs0039		Cellular processess	Signal transduction	N-acetylmannosamine kinase (EC 2.7.1.60) Transcriptional regulator	2,289	0,077	12,352	0,003	3,118	0,035	16,257	0,001
1031495	COG3458Q	gbs0040		Metabolism and transport	Secondary metabolites	Cephalosporin-C deacetylase (EC 3.1.1.41)	1,967	0,091	8,344	0,001	1,836	0,148	11,158	0,000
1030478	COG1737K	gbs0041		Cellular processess	Transcription	Transcriptional regulator, RpiR family	0,536	0,145	1,629	0,082	0,491	0,113	1,615	0,078
1031720	COG0151F	gbs0042	purD	Metabolism and transport	Purines, pyrimidines, nucleosides, and nucleotides	Phosphoribosylamine--glycine ligase (EC 6.3.4.13)	0,855	0,277	0,541	0,005	0,885	0,372	0,501	0,004
1030779	COG0041F	gbs0043	purE	Metabolism and transport	Purines, pyrimidines, nucleosides, and nucleotides	Phosphoribosylaminoimidazole carboxylase carboxyltransferase subunit (EC 4.1.1.2	0,571	0,016	0,280	0,001	0,611	0,024	0,222	0,001
1030983	COG0026F	gbs0044	purK	Metabolism and transport	Purines, pyrimidines, nucleosides, and nucleotides	Phosphoribosylaminoimidazole carboxylase NCAIR mutase subunit (EC 4.1.1.21)	0,774	0,111	0,335	0,001	0,798	0,150	0,244	0,000
1030998	-	gbs0045		Hypothetical		Hypothetical protein	0,557	0,006	0,207	0,000	0,495	0,003	0,192	0,000
1030996	-	gbs0046		Hypothetical		Hypothetical protein	0,646	0,010	0,292	0,000	0,587	0,005	0,284	0,000
1030994	COG0015F	gbs0047	purB	Metabolism and transport	Purines, pyrimidines, nucleosides, and nucleotides	Adenylosuccinate lyase (EC 4.3.2.2)	0,686	0,009	0,409	0,000	0,679	0,008	0,426	0,000
1030979	-	gbs0048		Cellular processess	Transcription	Transcriptional regulator	0,713	0,043	0,519	0,000	0,591	0,002	0,650	0,003
1030989	COG2255L	gbs0049	ruvB	Cellular processess	DNA replication, recombination and repair	Holliday junction DNA helicase ruvB	0,645	0,007	0,651	0,041	0,605	0,003	0,909	0,463
1030982	COG0394T	gbs0050		Cellular processess	Signal transduction	Protein tyrosine phosphatase (EC 3.1.3.48)	1,067	0,366	0,544	0,000	0,927	0,307	0,536	0,000
1030978	COG4642S	gbs0051		Hypothetical		Hypothetical membrane associated protein	0,802	0,034	0,346	0,000	0,610	0,000	0,451	0,000
1030985	COG1835I	gbs0052		Metabolism and transport	Fatty acid and phospholipids	Acyltransferase family	0,472	0,000	0,528	0,000	0,426	0,000	0,535	0,000
1030824	COG1012C, C	gbs0053	adh2	Metabolism and transport	Energy production and conversion	Alcohol dehydrogenase (EC 1.1.1.1) Acetaldehyde dehydrogenase [acetylating] (E	1,264	0,258	2,805	0,020	1,407	0,137	3,821	0,009
1030992	COG1064R	gbs0054	adhA	Metabolism and transport	Energy production and conversion	Alcohol dehydrogenase (EC 1.1.1.1)	1,649	0,142	5,869	0,000	2,156	0,024	16,402	0,000
1031381	COG0498E	gbs0055	thrC	Metabolism and transport	Amino acids, peptides, aminosugars and amines	Threonine synthase (EC 4.2.3.1)	0,576	0,001	0,546	0,003	0,540	0,002	1,080	0,515
1030757	COG0534V	gbs0056		Cellular processess	Toxin production and resistance	Na+ driven multidrug efflux pump	0,813	0,110	1,703	0,014	0,944	0,488	1,638	0,004
1030755	COG0051J	gbs0057	rpsJ	Cellular processess	Translation, ribosomal structure and biogenesis	SSU ribosomal protein S10P	0,837	0,038	0,805	0,120	0,840	0,121	0,714	0,013
1030743	-	gbs0058	rplC	Cellular processess	Translation, ribosomal structure and biogenesis	LSU ribosomal protein L3P	0,923	0,416	0,839	0,106	0,910	0,354	0,780	0,047
1031368	-	gbs0059	rplD	Cellular processess	Translation, ribosomal structure and biogenesis	LSU ribosomal protein L1E (= L4P)	1,108	0,350	0,828	0,122	1,128	0,212	0,830	0,120
1030756	COG0089J	gbs0060	rplW	Cellular processess	Translation, ribosomal structure and biogenesis	LSU ribosomal protein L23P	1,136	0,312	0,757	0,029	1,098	0,393	0,784	0,049
1030731	COG0090J	gbs0061	rplB	Cellular processess	Translation, ribosomal structure and biogenesis	LSU ribosomal protein L2P	1,014	0,907	0,686	0,012	0,992	0,949	0,650	0,007
1030738	COG0185J	gbs0062	rpsS	Cellular processess	Translation, ribosomal structure and biogenesis	SSU ribosomal protein S19P	1,285	0,032	0,872	0,259	1,321	0,033	0,836	0,124
1030594	-	gbs0063	rplV	Cellular processess	Translation, ribosomal structure and biogenesis	LSU ribosomal protein L22P	1,462	0,007	0,989	0,933	1,467	0,015	0,883	0,347
1031362	COG0092J	gbs0064	rpsC	Cellular processess	Translation, ribosomal structure and biogenesis	SSU ribosomal protein S3P	1,531	0,011	0,962	0,752	1,496	0,006	0,895	0,417
1031364	COG0197J	gbs0065	rplP	Cellular processess	Translation, ribosomal structure and biogenesis	LSU ribosomal protein L16P	1,508	0,008	0,910	0,462	1,455	0,006	0,876	0,250
1031365	-	gbs0066	rpmC	Cellular processess	Translation, ribosomal structure and biogenesis	LSU ribosomal protein L29P	1,532	0,011	0,948	0,693	1,489	0,007	0,891	0,367
1031359	COG0186J	gbs0067	rpsQ	Cellular processess	Translation, ribosomal structure and biogenesis	SSU ribosomal protein S17P	1,214	0,092	0,670	0,006	1,157	0,274	0,632	0,001
1031447	-	gbs0068	rplN	Cellular processess	Translation, ribosomal structure and biogenesis	LSU ribosomal protein L14P	1,569	0,033	0,828	0,118	1,491	0,021	0,793	0,062
1030496	COG0198J	gbs0069	rplX	Cellular processess	Translation, ribosomal structure and biogenesis	LSU ribosomal protein L24P	1,812	0,001	1,026	0,817	1,702	0,003	1,044	0,720
1031361	COG0094J	gbs0070	rplE	Cellular processess	Translation, ribosomal structure and biogenesis	LSU ribosomal protein L5P	1,549	0,007	0,853	0,228	1,599	0,013	0,756	0,052
1031357	COG0199J	gbs0071	rpsN	Cellular processess	Translation, ribosomal structure and biogenesis	SSU ribosomal protein S14P	1,610	0,006	0,926	0,484	1,602	0,003	0,846	0,136
1030668	-	gbs0072	rpsH	Cellular processess	Translation, ribosomal structure and biogenesis	SSU ribosomal protein S8P	1,416	0,024	0,772	0,066	1,339	0,052	0,729	0,032
1031356	COG0097J	gbs0073	rplF	Cellular processess	Translation, ribosomal structure and biogenesis	LSU ribosomal protein L6P	1,613	0,008	0,938	0,604	1,556	0,003	0,853	0,212
1030664	COG0256J	gbs0074	rplR	Cellular processess	Translation, ribosomal structure and biogenesis	LSU ribosomal protein L18P	1,572	0,003	0,999	0,991	1,628	0,001	0,970	0,770
1030663	COG0098J	gbs0075	rpsE	Cellular processess	Translation, ribosomal structure and biogenesis	SSU ribosomal protein S5P	1,734	0,003	0,968	0,749	1,752	0,002	0,886	0,255
1031353	COG1841J	gbs0076	rpmD	Cellular processess	Translation, ribosomal structure and biogenesis	LSU ribosomal protein L30P	1,596	0,010	0,812	0,079	1,601	0,001	0,835	0,141
1031349	COG0200J	gbs0077	rplO	Cellular processess	Translation, ribosomal structure and biogenesis	LSU ribosomal protein L15P	1,592	0,024	0,849	0,233	1,494	0,014	0,960	0,755
1030657	COG0201U	gbs0078	secY	Cellular processess	Posttranslational modification, protein turnover, chaperones	Protein translocase subunit secY	1,236	0,117	0,737	0,034	1,077	0,506	0,720	0,014
1030590	COG0563F	gbs0079	adk	Metabolism and transport	Purines, pyrimidines, nucleosides, and nucleotides	Adenylate kinase (EC 2.7.4.3) Nucleoside-diphosphate kinase (EC 2.7.4.6)	0,774	0,133	0,748	0,153	0,666	0,024	0,582	0,009
1031340	COG0361J	gbs0080	infA	Cellular processess	Translation, ribosomal structure and biogenesis	Bacterial Protein Translation Initiation Factor 1 (IF-1)	1,869	0,000	0,996	0,965	1,620	0,002	0,922	0,427
1031343	COG0257J	gbs0081	rpmJ	Cellular processess	Translation, ribosomal structure and biogenesis	LSU ribosomal protein L36P	2,094	0,000	1,041	0,674	1,939	0,000	0,929	0,504
1030659	COG0099J	gbs0082	rpsM	Cellular processess	Translation, ribosomal structure and biogenesis	SSU ribosomal protein S13P	1,883	0,001	0,898	0,341	1,670	0,003	0,786	0,057
1030596	COG0100J	gbs0083	rpsK	Cellular processess	Translation, ribosomal structure and biogenesis	SSU ribosomal protein S11P	1,547	0,001	1,025	0,816	1,496	0,003	0,959	0,622
1031335	COG0202K	gbs0084	rpoA	Cellular processess	Transcription	DNA-directed RNA polymerase alpha chain (EC 2.7.7.6)	1,227	0,106	0,810	0,054	1,039	0,721	0,755	0,017
1030588	COG0203J	gbs0085	rplQ	Cellular processess	Translation, ribosomal structure and biogenesis	LSU ribosomal protein L17P	0,824	0,042	0,710	0,007	0,756	0,011	0,710	0,006
1030554	-	gbs0086		Hypothetical		Hypothetical protein	0,697	0,349	2,115	0,267	0,404	0,056	0,298	0,021
1031450	-	gbs0087		Hypothetical		Hypothetical protein	0,726	0,246	1,175	0,723	1,439	0,412	0,855	0,717

1031323	-	gbs0088		Hypothetical		Hypothetical protein	1,180	0,174	0,463	0,006	1,158	0,264	0,855	0,488
1030498	-	gbs0089		Hypothetical		Hypothetical protein	3,581	0,001	0,677	0,000	2,439	0,001	0,691	0,001
1031446	-	gbs0090	comX1.1	Cellular processess	DNA transformation	Competence-specific sigma factor ComX	1,344	0,356	14,547	0,025	1,986	0,025	2,458	0,016
1031317	COG0406G	gbs0091		Metabolism and transport	Carbohydrates, organic alcohols, and acids	Phosphoglycerate mutase family protein	1,382	0,000	0,907	0,504	1,384	0,000	0,876	0,208
1030544	-	gbs0092		Cell Envelope	Biosynthesis and degradation of murein sacculus and peptidoglycar	D-alanyl-D-alanine metallocarboxypeptidase (EC 3.4.17.14)	0,613	0,002	0,549	0,001	0,558	0,001	0,670	0,006
1031431	COG1705NU	gbs0093		Cell Envelope	Biosynthesis and degradation of murein sacculus and peptidoglycar	N-acetylmuramoyl-L-alanine amidase (EC 3.5.1.28)	0,389	0,000	0,537	0,000	0,364	0,000	0,537	0,000
1030538	COG1420K	gbs0094	hrcA	Cellular processess	Transcription	Heat-inducible transcription repressor hrcA	0,708	0,251	1,200	0,414	0,887	0,659	1,587	0,049
1031176	COG0576O	gbs0095	grpE	Cellular processess	Posttranslational modification, protein turnover, chaperones	GrpE protein	0,745	0,344	1,159	0,548	0,870	0,611	1,563	0,060
1030530	COG0443O	gbs0096	dnaK	Cellular processess	Posttranslational modification, protein turnover, chaperones	Chaperone protein dnaK	0,862	0,301	0,805	0,155	0,970	0,818	1,087	0,518
1031312	COG0484O	gbs0097	dnaJ	Cellular processess	Posttranslational modification, protein turnover, chaperones	Chaperone protein dnaJ	0,355	0,011	0,247	0,006	0,330	0,010	0,343	0,010
1031314	COG1167KE	gbs0098		Cellular processess	Transcription	Transcriptional regulator, GntR family AMINOTRANSFERASE CLASS-I (EC 2.6.1.-	0,971	0,898	0,623	0,005	1,009	0,970	0,364	0,000
1031307	COG0101J	gbs0099	truA	Cellular processess	Translation, ribosomal structure and biogenesis	tRNA pseudouridine synthase A (EC 4.2.1.70)	0,687	0,002	3,323	0,000	0,719	0,061	3,596	0,000
1030485	COG0351H	gbs0100	thiD	Metabolism and transport	Cofactors, prosthetic groups, and carriers	Phosphomethylpyrimidine kinase (EC 2.7.4.7) Hydroxymethylpyrimidine kinase (EC	0,534	0,001	2,165	0,000	0,583	0,002	2,773	0,000
1030521	COG4720S	gbs0101		Hypothetical		Hypothetical membrane spanning protein	0,496	0,003	2,063	0,006	0,460	0,002	2,365	0,000
1031311	-	gbs0102		Hypothetical		Hypothetical protein	0,358	0,000	2,644	0,001	0,470	0,000	3,412	0,000
1031306	COG0668M	gbs0103		General function predicted only		Mechanosensitive ion channel	0,299	0,001	0,238	0,000	0,282	0,001	0,377	0,001
1031436	COG0544O	gbs0104	tig	Cellular processess	Posttranslational modification, protein turnover, chaperones	Trigger factor, ppiase (EC 5.2.1.8)	0,298	0,000	0,562	0,000	0,342	0,000	0,684	0,001
1031441	COG3343K	gbs0105	rpoE	Cellular processess	Transcription	DNA-directed RNA polymerase delta chain (EC 2.7.7.6)	0,401	0,000	0,393	0,000	0,343	0,000	0,402	0,000
1031303	COG0504F	gbs0106	pyrG	Metabolism and transport	Purines, pyrimidines, nucleosides, and nucleotides	CTP synthase (EC 6.3.4.2)	0,729	0,009	0,471	0,000	0,690	0,019	0,462	0,000
1031300	COG1073R	gbs0107		General function predicted only		Alpha beta hydrolase	0,258	0,000	0,271	0,000	0,233	0,000	0,349	0,001
1030724	-	gbs0108		Metabolism and transport	Purines, pyrimidines, nucleosides, and nucleotides	Deoxyuridine 5 -triphosphate nucleotidohydrolase (EC 3.6.1.23)	0,640	0,001	0,590	0,002	0,590	0,000	0,428	0,000
1031294	COG1066O	gbs0109	radA	Cellular processess	DNA replication, recombination and repair	DNA repair protein RadA	0,267	0,000	0,193	0,000	0,203	0,000	0,224	0,000
1031293	-	gbs0110		Metabolism and transport	Central intermediary metabolism	Carbonic anhydrase (EC 4.2.1.1)	1,277	0,058	0,522	0,002	1,222	0,099	0,322	0,000
1030491	COG1249C	gbs0111		Metabolism and transport	Energy production and conversion	Pyridine nucleotide-disulphide oxidoreductase family protein (EC 1.-.-.-)	0,948	0,513	0,510	0,000	1,028	0,742	0,487	0,000
1031263	COG0008J	gbs0112	gltX	Cellular processess	Translation, ribosomal structure and biogenesis	Glutamyl-tRNA synthetase (EC 6.1.1.17)	0,442	0,000	0,663	0,000	0,431	0,000	0,811	0,006
1031265	COG1879G	gbs0113	rbsB	Metabolism and transport	Carbohydrates, organic alcohols, and acids	D-ribose-binding protein	0,740	0,352	1,554	0,075	0,794	0,450	1,750	0,027
1030717	COG1172G	gbs0114		Metabolism and transport	Carbohydrates, organic alcohols, and acids	Ribose transport system permease protein rbsC	0,200	0,027	0,728	0,431	0,193	0,026	0,956	0,891
1030709	COG1129G	gbs0115		Metabolism and transport	Carbohydrates, organic alcohols, and acids	Ribose transport ATP-binding protein rbsA	1,204	0,571	2,092	0,013	1,216	0,513	2,455	0,002
1030707	COG1869G	gbs0116	rbsD	Metabolism and transport	Carbohydrates, organic alcohols, and acids	D-ribose mutarotase (EC 5.1.3.-)	0,957	0,916	1,781	0,112	1,055	0,892	1,818	0,082
1031255	COG0524G	gbs0117	rbsK	Metabolism and transport	Carbohydrates, organic alcohols, and acids	Ribokinase (EC 2.7.1.15)	1,077	0,872	1,281	0,558	0,923	0,871	1,358	0,472
1031258	COG1609K	gbs0118	rbsR	Cellular processess	Transcription	Ribose operon repressor	3,298	0,002	2,321	0,024	2,990	0,011	2,669	0,009
1030699	COG0577V	gbs0119		Transport and binding proteins	Unknown substrate	ABC transporter permease protein	3,062	0,018	6,567	0,049	2,933	0,119	4,219	0,025
1031253	COG1136V	gbs0120		Transport and binding proteins	Unknown substrate	ABC transporter ATP-binding protein	2,504	0,036	4,451	0,035	4,121	0,043	2,206	0,002
1031250	COG0745TK	gbs0121		Cellular processess	Signal transduction	Two-component response regulator	1,537	0,002	0,731	0,078	1,529	0,002	0,806	0,162
1031246	COG0642T	gbs0122		Cellular processess	Signal transduction	Phosphate regulon sensor protein phoR (EC 2.7.3.-)	0,634	0,001	0,367	0,001	0,404	0,001	0,409	0,001
1031244	COG0137E	gbs0123	argG	Metabolism and transport	Amino acids, peptides, aminosugars and amines	Argininosuccinate synthase (EC 6.3.4.5)	1,508	0,465	0,703	0,379	1,514	0,478	0,581	0,171
1030698	COG0165E	gbs0124	argH	Metabolism and transport	Amino acids, peptides, aminosugars and amines	Argininosuccinate lyase (EC 4.3.2.1)	0,747	0,518	0,232	0,005	0,652	0,359	0,181	0,005
1030788	COG0191G	gbs0125	fba	Metabolism and transport	Carbohydrates, organic alcohols, and acids	Fructose-bisphosphate aldolase (EC 4.1.2.13)	2,160	0,000	1,720	0,000	2,446	0,000	1,661	0,000
1030068	COG0039C	gbs0126		Metabolism and transport	Energy production and conversion	L-2-hydroxyisocaproate dehydrogenase (EC 1.1.1.-)	2,760	0,000	0,799	0,256	2,875	0,000	0,566	0,016
1031241	COG0227J	gbs0127	rpmV	Cellular processess	Translation, ribosomal structure and biogenesis	LSU ribosomal protein L28P	1,627	0,002	1,026	0,851	1,494	0,007	0,843	0,068
1030008	-	gbs0128	asp	Cellular processess	Posttranslational modification, protein turnover, chaperones	General stress protein, Gls24 family	1,576	0,000	0,896	0,098	1,414	0,000	1,010	0,805
1030784	COG1461R	gbs0129		General function predicted only		Dihydroxyacetone kinase family protein	0,654	0,000	0,615	0,000	0,638	0,000	0,767	0,001
1031234	COG0330O	gbs0130		Cellular processess	Posttranslational modification, protein turnover, chaperones	Membrane protease protein family	0,812	0,009	0,566	0,000	0,845	0,051	0,537	0,000
1031237	COG1126E	gbs0131		Metabolism and transport	Amino acids, peptides, aminosugars and amines	Amino acid transport ATP-binding protein	0,554	0,000	0,338	0,000	0,522	0,000	0,294	0,000
1031235	COG0765E, C	gbs0132		Metabolism and transport	Amino acids, peptides, aminosugars and amines	ABC transporter amino acid-binding protein Amino acid ABC transporter permease	0,711	0,003	0,419	0,000	0,685	0,001	0,372	0,000
1031233	COG4907S	gbs0133		Hypothetical		Hypothetical membrane spanning protein	0,800	0,018	0,691	0,005	0,723	0,007	0,773	0,050
1030804	-	gbs0134	uppP	Cellular processess	Toxin production and resistance	Bacitracin resistance protein (Putative undecaprenol kinase) (EC 2.7.1.66)	0,930	0,407	0,535	0,000	0,956	0,570	0,545	0,000
1029996	-	gbs0135	mecA	Cellular processess	Transcription	Negative regulator of genetic competence mecA	0,354	0,000	0,266	0,000	0,347	0,000	0,288	0,000
1031218	COG0472M	gbs0136		Metabolism and transport	Central intermediary metabolism	Undecaprenyl-phosphate alpha-N-acetylglucosaminephosphotransferase (EC 2.7.8.	0,359	0,000	0,325	0,000	0,294	0,000	0,508	0,000
1031016	COG0396O	gbs0137	sufC	Cellular processess	Posttranslational modification, protein turnover, chaperones	ATP-dependent transporter sufC	0,901	0,384	0,611	0,001	0,823	0,056	0,498	0,000
1031214	-	gbs0138	sufD	Metabolism and transport	Cofactors, prosthetic groups, and carriers	SufD protein	0,618	0,000	0,551	0,000	0,667	0,000	0,631	0,000
1031215	COG0520E	gbs0139	nifS3	Metabolism and transport	Amino acids, peptides, aminosugars and amines	Cysteine desulfurase (EC 2.8.1.7) Selenocysteine lyase (EC 4.4.1.16)	0,483	0,000	0,572	0,000	0,430	0,000	0,546	0,000
1031211	COG0822C	gbs0140	nifU	Metabolism and transport	Energy production and conversion	IscU protein	0,376	0,000	0,517	0,000	0,425	0,000	0,605	0,000
1030191	-	gbs0141	sufB	Transport and binding proteins	Unknown substrate	ABC transporter-associated protein sufB	0,458	0,000	0,563	0,000	0,438	0,000	0,653	0,000
1031213	COG1686M	gbs0142		Cell Envelope	Biosynthesis and degradation of murein sacculus and peptidoglycar	D-alanyl-D-alanine serine-type carboxypeptidase (EC 3.4.16.4)	1,152	0,284	1,011	0,954	1,031	0,798	0,866	0,479
1030695	COG1686M	gbs0143	dacA2	Cell Envelope	Biosynthesis and degradation of murein sacculus and peptidoglycar	D-alanyl-D-alanine serine-type carboxypeptidase (EC 3.4.16.4)	0,521	0,000	0,513	0,002	0,442	0,000	0,549	0,000
1030112	COG4166E	gbs0144	oppA	Metabolism and transport	Amino acids, peptides, aminosugars and amines	Oligopeptide-binding protein oppA	1,343	0,010	1,150	0,216	1,408	0,004	1,384	0,007
1030110	COG0601EP	gbs0145		Metabolism and transport	Amino acids, peptides, aminosugars and amines	Oligopeptide transport system permease protein oppB	0,505	0,000	0,513	0,000	0,514	0,000	0,658	0,001
1031444	COG1173EP	gbs0146	oppC	Metabolism and transport	Amino acids, peptides, aminosugars and amines	Oligopeptide transport system permease protein oppC	0,341	0,000	0,422	0,000	0,349	0,000	0,511	0,000
1031206	COG0444EP	gbs0147	oppD	Metabolism and transport	Amino acids, peptides, aminosugars and amines	Oligopeptide transport ATP-binding protein oppD	0,427	0,000	0,497	0,000	0,418	0,000	0,566	0,000
1031432	COG4608E	gbs0148	oppF	Metabolism and transport	Amino acids, peptides, aminosugars and amines	Oligopeptide transport ATP-binding protein oppF	0,525	0,001	0,550	0,001	0,530	0,001	0,681	0,007
1030799	COG1947I	gbs0149		Metabolism and transport	Fatty acid and phospholipids	4-diphosphocytidyl-2-C-methyl-D-erythritol kinase (EC 2.7.1.148)	0,315	0,000	0,512	0,000	0,337	0,000	0,547	0,000
1030844	COG1846K	gbs0150	adcR	Cellular processess	Transcription	Transcriptional regulator, MarR family	0,310	0,000	0,405	0,000	0,224	0,000	0,516	0,000
1031199	COG1121P	gbs0151	adcC	Metabolism and transport	Inorganic ion transport and metabolism	High-affinity zinc uptake system ATP-binding protein znuC	0,350	0,000	0,499	0,000	0,341	0,000	0,666	0,000
1031194	COG1108P	gbs0152	adcB	Metabolism and transport	Inorganic ion transport and metabolism	High-affinity zinc uptake system membrane protein znuB	0,168	0,000	0,295	0,000	0,108	0,000	0,289	0,000
1031196	-	gbs0153		General function predicted only		Streptodornase (EC 3.1.21.1)	1,724	0,106	0,782	0,350	1,604	0,130	0,691	0,123
1031384	COG0162J	gbs0154	tyrS	Cellular processess	Translation, ribosomal structure and biogenesis	Tyrosyl-tRNA synthetase (EC 6.1.1.1)	0,513	0,000	0,531	0,000	0,495	0,000	0,590	0,000
1031193	COG0744M	gbs0155		Cell Envelope	Biosynthesis and degradation of murein sacculus and peptidoglycar	Multimodular transpeptidase-transglycosylase PBP 1B (EC 2.4.1.129)	0,614	0,000	0,854	0,190	0,559	0,001	0,978	0,791
1031385	COG0085K	gbs0156	rpoB	Cellular processess	Transcription	DNA-directed RNA polymerase beta chain (EC 2.7.7.6)	0,524	0,000	0,844	0,086	0,537	0,000	0,813	0,022
1031192	COG0086K	gbs0157	rpoC	Cellular processess	Transcription	DNA-directed RNA polymerase beta chain (EC 2.7.7.6)	0,592	0,001	0,846	0,087	0,635	0,001	0,918	0,370
1031191	-	gbs0158		General function predicted only		Putative DNA binding protein	0,857	0,161	0,581	0,001	0,810	0,068	0,439	0,000
1030692	COG2804NU	gbs0159	comYA	Cellular processess	DNA transformation	ComG operon protein 1	5,844	0,024	10,223	0,019	6,096	0,155	4,216	0,000
1030286	COG1459NU	gbs0160	comYB	Cellular processess	DNA transformation	ComG operon protein 2	4,219	0,007	10,257	0,007	4,115	0,006	6,178	0,004
1031190	COG4537U	gbs0161	comYC	Cellular processess	DNA transformation	ComG operon protein 3	3,116	0,051	3,803	0,008	3,290	0,006	2,521	0,016
1030569	COG2165NU	gbs0162		Cellular processess	DNA transformation	ComG operon protein 4	4,247	0,035	14,296	0,044	4,107	0,002	10,740	0,054
1031678	COG2165NU	gbs0163		Cellular processess	DNA transformation	ComG operon protein 5	4,194	0,080	4,149	0,023	3,992	0,086	3,091	0,091
1031456	COG4940U	gbs0164	comYD	Cellular processess	DNA transformation	ComG operon protein 6	2,308	0,134	3,917	0,051	2,776	0,079	4,690	0,061
1031182	-	gbs0165		Cellular processess	DNA transformation	ComG operon protein 6	2,831	0,067	11,475	0,105	4,456	0,004	4,841	0,009
1030503	COG0827L	gbs0166		Cellular processess	DNA replication, recombination and repair	Adenine-specific methyltransferase (EC 2.1.1.72)	0,324	0,000	0,110	0,000	0,230	0,000	0,311	0,000
1031180	COG0282C	gbs0167	ackA	Metabolism and transport	Energy production and conversion	Acetate kinase (EC 2.7.2.1)	0,569	0,000	0,940	0,421	0,568	0,000	0,986	0,813
1030243	COG1476K	gbs0168		Cellular processess	Transcription	Transcriptional regulator, Cro CI family	0,575	0,000	0,381	0,000	0,457	0,000	0,508	0,000
1031383	-	gbs0169		Hypothetical		Hypothetical protein	0,427	0,000	0,493	0,026	0,383	0,000	0,479	0,000
1031177	-	gbs0170		Cellular processess	Posttranslational modification, protein turnover, chaperones	CAAX amino terminal protease family	0,598	0,000	0,459	0,000	0,585	0,000	0,530	0,000
1030209	COG0345E	gbs0171	proC	Metabolism and transport	Amino acids, peptides, aminosugars and amines	Pyrroline-5-carboxylate reductase (EC 1.5.1.2)	0,434	0,000	0,349	0,000	0,384	0,000	0,459	0,000
1030208	COG1363G	gbs0172	pepA	Metabolism and transport	Carbohydrates, organic alcohols, and acids	Glutamyl aminopeptidase (EC 3.4.11.7)	0,753	0,013	0,612	0,000	0,690	0,001	0,732	0,003
1031171	-	gbs0173		Hypothetical		Hypothetical protein	0,301	0,000	0,372	0,000	0,263	0,000	0,425	0,000
1031679	-	gbs0174		Hypothetical		Hypothetical membrane associated protein	0,302	0,000	0,389	0,000	0,223	0,000	0,531	0,000
1030857	COG0526OC	gbs0175		Metabolism and transport	Energy production and conversion	Thioredoxin	0,302	0,000	0,370	0,000	0,269	0,000	0,421	0,000

1030174	COG0073R	gbs0176		General function predicted only		tRNA binding domain protein	0,571	0,000	0,562	0,000	0,580	0,000	0,483	0,000
1031169	COG0500QR	gbs0177		Metabolism and transport	Secondary metabolites	Methyltransferase (EC 2.1.1.-)	0,810	0,291	0,574	0,009	0,691	0,070	0,433	0,003
1030805	COG0629L	gbs0178		Cellular processess	DNA replication, recombination and repair	Single-strand DNA binding protein	13,562	0,000	10,234	0,096	7,841	0,012	3,091	0,000
1030840	COG0637R	gbs0179		Metabolism and transport	Carbohydrates, organic alcohols, and acids	Beta-phosphoglucomutase (EC 5.4.2.6) Glucose-1-phosphate phosphodismutase (	1,410	0,044	1,042	0,870	1,058	0,790	1,084	0,685
1031387	COG3275T	gbs0180		Cellular processess	Signal transduction	Autolysin sensor kinase (EC 2.7.3.-)	0,588	0,031	1,278	0,355	0,668	0,162	1,020	0,930
1029880	COG3279KT	gbs0181		Cellular processess	Transcription	Autolysin response regulator	1,387	0,091	1,944	0,003	1,254	0,158	2,207	0,009
1031167	COG1380R	gbs0182		Cell Envelope	Biosynthesis and degradation of murein sacculus and peptidoglycar	Murein hydrolase exporter	8,338	0,013	148,485	0,001	12,617	0,012	23,498	0,000
1030827	-	gbs0183		Cell Envelope	Biosynthesis and degradation of murein sacculus and peptidoglycar	Murein hydrolase export regulator	4,845	0,000	74,021	0,000	7,016	0,001	16,217	0,000
1031166	COG0747E	gbs0184	dppA	Metabolism and transport	Amino acids, peptides, aminosugars and amines	Dipeptide-binding protein	1,090	0,440	0,418	0,000	0,977	0,886	0,379	0,000
1030990	COG0601EP	gbs0185	dppB	Metabolism and transport	Amino acids, peptides, aminosugars and amines	Dipeptide transport system permease protein dppB	0,521	0,005	0,593	0,069	0,365	0,000	0,319	0,000
1030966	COG1173EP	gbs0186	dppC	Metabolism and transport	Amino acids, peptides, aminosugars and amines	Dipeptide transport system permease protein dppC	0,294	0,000	0,546	0,100	0,230	0,000	0,248	0,000
1031164	COG0444EP	gbs0187	dppD	Metabolism and transport	Amino acids, peptides, aminosugars and amines	Dipeptide transport ATP-binding protein dppD	0,362	0,000	0,455	0,000	0,455	0,004	0,304	0,000
1030832	COG1124EP	gbs0188	dppE	Metabolism and transport	Amino acids, peptides, aminosugars and amines	Dipeptide transport ATP-binding protein dppF	0,355	0,001	0,598	0,248	0,276	0,000	0,533	0,025
1031157	COG1263G	gbs0189		Metabolism and transport	Carbohydrates, organic alcohols, and acids	PTS system, trehalose-specific IIBC component (EC 2.7.1.69) PTS system, trehalc	1,154	0,708	3,572	0,000	1,054	0,893	2,829	0,001
1031158	-	gbs0190	dexS	Metabolism and transport	Carbohydrates, organic alcohols, and acids	Trehalose-6-phosphate hydrolase (EC 3.2.1.93)	0,500	0,103	1,975	0,008	0,525	0,124	2,039	0,006
1031156	COG1762GT,	gbs0191		Cellular processess	Transcription	Transcription antiterminator, BglG family PTS system, mannitol (Cryptic)-specific II/	4,128	0,002	18,804	0,016	7,897	0,007	16,538	0,031
1030573	COG3414G	gbs0192		Metabolism and transport	Carbohydrates, organic alcohols, and acids	PTS SYSTEM, IIB COMPONENT (EC 2.7.1.69)	3,363	0,000	9,648	0,014	4,171	0,002	9,050	0,018
1029929	-	gbs0193	ulaA	Transport and binding proteins	Unknown substrate	Putative transport protein sgaT	2,783	0,014	11,238	0,007	3,599	0,002	12,019	0,078
1030740	COG3959G	gbs0194		Metabolism and transport	Carbohydrates, organic alcohols, and acids	Transketolase (EC 2.2.1.1)	2,643	0,150	14,385	0,034	3,056	0,019	16,363	0,039
1031454	COG3958G	gbs0195		Metabolism and transport	Carbohydrates, organic alcohols, and acids	Transketolase (EC 2.2.1.1)	1,044	0,888	9,831	0,020	0,965	0,903	10,197	0,045
1031153	COG4097P	gbs0196		Metabolism and transport	Inorganic ion transport and metabolism	Vanillate O-demethylase oxidoreductase (EC 1.14.13.-)	6,139	0,000	3,499	0,017	5,374	0,000	2,825	0,032
1031150	-	gbs0197	rpsO	Cellular processess	Translation, ribosomal structure and biogenesis	SSU ribosomal protein S15P	0,627	0,001	0,560	0,000	0,650	0,003	0,594	0,000
1031145	COG1185J	gbs0198	pnpA	Cellular processess	Translation, ribosomal structure and biogenesis	Polyribonucleotide nucleotidyltransferase (EC 2.7.7.8) Polynucleotide adenylyltrans	0,273	0,000	0,408	0,000	0,249	0,000	0,443	0,000
1030002	-	gbs0199		Hypothetical		Hypothetical protein	0,250	0,000	0,434	0,000	0,288	0,000	0,542	0,000
1031138	COG1045E	gbs0200	cysE	Metabolism and transport	Amino acids, peptides, aminosugars and amines	Serine acetyltransferase (EC 2.3.1.30)	0,177	0,000	0,337	0,000	0,176	0,000	0,360	0,000
1031141	-	gbs0201		Hypothetical		Hypothetical protein	0,219	0,000	0,414	0,000	0,232	0,000	0,442	0,000
1031132	COG0215J	gbs0202	cysS	Cellular processess	Translation, ribosomal structure and biogenesis	Cysteinyl-tRNA synthetase (EC 6.1.1.16)	0,264	0,000	0,416	0,000	0,247	0,000	0,488	0,000
1031126	COG1939S	gbs0203		Hypothetical		Hypothetical protein	0,315	0,000	0,432	0,000	0,326	0,000	0,497	0,000
1031127	COG0566J	gbs0204		Cellular processess	Translation, ribosomal structure and biogenesis	23S rRNA Gm2251 methyltransferase (EC 2.1.1.-)	0,906	0,276	0,710	0,013	0,692	0,014	0,817	0,030
1031004	-	gbs0205		Hypothetical		Hypothetical cytosolic protein	0,922	0,384	0,935	0,586	0,846	0,229	0,991	0,902
1030748	COG1307S	gbs0206		Metabolism and transport	Fatty acid and phospholipids	Fatty acid-binding protein, DegV family	0,512	0,000	0,577	0,000	0,505	0,000	0,507	0,000
1030907	COG1476K	gbs0207		Cellular processess	Transcription	Transcriptional regulator, Cro CI family	2,385	0,095	1,140	0,668	1,239	0,436	0,768	0,454
1030906	COG2826L	gbs0208		Mobile and extrachromosomal elements		Transposase	1,508	0,019	1,719	0,112	1,346	0,144	2,797	0,000
1030745	-	gbs0209	rplM	Cellular processess	Translation, ribosomal structure and biogenesis	LSU ribosomal protein L13P	0,821	0,033	0,659	0,001	0,714	0,006	0,640	0,001
1031664	-	gbs0210	rpsI	Cellular processess	Translation, ribosomal structure and biogenesis	SSU ribosomal protein S9P	0,946	0,266	0,732	0,021	0,998	0,977	0,699	0,002
1031123	COG0582L	gbs0211	int.1	Mobile and extrachromosomal elements		DNA integration recombination inversion protein	1,043	0,781	1,055	0,780	1,313	0,028	1,453	0,048
1031018	-	gbs0212		Hypothetical		Hypothetical protein	1,035	0,765	0,802	0,154	1,079	0,609	0,998	0,988
1030061	-	gbs0213		Hypothetical		Hypothetical protein	1,867	0,120	4,673	0,083	1,444	0,388	5,179	0,116
1030798	-	gbs0214		Hypothetical		Hypothetical protein	2,291	0,005	3,403	0,005	2,555	0,020	4,510	0,001
1031124	-	gbs0215		Hypothetical		Hypothetical protein	1,528	0,366	6,555	0,047	3,318	0,015	1,990	0,127
1030509	COG1393P	gbs0216		Hypothetical		Hypothetical protein	2,019	0,139	3,160	0,048	0,997	0,993	1,525	0,208
1031448	-	gbs0217		General function predicted only		Replication initiation protein, RepA family	2,966	0,044	3,588	0,026	2,220	0,146	4,213	0,008
1030104	-	gbs0218		Hypothetical		Hypothetical protein	2,414	0,160	2,838	0,205	1,065	0,882	2,648	0,092
1031125	COG1196D	gbs0219		Mobile and extrachromosomal elements		Plasmid recombination protein, Mob family	2,410	0,008	3,809	0,001	3,006	0,000	4,919	0,000
1029946	-	gbs0220		Hypothetical		Hypothetical protein	0,766	0,338	1,718	0,247	0,715	0,286	1,044	0,858
1030479	-	gbs0221		Hypothetical		Hypothetical cytosolic protein	1,972	0,000	1,512	0,002	2,292	0,000	1,199	0,217
1030793	-	gbs0222		Cellular processess	DNA replication, recombination and repair	DNA-damage-inducible protein J	2,523	0,000	1,253	0,063	2,666	0,000	1,095	0,680
1030797	COG4842S	gbs0223		Hypothetical		Hypothetical protein	1,798	0,000	0,512	0,023	1,588	0,006	0,627	0,047
1030450	-	gbs0224		Hypothetical		Hypothetical protein	2,896	0,000	1,619	0,027	2,519	0,000	1,439	0,002
1031146	-	gbs0225		Cellular processess	DNA replication, recombination and repair	Hypothetical protein	1,515	0,012	0,403	0,000	1,294	0,078	0,401	0,000
1031149	-	gbs0226		Hypothetical		Hypothetical protein	1,134	0,182	0,841	0,167	0,999	0,996	0,648	0,003
1030786	-	gbs0228		Hypothetical		Hypothetical protein	0,851	0,237	0,241	0,000	0,837	0,348	0,395	0,000
1031152	-	gbs0229		Hypothetical		Hypothetical protein	0,955	0,595	0,519	0,003	0,738	0,000	0,522	0,000
1029978	-	gbs0230	mutR	Cellular processess	Transcription	Transcriptional regulator	2,845	0,000	0,537	0,005	2,298	0,000	0,675	0,093
1030927	-	gbs0231	mefE	Cellular processess	Toxin production and resistance	Macrolide-efflux protein	1,736	0,010	1,046	0,823	1,193	0,303	0,583	0,081
1031154	COG1174E	gbs0232		Metabolism and transport	Amino acids, peptides, aminosugars and amines	Glycine betaine carnitine choline transport system permease protein opuCD	0,392	0,000	0,528	0,000	0,433	0,000	0,449	0,000
1031424	COG1732M	gbs0233		Metabolism and transport	Amino acids, peptides, aminosugars and amines	Glycine betaine carnitine choline-binding protein	0,581	0,000	0,517	0,000	0,631	0,000	0,641	0,000
1031155	COG1174E	gbs0234		Metabolism and transport	Amino acids, peptides, aminosugars and amines	Glycine betaine carnitine choline transport system permease protein opuCB	0,473	0,000	0,317	0,000	0,396	0,000	0,489	0,001
1030037	COG1125E	gbs0235		Metabolism and transport	Amino acids, peptides, aminosugars and amines	Glycine betaine carnitine choline transport ATP-binding protein opuCA	0,735	0,005	0,515	0,000	0,809	0,019	0,641	0,000
1030836	-	gbs0236		Hypothetical		Hypothetical protein	1,422	0,006	1,218	0,184	1,452	0,025	0,876	0,288
1031440	COG0582L	gbs0237		Mobile and extrachromosomal elements		DNA integration recombination inversion protein	0,969	0,767	1,096	0,595	1,081	0,696	1,508	0,004
1031168	-	gbs0238		Hypothetical		Hypothetical protein	0,806	0,130	0,978	0,937	0,739	0,077	1,291	0,046
1031172	-	gbs0239		General function predicted only		Rep protein	0,880	0,242	1,830	0,105	1,055	0,839	1,509	0,091
1031173	-	gbs0240		Hypothetical		Hypothetical protein	1,798	0,002	0,881	0,339	1,787	0,004	1,113	0,326
1031453	COG1674D	gbs0241		Cellular processess	Cell division	FtsK SpoIIIE family	1,675	0,004	0,730	0,093	1,566	0,003	1,219	0,193
1031174	-	gbs0242		Hypothetical		Hypothetical protein	1,519	0,001	0,574	0,000	1,602	0,020	0,903	0,250
1030826	-	gbs0243		Hypothetical		Hypothetical protein	1,757	0,007	1,123	0,523	1,852	0,005	1,228	0,211
1030232	-	gbs0244		Hypothetical		Hypothetical protein	0,836	0,190	1,235	0,312	0,803	0,211	1,251	0,182
1031189	COG1670J	gbs0245		Metabolism and transport	Central intermediary metabolism	Acetyltransferase (EC 2.3.1.-)	1,410	0,005	0,730	0,052	1,118	0,304	0,840	0,150
1031175	COG1670J	gbs0246		Cellular processess	Translation, ribosomal structure and biogenesis	Ribosomal-protein-alanine acetyltransferase (EC 2.3.1.128)	1,116	0,260	0,578	0,001	0,965	0,735	0,793	0,041
1030934	COG1670J	gbs0247		Cellular processess	Translation, ribosomal structure and biogenesis	Ribosomal-protein-serine acetyltransferase (EC 2.3.1.-)	0,757	0,033	0,558	0,002	0,705	0,016	0,708	0,016
1031386	-	gbs0248		general function predicted only		ECF-type sigma factor negative effector	1,838	0,008	1,188	0,561	1,245	0,333	0,765	0,270
1030245	COG1595K	gbs0249		Cellular processess	Transcription	RNA polymerase ECF-type sigma factor	4,036	0,001	0,658	0,213	1,760	0,244	1,139	0,787
1031184	COG1309K	gbs0250		Cellular processess	Transcription	Transcriptional regulator, TetR family	0,352	0,000	2,507	0,320	1,094	0,809	0,850	0,559
1031185	COG0842V	gbs0251	sagI	Transport and binding proteins	Unknown substrate	ABC transporter permease protein	2,409	0,149	3,805	0,085	1,618	0,376	3,394	0,045
1031420	COG1131V	gbs0252	sagG	Cellular processess	Toxin production and resistance	Daunorubicin resistance ATP-binding protein drrA	1,881	0,143	5,845	0,082	3,583	0,013	4,132	0,102
1031187	-	gbs0253		Hypothetical		Hypothetical protein	0,738	0,040	0,790	0,029	0,670	0,002	0,774	0,033
1031382	COG1695K	gbs0254		Cellular processess	Transcription	Transcriptional regulator, PadR family	0,358	0,000	0,389	0,001	0,224	0,000	0,565	0,002
1030359	-	gbs0255		Hypothetical		Hypothetical protein	1,828	0,002	1,361	0,105	1,704	0,015	1,289	0,004
1031426	COG1820G	gbs0256	nagA	Metabolism and transport	Carbohydrates, organic alcohols, and acids	N-acetylglucosamine-6-phosphate deacetylase (EC 3.5.1.25)	1,004	0,958	0,611	0,000	0,876	0,058	0,870	0,049
1030358	-	gbs0257		Hypothetical		Hypothetical membrane spanning protein	0,844	0,279	0,612	0,029	0,691	0,054	0,478	0,006
1030838	-	gbs0258	glyQ	Cellular processess	Translation, ribosomal structure and biogenesis	Glycyl-tRNA synthetase alpha chain (EC 6.1.1.14)	0,525	0,003	0,453	0,001	0,522	0,003	0,426	0,001
1031429	COG1182I	gbs0259		Metabolism and transport	Fatty acid and phospholipids	FMN-dependent NADH-azoreductase (EC 1.6.99.2)	0,359	0,000	0,330	0,000	0,315	0,000	0,339	0,000
1031195	COG0751J	gbs0260	glyS	Cellular processess	Translation, ribosomal structure and biogenesis	Glycyl-tRNA synthetase beta chain (EC 6.1.1.14)	0,510	0,003	0,470	0,001	0,568	0,005	0,540	0,004
1030775	-	gbs0261		Hypothetical		Hypothetical cytosolic protein	0,561	0,005	0,361	0,001	0,626	0,009	0,397	0,001
1030809	-	gbs0262		Hypothetical		Hypothetical membrane spanning protein	0,456	0,001	0,669	0,040	0,546	0,002	0,551	0,003
1030693	COG0554C	gbs0263	glpK	Metabolism and transport	Energy production and conversion	Glycerol kinase (EC 2.7.1.30)	2,920	0,000	10,711	0,001	3,441	0,000	9,690	0,001
1031442	COG0578C	gbs0264	glpD	Metabolism and transport	Energy production and conversion	Alpha-glycerophosphate oxidase (EC 1.1.3.21)	1,145	0,661	11,082	0,001	1,818	0,053	11,387	0,001

1030430	-	gbs0265	glpF	Metabolism and transport	Carbohydrates, organic alcohols, and acids	Glycerol uptake facilitator protein	1,026	0,910	10,506	0,000	1,646	0,038	9,881	0,000
1031204	COG0446R	gbs0266		Metabolism and transport	Central intermediary metabolism	NADH peroxidase (EC 1.11.1.1)	0,506	0,058	3,072	0,000	0,439	0,038	4,695	0,000
1030416	-	gbs0267	mga	Hypothetical		trans-acting positive regulator	0,337	0,006	3,249	0,000	0,477	0,023	4,871	0,000
1030442	COG0021G	gbs0268	tkt	Metabolism and transport	Carbohydrates, organic alcohols, and acids	Transketolase (EC 2.2.1.1)	0,955	0,740	1,249	0,050	0,929	0,551	1,537	0,002
1030385	-	gbs0269		Hypothetical		Hypothetical protein	0,439	0,000	0,730	0,008	0,365	0,000	0,895	0,239
1031207	COG1131V	gbs0270		Transport and binding proteins	Unknown substrate	ABC transporter ATP-binding protein	0,685	0,001	0,960	0,710	0,655	0,001	1,037	0,732
1030079	-	gbs0271		Transport and binding proteins	Unknown substrate	ABC transporter permease protein	0,375	0,000	0,668	0,006	0,358	0,000	0,928	0,486
1031209	COG1263G	gbs0272		Metabolism and transport	Carbohydrates, organic alcohols, and acids	PTS system, beta-glucoside-specific IIABC component (EC 2.7.1.69)	1,228	0,431	2,338	0,004	0,897	0,710	1,429	0,122
1031210	COG0263E	gbs0273	proB	Metabolism and transport	Amino acids, peptides, aminosugars and amines	Glutamate 5-kinase (EC 2.7.2.11)	0,446	0,000	0,275	0,000	0,361	0,000	0,475	0,001
1031437	COG0014E	gbs0274	proA	Metabolism and transport	Amino acids, peptides, aminosugars and amines	Gamma-glutamyl phosphate reductase (EC 1.2.1.41)	0,514	0,000	0,580	0,001	0,461	0,000	0,652	0,001
1031208	-	gbs0275	mraW	General function predicted only		S-adenosyl-methyltransferase mraW (EC 2.1.1.-)	0,159	0,000	0,283	0,000	0,114	0,000	0,404	0,000
1030691	-	gbs0276	ftsL	Cellular processess	Cell division	Cell division protein ftsL	0,097	0,000	0,211	0,000	0,069	0,000	0,283	0,000
1031219	COG0768M	gbs0277		Cell Envelope	Biosynthesis and degradation of murein sacculus and peptidoglycar	Division specific D,D-transpeptidase Cell division protein ftsI	0,319	0,000	0,677	0,007	0,384	0,000	0,650	0,003
1030464	COG0472M	gbs0278	mraY	Cell Envelope	Biosynthesis and degradation of murein sacculus and peptidoglycar	Phospho-N-acetylmuramoyl-pentapeptide-transferase (EC 2.7.8.13)	0,231	0,000	0,419	0,000	0,248	0,000	0,410	0,000
1031649	COG0513LKJ	gbs0279		Cellular processess	DNA replication, recombination and repair	ATP-dependent RNA helicase	1,297	0,169	0,818	0,176	1,369	0,047	0,619	0,001
1031220	COG0834ET	gbs0280		Transport and binding proteins	Unknown substrate	ABC transporter substrate-binding protein	0,896	0,471	0,617	0,020	0,769	0,101	0,544	0,006
1030039	COG0765E	gbs0281		Metabolism and transport	Amino acids, peptides, aminosugars and amines	Amino acid ABC transporter permease protein	0,826	0,184	0,396	0,000	0,691	0,020	0,351	0,000
1030231	COG1126E	gbs0282		Metabolism and transport	Amino acids, peptides, aminosugars and amines	Amino acid transport ATP-binding protein	0,515	0,001	0,345	0,000	0,501	0,000	0,324	0,000
1030089	-	gbs0283	aapA	Hypothetical		Hypothetical membrane associated protein	0,774	0,018	0,818	0,099	0,589	0,001	0,968	0,659
1030075	COG0492O	gbs0284	trxB	Metabolism and transport	Energy production and conversion	Thioredoxin reductase (EC 1.8.1.9)	1,083	0,153	0,984	0,777	1,018	0,715	0,937	0,382
1030696	COG1488H	gbs0285	nadE	Metabolism and transport	Cofactors, prosthetic groups, and carriers	Nicotinate phosphoribosyltransferase (EC 2.4.2.11)	0,554	0,000	0,375	0,000	0,516	0,000	0,364	0,000
1031238	COG0171H	gbs0286	nadE	Metabolism and transport	Cofactors, prosthetic groups, and carriers	NH(3)-dependent NAD(+) synthetase (EC 6.3.5.1)	0,680	0,000	0,539	0,000	0,657	0,000	0,576	0,000
1030350	COG3579E	gbs0287	pepC	Metabolism and transport	Amino acids, peptides, aminosugars and amines	Aminopeptidase C (EC 3.4.22.40)	1,433	0,002	0,726	0,012	1,398	0,014	0,723	0,001
1031240	COG0744M	gbs0288	ponA	Cell Envelope	Biosynthesis and degradation of murein sacculus and peptidoglycar	Multimodular transpeptidase-transglycosylase PBP 1A	0,457	0,000	0,693	0,001	0,496	0,000	0,908	0,118
1031247	COG3331R	gbs0289	recU	Cellular processess	DNA replication, recombination, and repair	Recombination protein recU	0,254	0,000	0,560	0,000	0,213	0,000	0,645	0,000
1031242	-	gbs0290		Hypothetical		Hypothetical cytosolic protein	0,805	0,122	1,750	0,031	0,881	0,569	1,635	0,001
1030697	COG3599D	gbs0291		Cellular processess	Cell division	Cell division initiation protein DivIVA	0,572	0,000	0,425	0,000	0,544	0,000	0,490	0,000
1031245	COG0116L	gbs0292		Metabolism and transport	Central intermediary metabolism	Methyltransferase (EC 2.1.1.-)	0,389	0,000	0,423	0,000	0,401	0,000	0,525	0,000
1031248	-	gbs0293		Hypothetical		Hypothetical protein	0,414	0,000	0,467	0,000	0,412	0,000	0,614	0,000
1030700	-	gbs0294	luxS	Cellular processess	Signal transduction	Autoinducer-2 production protein luxS (EC 3.13.1.-)	0,910	0,186	0,649	0,000	0,815	0,050	0,852	0,119
1031251	COG1418R	gbs0295		Metabolism and transport	Central intermediary metabolism	Hydrolase (HAD superfamily)	0,435	0,000	0,671	0,001	0,435	0,000	0,731	0,007
1031256	COG1131V	gbs0296		Transport and binding proteins	Unknown substrate	ABC transporter ATP-binding protein	2,618	0,016	1,281	0,549	2,853	0,001	1,731	0,100
1031254	COG0842V	gbs0297		Transport and binding proteins	Unknown substrate	ABC transporter permease protein	1,633	0,122	1,626	0,221	1,509	0,038	1,348	0,428
1031261	COG4585T	gbs0298		Cellular processess	Signal transduction	Sensory Transduction Histidine Kinase (EC 2.7.3.-)	0,754	0,116	1,128	0,345	0,977	0,862	1,163	0,345
1030718	COG2197TK	gbs0299		Cellular processess	Signal transduction	Two-component response regulator	0,581	0,000	0,724	0,129	0,399	0,000	0,507	0,000
1030702	-	gbs0300		Hypothetical		Hypothetical membrane spanning protein	0,671	0,014	1,171	0,402	0,707	0,053	0,871	0,292
1031266	COG0194F	gbs0301	gmk	Metabolism and transport	Purines, pyrimidines, nucleosides, and nucleotides	Guanylate kinase (EC 2.7.4.8)	0,219	0,000	0,363	0,000	0,207	0,000	0,472	0,000
1031267	COG1758K	gbs0302		Cellular processess	Transcription	DNA-directed RNA polymerase omega chain (EC 2.7.7.6)	0,417	0,000	0,510	0,000	0,410	0,000	0,570	0,000
1031268	COG1198L	gbs0303	priA	Cellular processess	DNA replication, recombination and repair	Primosomal protein N	0,469	0,003	0,513	0,003	0,557	0,017	0,557	0,016
1031270	COG0223J	gbs0304	fmt	Cellular processess	Translation, ribosomal structure and biogenesis	Methionyl-tRNA formyltransferase (EC 2.1.2.9)	1,128	0,164	0,791	0,152	1,342	0,002	0,766	0,056
1030722	COG0144J	gbs0305	sunL	Cellular processess	Translation, ribosomal structure and biogenesis	16S rRNA m(5)C 967 methyltransferase (EC 2.1.1.-)	0,499	0,000	0,496	0,000	0,466	0,000	0,422	0,000
1031295	COG0631T	gbs0306	stp1/pppl	Cellular processess	Signal transduction	Protein phosphatase 2C (EC 3.1.3.16)	0,475	0,000	0,657	0,000	0,450	0,000	0,660	0,000
1030725	COG0515RTK	gbs0307	stk1	Cellular processess	Signal transduction	Serine threonine protein kinase (EC 2.7.1.37)	0,557	0,000	0,641	0,000	0,523	0,000	0,790	0,001
1030188	COG4758S	gbs0308	yvqF	Transport and binding proteins	Unknown substrate	Transporter yvqF	0,625	0,005	1,001	0,997	0,785	0,130	1,148	0,166
1031297	COG4585T	gbs0309	yvqE	Cellular processess	Signal transduction	Two-component sensor protein yvqE (EC 2.7.3.-)	0,272	0,000	0,352	0,000	0,197	0,000	0,169	0,000
1031299	COG2197TK	gbs0310	yvqC	Cellular processess	Signal transduction	Two-component response regulator yvqC	0,918	0,352	1,088	0,652	1,020	0,818	1,374	0,000
1031439	COG0561R, (	gbs0311		Metabolism and transport	Central intermediary metabolism	Peptidyl-prolyl cis-trans isomerase (EC 5.2.1.8)	0,485	0,000	0,648	0,001	0,520	0,000	0,659	0,001
1030520	COG1098J	gbs0312		Cellular processess	Translation, ribosomal structure and biogenesis	S1-type RNA-binding domain	0,412	0,000	0,461	0,000	0,436	0,000	0,554	0,001
1030470	COG1180O	gbs0313		Metabolism and transport	Energy production and conversion	Pyruvate formate-lyase activating enzyme (EC 1.97.1.4)	5,393	0,000	4,714	0,000	5,371	0,001	4,771	0,000
1031443	COG1349KG	gbs0314		Cellular processess	Transcription	Transcriptional regulator, DeoR family	1,240	0,182	0,793	0,260	0,967	0,844	1,010	0,950
1031449	COG2390K	gbs0315		Cellular processess	Transcription	Transcriptional regulatory protein	0,612	0,062	0,826	0,369	0,505	0,026	0,976	0,908
1031313	-	gbs0316		Metabolism and transport	Carbohydrates, organic alcohols, and acids	PTS system, cellobiose-specific IIA component (EC 2.7.1.69)	4,126	0,000	2,761	0,029	3,855	0,000	3,974	0,000
1031316	COG1440G	gbs0317		Metabolism and transport	Carbohydrates, organic alcohols, and acids	PTS system, cellobiose-specific IIB component (EC 2.7.1.69)	3,824	0,000	2,998	0,000	3,289	0,005	3,543	0,000
1030527	COG1455G	gbs0318		Metabolism and transport	Carbohydrates, organic alcohols, and acids	PTS system, cellobiose-specific IIC component (EC 2.7.1.69)	2,776	0,001	4,151	0,002	2,376	0,001	4,703	0,000
1030546	COG1882C	gbs0319	pflD	Metabolism and transport	Energy production and conversion	Formate acetyltransferase (EC 2.3.1.54)	3,122	0,000	6,209	0,000	3,651	0,000	7,919	0,000
1031318	-	gbs0320	mipB	Metabolism and transport	Energy production and conversion	Transaldolase (EC 2.2.1.2)	1,200	0,682	5,478	0,000	1,463	0,359	8,495	0,000
1030547	COG0371C	gbs0321	gldA	Metabolism and transport	Energy production and conversion	Glycerol dehydrogenase (EC 1.1.1.6)	1,702	0,157	7,191	0,000	2,276	0,020	9,677	0,000
1031319	COG0031E	gbs0322	cysK	Metabolism and transport	Amino acids, peptides, aminosugars and amines	Cysteine synthase (EC 2.5.1.47)	0,529	0,000	0,442	0,000	0,399	0,000	0,350	0,000
1030550	COG1739S	gbs0323		Hypothetical		Hypothetical protein	0,188	0,000	0,324	0,000	0,245	0,000	0,318	0,000
1031416	COG4098L	gbs0324	comFA	Cellular processess	DNA transformation	COMF operon protein 1	3,388	0,002	17,643	0,049	2,959	0,030	17,869	0,002
1031320	COG1040R	gbs0325	comFC	General function predicted only		COMF operon protein 3	2,426	0,034	4,740	0,004	3,044	0,007	6,305	0,000
1030561	COG1544J	gbs0326		Cellular processess	Translation, ribosomal structure and biogenesis	Ribosome-associated factor Y	6,773	0,000	8,299	0,000	7,615	0,000	6,173	0,000
1031329	COG0527E	gbs0327		Metabolism and transport	Amino acids, peptides, aminosugars and amines	Aspartokinase (EC 2.7.2.4)	0,474	0,003	0,981	0,929	0,551	0,015	0,971	0,857
1031799	COG0637R	gbs0328		Metabolism and transport	Carbohydrates, organic alcohols, and acids	Beta-phosphoglucomutase (EC 5.4.2.6) Glucose-1-phosphate phosphodismutase (	0,246	0,000	0,271	0,000	0,200	0,000	0,309	0,000
1031330	COG1024I	gbs0329	phaB	Metabolism and transport	Fatty acid and phospholipids	Enoyl-CoA hydratase (EC 4.2.1.17)	0,912	0,609	0,403	0,005	0,641	0,056	0,226	0,002
1031333	COG1846K	gbs0330		Cellular processess	Transcription	Transcriptional regulator, MarR family	1,004	0,977	0,951	0,754	0,855	0,342	0,874	0,439
1030728	COG0332I	gbs0331	fabH	Metabolism and transport	Fatty acid and phospholipids	3-oxoacyl-[acyl-carrier-protein] synthase III (EC 2.3.1.41)	0,955	0,790	0,852	0,291	0,757	0,111	0,746	0,068
1031336	COG0236IQ	gbs0332	acpP	Metabolism and transport	Fatty acid and phospholipids	Acyl carrier protein	1,023	0,843	0,729	0,015	0,756	0,057	0,797	0,038
1031832	COG2070R	gbs0333	fabK	Metabolism and transport	Fatty acid and phospholipids	Enoyl-[acyl-carrier protein] reductase (NADH) (EC 1.3.1.9)	0,811	0,251	0,737	0,136	0,647	0,046	0,741	0,148
1031797	COG0331I	gbs0334	fabD	Metabolism and transport	Fatty acid and phospholipids	Malonyl-CoA-[acyl-carrier-protein] transacylase (EC 2.3.1.39)	0,722	0,211	0,688	0,162	0,604	0,092	0,674	0,157
1030587	COG1028IQR	gbs0335	fabG	Metabolism and transport	Fatty acid and phospholipids	3-oxoacyl-[acyl-carrier protein] reductase (EC 1.1.1.100)	0,609	0,061	1,039	0,854	0,634	0,070	1,093	0,631
1031339	COG0304IQ	gbs0336	fabF	Metabolism and transport	Fatty acid and phospholipids	3-oxoacyl-[acyl-carrier-protein] synthase (EC 2.3.1.41)	0,672	0,100	1,344	0,066	0,689	0,117	1,280	0,130
1030727	COG0511I	gbs0337	accB	Metabolism and transport	Fatty acid and phospholipids	Biotin carboxyl carrier protein of acetyl-CoA carboxylase (EC 6.4.1.2)	0,703	0,099	1,180	0,188	0,659	0,037	1,365	0,027
1031344	COG0764I	gbs0338		Metabolism and transport	Fatty acid and phospholipids	(3R)-hydroxyacyl-[acyl carrier protein] dehydratase (EC 4.2.1.60)	0,401	0,006	0,993	0,964	0,353	0,004	1,076	0,639
1030589	COG0439I	gbs0339	accC	Metabolism and transport	Fatty acid and phospholipids	Biotin carboxylase (EC 6.3.4.14)	0,497	0,012	1,108	0,492	0,479	0,008	1,117	0,439
1031347	COG0777I	gbs0340	accA	Metabolism and transport	Fatty acid and phospholipids	Acetyl-coenzyme A carboxylase carboxyl transferase subunit beta (EC 6.4.1.2)	0,655	0,038	1,127	0,318	0,619	0,019	1,176	0,189
1031345	COG0825I	gbs0341	accD	Metabolism and transport	Fatty acid and phospholipids	Acetyl-coenzyme A carboxylase carboxyl transferase subunit alpha (EC 6.4.1.2)	0,572	0,019	0,861	0,344	0,537	0,012	1,018	0,898
1030828	-	gbs0342		Hypothetical		Hypothetical protein	1,621	0,004	0,786	0,020	1,447	0,012	0,703	0,014
1031352	COG0172J	gbs0343	serS	Cellular processess	Translation, ribosomal structure and biogenesis	Seryl-tRNA synthetase (EC 6.1.1.11)	1,026	0,815	0,791	0,109	1,082	0,482	0,730	0,032
1030671	COG3274S	gbs0344		Hypothetical		Integral membrane protein	0,343	0,000	0,418	0,001	0,299	0,000	0,522	0,002
1030667	-	gbs0345	manO	Hypothetical		Hypothetical cytosolic protein	0,618	0,000	0,562	0,000	0,493	0,000	0,654	0,000
1030670	-	gbs0346	manN	Metabolism and transport	Carbohydrates, organic alcohols, and acids	PTS system, mannose-specific IID component (EC 2.7.1.69)	0,925	0,448	1,084	0,493	1,024	0,834	1,723	0,001
1030669	-	gbs0347	manM	Metabolism and transport	Carbohydrates, organic alcohols, and acids	PTS system, mannose-specific IIC component (EC 2.7.1.69)	0,879	0,226	1,041	0,700	0,935	0,582	1,389	0,005
1030489	COG2893G	gbs0348	manL	Metabolism and transport	Carbohydrates, organic alcohols, and acids	PTS system, mannose-specific IIAB component (EC 2.7.1.69)	1,309	0,034	0,839	0,117	1,247	0,057	1,455	0,002
1030672	COG0561R	gbs0349		Metabolism and transport	Central intermediary metabolism	Hydrolase (HAD superfamily)	0,183	0,000	0,447	0,000	0,273	0,000	0,513	0,000
1031417	-	gbs0350		Hypothetical		Hypothetical protein	0,557	0,000	0,739	0,003	0,489	0,000	0,775	0,018
1031366	-	gbs0351		General function predicted only		Permease	0,435	0,000	0,252	0,000	0,350	0,000	0,204	0,000
1030742	COG2252R	gbs0352		Metabolism and transport	Purines, pyrimidines, nucleosides, and nucleotides	Guanine-hypoxanthine permease	0,419	0,001	0,239	0,000	0,390	0,001	0,170	0,000

1030741	COG0802R	gbs0353		General function predicted only		ATP GTP hydrolase	0,246	0,000	0,283	0,000	0,249	0,000	0,359	0,000
1030754	-	gbs0354		Metabolism and transport	Central intermediary metabolism	Acetyltransferase, GNAT family	0,225	0,000	0,228	0,000	0,130	0,000	0,335	0,000
1031369	COG1316K	gbs0355	lytR	Cellular processess	Transcription	Transcriptional regulator, LytR family	0,311	0,000	0,248	0,000	0,282	0,000	0,353	0,000
1031370	-	gbs0356		Hypothetical		Hypothetical protein	1,027	0,804	0,632	0,004	0,903	0,433	0,691	0,007
1031380	COG0537FGI	gbs0357	hit	Metabolism and transport	Purines, pyrimidines, nucleosides, and nucleotides	Adenosine 5 -monophosphoramidase	1,700	0,001	0,953	0,698	1,531	0,002	1,055	0,644
1031374	-	gbs0358		Hypothetical		Hypothetical protein	1,795	0,006	0,945	0,446	1,634	0,007	0,954	0,659
1030766	COG1131V	gbs0359		Transport and binding proteins	Unknown substrate	ABC-type transporter ATP-binding protein ecsA	1,245	0,029	0,945	0,463	1,224	0,219	0,804	0,022
1031402	-	gbs0360		Transport and binding proteins	Unknown substrate	Protein ecsB	0,491	0,000	0,558	0,000	0,488	0,000	0,622	0,000
1031674	-	gbs0364		Hypothetical		Hypothetical protein	0,697	0,492	0,957	0,931	0,673	0,510	0,770	0,608
1031644	-	gbs0393		Hypothetical		LPXTG Hypothetical protein	1,682	0,000	0,358	0,000	1,745	0,000	0,311	0,000
1031640	-	gbs0402		Hypothetical		TRSE PROTEIN	2,508	0,000	2,832	0,007	2,571	0,002	1,953	0,067
1031639	-	gbs0403		Hypothetical		Hypothetical protein	1,356	0,160	1,797	0,030	1,395	0,220	1,752	0,018
1031636	COG1705NU	gbs0404		Hypothetical		Hypothetical protein	1,629	0,000	2,221	0,188	1,765	0,005	1,785	0,023
1031632	-	gbs0405		Hypothetical		Hypothetical protein	1,954	0,000	2,558	0,001	1,626	0,026	1,934	0,002
1031633	COG1192D	gbs0406		Cellular processess	Cell division	Hypothetical protein	1,012	0,926	1,043	0,779	0,946	0,641	1,069	0,688
1031631	-	gbs0407		Hypothetical		Hypothetical protein	0,626	0,004	0,576	0,002	0,663	0,007	0,550	0,001
1031629	-	gbs0408		Hypothetical		Hypothetical protein	0,600	0,002	0,543	0,001	0,524	0,001	0,558	0,001
1031627	-	gbs0409		Hypothetical		Hypothetical protein	0,829	0,042	0,701	0,005	0,863	0,069	0,813	0,061
1031628	-	gbs0410		Hypothetical		Hypothetical protein	0,709	0,410	1,653	0,159	0,932	0,858	1,348	0,426
1031625	COG0510M	gbs0411		General function predicted only		Phosphotransferase enzyme family	0,253	0,000	0,688	0,000	0,280	0,000	0,877	0,007
1029806	COG0220R	gbs0412		Cellular processess	Translation, ribosomal structure and biogenesis	tRNA (m(7)G46) methyltransferase (EC 2.1.1.33)	0,232	0,000	0,678	0,000	0,255	0,000	0,761	0,002
1031630	COG0779S	gbs0413		Hypothetical		Hypothetical cytosolic protein	0,628	0,001	0,530	0,000	0,560	0,000	0,554	0,001
1029803	COG0195K	gbs0414	nusA	Cellular processess	Transcription	N utilization substance protein A	0,594	0,000	0,618	0,000	0,559	0,000	0,634	0,001
1031624	COG2740K	gbs0415		Hypothetical		Hypothetical cytosolic protein	0,539	0,005	0,364	0,000	0,402	0,000	0,459	0,000
1030800	COG1358J	gbs0416		Cellular processess	Translation, ribosomal structure and biogenesis	LSU ribosomal protein L7AE	0,662	0,003	0,632	0,001	0,650	0,005	0,733	0,009
1031626	COG0532J	gbs0417	infB	Cellular processess	Translation, ribosomal structure and biogenesis	Bacterial Protein Translation Initiation Factor 2 (IF-2)	0,862	0,078	1,141	0,173	0,894	0,225	1,065	0,400
1029800	COG0858J	gbs0418	rbfA	Cellular processess	Translation, ribosomal structure and biogenesis	Ribosome-binding factor A	0,587	0,000	0,542	0,000	0,516	0,000	0,698	0,003
1031622	COG0657I	gbs0419		Metabolism and transport	Fatty acid and phospholipids	Esterase (EC 3.1.1.-)	0,306	0,000	0,333	0,000	0,338	0,000	0,340	0,000
1031825	COG3682K	gbs0420	copY	Cellular processess	Transcription	CopAB ATPases metal-fist type repressor	2,082	0,000	0,979	0,920	1,850	0,003	0,868	0,412
1029796	COG2217P	gbs0421	copA	Metabolism and transport	Inorganic ion transport and metabolism	Copper-exporting ATPase (EC 3.6.3.4)	1,520	0,022	1,766	0,001	1,469	0,050	1,788	0,002
1029801	COG2608P	gbs0422	copZ	Metabolism and transport	Inorganic ion transport and metabolism	Copper chaperone copZ	1,026	0,853	1,329	0,037	1,118	0,407	1,250	0,120
1030027	COG2860S	gbs0423		Hypothetical		Hypothetical membrane spanning protein	0,723	0,014	0,451	0,000	0,519	0,000	0,435	0,000
1031623	COG0561R	gbs0424		Metabolism and transport	Central intermediary metabolism	Hydrolase (HAD superfamily)	0,625	0,003	0,620	0,027	0,631	0,004	0,580	0,002
1030017	COG0258L, C	gbs0425	polA	Cellular processess	DNA replication, recombination and repair	DNA polymerase I (EC 2.7.7.7)	0,320	0,000	0,360	0,000	0,318	0,000	0,489	0,000
1031621	COG1832R	gbs0426		General function predicted only		CoA binding protein	0,277	0,000	0,392	0,000	0,270	0,000	0,443	0,000
1031826	COG0735P	gbs0427	perR	Metabolism and transport	Inorganic ion transport and metabolism	Oxidative stress response regulator BosR	0,382	0,000	0,548	0,000	0,360	0,000	0,624	0,000
1029793	-	gbs0428		Transport and binding proteins	Unknown substrate	LPXTG Transporter	11,445	0,000	6,411	0,000	12,099	0,000	4,449	0,001
1029791	COG0745TK	gbs0429		Cellular processess	Signal transduction	Two-component response regulator SaeR	1,673	0,000	1,079	0,588	1,531	0,002	1,012	0,948
1031620	COG0642T	gbs0430		Cellular processess	Signal transduction	Sensory transduction protein kinase SaeS (EC 2.7.3.-)	1,951	0,000	1,341	0,105	1,887	0,000	1,492	0,073
1029798	COG5523S	gbs0431		Hypothetical		Integral membrane protein	0,266	0,000	0,489	0,000	0,213	0,000	0,460	0,000
1031618	COG0343J	gbs0432	tgt	Cellular processess	Translation, ribosomal structure and biogenesis	Queuine tRNA-ribosyltransferase (EC 2.4.2.29)	0,770	0,094	0,249	0,000	0,588	0,010	0,514	0,007
1030136	COG4357S	gbs0433		Hypothetical		Zinc finger protein	0,746	0,409	0,456	0,002	0,872	0,508	0,259	0,000
1031619	COG1268R	gbs0434		Metabolism and transport	Cofactors, prosthetic groups, and carriers	BioY protein	2,317	0,012	1,774	0,143	2,088	0,114	0,819	0,588
1031614	COG1234R	gbs0435		Cellular processess	Toxin production and resistance	Metal-dependent hydrolase (EC 3.-.-.-)	1,739	0,000	0,705	0,019	1,550	0,005	0,683	0,014
1031613	COG0590FJ	gbs0436		Cellular processess	Translation, ribosomal structure and biogenesis	tRNA-specific adenosine deaminase (EC 3.5.4.-)	1,268	0,044	0,571	0,001	1,099	0,395	0,720	0,012
1031617	COG0166G	gbs0437	pgi	Metabolism and transport	Carbohydrates, organic alcohols, and acids	Glucose-6-phosphate isomerase (EC 5.3.1.9)	0,905	0,126	0,793	0,001	0,943	0,410	0,811	0,001
1031610	-	gbs0438		Metabolism and transport	Amino acids, peptides, aminosugars and amines	5-formyltetrahydrofolate cyclo-ligase (EC 6.3.3.2)	0,778	0,015	0,642	0,001	0,695	0,003	0,543	0,000
1031616	COG0705R	gbs0439		General function predicted only		Integral membrane protein (Rhomboid family)	0,749	0,016	0,654	0,003	0,692	0,004	0,663	0,003
1031615	COG1744R	gbs0440		Metabolism and transport	Purines, pyrimidines, nucleosides, and nucleotides	Nucleoside-binding protein	0,302	0,000	0,285	0,000	0,300	0,000	0,287	0,000
1031606	COG1210M	gbs0441	hasC	Cell Envelope	Biosynthesis and degradation of surface polysaccharides and lipopo	UTP--glucose-1-phosphate uridylyltransferase (EC 2.7.7.9)	0,436	0,000	0,484	0,000	0,408	0,000	0,522	0,000
1031612	COG0240C	gbs0442	gpsA	Metabolism and transport	Energy production and conversion	Glycerol-3-phosphate dehydrogenase [NAD(P)+] (EC 1.1.1.94)	0,603	0,000	0,771	0,023	0,668	0,000	0,882	0,037
1031609	COG0594J	gbs0443	rnpA	Cellular processess	Transcription	Ribonuclease P protein component (EC 3.1.26.5)	0,323	0,000	0,377	0,000	0,277	0,000	0,502	0,000
1031611	COG0706U	gbs0444		Cell Envelope	Other	60 kDa inner membrane protein YIDC	0,410	0,000	0,361	0,000	0,406	0,000	0,481	0,000
1031607	COG1847R	gbs0445		General function predicted only		Jag protein	0,520	0,000	0,583	0,000	0,493	0,000	0,735	0,003
1031602	COG3557J	gbs0446		Cellular processess	Translation, ribosomal structure and biogenesis	Hypothetical cytosolic protein	0,384	0,000	0,460	0,000	0,338	0,000	0,508	0,000
1031599	COG2137R	gbs0447	recX	Cellular processess	DNA replication, recombination, and repair	Regulatory protein recX	0,253	0,000	0,350	0,000	0,234	0,000	0,341	0,000
1031600	COG2265J	gbs0448		Cellular processess	Translation, ribosomal structure and biogenesis	tRNA (Uracil-5-) -methyltransferase (EC 2.1.1.35)	0,887	0,170	1,034	0,845	0,804	0,067	1,156	0,131
1029792	COG2820F	gbs0449		Metabolism and transport	Purines, pyrimidines, nucleosides, and nucleotides	Phosphorylase, Pnp Udp family	0,755	0,012	0,762	0,004	0,780	0,012	0,564	0,000
1031595	COG2153R	gbs0450		Metabolism and transport	Central intermediary metabolism	Acetyltransferase, GNAT family	0,783	0,048	0,493	0,000	0,673	0,001	0,548	0,000
1031603	COG1404O	gbs0451	cspA	Cellular processess	Posttranslational modification, protein turnover, chaperones	LPXTS C5A peptidase precursor (EC 3.4.21.-)	0,448	0,006	0,439	0,005	0,471	0,007	0,260	0,001
1031598	COG0463M	gbs0452		Cell Envelope	Biosynthesis and degradation of murein sacculus and peptidoglycar	alpha-L-Rha alpha-1,3-L-rhamnosyltransferase (EC 2.4.1.-)	0,376	0,000	0,359	0,000	0,360	0,000	0,490	0,001
1030121	COG0208F	gbs0453	nrdF.1	Metabolism and transport	Purines, pyrimidines, nucleosides, and nucleotides	Ribonucleoside-diphosphate reductase beta chain (EC 1.17.4.1)	0,692	0,085	1,249	0,281	0,635	0,027	1,548	0,045
1031597	COG1780F	gbs0454	nrdI	Metabolism and transport	Purines, pyrimidines, nucleosides, and nucleotides	NrdI protein	0,642	0,034	1,650	0,008	0,475	0,001	1,760	0,000
1031593	COG0209F	gbs0455	nrdE.1	Metabolism and transport	Purines, pyrimidines, nucleosides, and nucleotides	Ribonucleoside-diphosphate reductase alpha chain (EC 1.17.4.1)	0,664	0,094	2,011	0,020	0,488	0,018	2,230	0,000
1031596	-	gbs0456	lrrG	Hypothetical		LPXTS Cell surface protein	0,299	0,003	0,975	0,916	0,245	0,002	1,691	0,088
1031594	COG3576R	gbs0457		Hypothetical		Hypothetical cytosolic protein	0,754	0,178	2,071	0,003	0,697	0,081	2,348	0,001
1031588	COG3708S	gbs0458		Cellular processess	Transcription	Transcriptional regulator, AraC family	2,368	0,009	1,433	0,217	2,356	0,002	1,810	0,002
1031592	COG4367S	gbs0459		Hypothetical		Hypothetical protein	3,534	0,040	1,218	0,706	2,274	0,110	1,010	0,981
1031590	COG0599S	gbs0460		Metabolism and transport	Central intermediary metabolism	4-carboxymuconolactone decarboxylase (EC 4.1.1.44)	4,588	0,000	2,825	0,010	3,884	0,002	2,276	0,031
1031583	COG1917S	gbs0461		Hypothetical		Hypothetical cytosolic protein	3,438	0,001	3,352	0,002	2,779	0,001	1,931	0,056
1031591	COG0789K	gbs0462		Cellular processess	Transcription	Transcriptional regulator, MerR family	2,324	0,065	1,780	0,401	2,028	0,154	1,447	0,336
1031582	COG1063ER	gbs0464		Metabolism and transport	Carbohydrates, organic alcohols, and acids	Sorbitol dehydrogenase (EC 1.1.1.14)	4,255	0,001	4,420	0,031	4,898	0,000	3,424	0,021
1031584	COG0656R	gbs0466		Metabolism and transport	Central intermediary metabolism	Aldose reductase (EC 1.1.1.21)	5,229	0,000	2,927	0,008	5,065	0,000	1,791	0,045
1031586	COG1230P	gbs0467	czcD	Metabolism and transport	Inorganic ion transport and metabolism	Cobalt-zinc-cadmium resistance protein czcD	3,403	0,000	1,532	0,008	2,787	0,001	0,902	0,375
1031579	COG1309K	gbs0468		Cellular processess	Transcription	Transcriptional regulator, TetR family	0,325	0,000	0,509	0,068	0,104	0,000	0,437	0,000
1031581	COG4753T	gbs0469		Cellular processess	Transcription	Transcriptional regulator, AraC family	0,567	0,003	0,569	0,002	0,454	0,000	0,728	0,035
1031585	-	gbs0470	alp2	Hypothetical		LPXTG Cell surface protein	1,290	0,043	0,873	0,407	1,144	0,274	0,728	0,046
1031575	COG3077L	gbs0471		Cellular processess	DNA replication, recombination and repair	DNA-damage-inducible protein J	0,656	0,005	0,743	0,032	0,643	0,003	0,730	0,032
1031578	-	gbs0472		Hypothetical		Hypothetical cytosolic protein	0,588	0,001	0,670	0,006	0,516	0,000	0,671	0,006
1031577	-	gbs0473		Hypothetical		Hypothetical exported protein	0,941	0,501	1,013	0,882	0,888	0,194	0,876	0,171
1031567	-	gbs0474		Hypothetical		Hypothetical cytosolic protein	0,204	0,000	0,264	0,000	0,163	0,000	0,243	0,000
1031573	COG1396K	gbs0475		Cellular processess	Transcription	Transcriptional regulator, Cro CI family	1,298	0,397	2,107	0,040	1,001	0,998	2,705	0,046
1031572	-	gbs0476		Hypothetical		Membrane protein	2,328	0,058	3,548	0,055	5,032	0,000	5,338	0,036
1031571	-	gbs0477		Hypothetical		Hypothetical exported protein	2,414	0,071	2,603	0,190	1,683	0,278	2,647	0,041
1031580	-	gbs0478		Hypothetical		Hypothetical exported protein	6,656	0,001	22,354	0,099	2,910	0,032	4,489	0,003
1031568	-	gbs0479		Hypothetical		LPXTG Cell surface protein	2,606	0,034	6,187	0,008	3,317	0,064	6,328	0,001
1031566	-	gbs0480		Hypothetical		Hypothetical membrane spanning protein	1,350	0,174	0,781	0,186	1,409	0,092	0,695	0,004
1031576	-	gbs0481		Hypothetical		Hypothetical protein	19,023	0,022	3,887	0,033	11,449	0,025	5,582	0,049

1031561	-	gbs0482		Mobile and extrachromosomal elements		DNA integration recombination inversion protein	2,152	0,001	0,710	0,225	1,536	0,078	0,460	0,012
1031558	COG1917S	gbs0484		General function predicted only		Putative acetate kinase	1,187	0,131	0,924	0,555	1,210	0,202	0,760	0,005
1031569	COG1917S	gbs0485		Hypothetical		Hypothetical cytosolic protein	1,988	0,000	1,484	0,020	2,061	0,001	1,192	0,037
1031565	COG0500QR	gbs0486		Metabolism and transport	Secondary metabolites	SAM-dependent methyltransferase	2,604	0,000	1,611	0,000	2,519	0,000	1,369	0,003
1031557	-	gbs0487		Hypothetical		Hypothetical protein	1,146	0,293	0,398	0,000	1,111	0,531	0,456	0,000
1031560	-	gbs0488		Hypothetical		Hypothetical cytosolic protein	0,385	0,000	0,183	0,000	0,325	0,000	0,314	0,000
1031551	COG1670J	gbs0489		Cellular processess	Translation, ribosomal structure and biogenesis	Ribosomal-protein-serine acetyltransferase (EC 2.3.1.-)	0,504	0,000	0,410	0,001	0,426	0,000	0,438	0,000
1031559	COG1670J	gbs0490		Cellular processess	Translation, ribosomal structure and biogenesis	Ribosomal-protein-serine acetyltransferase (EC 2.3.1.-)	0,567	0,000	0,646	0,007	0,642	0,001	0,721	0,008
1031554	COG0703E	gbs0491		Hypothetical		Hypothetical protein	0,393	0,000	0,331	0,000	0,354	0,000	0,483	0,000
1031555	COG0525J	gbs0492	valS	Cellular processess	Translation, ribosomal structure and biogenesis	Valyl-tRNA synthetase (EC 6.1.1.9)	0,777	0,004	0,879	0,136	0,769	0,001	0,843	0,034
1031552	-	gbs0493		Hypothetical		Hypothetical protein	5,206	0,000	1,154	0,147	3,850	0,000	1,066	0,298
1031550	COG0673R	gbs0494		Metabolism and transport	Central intermediary metabolism	NAD-dependent oxidoreductase	0,811	0,027	0,632	0,054	0,904	0,329	0,508	0,006
1031556	COG0598P	gbs0495		Metabolism and transport	Inorganic ion transport and metabolism	Magnesium and cobalt transport protein corA	0,604	0,004	0,973	0,896	0,709	0,011	0,624	0,043
1031548	COG2315S	gbs0496		Hypothetical		Hypothetical cytosolic protein	0,202	0,000	0,340	0,002	0,430	0,002	0,408	0,002
1031547	COG2502E	gbs0497	asnA	Metabolism and transport	Amino acids, peptides, aminosugars and amines	Aspartate--ammonia ligase (EC 6.3.1.1)	0,489	0,001	0,212	0,000	0,374	0,000	0,109	0,000
1031540	-	gbs0498		Metabolism and transport	Energy production and conversion	Thioredoxin	1,521	0,002	0,942	0,833	1,341	0,015	0,573	0,012
1031553	COG0742L	gbs0499		Cellular processess	DNA replication, recombination and repair	Methyltransferase (EC 2.1.1.-)	0,710	0,032	0,394	0,000	0,612	0,002	0,596	0,000
1031539	-	gbs0500		Hypothetical		Hypothetical protein	0,629	0,001	0,598	0,004	0,497	0,000	0,500	0,000
1031546	COG0669H	gbs0501		Metabolism and transport	Cofactors, prosthetic groups, and carriers	Phosphopantetheine adenylyltransferase (EC 2.7.7.3)	0,598	0,001	0,461	0,000	0,469	0,000	0,569	0,001
1031535	COG3480T	gbs0502		Cellular processess	Signal transduction	ATP-dependent endopeptidase Lon (EC 3.4.21.53)	0,434	0,000	0,413	0,000	0,386	0,000	0,426	0,000
1031532	COG0737F	gbs0503		Metabolism and transport	Purines, pyrimidines, nucleosides, and nucleotides	5 -nucleotidase (EC 3.1.3.5)	0,705	0,187	1,231	0,483	0,661	0,108	1,131	0,623
1031529	-	gbs0504		Hypothetical		Hypothetical cytosolic protein	0,612	0,001	0,403	0,000	0,471	0,000	0,584	0,000
1031542	COG0820R	gbs0505		General function predicted only		Radical SAM family enzyme	0,457	0,001	0,598	0,004	0,462	0,001	0,641	0,007
1031537	COG4767V	gbs0506		Hypothetical		VanZ family protein	0,301	0,000	0,442	0,001	0,346	0,000	0,356	0,000
1031534	COG1132V	gbs0507		Cellular processess	Toxin production and resistance	Multidrug resistance ABC transporter ATP-binding and permease protein	0,849	0,266	1,490	0,148	0,760	0,209	1,193	0,332
1031526	COG1132V	gbs0508		Cellular processess	Toxin production and resistance	Multidrug protein lipid ABC transporter family, ATP-binding and permease protein	0,527	0,006	1,139	0,593	0,397	0,004	1,105	0,651
1031538	COG0512EH	gbs0509	trpG	Metabolism and transport	Cofactors, prosthetic groups, and carriers	Anthranilate synthase component II (EC 4.1.3.27) Para-aminobenzoate synthase g	0,725	0,004	0,731	0,003	0,832	0,069	0,735	0,002
1031533	COG1268R	gbs0510		Metabolism and transport	Cofactors, prosthetic groups, and carriers	BioY protein	0,977	0,933	1,584	0,151	0,758	0,247	0,721	0,163
1031523	COG0502H	gbs0511		Metabolism and transport	Cofactors, prosthetic groups, and carriers	Biotin synthase (EC 2.8.1.6)	1,734	0,126	3,248	0,021	1,445	0,377	1,840	0,037
1031531	-	gbs0512		Hypothetical		Hypothetical protein	1,802	0,063	6,021	0,073	2,263	0,131	4,770	0,007
1031527	COG0318IQ	gbs0514		Metabolism and transport	Fatty acid and phospholipids	Long-chain-fatty-acid--CoA ligase (EC 6.2.1.3)	2,629	0,022	5,056	0,038	2,092	0,108	3,370	0,002
1031525	COG0177L	gbs0515	nth	Cellular processess	DNA replication, recombination and repair	Endonuclease III (EC 4.2.99.18)	1,187	0,175	0,957	0,616	1,367	0,005	0,808	0,167
1031520	-	gbs0516		General function predicted only		Type 4 prepilin-like proteins leader peptide processing enzyme	4,713	0,059	13,657	0,098	3,692	0,231	5,324	0,008
1031528	-	gbs0517		Hypothetical		Hypothetical cytosolic protein	0,453	0,000	0,576	0,000	0,325	0,000	0,623	0,000
1031524	COG1940KG	gbs0518	glcK	Metabolism and transport	Carbohydrates, organic alcohols, and acids	Glucokinase (EC 2.7.1.2)	0,795	0,005	0,950	0,389	0,747	0,002	1,135	0,031
1031517	COG0607P	gbs0519		Metabolism and transport	Inorganic ion transport and metabolism	Rhodanese-related sulfurtransferases	0,631	0,000	0,784	0,002	0,664	0,001	0,936	0,411
1031521	COG1217T	gbs0520	typA	Cellular processess	Signal transduction	GTP-binding protein TypA BipA	0,246	0,000	0,357	0,000	0,234	0,000	0,377	0,000
1031519	-	gbs0521		Hypothetical		Hypothetical membrane spanning protein	0,268	0,000	0,319	0,000	0,296	0,000	0,297	0,000
1031514	COG0771M	gbs0522	murD	Cell Envelope	Biosynthesis and degradation of murein sacculus and peptidoglycar	UDP-N-acetylmuramoylalanine--D-glutamate ligase (EC 6.3.2.9)	0,617	0,001	0,389	0,001	0,573	0,001	0,578	0,000
1031516	COG0707M	gbs0523	murG	Cell Envelope	Biosynthesis and degradation of murein sacculus and peptidoglycar	UDP-N-acetylglucosamine--N-acetylmuramyl-(pentapeptide) pyrophosphoryl-undeca	0,622	0,001	0,915	0,464	0,704	0,001	1,026	0,827
1031512	COG1589M	gbs0524	divIB	Cellular processess	Cell division	Cell-division initiation protein DivIB	0,447	0,000	0,679	0,002	0,470	0,000	0,780	0,011
1031518	COG0849D	gbs0525	ftsA	Cellular processess	Cell division	Cell division protein ftsA	0,346	0,000	0,639	0,000	0,330	0,000	0,828	0,008
1031509	COG0206D	gbs0526	ftsZ	Cellular processess	Cell division	Cell division protein ftsZ	0,524	0,000	0,948	0,519	0,553	0,000	1,262	0,001
1031508	COG0325R	gbs0527		General function predicted only		Pyridoxal-5 -phosphate family protein	0,207	0,000	0,631	0,000	0,190	0,000	0,942	0,397
1031513	COG1799S	gbs0528		Hypothetical		Hypothetical cytosolic protein	0,262	0,000	0,580	0,000	0,265	0,000	0,880	0,099
1031506	-	gbs0529		Hypothetical		Integral membrane protein	0,270	0,000	0,750	0,009	0,278	0,000	1,020	0,666
1031510	COG2302S	gbs0530		General function predicted only		RNA binding protein	0,209	0,000	0,690	0,001	0,211	0,000	0,973	0,466
1031511	COG3599D	gbs0531	divIVAS	Cellular processess	Cell division	Cell division initiation protein DivIVA	0,291	0,000	0,666	0,000	0,309	0,000	0,860	0,025
1031507	COG0060J	gbs0532	ileS	Cellular processess	Translation, ribosomal structure and biogenesis	Isoleucyl-tRNA synthetase (EC 6.1.1.5)	0,514	0,000	0,585	0,001	0,540	0,000	0,586	0,001
1031505	-	gbs0533		Hypothetical		Hypothetical cytosolic protein	6,604	0,000	2,412	0,002	6,329	0,000	1,832	0,007
1031515	COG0494LR	gbs0534	mutT	General function predicted only		Mutator mutT protein (7,8-dihydro-8-oxoguanine-triphosphatase) (EC 3.6.1.-)	2,058	0,000	0,753	0,064	2,117	0,001	1,272	0,391
1031503	COG0542O	gbs0535	clpE	Cellular processess	Posttranslational modification, protein turnover, chaperones	ATP-dependent endopeptidase clp ATP-binding subunit clpE	1,547	0,059	1,474	0,070	1,771	0,027	2,458	0,001
1031504	-	gbs0536		Hypothetical		Hypothetical cytosolic protein	0,312	0,000	0,397	0,000	0,300	0,000	0,360	0,000
1031502	COG0765E	gbs0537	artQ	Metabolism and transport	Amino acids, peptides, aminosugars and amines	Arginine transport system permease protein artQ	0,341	0,025	0,293	0,019	0,382	0,033	0,238	0,014
1031497	COG1126E	gbs0538	artP	Metabolism and transport	Amino acids, peptides, aminosugars and amines	Arginine transport ATP-binding protein artP	0,416	0,033	0,257	0,012	0,406	0,031	0,270	0,013
1031498	COG1109G	gbs0539		Metabolism and transport	Carbohydrates, organic alcohols, and acids	Phosphoglucomutase (EC 5.4.2.2) Phosphomannomutase (EC 5.4.2.8)	0,838	0,214	1,304	0,033	0,854	0,132	1,417	0,008
1031499	COG0190H	gbs0540	folD	Metabolism and transport	Cofactors, prosthetic groups, and carriers	Methylenetetrahydrofolate dehydrogenase (NADP+) (EC 1.5.1.5) Methenyltetrahyd	1,094	0,496	0,988	0,939	1,242	0,136	1,113	0,282
1031492	COG0063G	gbs0541		Metabolism and transport	Carbohydrates, organic alcohols, and acids	Sugar kinase	0,574	0,009	0,452	0,003	0,621	0,021	0,333	0,000
1031490	COG1570L	gbs0542		Cellular processess	DNA replication, recombination and repair	Exodeoxyribonuclease VII large subunit (EC 3.1.11.6)	0,750	0,014	1,258	0,096	0,902	0,302	1,356	0,006
1031496	COG1722L	gbs0543	xseB	Cellular processess	DNA replication, recombination and repair	Exodeoxyribonuclease VII small subunit (EC 3.1.11.6)	0,608	0,004	1,333	0,030	0,767	0,045	1,399	0,012
1031500	COG0142H	gbs0544	fps	Metabolism and transport	Cofactors, prosthetic groups, and carriers	Dimethylallyltransferase (EC 2.5.1.1) Geranyltranstransferase (EC 2.5.1.10)	0,325	0,000	0,635	0,002	0,326	0,000	0,886	0,128
1031493	COG1189J	gbs0545	hemA	Cellular processess	Translation, ribosomal structure and biogenesis	Hemolysin	0,345	0,000	0,899	0,377	0,334	0,000	0,929	0,364
1031491	COG1438K	gbs0546	argR1	Cellular processess	Transcription	Arginine repressor, argR	0,243	0,000	0,633	0,001	0,269	0,000	0,891	0,109
1031484	COG0497L	gbs0547	recN	Cellular processess	DNA replication, recombination and repair	DNA repair protein recN	0,402	0,000	1,044	0,371	0,389	0,000	1,149	0,070
1031489	COG1307S	gbs0548		Metabolism and transport	Fatty acid and phospholipids	Fatty acid-binding protein, DegV family	0,343	0,000	0,655	0,000	0,337	0,000	0,706	0,000
1031488	COG2755E	gbs0549		Metabolism and transport	Fatty acid and phospholipids	Lipase Acylhydrolase with GDSL-like motif	0,210	0,000	0,586	0,000	0,183	0,000	0,691	0,001
1031479	-	gbs0550		Hypothetical		Hypothetical membrane associated protein	0,186	0,000	0,433	0,000	0,172	0,000	0,516	0,000
1031763	-	gbs0551		Cellular processess	DNA replication, recombination and repair	DNA-binding protein HU	2,598	0,000	1,404	0,043	2,602	0,000	1,169	0,143
1031782	-	gbs0552		Hypothetical		Hypothetical protein	3,795	0,000	0,658	0,001	3,247	0,000	0,423	0,000
1031236	COG0167F	gbs0553	pyrD	Metabolism and transport	Purines, pyrimidines, nucleosides, and nucleotides	Dihydroorotate dehydrogenase (EC 1.3.3.1)	0,908	0,584	0,286	0,002	0,917	0,624	0,219	0,001
1031697	COG2348V	gbs0554		Cellular processess	Toxin production and resistance	Factor essential for expression of methicillin resistance	0,425	0,000	0,437	0,000	0,382	0,000	0,631	0,000
1031713	COG2348V	gbs0555	murM	Cell Envelope	Biosynthesis and degradation of murein sacculus and peptidoglycar	UDP-N-acetylmuramoylpentapeptide-lysine N(6)-alanyltransferase (EC 2.3.2.10) U	0,324	0,000	0,446	0,000	0,340	0,000	0,556	0,000
1030787	COG2348V	gbs0556		Cell Envelope	Biosynthesis and degradation of murein sacculus and peptidoglycar	UDP-N-acetylmuramoylpentapeptide-lysine N(6)-alanyltransferase (EC 2.3.2.10) U	0,553	0,000	0,673	0,004	0,533	0,000	0,854	0,105
1031795	COG0561R	gbs0557		Metabolism and transport	Central intermediary metabolism	Hydrolase (HAD superfamily)	0,368	0,000	0,356	0,000	0,303	0,000	0,428	0,000
1030920	COG1078R	gbs0558		General function predicted only		dGTP triphosphohydrolase	0,350	0,000	0,404	0,000	0,329	0,000	0,573	0,002
1031474	-	gbs0559		Hypothetical		Hypothetical cytosolic protein	0,553	0,003	0,421	0,000	0,517	0,000	0,436	0,000
1030837	COG0474P	gbs0560	pacL	Metabolism and transport	Inorganic ion transport and metabolism	Calcium-transporting ATPase (EC 3.6.3.8)	0,870	0,177	0,991	0,925	0,789	0,061	1,107	0,281
1031766	COG1409R	gbs0561		Metabolism and transport	Central intermediary metabolism	Phosphoesterase	0,448	0,000	0,316	0,000	0,235	0,000	0,176	0,000
1031434	-	gbs0562		Metabolism and transport	Carbohydrates, organic alcohols, and acids	Fructose-1,6-bisphosphatase (EC 3.1.3.11)	0,873	0,611	2,877	0,001	0,939	0,804	2,911	0,002
1031105	COG1600C	gbs0563		Metabolism and transport	Energy production and conversion	iron-sulfur cluster-binding protein	0,442	0,000	0,646	0,031	0,351	0,000	1,282	0,087
1031794	COG1186J	gbs0564	prfB	Cellular processess	Translation, ribosomal structure and biogenesis	Bacterial Peptide Chain Release Factor 2 (RF-2)	0,387	0,000	0,505	0,000	0,381	0,000	0,616	0,001
1030257	COG2884D	gbs0565	ftsE	Cellular processess	Cell division	Cell division ATP-binding protein ftsE	0,511	0,000	0,623	0,000	0,512	0,000	0,675	0,000
1031425	COG2177D	gbs0566	ftsX	Cellular processess	Cell division	Cell division protein ftsX	0,604	0,000	0,571	0,000	0,564	0,000	0,742	0,000
1031796	COG0596R	gbs0567		General function predicted only		CARBOXYMETHYLENEBUTENOLIDASE-RELATED PROTEIN	0,316	0,001	0,345	0,001	0,277	0,001	0,485	0,005
1031200	COG0491R	gbs0568		General function predicted only		Hydroxyacylglutathione hydrolase (EC 3.1.2.6)	0,470	0,000	0,578	0,000	0,483	0,000	0,636	0,001
1031652	COG1028IQR	gbs0569		Metabolism and transport	Fatty acid and phospholipids	(R,R)-butanediol dehydrogenase (EC 1.1.1.4) Acetoin dehydrogenase (EC 1.1.1.5)	4,183	0,000	4,854	0,001	4,539	0,000	3,919	0,001
1031729	COG0847L, C	gbs0570	dinG	Cellular processess	DNA replication, recombination and repair	ATP-dependent helicase, DinG family	0,400	0,000	0,571	0,004	0,253	0,000	0,861	0,183
1031689	COG0436E	gbs0571	aspC	Metabolism and transport	Amino acids, peptides, aminosugars and amines	Aspartate aminotransferase (EC 2.6.1.1)	0,322	0,000	0,473	0,000	0,278	0,000	0,558	0,000

1031758	COG0017J	gbs0572	asnS	Cellular processess	Translation, ribosomal structure and biogenesis	Asparaginyl-tRNA synthetase (EC 6.1.1.22)	0,290	0,000	0,393	0,000	0,273	0,000	0,464	0,000
1031767	-	gbs0573		Hypothetical		Integral membrane protein	0,592	0,010	0,402	0,001	0,462	0,002	0,425	0,001
1031428	COG1957F	gbs0574		Metabolism and transport	Purines, pyrimidines, nucleosides, and nucleotides	Inosine-uridine preferring nucleoside hydrolase (EC 3.2.2.1)	0,863	0,158	0,611	0,001	0,710	0,006	0,638	0,002
1031406	COG1764O	gbs0575		Cellular processess	Posttranslational modification, protein turnover, chaperones	Osmotically inducible protein C	0,652	0,037	0,245	0,001	0,486	0,007	0,448	0,005
1031160	COG1660R	gbs0576		General function predicted only		ATP-binding protein (contains P-loop)	0,973	0,663	1,014	0,827	1,248	0,032	1,161	0,028
1031710	-	gbs0577		Hypothetical		Hypothetical membrane associated protein	1,084	0,136	1,297	0,056	1,116	0,196	1,610	0,000
1031411	COG1481S	gbs0578		Hypothetical		Hypothetical cytosolic protein	0,387	0,000	0,560	0,000	0,403	0,000	0,649	0,000
1031472	-	gbs0579	pepD	Cellular processess	Posttranslational modification, protein turnover, chaperones	Dipeptidase A (EC 3.4.13.-)	0,597	0,000	0,604	0,006	0,635	0,000	0,811	0,023
1031163	COG0803P, C	gbs0580		Metabolism and transport	Inorganic ion transport and metabolism	High-affinity zinc uptake system protein znuA precursor Hypothetical exported prote	0,675	0,005	1,088	0,447	0,852	0,239	1,044	0,674
1031350	COG0254J	gbs0581	rpmE	Cellular processess	Translation, ribosomal structure and biogenesis	LSU ribosomal protein L31P	1,212	0,003	0,754	0,008	1,065	0,434	0,771	0,008
1031269	COG0618R	gbs0582		Metabolism and transport	Central intermediary metabolism	Phosphoesterase, DHH family protein	0,925	0,376	0,735	0,008	0,908	0,224	0,583	0,000
1031736	COG1816F	gbs0583	add	Metabolism and transport	Purines, pyrimidines, nucleosides, and nucleotides	Adenosine deaminase (EC 3.5.4.4)	0,865	0,424	0,702	0,118	0,837	0,286	0,722	0,117
1030782	COG0716C	gbs0584		Metabolism and transport	Energy production and conversion	Flavodoxin	0,468	0,000	0,661	0,000	0,463	0,000	0,751	0,001
1030889	-	gbs0585		Metabolism and transport	Amino acids, peptides, aminosugars and amines	Chorismate mutase (EC 5.4.99.5)	0,213	0,000	0,364	0,000	0,215	0,000	0,493	0,000
1031186	COG0038P	gbs0586		Metabolism and transport	Inorganic ion transport and metabolism	Chloride channel protein	0,390	0,000	0,584	0,009	0,407	0,000	0,702	0,003
1031049	-	gbs0587	rplS	Cellular processess	Translation, ribosomal structure and biogenesis	LSU ribosomal protein L19P	0,767	0,018	0,732	0,053	0,788	0,040	0,570	0,000
1030772	COG2963L	gbs0589		Mobile and extrachromosomal elements		Transposase	0,792	0,106	1,476	0,041	0,888	0,380	1,043	0,804
1031302	-	gbs0593		Hypothetical		Hypothetical protein	1,313	0,556	5,128	0,026	1,375	0,469	2,747	0,077
1031026	-	gbs0594		Transport and binding proteins	Unknown substrate	ABC transporter permease protein	3,999	0,001	0,848	0,502	3,098	0,001	0,709	0,156
1031257	COG1136V	gbs0595		Transport and binding proteins	Unknown substrate	ABC transporter ATP-binding protein	3,896	0,000	1,567	0,001	3,850	0,000	1,202	0,188
1031334	COG0577V	gbs0596		Transport and binding proteins	unknown substrate	ABC transporter permease protein	3,174	0,000	1,281	0,176	3,209	0,000	1,045	0,760
1031326	COG0745TK	gbs0597	vcnS	hypothetical		Hypothetical protein	0,707	0,112	0,959	0,905	0,737	0,058	0,541	0,005
1031040	COG0642T	gbs0598	vcnR	Cellular processess	Signal transduction	Phosphate regulon sensor protein phoR (EC 2.7.3.-)	0,453	0,001	0,324	0,000	0,286	0,000	0,500	0,001
1031031	-	gbs0600		Hypothetical		Hypothetical protein	3,928	0,000	1,464	0,002	4,389	0,001	1,093	0,249
1031223	-	gbs0601		Cell Envelope	Biosynthesis and degradation of murein sacculus and peptidoglycar	Rod shape-determining protein rodA	0,271	0,000	0,379	0,000	0,298	0,000	0,355	0,000
1031348	COG0546R	gbs0602		Metabolism and transport	Central intermediary metabolism	Phosphatase	0,257	0,000	0,586	0,000	0,223	0,000	0,768	0,000
1031332	COG0187L	gbs0603	gyrB	Cellular processess	DNA replication, recombination and repair	DNA gyrase subunit B (EC 5.99.1.3)	0,422	0,000	0,726	0,000	0,440	0,000	0,861	0,030
1031401	COG4477D	gbs0604		Cellular processess	Cell division	Septation ring formation regulator	0,307	0,000	0,588	0,000	0,325	0,000	0,710	0,000
1031358	COG0560E	gbs0605		Metabolism and transport	Amino acids, peptides, aminosugars and amines	Phosphoserine phosphatase (EC 3.1.3.3)	1,297	0,035	0,693	0,054	1,175	0,168	0,582	0,001
1031351	COG0494LR	gbs0606		Cellular processess	DNA replication, recombination and repair	Phosphohydrolase (MutT nudix family protein)	0,957	0,641	0,546	0,002	0,867	0,178	0,891	0,385
1031360	COG5506S	gbs0607		Hypothetical		Hypothetical cytosolic protein	0,918	0,473	0,591	0,020	0,786	0,092	0,800	0,150
1031354	COG0148G	gbs0608	eno	Metabolism and transport	Carbohydrates, organic alcohols, and acids	Enolase (EC 4.2.1.11)	1,904	0,000	1,990	0,000	2,017	0,000	2,127	0,000
1031375	-	gbs0609		General function predicted only		Streptodornase (EC 3.1.21.1)	1,684	0,121	7,388	0,000	2,866	0,000	9,021	0,000
1031372	COG0128E	gbs0610	aroA	Metabolism and transport	Amino acids, peptides, aminosugars and amines	3-phosphoshikimate 1-carboxyvinyltransferase (EC 2.5.1.19)	0,956	0,660	0,602	0,001	0,826	0,070	0,838	0,140
1031039	COG0703E	gbs0611	aroK	Metabolism and transport	Amino acids, peptides, aminosugars and amines	Shikimate kinase (EC 2.7.1.71)	0,450	0,000	0,565	0,001	0,457	0,000	0,598	0,001
1030877	COG1316K	gbs0612		Cellular processess	Transcription	Transcriptional regulator, LytR family	0,540	0,000	0,749	0,003	0,582	0,000	0,738	0,015
1030885	COG2265J	gbs0613		Cellular processess	Translation, ribosomal structure and biogenesis	23S rRNA m(5)U 1939 methyltransferase (EC 2.1.1.-)	0,184	0,000	0,329	0,000	0,379	0,000	0,292	0,000
1030908	-	gbs0614		Hypothetical		Hypothetical protein	0,266	0,000	0,517	0,000	0,278	0,000	0,448	0,000
1031260	COG3700R	gbs0615		General function predicted only		Class B acid phosphatase (EC 3.1.3.2)	4,401	0,000	2,677	0,000	4,182	0,000	2,939	0,000
1030879	COG4283S	gbs0616		Hypothetical		Hypothetical cytosolic protein	2,691	0,002	2,570	0,000	2,344	0,004	3,190	0,000
1031170	-	gbs0617		Hypothetical		Hypothetical protein	0,304	0,002	0,499	0,020	0,235	0,000	0,719	0,433
1031249	COG1309K	gbs0618		Cellular processess	Transcription	Transcriptional regulator, TetR family	0,661	0,053	1,768	0,045	0,612	0,134	1,428	0,270
1030880	-	gbs0619		Hypothetical		Hypothetical protein	1,087	0,725	2,968	0,069	1,212	0,499	3,133	0,011
1031243	COG2963L	gbs0621		Mobile and extrachromosomal elements		Transposase	0,533	0,000	2,272	0,000	0,541	0,000	3,420	0,000
1031262	-	gbs0622		Hypothetical		Hypothetical protein	1,075	0,785	1,364	0,288	0,845	0,376	2,035	0,037
1031121	-	gbs0625		Cellular processess	Posttranslational modification, protein turnover, chaperones	33 kDa chaperonin	0,705	0,001	2,752	0,000	0,925	0,402	3,842	0,000
1030901	-	gbs0627	araC	Cellular processess	Transcription	Transcriptional regulator, AraC family	1,392	0,039	2,589	0,000	1,401	0,092	3,624	0,000
1031310	COG4932M	gbs0628		Hypothetical		IPXTG Hypothetical protein	3,648	0,001	5,881	0,000	4,473	0,000	6,127	0,000
1031068	COG4932M	gbs0629		Cellular processess	Posttranslational modification, protein turnover, chaperones	IPXTG Hypothetical protein	1,893	0,275	2,792	0,011	2,088	0,058	2,405	0,102
1031473	COG3764M	gbs0630		Cellular processess	Posttranslational modification, protein turnover, chaperones	Sortase	1,739	0,227	6,812	0,053	1,894	0,096	3,644	0,036
1031341	COG3764M	gbs0631		Cellular processess	Posttranslational modification, protein turnover, chaperones	Sortase	2,420	0,068	5,596	0,003	2,501	0,001	4,476	0,000
1031148	COG4932M	gbs0632		Cell Envelope	Other	FPKTG Collagen adhesion protein	4,504	0,009	8,676	0,008	3,466	0,150	7,647	0,005
1030835	COG4932M	gbs0636		Metabolism and transport	Energy production and conversion	Collagen adhesion protein	6,815	0,017	8,519	0,023	11,457	0,021	8,853	0,003
1031305	-	gbs0638		Hypothetical		Hypothetical protein	0,468	0,000	0,564	0,003	0,340	0,000	0,619	0,023
1031469	-	gbs0639		Hypothetical		Hypothetical protein	3,330	0,003	14,586	0,083	4,678	0,010	3,809	0,000
1030886	COG1680V	gbs0640		Cellular processess	Toxin production and resistance	Beta-lactamase family protein	0,353	0,000	0,431	0,000	0,288	0,000	0,536	0,000
1030753	COG4586R	gbs0641	drrA	General function predicted only		Daunorubicin resistance ATP-binding protein drrA	0,338	0,035	1,556	0,070	0,223	0,019	1,526	0,103
1031475	COG4587R	gbs0642		General function predicted only		Daunorubicin resistance transmembrane protein	0,184	0,005	1,877	0,020	0,270	0,008	2,018	0,002
1031396	COG3694R	gbs0643		Transport and binding proteins	Unknown substrate	ABC transporter permease protein	0,312	0,003	1,801	0,013	0,234	0,001	1,461	0,249
1031464	-	gbs0644	cylX	Hypothetical		Hypothetical protein	1,967	0,000	0,794	0,137	1,494	0,010	0,887	0,349
1031688	COG0331I	gbs0645	cylD	Metabolism and transport	Fatty acid and phospholipids	Malonyl-CoA-[acyl-carrier-protein] transacylase (EC 2.3.1.39)	2,128	0,000	1,044	0,700	1,850	0,000	0,999	0,994
1031686	COG1028IQR	gbs0646	cylG	Metabolism and transport	Fatty acid and phospholipids	3-oxoacyl-[acyl-carrier protein] reductase (EC 1.1.1.100)	1,748	0,001	1,036	0,750	1,826	0,000	1,226	0,020
1031687	COG0236IQ	gbs0647	acpC	Metabolism and transport	Fatty acid and phospholipids	Acyl carrier protein	1,203	0,130	0,761	0,068	1,243	0,076	0,964	0,736
1031692	COG0764I	gbs0648	cylZ	Metabolism and transport	Fatty acid and phospholipids	(3R)-hydroxymyristoyl-[acyl carrier protein] dehydratase (EC 4.2.1.-)	1,213	0,020	1,094	0,296	1,298	0,017	1,321	0,019
1031693	COG1131V	gbs0649	cylA	Transport and binding proteins	Unknown substrate	Hemolysin export ABC transporter ATP-binding protein	0,721	0,008	0,823	0,070	0,844	0,117	1,124	0,197
1031691	-	gbs0650	cylB	Transport and binding proteins	Unknown substrate	Hypothetical ABC transporter permease protein	0,566	0,001	0,760	0,092	0,658	0,006	1,173	0,173
1031430	-	gbs0651	cylE	Hypothetical		Hypothetical protein	0,596	0,002	0,976	0,851	0,618	0,002	1,452	0,012
1031690	COG0404E	gbs0652	cylF	Metabolism and transport	Amino acids, peptides, aminosugars and amines	Aminomethyltransferase (EC 2.1.2.10)	0,669	0,003	0,870	0,205	0,725	0,011	1,427	0,023
1031699	COG0304IQ	gbs0653	cylI	Metabolism and transport	Fatty acid and phospholipids	3-oxoacyl-[acyl-carrier-protein] synthase (EC 2.3.1.41) Chain length factor	0,529	0,000	1,064	0,527	0,551	0,000	1,496	0,008
1031696	COG1819GC	gbs0654	cylJ	Metabolism and transport	Carbohydrates, organic alcohols, and acids	UDP glycosyltransferase (EC 2.4.1.-)	0,455	0,000	0,953	0,747	0,408	0,000	1,629	0,018
1031698	-	gbs0655	cylK	Hypothetical		Hypothetical protein	0,355	0,000	0,989	0,956	0,316	0,000	1,734	0,014
1031700	COG0577V	gbs0657		Transport and binding proteins	Unknown substrate	ABC transporter permease protein	1,499	0,238	5,495	0,047	3,498	0,086	2,427	0,096
1031695	-	gbs0658		Hypothetical		Hypothetical protein	2,531	0,203	3,866	0,023	1,155	0,751	3,546	0,116
1031701	COG1136V	gbs0659		Transport and binding proteins	Unknown substrate	ABC transporter ATP-binding protein	2,850	0,043	6,065	0,013	2,333	0,013	2,002	0,033
1031115	COG4529S	gbs0660		Hypothetical		Hypothetical exported protein	0,798	0,003	0,797	0,112	0,567	0,002	0,888	0,574
1031706	-	gbs0661		Cellular processess	DNA transformation	DNA-entry nuclease (EC 3.1.30.-)	2,328	0,000	2,316	0,003	2,293	0,000	1,989	0,001
1031702	COG0586S	gbs0662		General function predicted only		DedA family protein	0,546	0,013	0,264	0,001	0,537	0,011	0,429	0,006
1031703	COG4619R	gbs0663		Transport and binding proteins	Unknown substrate	ABC transporter ATP-binding protein	0,406	0,004	0,144	0,000	0,368	0,002	0,206	0,001
1031704	COG0390R	gbs0664		Transport and binding proteins	Unknown substrate	ABC transporter permease protein	0,408	0,004	0,187	0,000	0,272	0,001	0,225	0,001
1031705	-	gbs0665		Hypothetical		Hypothetical protein	1,445	0,030	1,301	0,365	1,386	0,011	1,103	0,513
1031707	COG0583K	gbs0666		Cellular processess	Transcription	Transcriptional regulators, LysR family	0,894	0,286	0,507	0,004	0,647	0,047	0,558	0,002
1031052	COG3641R	gbs0667		Hypothetical		Hypothetical membrane spanning protein	0,557	0,003	0,518	0,002	0,540	0,002	0,411	0,001
1031708	COG1052CHI	gbs0668	ddh	Metabolism and transport	Central intermediary metabolism	D-2-hydroxyacid dehydrogenase (EC 1.1.1.-)	0,558	0,001	0,569	0,001	0,546	0,000	0,460	0,000
1031711	COG2211G	gbs0669		Metabolism and transport	Carbohydrates, organic alcohols, and acids	SUGAR SODIUM SYMPORTER	4,003	0,000	5,145	0,002	3,755	0,000	5,247	0,001
1031712	COG0524G	gbs0670		Metabolism and transport	Carbohydrates, organic alcohols, and acids	2-dehydro-3-deoxygluconokinase (EC 2.7.1.45)	1,819	0,181	2,297	0,020	0,811	0,594	1,842	0,109
1031721	COG3250G	gbs0671	lacZ	Metabolism and transport	Carbohydrates, organic alcohols, and acids	Beta-glucuronidase (EC 3.2.1.31)	0,580	0,158	1,047	0,883	0,926	0,774	1,161	0,661
1031715	COG2186K	gbs0672		Cellular processess	Transcription	Transcriptional regulator, GntR family	1,667	0,000	0,677	0,025	1,389	0,000	0,741	0,020
1031709	COG0800G	gbs0673		Metabolism and transport	Carbohydrates, organic alcohols, and acids	4-Hydroxy-2-oxoglutarate aldolase (EC 4.1.3.16) 2-dehydro-3-deoxyphosphoglucol	4,676	0,003	5,115	0,020	5,352	0,000	6,303	0,053

1030888	COG1904G	gbs0674		Metabolism and transport	Carbohydrates, organic alcohols, and acids	Uronate isomerase (EC 5.3.1.12)	2,303	0,079	3,326	0,099	2,785	0,105	2,911	0,301
1031717	COG1312G	gbs0675		Metabolism and transport	Carbohydrates, organic alcohols, and acids	Mannonate dehydratase (EC 4.2.1.8)	1,183	0,402	4,015	0,020	1,110	0,681	4,124	0,032
1031719	COG1028IQR	gbs0676		Metabolism and transport	Carbohydrates, organic alcohols, and acids	Fructuronate reductase (EC 1.1.1.57)	1,964	0,000	4,125	0,005	2,086	0,000	4,894	0,004
1031716	COG0546R	gbs0677		Metabolism and transport	Central intermediary metabolism	Phosphoglycolate phosphatase (EC 3.1.3.18)	0,626	0,136	4,060	0,002	0,952	0,855	4,487	0,043
1031371	-	gbs0678		Metabolism and transport	Carbohydrates, organic alcohols, and acids	Beta-N-acetylhexosaminidase (EC 3.2.1.52)	1,000	0,999	2,178	0,013	1,231	0,556	3,735	0,008
1031724	COG0006E	gbs0679	pepQ	Metabolism and transport	Amino acids, peptides, aminosugars and amines	Xaa-Pro aminopeptidase (EC 3.4.11.9)	1,143	0,148	0,838	0,152	1,073	0,429	1,081	0,322
1031718	COG1609K	gbs0680	ccpA	Cellular processess	Transcription	Catabolite control protein A	0,788	0,083	2,359	0,000	0,758	0,048	2,346	0,000
1031212	-	gbs0681		Metabolism and transport	Carbohydrates, organic alcohols, and acids	Alpha-amylase (EC 3.2.1.1)	0,803	0,026	1,235	0,021	0,764	0,022	1,628	0,000
1031321	COG0438M	gbs0682		Metabolism and transport	Central intermediary metabolism	Glycosyltransferase (EC 2.4.1.-)	0,517	0,000	0,682	0,000	0,482	0,000	0,885	0,040
1031726	COG0438M	gbs0683		Metabolism and transport	Central intermediary metabolism	1,2-diacylglycerol 3-glucosyltransferase (EC 2.4.1.157)	0,464	0,000	0,641	0,000	0,415	0,000	0,738	0,005
1031722	COG0441J	gbs0684	thrS	Cellular processess	Translation, ribosomal structure and biogenesis	Threonyl-tRNA synthetase (EC 6.1.1.3)	0,679	0,008	0,841	0,176	0,639	0,004	1,193	0,227
1031725	COG0745TK	gbs0685		Cellular processess	Transcription	Transcriptional regulatory protein	0,284	0,001	0,393	0,002	0,216	0,000	0,279	0,001
1031730	-	gbs0686		Hypothetical		Hypothetical cytosolic protein	1,211	0,024	0,977	0,794	0,907	0,290	1,064	0,485
1031723	-	gbs0687		Hypothetical		Hypothetical membrane associated protein	9,320	0,001	11,153	0,000	13,045	0,003	8,484	0,001
1031728	COG0765E	gbs0688		Metabolism and transport	Amino acids, peptides, aminosugars and amines	Glutamine transport system permease protein glnP	1,553	0,101	1,547	0,195	1,774	0,001	2,055	0,013
1031732	COG0765E	gbs0689		Metabolism and transport	Amino acids, peptides, aminosugars and amines	Glutamine transport system permease protein glnP	1,595	0,117	1,589	0,118	2,646	0,039	1,298	0,434
1031714	COG0834ET	gbs0690		Metabolism and transport	Amino acids, peptides, aminosugars and amines	Glutamine-binding protein	1,411	0,447	0,847	0,457	1,573	0,134	0,715	0,329
1031301	COG1126E	gbs0691		Metabolism and transport	Amino acids, peptides, aminosugars and amines	Glutamine transport ATP-binding protein glnQ	1,821	0,080	1,656	0,311	2,630	0,020	1,698	0,435
1031731	-	gbs0692		Hypothetical		Hypothetical protein	0,361	0,012	0,999	0,998	0,730	0,582	0,917	0,757
1031733	-	gbs0693		Hypothetical		Hypothetical protein	0,989	0,976	2,096	0,077	1,173	0,656	0,998	0,995
1031727	-	gbs0694		Hypothetical		Hypothetical protein	2,204	0,059	3,500	0,017	1,849	0,112	2,554	0,082
1031740	-	gbs0695		Hypothetical		Hypothetical protein	3,883	0,001	5,205	0,011	3,968	0,019	5,808	0,001
1031737	-	gbs0696		Hypothetical		Hypothetical protein	1,235	0,556	2,602	0,223	1,803	0,150	1,177	0,616
1031741	-	gbs0697		Hypothetical		Hypothetical protein	1,191	0,571	2,024	0,175	1,280	0,358	1,697	0,041
1031735	-	gbs0698		General function predicted only		RepR protein	0,987	0,960	2,306	0,165	1,058	0,813	1,443	0,359
1031745	-	gbs0699		Hypothetical		Hypothetical protein	0,972	0,941	0,672	0,101	1,123	0,742	0,891	0,761
1031734	-	gbs0700		Hypothetical		Hypothetical protein	1,997	0,017	3,687	0,012	2,276	0,000	2,800	0,018
1031749	-	gbs0701		Hypothetical		Hypothetical protein	0,480	0,005	1,009	0,978	0,474	0,003	0,503	0,005
1031738	-	gbs0702		Hypothetical		Hypothetical protein	0,747	0,191	1,774	0,453	0,753	0,412	0,689	0,040
1031743	-	gbs0703		Hypothetical		Hypothetical protein	1,983	0,071	2,559	0,164	1,112	0,698	1,804	0,177
1031753	-	gbs0708		Hypothetical		Hypothetical protein	1,192	0,208	3,443	0,022	1,562	0,215	1,882	0,023
1031750	COG0542O	gbs0718		Hypothetical		Hypothetical protein	1,925	0,000	2,122	0,016	2,145	0,012	2,183	0,002
1031769	-	gbs0727		Hypothetical		Hypothetical protein	5,193	0,001	2,409	0,001	5,648	0,001	2,691	0,012
1030882	-	gbs0728		Hypothetical		Hypothetical protein	3,773	0,002	2,517	0,000	3,981	0,001	2,475	0,022
1031772	COG0272L	gbs0729		Hypothetical		Hypothetical protein	2,543	0,000	2,238	0,026	2,826	0,002	2,207	0,002
1031768	-	gbs0730		Hypothetical		Hypothetical protein	2,414	0,000	1,123	0,709	2,074	0,000	1,101	0,661
1031771	-	gbs0731		Hypothetical		Hypothetical protein	2,468	0,000	2,237	0,032	2,406	0,002	1,985	0,005
1030878	COG0745TK	gbs0741	vicR	Cellular processess	Signal transduction	Two-component response regulator VicR	0,689	0,001	1,093	0,332	0,617	0,000	1,496	0,001
1031774	COG5002T	gbs0742	vicK	Cellular processess	Signal transduction	Two-component sensor histidine kinase VicK (EC 2.7.3.-)	0,501	0,000	1,283	0,044	0,502	0,000	1,616	0,000
1031197	COG1235R	gbs0743	vicX	General function predicted only		Zn-dependent hydrolase (beta-lactamase superfamily)	0,222	0,000	1,167	0,161	0,206	0,000	1,772	0,000
1030918	COG2832S	gbs0744		Hypothetical		Hypothetical membrane spanning protein	0,272	0,000	1,334	0,004	0,275	0,000	1,687	0,000
1031165	COG0571K	gbs0745	rncS	Cellular processess	Transcription	Ribonuclease III (EC 3.1.26.3)	0,312	0,000	0,592	0,004	0,382	0,000	0,584	0,001
1031776	COG1196D	gbs0746	smc	Cellular processess	Cell division	Chromosome partition protein smc	0,384	0,000	0,630	0,017	0,427	0,000	0,817	0,266
1030834	COG0561R	gbs0747		Metabolism and transport	Central intermediary metabolism	Hydrolase (HAD superfamily)	0,556	0,001	0,975	0,886	0,574	0,001	1,127	0,179
1031298	COG0561R	gbs0748		Metabolism and transport	Central intermediary metabolism	Hydrolase (HAD superfamily)	0,471	0,000	0,806	0,081	0,468	0,000	1,043	0,617
1031780	COG0552U	gbs0749	ftsY	Cellular processess	Cell division	Cell division protein ftsY	0,520	0,000	0,987	0,891	0,558	0,000	1,262	0,006
1031777	COG3689S	gbs0750		Hypothetical		Hypothetical membrane spanning protein	0,396	0,000	0,245	0,000	0,352	0,000	0,289	0,000
1031216	COG0701R	gbs0751		Hypothetical		Hypothetical membrane spanning protein	0,402	0,000	0,351	0,000	0,345	0,000	0,322	0,000
1031779	-	gbs0752		Hypothetical		Hypothetical protein	0,520	0,000	0,410	0,000	0,458	0,000	0,317	0,000
1031151	COG2141C	gbs0753		Metabolism and transport	Energy production and conversion	Luciferase-like monooxygenase (EC 1.14.-.-)	3,215	0,023	1,844	0,030	3,541	0,032	2,378	0,070
1031205	COG2183K	gbs0754		Cellular processess	Transcription	TRANSCRIPTION ACCESSORY PROTEIN (S1 RNA binding domain)	0,239	0,000	0,435	0,000	0,306	0,000	0,681	0,001
1031050	-	gbs0755		Cellular processess	Toxin production and resistance	Metallopeptidase, SprT family (EC 3.4.24.-)	0,407	0,000	0,832	0,174	0,419	0,000	0,920	0,329
1031462	COG1983KT	gbs0756		Cellular processess	Transcription	Stress-responsive transcriptional regulator PspC	1,097	0,589	3,115	0,007	1,355	0,193	3,136	0,034
1031783	COG1493T	gbs0757	ptsK	Cellular processess	Signal transduction	HPR(SER) KINASE (EC 2.7.1.-) PHOSPHATASE (EC 3.1.3.-)	2,125	0,000	1,939	0,006	2,081	0,000	1,454	0,071
1031041	-	gbs0758	lgt	Cellular processess	Posttranslational modification, protein turnover, chaperones	Prolipoprotein diacylglyceryl transferase (EC 2.4.99.-)	1,199	0,133	1,609	0,015	1,268	0,087	1,213	0,198
1031264	COG4768R	gbs0759		Hypothetical		Hypothetical protein	1,599	0,007	2,583	0,001	1,506	0,010	2,047	0,002
1031781	-	gbs0760		Hypothetical		Hypothetical exported protein	1,477	0,030	2,400	0,002	1,344	0,037	2,079	0,002
1031786	-	gbs0761		Hypothetical		Hypothetical protein	0,384	0,000	0,779	0,013	0,395	0,000	0,941	0,381
1031183	-	gbs0762		Cellular processess	Posttranslational modification, protein turnover, chaperones	Peptidase family U32	0,315	0,000	0,643	0,006	0,329	0,000	0,605	0,003
1031342	-	gbs0763		Cellular processess	Posttranslational modification, protein turnover, chaperones	Peptidase family U32	0,268	0,000	0,586	0,064	0,279	0,001	0,481	0,009
1031363	COG4443S	gbs0764		Hypothetical		Hypothetical cytosolic protein	1,494	0,000	0,707	0,035	1,363	0,000	0,829	0,031
1031785	-	gbs0765		Hypothetical		Hypothetical protein	2,538	0,000	2,307	0,005	2,597	0,000	1,766	0,001
1031379	COG1914P	gbs0766		Metabolism and transport	Inorganic ion transport and metabolism	Manganese transport protein mntH	0,710	0,005	0,568	0,001	0,594	0,001	0,698	0,004
1031104	COG0117H, C	gbs0767		Metabolism and transport	Cofactors, prosthetic groups, and carriers	Diaminohydroxyphosphoribosylaminopyrimidine deaminase (EC 3.5.4.26) 5-amino-	1,009	0,980	0,360	0,068	0,993	0,985	0,321	0,056
1031789	COG0307H	gbs0768		Metabolism and transport	Cofactors, prosthetic groups, and carriers	Riboflavin synthase alpha chain (EC 2.5.1.9)	1,973	0,109	0,520	0,120	1,798	0,121	0,308	0,035
1031790	COG0807H	gbs0769		Metabolism and transport	Cofactors, prosthetic groups, and carriers	GTP cyclohydrolase II (EC 3.5.4.25) 3,4-dihydroxy-2-butanone-4-phosphate syntha	2,238	0,061	0,608	0,162	1,979	0,076	0,459	0,059
1031373	-	gbs0770		Metabolism and transport	Cofactors, prosthetic groups, and carriers	6,7-dimethyl-8-ribityllumazine synthase (EC 2.5.1.9)	2,807	0,040	0,649	0,191	2,661	0,028	0,421	0,043
1031784	COG1190J	gbs0771	lysS	Cellular processess	Translation, ribosomal structure and biogenesis	Lysyl-tRNA synthetase (EC 6.1.1.6)	0,388	0,000	0,578	0,000	0,375	0,000	0,540	0,000
1031788	COG1011R	gbs0772		Metabolism and transport	Central intermediary metabolism	Haloacid dehalogenase-like hydrolase	0,488	0,000	0,250	0,000	0,280	0,000	0,308	0,000
1031445	COG0406G	gbs0773		Metabolism and transport	Carbohydrates, organic alcohols, and acids	Phosphoglycerate mutase family protein	2,293	0,000	2,107	0,007	2,612	0,001	1,751	0,004
1031793	COG2606S	gbs0774		Cellular processess	Transcription	Transcriptional regulator	1,951	0,001	1,325	0,247	1,852	0,020	1,175	0,319
1031787	COG0451MG	gbs0775		General function predicted only		NADH-ubiquinone oxidoreductase 39-40 kDa subunit homolog	0,935	0,709	0,418	0,002	1,138	0,574	1,280	0,312
1031042	COG3757M	gbs0776		Cellular processess	Posttranslational modification, protein turnover, chaperones	N-acetylmuramoyl-L-alanine amidase (EC 3.5.1.28)	0,533	0,000	0,561	0,000	0,469	0,000	0,566	0,000
1031791	-	gbs0777		Hypothetical		Integral membrane protein	1,168	0,135	0,760	0,196	0,599	0,002	0,968	0,832
1030905	-	gbs0778		Hypothetical		Hypothetical exported protein	2,081	0,000	1,010	0,939	1,695	0,001	0,647	0,002
1031466	COG1164E	gbs0779	pepF	Metabolism and transport	Amino acids, peptides, aminosugars and amines	Oligoendopeptidase F (EC 3.4.24.-)	0,688	0,000	0,441	0,000	0,705	0,001	0,414	0,000
1031917	COG2352C	gbs0780	ppc	Metabolism and transport	Energy production and conversion	Phosphoenolpyruvate carboxylase (EC 4.1.1.31)	0,382	0,000	0,231	0,000	0,360	0,000	0,249	0,000
1031792	-	gbs0781	ftsW	Cellular processess	Cell division	Cell division protein ftsW	0,384	0,000	0,684	0,108	0,276	0,000	0,584	0,007
1031913	COG0050J	gbs0782	tuf	Cellular processess	Translation, ribosomal structure and biogenesis	Protein Translation Elongation Factor Tu (EF-TU)	2,300	0,000	2,622	0,000	2,500	0,000	2,765	0,000
1031916	COG0149G	gbs0783	tpiA	Metabolism and transport	Carbohydrates, organic alcohols, and acids	Triosephosphate isomerase (EC 5.3.1.1)	1,101	0,061	0,975	0,591	1,113	0,176	1,201	0,066
1031918	COG0588G	gbs0784	gpmA	Metabolism and transport	Carbohydrates, organic alcohols, and acids	Phosphoglycerate mutase (EC 5.4.2.1)	1,050	0,436	0,942	0,376	1,003	0,929	1,085	0,217
1031914	COG0768M	gbs0785		Cell Envelope	Biosynthesis and degradation of murein sacculus and peptidoglycar	Penicillin-binding protein	0,353	0,000	0,290	0,000	0,308	0,000	0,408	0,000
1031920	COG0353L	gbs0786	recR	Cellular processess	DNA replication, recombination and repair	Recombination protein recR	0,353	0,000	0,421	0,000	0,342	0,000	0,335	0,000
1031911	COG1181M	gbs0787	ddl	Cell Envelope	Biosynthesis and degradation of murein sacculus and peptidoglycar	D-alanine--D-alanine ligase (EC 6.3.2.4)	0,224	0,000	0,380	0,000	0,207	0,000	0,361	0,000
1031919	COG0770M	gbs0788	murF	Cell Envelope	Biosynthesis and degradation of murein sacculus and peptidoglycar	UDP-N-acetylmuramoyl-tripeptide--D-alanyl-D-alanine ligase (EC 6.3.2.10)	0,465	0,000	0,838	0,011	0,514	0,000	0,846	0,011
1031915	-	gbs0789		Transport and binding proteins	Unknown substrate	Transporter, MFS superfamily	0,458	0,015	0,141	0,002	0,401	0,010	0,069	0,001
1031924	COG5522S	gbs0790		Hypothetical		Hypothetical membrane spanning protein	0,217	0,000	0,349	0,000	0,208	0,000	0,352	0,000
1031910	-	gbs0791		Hypothetical		LPXTG Surface anchor protein	0,517	0,000	0,926	0,503	0,588	0,001	0,805	0,115

1031905	COG4108J	gbs0792	prfC	Cellular processess	Translation, ribosomal structure and biogenesis	Bacterial Peptide Chain Release Factor 3 (RF-3)	0,446	0,000	0,708	0,003	0,507	0,000	0,672	0,001
1031912	-	gbs0793		Hypothetical		Hypothetical cytosolic protein	0,804	0,061	0,327	0,000	0,610	0,004	0,416	0,000
1031922	COG1135P	gbs0794		Metabolism and transport	Inorganic ion transport and metabolism	Probable D-methionine transport ATP-binding protein metN	1,139	0,441	1,048	0,617	1,104	0,609	0,966	0,786
1031921	COG2011P	gbs0795		Transport and binding proteins	Unknown substrate	ABC transporter permease protein	0,461	0,000	0,720	0,026	0,409	0,000	0,648	0,028
1031923	COG1464P	gbs0796		Transport and binding proteins	Unknown substrate	ABC transporter substrate-binding protein	0,829	0,087	1,014	0,903	0,736	0,018	0,888	0,266
1031907	COG0513LKJ	gbs0797	deaD	Cellular processess	DNA replication, recombination and repair	ATP-dependent RNA helicase	0,239	0,000	0,166	0,000	0,239	0,000	0,108	0,000
1031909	COG2827L	gbs0798		Hypothetical		Hypothetical protein with endo excinuclease domain	0,608	0,003	0,696	0,008	0,605	0,001	0,860	0,138
1031906	COG4123R	gbs0799		Metabolism and transport	Central intermediary metabolism	Methyltransferase (EC 2.1.1.-)	0,354	0,000	0,453	0,002	0,345	0,000	0,551	0,007
1031926	COG0204I	gbs0800		Metabolism and transport	Fatty acid and phospholipids	1-acyl-sn-glycerol-3-phosphate acyltransferase (EC 2.3.1.51)	0,578	0,000	0,764	0,000	0,588	0,000	0,758	0,013
1031925	COG1555L	gbs0801		Cellular processess	DNA transformation	COME operon protein 1	3,065	0,016	11,253	0,041	1,952	0,210	8,886	0,088
1031903	COG0658R, (	gbs0802	comEC	General function predicted only		COME operon protein 3	1,462	0,153	3,781	0,017	1,651	0,247	1,581	0,229
1031902	COG0561R	gbs0803		Metabolism and transport	Central intermediary metabolism	Hydrolase (HAD superfamily)	1,183	0,420	1,166	0,515	1,344	0,100	1,754	0,002
1031904	COG1609K	gbs0804		Cellular processess	Transcription	Catabolite control protein B	1,306	0,026	1,575	0,000	1,341	0,065	1,642	0,005
1031908	COG0657I	gbs0805		Metabolism and transport	Fatty acid and phospholipids	Esterase (EC 3.1.1.-)	0,198	0,000	0,367	0,026	0,146	0,000	0,213	0,000
1031888	COG0500QR	gbs0806		Metabolism and transport	Secondary metabolites	Biotin synthesis protein bioC	2,084	0,006	2,292	0,016	2,459	0,004	2,117	0,004
1031901	COG1466L	gbs0807		Cellular processess	DNA replication, recombination and repair	DNA polymerase III, delta subunit (EC 2.7.7.7)	0,489	0,000	0,891	0,249	0,546	0,000	1,069	0,462
1031927	COG0605P	gbs0808	sodA	Metabolism and transport	Inorganic ion transport and metabolism	Superoxide dismutase (EC 1.15.1.1)	2,431	0,000	1,618	0,000	2,475	0,000	1,177	0,112
1031900	COG3711K	gbs0809	licT	Cellular processess	Transcription	Transcription antiterminator, BglG family	1,987	0,188	16,514	0,110	2,529	0,151	9,582	0,091
1031929	COG1263G	gbs0810		Metabolism and transport	Carbohydrates, organic alcohols, and acids	PTS system, beta-glucoside-specific IIABC component (EC 2.7.1.69)	2,268	0,264	21,825	0,086	1,370	0,395	8,767	0,216
1031897	-	gbs0811	bglA	Metabolism and transport	Carbohydrates, organic alcohols, and acids	6-phospho-beta-glucosidase (EC 3.2.1.86)	1,077	0,701	4,628	0,079	1,343	0,207	3,594	0,042
1031899	COG3835KT	gbs0812		Cellular processess	Transcription	Transcriptional regulatory protein	1,164	0,260	0,955	0,871	1,169	0,287	1,695	0,001
1031896	-	gbs0813		Metabolism and transport	Energy production and conversion	Glycerate kinase (EC 2.7.1.31)	4,702	0,044	3,308	0,000	4,804	0,015	8,340	0,021
1031898	COG2610GE	gbs0814		Metabolism and transport	Carbohydrates, organic alcohols, and acids	Gluconate permease	1,874	0,005	3,020	0,000	2,207	0,009	3,294	0,000
1031893	COG1686M, (	gbs0815		Cellular processess	Posttranslational modification, protein turnover, chaperones	Peptidase family S11	0,406	0,000	0,854	0,438	0,604	0,024	1,308	0,012
1031928	COG1846K	gbs0816		Cellular processess	Transcription	Transcriptional regulator, MarR family	0,622	0,000	0,302	0,000	0,556	0,000	0,341	0,000
1031930	-	gbs0817	queA	Cellular processess	Translation, ribosomal structure and biogenesis	S-adenosylmethionine:tRNA ribosyltransferase-isomerase (EC 5.-.-.-)	0,620	0,000	0,677	0,003	0,531	0,000	0,646	0,000
1031894	COG1814S	gbs0818		Hypothetical		Hypothetical membrane associated protein	1,047	0,764	1,075	0,733	1,023	0,888	1,307	0,148
1031895	COG0363G	gbs0819	nagB	Metabolism and transport	Carbohydrates, organic alcohols, and acids	Glucosamine-6-phosphate isomerase (EC 3.5.99.6)	0,633	0,017	0,333	0,001	0,662	0,025	0,377	0,001
1031890	COG0435O	gbs0820		Metabolism and transport	Central intermediary metabolism	Glutathione S-transferase family protein	1,250	0,390	0,967	0,886	1,426	0,211	1,117	0,700
1031932	COG1187J	gbs0821		Cellular processess	Translation, ribosomal structure and biogenesis	Ribosomal small subunit pseudouridine synthase A (EC 4.2.1.70)	0,322	0,000	0,244	0,000	0,253	0,000	0,312	0,000
1031934	-	gbs0822		Transport and binding proteins	Unknown substrate	Transporter, MFS superfamily	0,352	0,001	0,515	0,007	0,413	0,003	0,576	0,015
1031931	COG4469R	gbs0823		Cellular processess	DNA transformation	Putative competence protein transcription factor	3,589	0,003	3,981	0,164	1,990	0,232	3,524	0,024
1029783	COG1164E	gbs0824	pepB	Metabolism and transport	Amino acids, peptides, aminosugars and amines	Oligoendopeptidase F (EC 3.4.24.-)	0,935	0,506	0,694	0,026	0,885	0,146	0,593	0,000
1029784	COG0546R	gbs0825		Metabolism and transport	Central intermediary metabolism	Phosphoglycolate phosphatase (EC 3.1.3.18)	0,403	0,000	0,522	0,116	0,374	0,000	0,373	0,000
1031933	COG4122R	gbs0826		Cellular processess	Posttranslational modification, protein turnover, chaperones	O-methyltransferase (EC 2.1.1.-)	0,292	0,000	0,256	0,000	0,305	0,000	0,338	0,000
1031892	COG0760O	gbs0827	prsA	Cellular processess	Posttranslational modification, protein turnover, chaperones	Peptidyl-prolyl cis-trans isomerase (EC 5.2.1.8)	0,589	0,000	0,401	0,000	0,514	0,000	0,394	0,000
1029786	COG4894S	gbs0828		Hypothetical		Hypothetical protein	0,392	0,000	0,377	0,000	0,379	0,000	0,325	0,000
1029789	COG0013J	gbs0829	alaS	Cellular processess	Translation, ribosomal structure and biogenesis	Alanyl-tRNA synthetase (EC 6.1.1.7)	0,481	0,000	0,444	0,000	0,516	0,000	0,538	0,000
1029810	COG1811R	gbs0830		Hypothetical		Hypothetical membrane spanning protein	0,512	0,001	0,520	0,001	0,542	0,003	0,551	0,002
1031935	COG1442M	gbs0831		Metabolism and transport	Central intermediary metabolism	Lipopolysaccharide 1,2-glucosyltransferase (EC 2.4.1.58)	0,831	0,093	1,325	0,082	0,733	0,069	1,119	0,376
1029812	-	gbs0832		Hypothetical		Hypothetical protein	5,862	0,000	5,166	0,001	5,630	0,000	10,586	0,000
1029805	COG1476K	gbs0833		Cellular processess	Transcription	Transcriptional regulator, Cro CI family	6,011	0,000	4,022	0,000	6,118	0,000	9,079	0,000
1029809	-	gbs0834		Hypothetical		Hypothetical membrane spanning protein	2,273	0,002	2,642	0,003	2,248	0,000	5,085	0,000
1029785	COG3859S	gbs0835		Transport and binding proteins	Unknown substrate	Thiamine transporter	0,595	0,001	0,817	0,251	0,504	0,003	0,609	0,016
1029811	COG0208F	gbs0836	nrdF.2	Metabolism and transport	Purines, pyrimidines, nucleosides, and nucleotides	Ribonucleoside-diphosphate reductase beta chain (EC 1.17.4.1)	0,302	0,000	0,458	0,000	0,290	0,000	0,487	0,000
1029807	COG0209F	gbs0837	nrdE.2	Metabolism and transport	Purines, pyrimidines, nucleosides, and nucleotides	Ribonucleoside-diphosphate reductase alpha chain (EC 1.17.4.1)	0,537	0,000	0,717	0,008	0,551	0,001	0,810	0,046
1029814	COG0695O	gbs0838	nrdH	Cellular processess	Posttranslational modification, protein turnover, chaperones	Glutaredoxin	0,690	0,002	0,591	0,000	0,600	0,000	0,608	0,000
1029813	COG1925G	gbs0839	ptsH	Metabolism and transport	Carbohydrates, organic alcohols, and acids	Phosphocarrier protein HPr	2,801	0,000	2,377	0,000	3,142	0,000	2,380	0,000
1029818	COG1080G	gbs0840	ptsI	Metabolism and transport	Carbohydrates, organic alcohols, and acids	Phosphoenolpyruvate-protein phosphotransferase (EC 2.7.3.9)	1,117	0,162	1,189	0,019	1,089	0,119	1,431	0,000
1029815	COG1012C	gbs0841	gapN	Metabolism and transport	Energy production and conversion	NADP-dependent glyceraldehyde-3-phosphate dehydrogenase (EC 1.2.1.9)	0,778	0,004	0,448	0,000	0,765	0,004	0,410	0,000
1029817	-	gbs0842		Cell Envelope	Biosynthesis and degradation of surface polysaccharides and lipopo	Peptidoglycan N-acetylglucosamine deacetylase (EC 3.5.1.-)	1,499	0,003	0,907	0,352	1,446	0,000	1,215	0,003
1029819	COG0513LKJ	gbs0843	deaD2	Cellular processess	DNA replication, recombination and repair	ATP-dependent RNA helicase	0,744	0,004	0,538	0,000	0,684	0,000	0,630	0,000
1029822	COG0572F	gbs0844	udk	Metabolism and transport	Purines, pyrimidines, nucleosides, and nucleotides	Uridine kinase (EC 2.7.1.48)	0,508	0,001	0,289	0,000	0,434	0,000	0,303	0,000
1029825	COG1956T	gbs0845		Cellular processess	Signal transduction	GAF domain-containing proteins	0,449	0,000	0,689	0,003	0,392	0,000	0,657	0,001
1029829	COG2812L	gbs0846		Cellular processess	DNA replication, recombination and repair	DNA polymerase III subunit gamma tau (EC 2.7.7.7)	0,486	0,000	0,517	0,000	0,437	0,000	0,560	0,000
1029820	-	gbs0847		Hypothetical		Hypothetical protein	0,417	0,000	0,851	0,335	0,407	0,000	0,660	0,005
1029826	COG0340H, (	gbs0848	birA	Cellular processess	Transcription	Biotin operon repressor Biotin--[acetyl-CoA-carboxylase] synthetase (EC 6.3.4.15)	0,530	0,000	0,709	0,049	0,534	0,000	0,912	0,441
1029821	COG0192H	gbs0849	metK2	Metabolism and transport	Cofactors, prosthetic groups, and carriers	S-adenosylmethionine synthetase (EC 2.5.1.6)	0,451	0,000	0,577	0,000	0,436	0,000	0,628	0,000
1029828	-	gbs0850	fbsB	Hypothetical		Hypothetical protein	2,266	0,060	1,644	0,121	1,604	0,244	2,299	0,215
1029823	-	gbs0851		Hypothetical		Hypothetical protein	1,370	0,370	1,333	0,463	1,501	0,135	0,753	0,407
1029834	-	gbs0852		Hypothetical		Hypothetical protein	0,518	0,001	1,108	0,582	0,481	0,011	0,887	0,541
1029824	COG4732S	gbs0853		Transport and binding proteins	Unknown substrate	Hydroxyethylthiazole permease	5,162	0,004	15,223	0,070	5,843	0,001	6,136	0,001
1029833	COG4721S	gbs0854		Transport and binding proteins	Unknown substrate	Hydroxymethylpyrimidine transport system permease protein	3,917	0,075	7,201	0,002	3,948	0,010	5,627	0,038
1029836	COG1122P	gbs0855		Metabolism and transport	Inorganic ion transport and metabolism	Transporter	1,469	0,222	5,000	0,234	2,843	0,258	2,714	0,034
1029832	COG0619P	gbs0856		Metabolism and transport	Inorganic ion transport and metabolism	Hydroxymethylpyrimidine transport system permease protein	2,054	0,127	5,867	0,006	4,841	0,035	3,376	0,112
1029838	COG0819K	gbs0857		Cellular processess	Transcription	Transcriptional activator tenA	3,806	0,000	6,270	0,025	5,105	0,001	4,051	0,001
1029827	COG0351H	gbs0858		Metabolism and transport	Cofactors, prosthetic groups, and carriers	Phosphomethylpyrimidine kinase (EC 2.7.4.7) Hydroxymethylpyrimidine kinase (EC	3,074	0,014	5,912	0,009	4,284	0,018	3,811	0,001
1029837	COG2145H	gbs0859		Metabolism and transport	Cofactors, prosthetic groups, and carriers	Hydroxyethylthiazole kinase (EC 2.7.1.50)	3,129	0,000	8,921	0,011	4,226	0,012	6,492	0,008
1029839	COG0352H	gbs0860		Metabolism and transport	Cofactors, prosthetic groups, and carriers	Thiamin-phosphate pyrophosphorylase (EC 2.5.1.3)	2,180	0,011	4,332	0,038	3,162	0,001	2,659	0,044
1029841	COG0766M	gbs0861	murZ	Cell Envelope	Biosynthesis and degradation of murein sacculus and peptidoglycar	UDP-N-acetylglucosamine 1-carboxyvinyltransferase (EC 2.5.1.7)	0,338	0,000	0,469	0,001	0,338	0,000	0,515	0,001
1029840	COG1670J	gbs0862		Cellular processess	Translation, ribosomal structure and biogenesis	Ribosomal-protein-S5-alanine acetyltransferase (EC 2.3.1.128)	0,402	0,000	0,793	0,065	0,367	0,000	0,966	0,539
1029848	COG4109K	gbs0863		Cellular processess	Transcription	Cytosolic protein containing multiple CBS domains	0,398	0,000	0,825	0,226	0,258	0,000	0,955	0,625
1029845	COG0024J	gbs0864	map	Cellular processess	Translation, ribosomal structure and biogenesis	Methionine aminopeptidase (EC 3.4.11.18)	0,381	0,000	0,778	0,014	0,405	0,000	0,927	0,415
1029846	COG1295S	gbs0865		Cellular processess	Transcription	Ribonuclease BN (EC 3.1.-.-)	0,266	0,000	0,866	0,083	0,300	0,000	1,016	0,854
1029847	COG2246S	gbs0866		General function predicted only		Bactoprenol-linked monosaccharide translocase (flippase type)	0,430	0,006	0,822	0,326	0,461	0,003	0,731	0,260
1029842	COG4708S	gbs0867		Hypothetical		Hypothetical membrane spanning protein	0,664	0,001	0,362	0,000	0,683	0,001	0,315	0,000
1029844	COG0272L	gbs0868	lig	Cellular processess	DNA replication, recombination and repair	NAD-dependent DNA ligase (EC 6.5.1.2)	0,789	0,060	0,896	0,508	0,669	0,002	1,002	0,981
1029843	COG1597IR	gbs0869		Metabolism and transport	Fatty acid and phospholipids	Diacylglycerol kinase family protein	0,491	0,000	0,703	0,001	0,453	0,000	0,810	0,025
1029853	COG1523G	gbs0870		Metabolism and transport	Carbohydrates, organic alcohols, and acids	Pullulanase (EC 3.2.1.41)	0,424	0,000	0,696	0,007	0,407	0,000	0,947	0,445
1029852	COG0296G	gbs0871		Metabolism and transport	Carbohydrates, organic alcohols, and acids	1,4-alpha-glucan branching enzyme (EC 2.4.1.18)	1,453	0,116	4,110	0,002	1,455	0,092	3,456	0,000
1029849	COG0448G	gbs0872	glgC	Metabolism and transport	Carbohydrates, organic alcohols, and acids	Glucose-1-phosphate adenylyltransferase catalytic subunit (EC 2.7.7.27)	1,397	0,174	4,305	0,000	1,445	0,101	3,491	0,000
1029851	COG0448G	gbs0873		Metabolism and transport	Carbohydrates, organic alcohols, and acids	Glucose-1-phosphate adenylyltransferase regulatory subunit (EC 2.7.7.27)	1,262	0,347	4,510	0,000	1,326	0,184	3,582	0,000
1029850	COG0297G	gbs0874		Metabolism and transport	Carbohydrates, organic alcohols, and acids	Glycogen synthase (EC 2.4.1.21)	0,775	0,287	4,391	0,000	0,808	0,379	3,167	0,000
1029854	-	gbs0875	atpE	Metabolism and transport	Energy production and conversion	ATP synthase C chain (EC 3.6.3.14)	0,836	0,060	0,950	0,542	0,778	0,009	0,923	0,426
1029856	-	gbs0876	atpB	Metabolism and transport	Energy production and conversion	ATP synthase A chain (EC 3.6.3.14)	0,468	0,000	0,772	0,000	0,465	0,000	0,869	0,010
1029857	COG0711C	gbs0877	atpF	Metabolism and transport	Energy production and conversion	ATP synthase B chain (EC 3.6.3.14)	0,495	0,000	0,853	0,032	0,444	0,000	0,848	0,016
1029859	COG0712C	gbs0878	atpH	Metabolism and transport	Energy production and conversion	ATP synthase delta chain (EC 3.6.3.14)	0,291	0,000	0,614	0,000	0,292	0,000	0,577	0,000
1029855	COG0056C	gbs0879	atpA	Metabolism and transport	Energy production and conversion	ATP synthase alpha chain (EC 3.6.3.14)	0,509	0,000	0,741	0,000	0,491	0,000	0,731	0,001

1029865	COG0224C	gbs0880	atpG	Metabolism and transport	Energy production and conversion	ATP synthase gamma chain (EC 3.6.3.14)	0,423	0,000	0,649	0,000	0,415	0,000	0,629	0,000
1029858	COG0055C	gbs0881	atpD	Metabolism and transport	Energy production and conversion	ATP synthase beta chain (EC 3.6.3.14)	0,509	0,000	0,719	0,002	0,524	0,000	0,779	0,005
1029863	-	gbs0882	atpC	Metabolism and transport	Energy production and conversion	ATP synthase epsilon chain (EC 3.6.3.14)	0,668	0,001	0,880	0,153	0,690	0,002	0,937	0,416
1029866	COG0766M	gbs0883	murA	Cell Envelope	Biosynthesis and degradation of murein sacculus and peptidoglycar	UDP-N-acetylglucosamine 1-carboxyvinyltransferase (EC 2.5.1.7)	0,477	0,000	0,516	0,000	0,475	0,000	0,623	0,000
1029867	-	gbs0884	epuA	General function predicted only		EpuA protein	0,638	0,003	0,920	0,487	0,821	0,131	0,890	0,227
1029861	-	gbs0885	endA	Cellular processess	DNA transformation	DNA-entry nuclease (EC 3.1.30.-)	0,171	0,000	0,140	0,000	0,174	0,000	0,294	0,000
1029860	COG0016J	gbs0886	pheS	Cellular processess	Translation, ribosomal structure and biogenesis	Phenylalanyl-tRNA synthetase alpha chain (EC 6.1.1.20)	0,424	0,000	0,500	0,000	0,378	0,000	0,524	0,000
1029864	-	gbs0887	pheT	Metabolism and transport	Central intermediary metabolism	Acetyltransferase, GNAT family	0,420	0,000	0,466	0,000	0,393	0,000	0,516	0,000
1029872	COG0072J, C	gbs0888	pheT	Cellular processess	Translation, ribosomal structure and biogenesis	Phenylalanyl-tRNA synthetase beta chain (EC 6.1.1.20)	0,589	0,002	0,808	0,066	0,649	0,005	0,760	0,025
1029871	COG3857L	gbs0890	rexB	Cellular processess	DNA replication, recombination and repair	ATP-dependent nuclease subunit B	0,418	0,000	0,540	0,011	0,286	0,000	0,733	0,067
1029875	COG1074L	gbs0891	rexA	Cellular processess	DNA replication, recombination and repair	ATP-dependent nuclease subunit A	0,435	0,000	0,921	0,457	0,457	0,000	0,932	0,516
1029873	COG0598P	gbs0892		Metabolism and transport	Inorganic ion transport and metabolism	Magnesium and cobalt transport protein corA	0,080	0,000	0,261	0,000	0,091	0,000	0,712	0,017
1029874	COG0486R	gbs0893	thdF	Cellular processess	Translation, ribosomal structure and biogenesis	tRNA (5-carboxymethylaminomethyl-2-thiouridylate) synthase	0,626	0,001	0,972	0,790	0,567	0,000	0,792	0,053
1029870	COG0488R	gbs0894		Transport and binding proteins	Unknown substrate	ABC transporter ATP-binding protein	0,244	0,000	0,419	0,000	0,163	0,000	0,663	0,001
1029869	COG1071C	gbs0895	acoA	Metabolism and transport	Energy production and conversion	Pyruvate dehydrogenase E1 component alpha subunit (EC 1.2.4.1)	1,173	0,090	2,063	0,000	1,219	0,044	1,937	0,000
1029878	COG0022C	gbs0896	acoB	Metabolism and transport	Energy production and conversion	Pyruvate dehydrogenase E1 component beta subunit (EC 1.2.4.1)	0,738	0,018	1,934	0,000	0,819	0,079	1,916	0,000
1029876	COG0508C	gbs0897	acoC	Metabolism and transport	Energy production and conversion	Dihydrolipoamide acetyltransferase component of pyruvate dehydrogenase complex	0,539	0,000	1,665	0,000	0,548	0,000	1,924	0,000
1029879	COG1249C	gbs0898	acoL	Metabolism and transport	Energy production and conversion	Dihydrolipoamide dehydrogenase (EC 1.8.1.4)	0,613	0,000	1,528	0,000	0,662	0,000	1,718	0,000
1029885	COG0095H	gbs0899	lplB	Metabolism and transport	Cofactors, prosthetic groups, and carriers	Lipoate-protein ligase A (EC 6.3.2.-)	0,569	0,000	1,487	0,001	0,593	0,000	1,865	0,000
1029877	COG3442R	gbs0900		General function predicted only		CobB CobQ-like glutamine amidotransferase domain	0,487	0,000	0,393	0,000	0,484	0,000	0,430	0,000
1029887	COG0769M	gbs0901		Cell Envelope	Biosynthesis and degradation of murein sacculus and peptidoglycar	UDP-N-acetylmuramoylalanyl-D-glutamate--2,6-diaminopimelate ligase (EC 6.3.2.13	0,331	0,000	0,420	0,000	0,374	0,000	0,485	0,000
1029881	-	gbs0902		Hypothetical		Hypothetical membrane spanning protein	1,368	0,043	0,976	0,744	1,395	0,097	1,017	0,841
1029884	COG4856S	gbs0903		Hypothetical		Hypothetical membrane associated protein	0,914	0,447	0,907	0,374	1,154	0,388	0,910	0,161
1029882	COG1109G	gbs0904	femD	Metabolism and transport	Carbohydrates, organic alcohols, and acids	Phosphoglucosamine mutase (EC 5.4.2.10)	0,693	0,001	1,017	0,822	0,797	0,032	0,985	0,822
1029890	COG3272S	gbs0905		Hypothetical		Hypothetical protein	0,828	0,374	0,834	0,490	0,981	0,918	0,952	0,815
1029886	-	gbs0906		Hypothetical		Hypothetical protein	0,879	0,354	0,884	0,594	0,902	0,429	0,766	0,175
1029883	COG0635H	gbs0907	hemN	Metabolism and transport	Cofactors, prosthetic groups, and carriers	Oxygen-independent coproporphyrinogen-III oxidase (EC 1.3.99.22)	0,356	0,000	0,784	0,219	0,398	0,000	0,774	0,049
1029889	COG3884I	gbs0908		Metabolism and transport	Fatty acid and phospholipids	Acyl-[acyl-carrier-protein] hydrolase (EC 3.1.2.14)	0,235	0,000	0,541	0,003	0,254	0,000	0,668	0,004
1029888	COG0647G	gbs0909		Metabolism and transport	Carbohydrates, organic alcohols, and acids	Haloacid dehalogenase-like hydrolase	0,630	0,000	0,980	0,882	0,569	0,000	0,906	0,194
1029895	-	gbs0910		Hypothetical		Hypothetical membrane spanning protein	0,202	0,000	0,475	0,002	0,213	0,000	0,388	0,000
1029893	COG3513S	gbs0911		Hypothetical		Hypothetical cytosolic protein	0,627	0,018	1,941	0,001	0,647	0,022	3,108	0,000
1029891	COG1518L	gbs0912		Hypothetical		Hypothetical cytosolic protein	0,220	0,000	1,578	0,000	0,241	0,000	3,058	0,000
1029900	COG3512S	gbs0913		Hypothetical		Hypothetical cytosolic protein	0,335	0,000	1,676	0,001	0,365	0,000	3,263	0,000
1029894	-	gbs0914		Hypothetical		Hypothetical protein	0,370	0,000	1,856	0,003	0,347	0,000	3,541	0,000
1029892	-	gbs0915		Hypothetical		Hypothetical protein	0,891	0,377	2,425	0,064	1,208	0,359	1,897	0,017
1029897	-	gbs0916	ndk	Metabolism and transport	Purines, pyrimidines, nucleosides, and nucleotides	Nucleoside diphosphate kinase (EC 2.7.4.6)	2,945	0,000	0,792	0,117	2,551	0,000	0,450	0,000
1029896	COG0481M	gbs0917	lepA	General function predicted only		GTP-binding protein lepA	1,118	0,257	0,807	0,102	1,027	0,755	0,902	0,219
1029899	COG4886S	gbs0918	inlA/blr	Cellular processess	Toxin production and resistance	Internalin protein	0,832	0,423	1,336	0,376	0,508	0,005	1,153	0,616
1029902	COG1418R	gbs0919		Cellular processess	Toxin production and resistance	Metal dependent hydrolase	0,199	0,000	0,205	0,000	0,179	0,000	0,144	0,000
1029898	-	gbs0920		Metabolism and transport	Central intermediary metabolism	Acetyltransferase (EC 2.3.1.-)	0,426	0,002	0,257	0,000	0,334	0,001	0,328	0,001
1029905	-	gbs0921	msrB	Cellular processess	Posttranslational modification, protein turnover, chaperones	Peptide methionine sulfoxide reductase msrB (EC 1.8.4.6)	0,811	0,028	0,467	0,000	0,735	0,006	0,579	0,000
1029904	COG0474P	gbs0922		Metabolism and transport	Inorganic ion transport and metabolism	Calcium-transporting ATPase (EC 3.6.3.8)	1,302	0,245	1,997	0,004	1,242	0,412	1,699	0,018
1029903	-	gbs0923		Metabolism and transport	Fatty acid and phospholipids	Lipase	0,388	0,002	0,779	0,170	0,434	0,003	0,912	0,543
1029901	COG0110R	gbs0924		General function predicted only		O-acetyltransferase (EC 2.3.1.-)	0,359	0,000	0,738	0,005	0,302	0,000	0,693	0,079
1029906	COG0398S	gbs0925		General function predicted only		Putative membrane-associated alkaline phosphatase	0,465	0,000	0,516	0,000	0,384	0,000	0,641	0,000
1029908	-	gbs0926		Transport and binding proteins	Unknown substrate	ABC transporter permease protein	1,148	0,076	0,677	0,001	1,080	0,273	0,611	0,000
1029912	COG1131V	gbs0927		Transport and binding proteins	Unknown substrate	ABC transporter ATP-binding protein	1,379	0,009	0,553	0,001	1,335	0,009	0,494	0,000
1029907	COG1725K	gbs0928		Cellular processess	Transcription	Transcriptional regulator, GntR family	2,681	0,000	0,698	0,010	2,377	0,000	0,533	0,000
1029916	COG0587L	gbs0929	dnaE	Cellular processess	DNA replication, recombination and repair	DNA polymerase III alpha subunit (EC 2.7.7.7)	0,210	0,000	0,186	0,000	0,200	0,000	0,232	0,000
1029911	COG0205G	gbs0930	pfkA	Metabolism and transport	Carbohydrates, organic alcohols, and acids	6-phosphofructokinase (EC 2.7.1.11)	0,712	0,000	0,561	0,000	0,680	0,000	0,689	0,000
1029910	-	gbs0931	pyk	Metabolism and transport	Energy production and conversion	Pyruvate kinase (EC 2.7.1.40)	0,805	0,009	0,761	0,015	0,873	0,097	0,855	0,067
1029917	-	gbs0932	sipC	Cellular processess	Posttranslational modification, protein turnover, chaperones	Signal peptidase I (EC 3.4.21.89)	2,872	0,001	1,848	0,001	3,373	0,001	1,580	0,003
1029918	COG0449M	gbs0933	glmS	Metabolism and transport	Amino acids, peptides, aminosugars and amines	Glucosamine--fructose-6-phosphate aminotransferase [isomerizing] (EC 2.6.1.16)	2,081	0,056	4,378	0,005	1,975	0,030	5,592	0,000
1029915	COG2824P	gbs0934		Metabolism and transport	Inorganic ion transport and metabolism	PhnA protein	0,323	0,000	0,383	0,001	0,209	0,000	0,640	0,015
1029909	COG0765E	gbs0935		Metabolism and transport	Amino acids, peptides, aminosugars and amines	Histidine transport system permease protein hisM	0,130	0,000	0,278	0,000	0,126	0,000	0,312	0,000
1029913	COG1126E	gbs0936		Metabolism and transport	Amino acids, peptides, aminosugars and amines	Histidine transport ATP-binding protein hisP	0,140	0,000	0,250	0,000	0,154	0,000	0,370	0,000
1029921	COG0834ET	gbs0937		Metabolism and transport	Amino acids, peptides, aminosugars and amines	Histidine-binding protein	0,198	0,000	0,332	0,000	0,166	0,000	0,413	0,000
1029920	COG0268J	gbs0938	rpsT	Cellular processess	Translation, ribosomal structure and biogenesis	SSU ribosomal protein S20P	2,037	0,000	0,852	0,314	1,814	0,001	0,755	0,014
1029914	COG1072H	gbs0939	coaA	Metabolism and transport	Cofactors, prosthetic groups, and carriers	Pantothenate kinase (EC 2.7.1.33)	0,343	0,000	0,674	0,002	0,303	0,000	0,544	0,000
1029926	COG2813J	gbs0940		Cellular processess	Translation, ribosomal structure and biogenesis	16S rRNA m(2)G 1207 methyltransferase (EC 2.1.1.52)	0,279	0,000	0,615	0,026	0,204	0,000	1,067	0,515
1029919	COG0295F	gbs0941	cdd	Metabolism and transport	Purines, pyrimidines, nucleosides, and nucleotides	Cytidine deaminase (EC 3.5.4.5)	0,517	0,000	1,081	0,478	0,467	0,000	1,300	0,007
1029925	COG1744R	gbs0942		Metabolism and transport	Purines, pyrimidines, nucleosides, and nucleotides	Nucleoside-binding protein	2,351	0,000	1,663	0,000	2,621	0,000	1,641	0,000
1029922	COG3845R	gbs0943		Metabolism and transport	Purines, pyrimidines, nucleosides, and nucleotides	Nucleoside transport ATP-binding protein	0,500	0,003	0,867	0,348	0,502	0,003	1,039	0,748
1029930	COG4603R	gbs0944		Metabolism and transport	Purines, pyrimidines, nucleosides, and nucleotides	Nucleoside transport system permease protein	0,410	0,003	0,671	0,044	0,434	0,004	0,796	0,180
1029927	COG1079R	gbs0945		Metabolism and transport	Purines, pyrimidines, nucleosides, and nucleotides	Nucleoside transport system permease protein	0,490	0,003	0,830	0,234	0,482	0,003	0,908	0,500
1029923	COG0446R	gbs0946	nox	Metabolism and transport	Energy production and conversion	NADH oxidase H2O-forming (EC 1.6.-.-)	0,564	0,000	0,690	0,000	0,645	0,000	0,724	0,003
1029928	COG0039C	gbs0947	ldh	Metabolism and transport	Energy production and conversion	L-lactate dehydrogenase (EC 1.1.1.27)	1,529	0,000	1,486	0,000	1,807	0,000	1,770	0,001
1029931	COG0188L	gbs0948	gyrA	Cellular processess	DNA replication, recombination and repair	DNA gyrase subunit A (EC 5.99.1.3)	0,297	0,000	0,734	0,013	0,341	0,000	0,864	0,036
1029933	COG3764M	gbs0949	sortA	Cellular processess	Posttranslational modification, protein turnover, chaperones	Sortase	0,255	0,000	0,545	0,000	0,245	0,000	0,723	0,000
1029924	COG0346E	gbs0950		Metabolism and transport	Amino acids, peptides, aminosugars and amines	Glyoxalase family protein	0,394	0,000	0,913	0,327	0,402	0,000	1,084	0,379
1029939	COG4086S	gbs0951		Hypothetical		Hypothetical exported protein	0,683	0,046	0,187	0,000	0,477	0,010	0,292	0,001
1029932	COG0475P	gbs0952		Metabolism and transport	Inorganic ion transport and metabolism	Na+ H+ antiporter napA	0,391	0,000	1,704	0,006	0,372	0,000	1,751	0,001
1029936	COG0518F, C	gbs0953	guaA	Metabolism and transport	Purines, pyrimidines, nucleosides, and nucleotides	GMP synthase [glutamine-hydrolyzing] (EC 6.3.5.2)	0,423	0,000	0,452	0,000	0,402	0,000	0,480	0,000
1029934	COG2188K	gbs0954		Cellular processess	Transcription	Transcriptional regulator, GntR family	0,503	0,000	0,652	0,019	0,513	0,000	0,745	0,012
1029944	COG1206J	gbs0955	gid	Cellular processess	Translation, ribosomal structure and biogenesis	Glucose inhibited division protein A	0,628	0,000	0,543	0,000	0,663	0,000	0,656	0,000
1029935	-	gbs0956		Metabolism and transport	Central intermediary metabolism	Acetyltransferase (EC 2.3.1.-)	0,665	0,001	0,613	0,016	0,671	0,002	0,795	0,022
1029942	COG1464P	gbs0957		Transport and binding proteins	Unknown substrate	ABC transporter substrate-binding protein	0,469	0,008	0,389	0,005	0,486	0,010	0,398	0,005
1029943	-	gbs0958		Hypothetical		Hypothetical protein	0,488	0,001	0,464	0,001	0,426	0,001	0,451	0,001
1029945	COG0793M	gbs0959		Cellular processess	Toxin production and resistance	Nisin-resistance protein	4,835	0,000	4,296	0,020	6,314	0,005	2,882	0,000
1029937	COG1136V	gbs0961		Transport and binding proteins	Unknown substrate	ABC transporter ATP-binding protein	1,088	0,469	1,080	0,674	1,036	0,771	1,059	0,704
1029948	-	gbs0962	salY	Transport and binding proteins	Unknown substrate	ABC transporter permease protein	0,320	0,000	0,398	0,009	0,734	0,139	0,309	0,000
1029949	COG0745TK	gbs0963		Cellular processess	Signal transduction	Two-component response regulator	2,007	0,001	0,932	0,714	2,041	0,001	0,980	0,884
1029955	COG0642T	gbs0964		Cellular processess	Signal transduction	Two-component sensor histidine kinase (EC 2.7.3.-)	1,012	0,929	0,690	0,104	1,197	0,377	1,039	0,821
1029941	COG0582L	gbs0965	xerD	Cellular processess	DNA replication, recombination and repair	Integrase recombinase xerD	0,168	0,000	0,205	0,000	0,154	0,000	0,186	0,000
1029954	COG4166E	gbs0966		Metabolism and transport	Amino acids, peptides, aminosugars and amines	Oligopeptide-binding protein oppA	0,526	0,015	0,268	0,001	0,497	0,012	0,253	0,001
1029950	-	gbs0967	satE	Hypothetical		Hypothetical protein	0,484	0,003	0,599	0,012	0,530	0,005	0,654	0,022
1029951	-	gbs0968	satD	Hypothetical		Hypothetical protein	0,795	0,150	0,725	0,189	1,110	0,456	0,757	0,113
1029969	COG3505U	gbs0983		General function predicted only		ATPASE VIRD4 HOMOLOG	0,972	0,865	1,926	0,025	1,561	0,073	1,863	0,053

1029974	-	gbs0984		Hypothetical		Hypothetical protein	1,266	0,140	1,469	0,249	1,156	0,346	1,074	0,562
1029971	COG0629L	gbs0985		Hypothetical		Hypothetical protein	1,903	0,002	0,954	0,655	1,761	0,006	0,749	0,037
1029973	-	gbs0987		Hypothetical		Hypothetical protein	1,585	0,003	0,790	0,218	1,629	0,015	0,782	0,039
1029975	-	gbs0988		Cell Envelope	Other	M protein	1,570	0,000	0,974	0,799	1,599	0,001	1,097	0,408
1029982	-	gbs0989		Hypothetical		Hypothetical protein	1,335	0,030	0,349	0,000	1,205	0,206	0,671	0,035
1029968	-	gbs0990		Hypothetical		Hypothetical protein	1,937	0,000	1,149	0,364	2,371	0,001	1,182	0,085
1029976	COG0542O	gbs0991		Cellular processess	DNA replication, recombination and repair	DNA topoisomerase III (EC 5.99.1.2)	1,543	0,036	1,037	0,880	1,574	0,052	1,675	0,075
1029977	COG0550L	gbs0992		General function predicted only		LtrC-like protein	1,198	0,478	1,869	0,263	1,977	0,052	1,416	0,206
1029981	-	gbs0993		Hypothetical		Hypothetical protein	2,546	0,000	1,924	0,024	2,405	0,001	1,802	0,013
1029972	-	gbs0994		Hypothetical		Hypothetical cytosolic protein	0,545	0,008	0,837	0,464	0,581	0,005	0,499	0,003
1029986	-	gbs0995		Hypothetical		Hypothetical protein	1,066	0,364	0,774	0,201	1,162	0,103	0,937	0,744
1029980	-	gbs0996		Mobile and extrachromosomal elements		DNA-entry nuclease (EC 3.1.30.-)	1,245	0,282	1,404	0,341	1,089	0,476	1,108	0,740
1029985	-	gbs0997		Hypothetical		Hypothetical protein	0,865	0,381	0,837	0,310	0,716	0,081	0,956	0,800
1029984	-	gbs0998		Hypothetical		Hypothetical protein	0,796	0,053	0,596	0,008	0,918	0,371	0,872	0,328
1029991	-	gbs0999		Hypothetical		Hypothetical protein	0,644	0,021	0,291	0,000	0,316	0,001	0,240	0,000
1029989	-	gbs1002		Hypothetical		Hypothetical protein	1,354	0,173	1,969	0,028	1,457	0,229	2,176	0,009
1029997	-	gbs1003		Hypothetical		Hypothetical protein	2,540	0,000	2,061	0,002	2,357	0,000	2,035	0,011
1029987	-	gbs1004		Hypothetical		Hypothetical protein	0,696	0,067	1,867	0,125	1,276	0,259	1,107	0,599
1029990	-	gbs1005		Hypothetical		Hypothetical protein	1,681	0,130	2,377	0,074	1,640	0,054	1,657	0,077
1030009	COG0541U	gbs1017	ffh	Cellular processess	Posttranslational modification, protein turnover, chaperones	SIGNAL RECOGNITION PARTICLE, SUBUNIT FFH SRP54	0,391	0,000	0,509	0,000	0,409	0,000	0,628	0,001
1030013	COG2739S	gbs1018	ylxM	General function predicted only		Signal recognition particle associated protein	0,262	0,000	0,495	0,000	0,212	0,000	0,568	0,000
1030010	COG0642T	gbs1019	ciaH	Cellular processess	Signal transduction	Sensor protein ciaH (EC 2.7.3.-)	0,943	0,360	0,762	0,005	0,863	0,110	0,883	0,064
1030007	COG0745TK	gbs1020	ciaR	Cellular processess	Transcription	Transcriptional regulatory protein ciaR	1,636	0,000	0,772	0,017	1,486	0,000	0,855	0,114
1030012	COG0308E	gbs1021	pepN	Metabolism and transport	Amino acids, peptides, aminosugars and amines	Aminopeptidase N (EC 3.4.11.15)	0,501	0,000	0,623	0,000	0,469	0,000	0,644	0,000
1030011	COG0704P	gbs1022	phoU	Metabolism and transport	Inorganic ion transport and metabolism	Phosphate transport system protein phoU	0,286	0,000	0,458	0,000	0,244	0,000	0,467	0,000
1030014	COG1117P	gbs1023	pstB	Metabolism and transport	Inorganic ion transport and metabolism	Phosphate transport ATP-binding protein pstB	0,277	0,000	0,586	0,001	0,285	0,000	0,502	0,000
1030019	COG1117P	gbs1024	pstB2	Metabolism and transport	Inorganic ion transport and metabolism	Phosphate transport ATP-binding protein pstB	0,326	0,000	0,539	0,000	0,285	0,000	0,589	0,000
1030016	COG0581P	gbs1025	pstA	Metabolism and transport	Inorganic ion transport and metabolism	Phosphate transport system permease protein pstA	0,324	0,000	0,626	0,002	0,309	0,000	0,610	0,000
1030015	COG0573P	gbs1026	pstC	Metabolism and transport	Inorganic ion transport and metabolism	Phosphate transport system permease protein pstC	0,268	0,000	0,617	0,010	0,263	0,000	0,580	0,000
1030018	COG0226P	gbs1027	pstS	Metabolism and transport	Inorganic ion transport and metabolism	Phosphate-binding protein	0,282	0,000	0,724	0,007	0,338	0,000	0,600	0,000
1030020	COG0144J, C	gbs1028		Cellular processess	Translation, ribosomal structure and biogenesis	Putative 23S rRNA m(5)C methyltransferase (EC 2.1.1.-)	0,356	0,000	0,495	0,000	0,331	0,000	0,577	0,000
1030022	COG0483G	gbs1029		Metabolism and transport	Carbohydrates, organic alcohols, and acids	Myo-inositol-1(or 4)-monophosphatase (EC 3.1.3.25)	0,353	0,000	0,506	0,000	0,368	0,000	0,558	0,000
1030031	-	gbs1030		Hypothetical		Hypothetical cytosolic protein	0,334	0,000	0,423	0,000	0,289	0,000	0,503	0,000
1030028	COG1393P	gbs1031	spxA	Metabolism and transport	Inorganic ion transport and metabolism	Arsenate reductase family protein	0,368	0,000	0,525	0,000	0,309	0,000	0,538	0,000
1030029	COG0196H	gbs1032	mreA	Metabolism and transport	Cofactors, prosthetic groups, and carriers	Riboflavin kinase (EC 2.7.1.26) FMN adenylyltransferase (EC 2.7.7.2)	0,250	0,000	0,343	0,000	0,273	0,000	0,358	0,000
1030025	COG0130J	gbs1033	truB	Cellular processess	Translation, ribosomal structure and biogenesis	tRNA pseudouridine synthase B (EC 4.2.1.70)	0,284	0,000	0,331	0,000	0,199	0,000	0,341	0,000
1030026	-	gbs1034		Metabolism and transport	Central intermediary metabolism	Acetyltransferase (EC 2.3.1.-)	0,749	0,009	0,836	0,039	0,793	0,021	0,756	0,002
1030024	COG4487S	gbs1035		Hypothetical		Hypothetical protein	0,800	0,024	0,679	0,002	0,756	0,008	0,723	0,006
1030034	COG0732V	gbs1036		Mobile and extrachromosomal elements		Type I restriction-modification system specificity subunit	1,159	0,328	0,794	0,057	1,074	0,604	0,830	0,173
1030030	-	gbs1037		Cellular processess	Posttranslational modification, protein turnover, chaperones	CAAX amino terminal protease family	0,461	0,000	0,645	0,000	0,436	0,000	0,612	0,000
1030036	COG0577V	gbs1038		Transport and binding proteins	Unknown substrate	ABC transporter permease protein	1,025	0,901	2,012	0,000	1,144	0,226	2,432	0,000
1030033	COG1136V	gbs1039		Transport and binding proteins	Unknown substrate	ABC transporter ATP-binding protein	1,422	0,084	2,388	0,000	1,502	0,001	2,519	0,003
1030032	COG0550L, C	gbs1040		Cellular processess	DNA replication, recombination and repair	DNA topoisomerase I (EC 5.99.1.2)	0,576	0,001	0,735	0,138	0,630	0,002	0,827	0,079
1030038	COG0758LU	gbs1041	smf	Cellular processess	DNA replication, recombination and repair	DNA processing protein	2,219	0,001	3,905	0,000	2,447	0,001	2,952	0,000
1030041	COG4607P	gbs1042		Metabolism and transport	Inorganic ion transport and metabolism	Ferric anguibactin-binding protein	0,762	0,085	1,047	0,743	0,759	0,077	1,295	0,065
1030035	COG4604P	gbs1043		Metabolism and transport	Inorganic ion transport and metabolism	Ferric anguibactin transport ATP-binding protein	0,297	0,000	0,608	0,003	0,224	0,000	0,732	0,066
1030043	COG4605P	gbs1044		Metabolism and transport	Inorganic ion transport and metabolism	Ferric anguibactin transport system permease protein fatC	0,281	0,000	0,603	0,003	0,268	0,000	0,769	0,054
1030042	COG4606P	gbs1045		Metabolism and transport	Inorganic ion transport and metabolism	Ferric anguibactin transport system permease protein fatD	0,383	0,000	0,716	0,020	0,354	0,000	0,836	0,131
1030040	COG0110R	gbs1046		Metabolism and transport	Fatty acid and phospholipids	O-acetyltransferase (cell wall biosynthesis) (EC 2.3.1.-)	0,273	0,000	0,448	0,000	0,331	0,000	0,619	0,000
1030046	COG0164L	gbs1047	rnhB	Cellular processess	Transcription	Ribonuclease HII (EC 3.1.26.4)	0,294	0,000	0,557	0,000	0,319	0,000	0,622	0,000
1030047	COG1161R	gbs1048		General function predicted only		GTP-binding protein	0,475	0,000	0,366	0,000	0,351	0,000	0,746	0,002
1030048	COG1376S	gbs1049		Hypothetical		Hypothetical exported protein	1,081	0,630	1,494	0,084	1,360	0,170	1,930	0,020
1030045	COG1966T	gbs1050		Cellular processess	Signal transduction	Carbon starvation protein A	2,605	0,000	9,443	0,000	2,815	0,000	11,061	0,000
1030051	COG3279KT	gbs1051	lytS	Cellular processess	Transcription	Autolysin response regulator	0,469	0,000	0,825	0,143	0,588	0,004	0,930	0,616
1030052	COG3275T	gbs1052	lytR	Cellular processess	Signal transduction	Autolysin sensor kinase (EC 2.7.3.-)	0,909	0,551	1,190	0,365	0,763	0,244	1,281	0,063
1030044	-	gbs1053		Hypothetical		Hypothetical protein	0,463	0,000	0,400	0,002	0,246	0,000	0,547	0,000
1030050	-	gbs1054		Hypothetical		Hypothetical protein	0,248	0,000	0,505	0,001	0,219	0,000	0,567	0,003
1030053	-	gbs1055		Hypothetical		Hypothetical protein	0,354	0,000	0,562	0,023	0,258	0,000	0,596	0,002
1030057	-	gbs1056		Hypothetical		Hypothetical protein	0,439	0,000	0,471	0,001	0,328	0,000	0,484	0,000
1030054	-	gbs1057		Hypothetical		Hypothetical protein	0,299	0,000	0,138	0,000	0,212	0,000	0,219	0,000
1030067	-	gbs1061		Hypothetical		Hypothetical protein	0,843	0,196	1,248	0,257	1,044	0,792	0,667	0,124
1030056	COG4495S	gbs1062		Hypothetical		Hypothetical cytosolic protein	0,770	0,252	0,552	0,137	0,718	0,149	0,588	0,063
1030055	-	gbs1063		Hypothetical		Hypothetical protein	0,420	0,003	0,449	0,145	0,389	0,001	0,313	0,000
1030062	-	gbs1064		Hypothetical		Hypothetical protein	0,927	0,733	1,225	0,480	0,695	0,070	0,716	0,154
1030059	-	gbs1065		Hypothetical		Hypothetical protein	0,804	0,274	0,721	0,164	0,907	0,614	0,335	0,001
1030069	-	gbs1066		Hypothetical		Hypothetical protein	0,431	0,010	0,151	0,001	0,392	0,007	0,258	0,002
1030060	-	gbs1067		Hypothetical		Hypothetical protein	0,726	0,062	0,392	0,001	0,356	0,001	0,261	0,000
1030065	COG1674D	gbs1068		Cellular processess	Cell division	DNA segregation ATPase and related proteins (FtsK SpoIIIE family)	1,034	0,907	0,847	0,655	1,198	0,419	0,791	0,439
1030066	-	gbs1069		Hypothetical		Hypothetical protein	0,668	0,248	0,632	0,264	0,712	0,299	0,480	0,082
1030070	COG4499S	gbs1070		Hypothetical		Hypothetical membrane associated protein	0,530	0,089	0,235	0,015	0,445	0,054	0,160	0,010
1030073	-	gbs1071		Hypothetical		Hypothetical protein	0,570	0,110	0,168	0,009	0,485	0,070	0,166	0,009
1030071	-	gbs1072		Hypothetical		Hypothetical protein	0,818	0,492	0,440	0,057	0,870	0,614	0,427	0,052
1030077	COG1511S	gbs1073		Mobile and extrachromosomal elements		Phage infection protein	1,525	0,073	0,611	0,144	1,178	0,558	0,775	0,394
1030076	COG4842S	gbs1074		Hypothetical		Hypothetical protein	1,963	0,003	0,384	0,009	2,236	0,006	0,235	0,002
1030074	COG5153UI	gbs1075		Hypothetical		Hypothetical cytosolic protein	1,104	0,682	0,251	0,012	1,117	0,662	0,222	0,010
1030082	-	gbs1076		Hypothetical		Hypothetical secreted protein	1,356	0,271	0,280	0,024	1,213	0,496	0,143	0,012
1030084	COG0458EF	gbs1077	carB	Metabolism and transport	Purines, pyrimidines, nucleosides, and nucleotides	Carbamoyl-phosphate synthase large chain (EC 6.3.5.5)	2,261	0,007	0,776	0,443	2,403	0,001	0,554	0,143
1030081	COG0505EF	gbs1078	carA	Metabolism and transport	Purines, pyrimidines, nucleosides, and nucleotides	Carbamoyl-phosphate synthase small chain (EC 6.3.5.5)	2,467	0,002	0,510	0,128	2,443	0,007	0,400	0,063
1030083	COG0540F	gbs1079	pyrB	Metabolism and transport	Purines, pyrimidines, nucleosides, and nucleotides	Aspartate carbamoyltransferase (EC 2.1.3.2)	2,241	0,009	0,734	0,410	2,262	0,007	0,612	0,241
1030072	COG0044F	gbs1080	pyrC	Metabolism and transport	Purines, pyrimidines, nucleosides, and nucleotides	Dihydroorotase (EC 3.5.2.3)	2,328	0,001	0,652	0,127	2,466	0,000	0,528	0,048
1030087	COG0461F	gbs1081	pyrE	Metabolism and transport	Purines, pyrimidines, nucleosides, and nucleotides	Orotate phosphoribosyltransferase (EC 2.4.2.10)	2,394	0,000	0,697	0,214	2,357	0,001	0,537	0,064
1030080	COG0284F	gbs1082	pyrF	Metabolism and transport	Purines, pyrimidines, nucleosides, and nucleotides	Orotidine 5 -phosphate decarboxylase (EC 4.1.1.23)	2,352	0,000	0,700	0,219	2,238	0,001	0,413	0,033
1030085	COG2966S, C	gbs1083		Transport and binding proteins	Unknown substrate	Threonine Serine Exporter	0,552	0,001	0,740	0,028	0,473	0,000	0,747	0,018
1030093	COG0488R	gbs1084		Transport and binding proteins	Unknown substrate	ABC transporter ATP-binding protein	0,247	0,000	0,354	0,000	0,339	0,000	0,529	0,000
1030086	COG1328F	gbs1085	rnhB	Metabolism and transport	Purines, pyrimidines, nucleosides, and nucleotides	Anaerobic ribonucleoside-triphosphate reductase (EC 1.17.4.2)	4,295	0,000	1,788	0,001	4,445	0,000	1,378	0,202
1030088	COG0136E	gbs1086		Metabolism and transport	Amino acids, peptides, aminosugars and amines	Aspartate-semialdehyde dehydrogenase (EC 1.2.1.11)	0,477	0,000	0,440	0,000	0,421	0,000	0,520	0,000
1030097	-	gbs1087	fbsA	Hypothetical		LPXTG Hypothetical protein	2,593	0,000	0,923	0,451	2,402	0,000	0,837	0,053
1030090	COG1502I	gbs1088		Metabolism and transport	Fatty acid and phospholipids	Cardiolipin synthetase (EC 2.7.8.-)	0,933	0,597	0,532	0,005	0,785	0,105	0,424	0,002

1030094	COG2759F	gbs1089	fhs.1	Metabolism and transport	Purines, pyrimidines, nucleosides, and nucleotides	Formate--tetrahydrofolate ligase (EC 6.3.4.3)	0,782	0,062	0,408	0,001	0,817	0,102	0,424	0,001
1030091	COG0095H	gbs1090	lplA	Metabolism and transport	Cofactors, prosthetic groups, and carriers	Lipoate-protein ligase A (EC 6.3.2.-)	0,349	0,000	0,576	0,002	0,302	0,000	0,751	0,042
1030092	COG0846K	gbs1091		Cellular processess	Transcription	SIR2 family protein	0,235	0,000	0,496	0,002	0,275	0,000	0,657	0,013
1030095	-	gbs1092		Cellular processess	Posttranslational modification, protein turnover, chaperones	ATPase associated with chromosome architecture replication	0,345	0,000	0,572	0,002	0,364	0,000	0,803	0,134
1030102	COG0509E	gbs1093		Metabolism and transport	Amino acids, peptides, aminosugars and amines	Glycine cleavage system H protein	0,294	0,000	0,453	0,000	0,220	0,000	0,512	0,000
1030100	COG2141C	gbs1094		Metabolism and transport	Energy production and conversion	Luciferase-like monooxygenase (EC 1.14.-.-)	0,209	0,000	0,414	0,000	0,169	0,000	0,519	0,000
1030096	COG1902C	gbs1095		Metabolism and transport	Energy production and conversion	Probable NADH-dependent flavin oxidoreductase yqiG (EC 1.-.-.-)	0,395	0,000	0,750	0,079	0,383	0,000	0,839	0,073
1030101	COG0095H	gbs1096		Metabolism and transport	Cofactors, prosthetic groups, and carriers	Lipoate-protein ligase A (EC 6.3.2.-)	0,318	0,000	0,462	0,000	0,306	0,000	0,781	0,197
1030107	COG0452H	gbs1097	dpfB	Metabolism and transport	Cofactors, prosthetic groups, and carriers	Phosphopantothenate--cysteine ligase (EC 6.3.2.5)	0,384	0,000	0,495	0,001	0,306	0,000	0,562	0,005
1030103	COG0452H	gbs1098	dfp	Metabolism and transport	Cofactors, prosthetic groups, and carriers	Phosphopantothenoylcysteine decarboxylase (EC 4.1.1.36)	0,626	0,004	0,950	0,731	0,750	0,024	0,885	0,239
1030098	COG4684S	gbs1099		Hypothetical		Integral membrane protein	0,579	0,000	0,471	0,000	0,577	0,000	0,447	0,000
1030099	COG1109G	gbs1100	pgmA	Metabolism and transport	Carbohydrates, organic alcohols, and acids	Phosphoglucomutase (EC 5.4.2.2) Phosphomannomutase (EC 5.4.2.8)	1,619	0,000	2,002	0,000	1,676	0,000	2,212	0,000
1030108	-	gbs1101		Hypothetical		Hypothetical protein	0,327	0,000	0,511	0,003	0,335	0,000	0,505	0,001
1030115	COG1132V	gbs1102		Cellular processess	Toxin production and resistance	Multidrug protein lipid ABC transporter family, ATP-binding and permease protein	0,318	0,000	0,374	0,000	0,256	0,000	0,503	0,000
1030106	COG1132V	gbs1103		Cellular processess	Toxin production and resistance	Multidrug resistance ABC transporter ATP-binding and permease protein	0,333	0,000	0,408	0,000	0,325	0,000	0,503	0,000
1030105	-	gbs1104		Hypothetical		Antigen	0,373	0,000	0,448	0,000	0,295	0,000	0,395	0,000
1030119	-	gbs1105		Hypothetical		Hypothetical protein	0,299	0,000	0,226	0,000	0,267	0,000	0,311	0,000
1030114	COG0112E	gbs1106	glyA	Metabolism and transport	Amino acids, peptides, aminosugars and amines	Serine hydroxymethyltransferase (EC 2.1.2.1)	0,454	0,000	0,449	0,000	0,516	0,000	0,425	0,000
1030116	COG0009J	gbs1107		Cellular processess	Translation, ribosomal structure and biogenesis	Sua5 YciO YrdC YwlC family protein	0,447	0,000	0,518	0,000	0,455	0,000	0,444	0,000
1030111	COG2890J	gbs1108	hemK	Cellular processess	Translation, ribosomal structure and biogenesis	Peptide release factor-glutamine N5-methyltransferase (EC 2.1.1.-)	0,325	0,000	0,360	0,000	0,275	0,000	0,349	0,000
1030109	COG0216J	gbs1109	prfA	Cellular processess	Translation, ribosomal structure and biogenesis	Bacterial Peptide Chain Release Factor 1 (RF-1)	0,429	0,000	0,274	0,000	0,414	0,000	0,295	0,000
1030113	COG1435F	gbs1110	tdk2	Metabolism and transport	Purines, pyrimidines, nucleosides, and nucleotides	Thymidine kinase (EC 2.7.1.21)	0,599	0,000	0,542	0,000	0,508	0,000	0,508	0,000
1030117	COG1942R	gbs1111		Metabolism and transport	Energy production and conversion	4-oxalocrotonate tautomerase (EC 5.3.2.-)	4,338	0,001	1,801	0,019	4,325	0,000	0,653	0,013
1030118	-	gbs1112	apbE	Metabolism and transport	Cofactors, prosthetic groups, and carriers	Iron-sulfur cluster assembly repair protein ApbE	4,129	0,000	4,979	0,000	3,227	0,000	8,005	0,000
1030120	COG0431R	gbs1113		Metabolism and transport	Central intermediary metabolism	NADPH-dependent FMN reductase family protein	3,875	0,000	5,429	0,000	3,255	0,000	8,268	0,000
1030123	COG0431R, (	gbs1114		Metabolism and transport	Central intermediary metabolism	Oxidoreductase (EC 1.1.1.-)	2,117	0,007	4,742	0,000	1,705	0,068	8,817	0,000
1030125	COG2116P	gbs1115		Metabolism and transport	Inorganic ion transport and metabolism	Formate nitrite transporter family protein	1,745	0,008	5,209	0,000	1,807	0,003	6,993	0,000
1030122	COG2233F	gbs1116		Metabolism and transport	Purines, pyrimidines, nucleosides, and nucleotides	Xanthine permease	0,327	0,000	0,275	0,000	0,336	0,000	0,274	0,000
1030124	COG0503F	gbs1117	xpt	Metabolism and transport	Purines, pyrimidines, nucleosides, and nucleotides	Xanthine phosphoribosyltransferase (EC 2.4.2.-)	0,344	0,000	0,315	0,000	0,346	0,000	0,289	0,000
1030130	-	gbs1118		Hypothetical		Hypothetical protein	0,321	0,017	0,683	0,255	0,444	0,048	0,473	0,047
1030126	-	gbs1119		Hypothetical		hypothetical phage protein	1,914	0,119	2,340	0,043	2,378	0,113	4,136	0,003
1030127	COG0863L, C	gbs1120		Cellular processess	DNA replication, recombination and repair	Chromosome partitioning protein parB Adenine-specific methyltransferase (EC 2.1.	4,973	0,000	8,396	0,001	5,947	0,000	7,527	0,000
1030132	COG3843U	gbs1121		General function predicted only		Relaxase	5,334	0,047	7,387	0,045	9,017	0,098	14,850	0,007
1030128	-	gbs1122		Mobile and extrachromosomal elements		Mobilisation protein	3,684	0,063	7,450	0,030	1,696	0,158	4,942	0,094
1030133	-	gbs1123		Hypothetical		Hypothetical protein	4,062	0,067	6,429	0,030	3,863	0,048	4,539	0,011
1030129	-	gbs1124		Hypothetical		Hypothetical protein	5,267	0,003	8,910	0,043	2,494	0,096	6,761	0,020
1030135	-	gbs1125		Hypothetical		Hypothetical protein	2,686	0,001	7,955	0,027	3,156	0,001	5,307	0,002
1030134	-	gbs1126		General function predicted only		LtrC-like protein	3,084	0,021	3,903	0,000	3,684	0,006	3,157	0,006
1030137	-	gbs1127		Hypothetical		Hypothetical protein	4,554	0,002	10,729	0,029	7,743	0,053	12,433	0,003
1030131	COG3505U	gbs1128		Mobile and extrachromosomal elements		TraG TraD family	1,167	0,662	2,632	0,003	1,538	0,223	2,453	0,064
1030141	-	gbs1129		Hypothetical		Hypothetical protein	4,684	0,008	7,457	0,002	3,388	0,049	6,189	0,015
1030138	-	gbs1130		Hypothetical		Hypothetical protein	3,663	0,015	7,184	0,005	3,034	0,005	3,329	0,000
1030143	-	gbs1131		Hypothetical		Hypothetical protein	2,731	0,051	2,532	0,085	2,884	0,064	1,586	0,152
1030142	-	gbs1132		Hypothetical		Hypothetical protein	1,371	0,185	7,475	0,036	1,222	0,369	5,269	0,007
1030145	COG3942R	gbs1133		Hypothetical		Hypothetical protein	6,997	0,000	10,948	0,004	8,035	0,000	11,999	0,001
1030140	-	gbs1134		Hypothetical		Hypothetical protein	1,058	0,715	0,603	0,080	0,933	0,708	0,640	0,232
1030146	COG3451U	gbs1135		Hypothetical		TRSE PROTEIN	5,583	0,058	12,255	0,095	6,009	0,002	12,406	0,018
1030150	-	gbs1136		Hypothetical		Hypothetical protein	4,449	0,011	7,580	0,013	5,209	0,000	5,430	0,002
1030147	-	gbs1137		Hypothetical		Hypothetical protein	2,120	0,149	3,582	0,060	2,740	0,045	2,977	0,068
1030139	-	gbs1138		Hypothetical		Hypothetical protein	2,332	0,113	2,691	0,016	2,222	0,033	2,522	0,070
1030151	-	gbs1139		Hypothetical		Hypothetical protein	2,835	0,034	4,065	0,002	2,729	0,066	2,545	0,000
1030152	-	gbs1140		Hypothetical		Hypothetical protein	7,526	0,019	7,326	0,031	8,692	0,068	6,963	0,002
1030149	-	gbs1141		Hypothetical		Hypothetical protein	4,294	0,001	3,187	0,003	4,741	0,005	2,438	0,016
1030144	-	gbs1142		Hypothetical		Hypothetical protein	3,509	0,001	7,160	0,000	3,785	0,000	5,042	0,002
1030157	-	gbs1143	epf	Hypothetical		LPXTG Cell surface protein	1,101	0,751	1,008	0,977	1,477	0,311	1,609	0,254
1030153	-	gbs1144		Hypothetical		LPXTG Cell surface protein	2,453	0,013	3,152	0,048	2,389	0,017	1,920	0,105
1030159	-	gbs1145		Cell Envelope	Other	LPXTG M protein	1,522	0,129	3,420	0,074	2,492	0,016	3,410	0,011
1030148	-	gbs1146		Hypothetical		Hypothetical protein	2,613	0,009	2,334	0,023	2,158	0,039	3,256	0,010
1030163	COG1396K	gbs1147		Cellular processess	Transcription	Transcriptional regulator, Cro CI family	3,130	0,011	1,460	0,139	2,866	0,036	2,065	0,182
1030155	-	gbs1148		Hypothetical		Hypothetical protein	2,229	0,163	8,289	0,190	2,531	0,129	2,080	0,045
1030154	-	gbs1149		General function predicted only		Replication initiator protein	2,505	0,083	6,711	0,022	5,214	0,007	4,458	0,033
1030156	-	gbs1150		Hypothetical		Hypothetical protein	2,074	0,046	9,844	0,118	4,422	0,102	3,389	0,022
1030166	-	gbs1151		Hypothetical		Hypothetical protein	4,396	0,006	7,654	0,010	13,614	0,163	5,811	0,001
1030158	-	gbs1152		Hypothetical		Hypothetical protein	3,877	0,050	8,200	0,086	5,252	0,090	5,740	0,056
1030160	-	gbs1153		Hypothetical		Hypothetical protein	2,825	0,111	7,534	0,071	2,242	0,016	4,572	0,043
1030167	COG0516F	gbs1154	guaC	Metabolism and transport	Purines, pyrimidines, nucleosides, and nucleotides	GMP reductase (EC 1.7.1.7)	1,074	0,492	0,399	0,000	1,013	0,886	0,242	0,000
1030161	-	gbs1155		Cellular processess	Toxin production and resistance	Multidrug resistance protein B	0,606	0,017	1,558	0,053	0,562	0,005	1,802	0,001
1030165	-	gbs1156		Cellular processess	Toxin production and resistance	Na+ driven multidrug efflux pump	0,309	0,002	0,308	0,002	0,301	0,002	0,248	0,001
1030162	COG3158P	gbs1157		Metabolism and transport	Inorganic ion transport and metabolism	Kup system potassium uptake protein	0,436	0,000	0,774	0,273	0,346	0,000	0,571	0,003
1030169	COG4221R	gbs1158		Metabolism and transport	Central intermediary metabolism	Short chain dehydrogenase	1,493	0,003	1,711	0,000	1,630	0,003	1,478	0,005
1030164	COG0280C	gbs1159	pta	Metabolism and transport	Energy production and conversion	Phosphate acetyltransferase (EC 2.3.1.8)	0,487	0,001	1,134	0,092	0,507	0,000	1,125	0,037
1030171	COG0564J	gbs1160		Cellular processess	Translation, ribosomal structure and biogenesis	Ribosomal large subunit pseudouridine synthase D (EC 4.2.1.70)	0,267	0,000	0,447	0,000	0,214	0,000	0,767	0,013
1030172	COG0061G	gbs1161	ppnK	Metabolism and transport	Carbohydrates, organic alcohols, and acids	ATP-NAD kinase (EC 2.7.1.23)	0,231	0,000	0,352	0,000	0,189	0,000	0,476	0,000
1030177	COG2357S	gbs1162		Metabolism and transport	Purines, pyrimidines, nucleosides, and nucleotides	GTP pyrophosphokinase homolog	0,396	0,000	0,556	0,000	0,364	0,000	0,618	0,001
1030173	COG4116S	gbs1163		General function predicted only		Organic phosphate binding CYTH family protein	1,713	0,003	0,791	0,131	1,605	0,004	0,894	0,185
1030168	COG0462FE	gbs1164		Metabolism and transport	Purines, pyrimidines, nucleosides, and nucleotides	Ribose-phosphate pyrophosphokinase (EC 2.7.6.1)	0,595	0,000	0,908	0,141	0,618	0,001	0,971	0,641
1030175	COG1104E	gbs1165	nifS2	Metabolism and transport	Amino acids, peptides, aminosugars and amines	Cysteine desulfurase (EC 2.8.1.7) Selenocysteine lyase (EC 4.4.1.16)	0,299	0,000	0,401	0,000	0,267	0,000	0,495	0,000
1030178	-	gbs1166		Hypothetical		Hypothetical cytosolic protein	0,313	0,000	0,347	0,000	0,318	0,000	0,447	0,000
1030176	COG2344R	gbs1167		General function predicted only		AT-rich DNA-binding protein	0,780	0,062	1,555	0,001	0,744	0,017	1,992	0,000
1030180	COG2003L	gbs1168	radC	Cellular processess	DNA replication, recombination and repair	DNA repair protein radC	17,935	0,009	8,356	0,002	18,897	0,052	7,447	0,003
1030179	COG0628R	gbs1169		Hypothetical		Hypothetical membrane spanning protein	0,816	0,026	0,868	0,173	0,799	0,022	1,023	0,752
1030181	-	gbs1170		Metabolism and transport	Carbohydrates, organic alcohols, and acids	6-phospho-beta-glucosidase (EC 3.2.1.86)	0,960	0,748	1,849	0,039	0,820	0,315	1,434	0,110
1030185	COG2755E	gbs1171		Metabolism and transport	Fatty acid and phospholipids	Lipase Acylhydrolase family protein	0,446	0,000	0,782	0,057	0,427	0,000	0,754	0,022
1030182	COG0561R	gbs1172		Metabolism and transport	Central intermediary metabolism	Hydrolase (HAD superfamily)	0,456	0,001	0,917	0,504	0,379	0,000	0,898	0,393
1030192	-	gbs1173		Cellular processess	Transcription	Transcriptional regulator, AraC family	0,193	0,002	0,198	0,003	0,244	0,004	0,742	0,201
1030186	COG0038P	gbs1174		Metabolism and transport	Inorganic ion transport and metabolism	Chloride channel protein	0,725	0,001	1,166	0,216	0,895	0,413	1,518	0,000
1030184	COG0687E	gbs1175		Metabolism and transport	Amino acids, peptides, aminosugars and amines	Spermidine putrescine-binding protein	0,386	0,000	0,932	0,585	0,419	0,000	0,967	0,769
1030190	COG1177E	gbs1176	potC	Metabolism and transport	Amino acids, peptides, aminosugars and amines	Spermidine putrescine transport system permease protein potC	0,281	0,000	0,690	0,010	0,304	0,000	0,723	0,013

1030194	COG1176E	gbs1177	potB	Metabolism and transport	Amino acids, peptides, aminosugars and amines	Spermidine putrescine transport system permease protein potB	0,223	0,000	0,659	0,025	0,242	0,000	0,742	0,065
1030187	COG3842E	gbs1178	potA	Metabolism and transport	Amino acids, peptides, aminosugars and amines	Spermidine putrescine transport ATP-binding protein potA	0,333	0,000	0,684	0,068	0,233	0,000	0,680	0,034
1030189	COG0812M	gbs1179	murB	Cell Envelope	Biosynthesis and degradation of murein sacculus and peptidoglycar	UDP-N-acetylenolpyruvoylglucosamine reductase (EC 1.1.1.158)	0,314	0,000	0,733	0,008	0,344	0,000	0,901	0,167
1030195	COG0801H	gbs1180	folK	Metabolism and transport	Cofactors, prosthetic groups, and carriers	2-amino-4-hydroxy-6-hydroxymethyldihydropteridine pyrophosphokinase (EC 2.7.6.3	0,327	0,000	0,434	0,001	0,277	0,000	0,511	0,002
1030197	COG1539H	gbs1181	folQ	Metabolism and transport	Cofactors, prosthetic groups, and carriers	Dihydroneopterin aldolase (EC 4.1.2.25)	0,181	0,000	0,289	0,000	0,170	0,000	0,266	0,000
1030193	COG0294H	gbs1182	folP	Metabolism and transport	Cofactors, prosthetic groups, and carriers	Dihydropteroate synthase (EC 2.5.1.15)	0,232	0,000	0,154	0,000	0,220	0,000	0,262	0,000
1030198	COG0302H	gbs1183	folE	Metabolism and transport	Cofactors, prosthetic groups, and carriers	GTP cyclohydrolase I (EC 3.5.4.16)	0,282	0,000	0,472	0,000	0,300	0,000	0,481	0,000
1030196	COG0285H	gbs1184	folC.1	Metabolism and transport	Cofactors, prosthetic groups, and carriers	Folylpolyglutamate synthase (EC 6.3.2.17) Dihydrofolate synthase (EC 6.3.2.12)	0,288	0,000	0,390	0,000	0,292	0,000	0,419	0,000
1030203	COG2962R	gbs1185		General function predicted only		Chloramphenicol-sensitive protein rarD	0,184	0,000	0,356	0,000	0,202	0,000	0,406	0,000
1030201	COG0083E	gbs1186		Metabolism and transport	Amino acids, peptides, aminosugars and amines	Homoserine kinase (EC 2.7.1.39)	0,383	0,000	0,536	0,000	0,433	0,000	0,641	0,001
1030200	COG0460E	gbs1187		Metabolism and transport	Amino acids, peptides, aminosugars and amines	Homoserine dehydrogenase (EC 1.1.1.3)	0,591	0,003	0,666	0,023	0,642	0,006	0,835	0,137
1030199	-	gbs1188		Cell Envelope	Biosynthesis and degradation of surface polysaccharides and lipopo	Polysaccharide deacetylase	0,631	0,000	0,727	0,036	0,606	0,000	0,758	0,007
1030205	COG1292M	gbs1191		Metabolism and transport	Amino acids, peptides, aminosugars and amines	Glycine betaine transporter	0,841	0,016	0,321	0,000	0,598	0,000	0,303	0,000
1030211	COG1012C	gbs1192	gabD	Metabolism and transport	Energy production and conversion	Succinate-semialdehyde dehydrogenase [NADP+] (EC 1.2.1.16)	1,342	0,119	2,353	0,000	1,399	0,081	2,444	0,000
1030206	-	gbs1193		Hypothetical		Hypothetical membrane spanning protein	0,745	0,030	0,489	0,002	0,623	0,005	0,731	0,037
1030213	-	gbs1194		Hypothetical		Hypothetical protein	1,721	0,003	0,820	0,072	1,664	0,005	0,795	0,064
1030214	-	gbs1195	ska	General function predicted only		streptokinase	4,384	0,027	2,072	0,019	4,202	0,030	2,660	0,000
1030207	COG1476K	gbs1196		Cellular processess	Transcription	Transcriptional regulator, Cro CI family	0,397	0,004	0,280	0,000	0,268	0,000	0,486	0,002
1030212	-	gbs1197		Hypothetical		Hypothetical protein	0,724	0,016	0,571	0,018	0,556	0,002	0,849	0,187
1030210	COG2077O	gbs1198	tpx	Metabolism and transport	Energy production and conversion	Thioredoxin peroxidase (EC 1.11.1.15)	1,629	0,000	1,326	0,001	1,720	0,000	1,147	0,075
1030217	COG4912L	gbs1199		Hypothetical		Hypothetical protein	0,434	0,002	0,612	0,037	0,300	0,000	0,689	0,042
1030218	COG0561R	gbs1200		Metabolism and transport	Central intermediary metabolism	Hydrolase (HAD superfamily)	0,341	0,001	0,689	0,029	0,321	0,001	0,560	0,005
1030216	COG0490P, C	gbs1201		Metabolism and transport	Inorganic ion transport and metabolism	NAD-dependent K+ or Na+ uptake system component Transcriptional regulator, Gr	0,393	0,001	0,792	0,104	0,374	0,001	0,871	0,277
1030219	-	gbs1202		Cellular processess	Posttranslational modification, protein turnover, chaperones	General stress protein, Gls24 family	1,396	0,033	2,824	0,000	1,602	0,006	2,367	0,000
1030221	-	gbs1203		Hypothetical		Hypothetical protein	1,628	0,005	3,464	0,001	1,957	0,010	2,803	0,000
1030215	-	gbs1204		Cellular processess	Posttranslational modification, protein turnover, chaperones	General stress protein, Gls24 family	1,434	0,030	2,791	0,000	1,569	0,014	2,161	0,000
1030223	-	gbs1205		General function predicted only		Small integral membrane protein	1,555	0,012	2,717	0,001	1,628	0,024	2,027	0,001
1030222	-	gbs1206		Hypothetical		Hypothetical protein	1,409	0,057	2,460	0,001	1,421	0,072	1,808	0,006
1030224	COG2261S	gbs1207		Hypothetical		Integral membrane protein	1,791	0,005	2,636	0,000	1,727	0,003	1,879	0,007
1030220	COG2261S	gbs1208		Hypothetical		Integral membrane protein	2,546	0,000	3,797	0,000	2,561	0,000	2,723	0,001
1030227	COG0210L	gbs1209	pcrA	Cellular processess	DNA replication, recombination and repair	DNA helicase II (EC 3.6.1.-)	0,449	0,001	0,685	0,015	0,393	0,000	0,693	0,028
1030226	COG2050Q	gbs1210		Metabolism and transport	Secondary metabolites	Thioesterase superfamily protein	1,073	0,378	0,978	0,861	0,932	0,440	0,776	0,033
1030225	COG2233F	gbs1211	pyrP	Metabolism and transport	Purines, pyrimidines, nucleosides, and nucleotides	Uracil permease	1,013	0,955	0,515	0,055	1,021	0,927	0,404	0,023
1030230	COG1115E	gbs1212		Metabolism and transport	Amino acids, peptides, aminosugars and amines	Na(+)-linked D-alanine glycine permease	0,321	0,004	0,290	0,003	0,311	0,004	0,236	0,002
1030229	COG0053P	gbs1213		Metabolism and transport	Inorganic ion transport and metabolism	Cobalt-zinc-cadmium resistance protein czcD	0,368	0,001	0,268	0,000	0,302	0,001	0,224	0,000
1030228	COG1380R	gbs1214		Cell Envelope	Biosynthesis and degradation of murein sacculus and peptidoglycar	Murein hydrolase exporter	0,481	0,002	0,896	0,605	0,644	0,032	0,308	0,000
1030236	-	gbs1215		Cell Envelope	Biosynthesis and degradation of murein sacculus and peptidoglycar	Murein hydrolase export regulator	0,684	0,028	0,839	0,632	0,652	0,007	0,721	0,043
1030234	-	gbs1216		Mobile and extrachromosomal elements		Phage protein	1,300	0,056	0,575	0,000	1,228	0,152	0,548	0,000
1030237	-	gbs1217		Hypothetical		Hypothetical protein	2,223	0,213	10,993	0,090	11,856	0,198	3,023	0,099
1030233	-	gbs1218		Hypothetical		Hypothetical protein	0,455	0,000	0,718	0,014	0,430	0,000	0,760	0,036
1030242	-	gbs1219		Mobile and extrachromosomal elements		Phage protein	5,538	0,035	20,777	0,088	8,216	0,004	5,346	0,000
1030241	-	gbs1220		Hypothetical		Hypothetical protein	0,284	0,000	0,621	0,084	0,334	0,000	0,482	0,000
1030240	COG2932K	gbs1221		Cellular processess	Transcription	Phage transcriptional repressor	1,503	0,002	1,127	0,350	1,632	0,002	1,410	0,056
1030239	-	gbs1222		Mobile and extrachromosomal elements		Phage protein	0,831	0,203	0,705	0,030	0,889	0,317	1,062	0,627
1030238	COG4823V	gbs1223		Hypothetical		Hypothetical protein	0,609	0,017	1,247	0,148	0,551	0,005	1,161	0,375
1030246	COG0582L	gbs1224	int.3	Mobile and extrachromosomal elements		DNA integration recombination inversion protein	1,283	0,164	2,678	0,002	1,380	0,054	2,652	0,000
1030244	COG0539J	gbs1225		Cellular processess	Translation, ribosomal structure and biogenesis	SSU ribosomal protein S1P	0,725	0,003	0,902	0,271	0,718	0,001	0,841	0,083
1030250	-	gbs1226		Hypothetical		Hypothetical cytosolic protein	0,545	0,001	0,274	0,000	0,497	0,000	0,283	0,000
1030248	COG0115EH	gbs1227	bcaT	Metabolism and transport	Amino acids, peptides, aminosugars and amines	Branched-chain amino acid aminotransferase (EC 2.6.1.42)	0,858	0,189	0,449	0,000	0,825	0,126	0,421	0,000
1030251	COG0188L	gbs1228	parC	Cellular processess	DNA replication, recombination and repair	Topoisomerase IV subunit A (EC 5.99.1.-)	0,361	0,000	0,310	0,000	0,342	0,000	0,502	0,001
1030247	COG0187L	gbs1229	parE	Cellular processess	DNA replication, recombination and repair	Topoisomerase IV subunit B (EC 5.99.1.-)	0,438	0,001	0,465	0,004	0,328	0,000	0,529	0,003
1030254	COG0344S	gbs1230		Hypothetical		Hypothetical membrane spanning protein	0,677	0,008	0,883	0,242	0,618	0,001	0,878	0,226
1030253	COG0692L	gbs1231	ung	Cellular processess	DNA replication, recombination and repair	Uracil-DNA glycosylase (EC 3.2.2.-)	1,104	0,261	1,346	0,045	1,151	0,120	1,204	0,179
1030255	-	gbs1232		Hypothetical		Hypothetical protein	0,465	0,000	0,862	0,025	0,495	0,000	0,845	0,007
1030252	COG1083M, (	gbs1233	neuA	General function predicted only		Acylneuraminate cytidylyltransferase (EC 2.7.7.43)	0,186	0,000	0,436	0,003	0,163	0,000	0,542	0,009
1030259	COG0110R	gbs1234	neuD	Cell Envelope	Biosynthesis and degradation of surface polysaccharides and lipopo	Sialic acid biosynthesis protein NeuD	0,253	0,000	0,613	0,011	0,256	0,000	0,654	0,020
1030260	COG0381M	gbs1235	neuC	Cell Envelope	Biosynthesis and degradation of surface polysaccharides and lipopo	UDP-N-acetylglucosamine 2-epimerase (N-acetylmannosamine-forming) (EC 5.1.3.-	0,179	0,000	0,515	0,002	0,156	0,000	0,570	0,004
1030261	COG2089M	gbs1236	neuB	Cell Envelope	Biosynthesis and degradation of surface polysaccharides and lipopo	N-acetylneuraminate synthase (EC 2.5.1.56)	0,526	0,004	0,715	0,048	0,527	0,004	0,843	0,232
1030258	COG2244R	gbs1237	cpsM	Cell Envelope	Biosynthesis and degradation of surface polysaccharides and lipopo	Capsular polysaccharide protein CpsK	0,102	0,000	0,361	0,000	0,127	0,000	0,431	0,001
1030256	-	gbs1237.1	cpsL	Cell Envelope	Biosynthesis and degradation of surface polysaccharides and lipopo	beta-D-Galp alpha-2,3-sialyltransferase (EC 2.4.99.-)	0,144	0,000	0,445	0,001	0,135	0,000	0,538	0,003
1030262	COG0463M	gbs1238	CpslaJ	Cell Envelope	Biosynthesis and degradation of surface polysaccharides and lipopo	beta-D-GlcNAc beta-1,4-galactosyltransferase (EC 2.4.1.-)	0,116	0,000	0,383	0,000	0,127	0,000	0,491	0,001
1030263	COG0463M	gbs1239	cpsJ	Cell Envelope	Biosynthesis and degradation of surface polysaccharides and lipopo	beta-D-Galp beta-1,3-N-acetylglucosaminyltransferase (EC 2.4.1.-)	0,121	0,000	0,386	0,000	0,147	0,000	0,443	0,000
1030265	-	gbs1240	cpsI	General function predicted only		Secreted polysaccharide polymerase	0,093	0,000	0,435	0,000	0,120	0,000	0,404	0,000
1030267	-	gbs1241	cpsG	Cell Envelope	Biosynthesis and degradation of surface polysaccharides and lipopo	beta-D-Glcp beta-1,4-galactosyltransferase (EC 2.4.1.-)	0,166	0,000	0,504	0,000	0,167	0,000	0,587	0,001
1030269	COG0707M	gbs1242	cpsF	Metabolism and transport	Carbohydrates, organic alcohols, and acids	Beta-1,4-galactosyltransferase accessory protein	0,161	0,000	0,425	0,000	0,128	0,000	0,527	0,000
1030266	COG2148M	gbs1243	cpsE	Cell Envelope	Biosynthesis and degradation of surface polysaccharides and lipopo	Undecaprenyl-phosphate beta-glucosephosphotransferase (EC 2.7.8.-)	0,208	0,000	0,567	0,000	0,210	0,000	0,591	0,000
1030264	COG0489D	gbs1244	cpsD	Cell Envelope	Biosynthesis and degradation of surface polysaccharides and lipopo	Tyrosine-protein kinase (capsular polysaccharide biosynthesis)	0,297	0,000	0,479	0,000	0,273	0,000	0,617	0,000
1030272	COG3944M	gbs1245	cpsC	Cellular processess	Cell division	Chain length regulator (capsular polysaccharide biosynthesis)	0,225	0,000	0,369	0,000	0,194	0,000	0,465	0,000
1030270	COG4464GM	gbs1246	cpsB	Cell Envelope	Biosynthesis and degradation of surface polysaccharides and lipopo	Phosphotyrosine-protein phosphatase (capsular polysaccharide biosynthesis) (EC 3	0,132	0,000	0,347	0,000	0,142	0,000	0,413	0,000
1030271	COG1316K	gbs1247	cpsA	Cellular processess	Transcription	Transcriptional activator CpsA	0,277	0,000	0,481	0,000	0,307	0,000	0,464	0,000
1030274	COG0583K	gbs1248	cpsY	Cellular processess	Transcription	Transcriptional regulators, LysR family	0,545	0,000	0,814	0,032	0,538	0,000	0,798	0,011
1030275	-	gbs1250	cpsX	Cellular processess	Signal transduction	Histidine protein kinase	0,518	0,000	1,533	0,000	0,507	0,000	1,924	0,000
1030276	COG0813F	gbs1251	deoD2	Metabolism and transport	Purines, pyrimidines, nucleosides, and nucleotides	Purine nucleoside phosphorylase (EC 2.4.2.1)	0,521	0,000	1,340	0,037	0,459	0,000	1,785	0,000
1030273	COG0038P	gbs1252		Metabolism and transport	Inorganic ion transport and metabolism	Chloride channel protein	0,400	0,000	1,265	0,024	0,370	0,000	1,684	0,000
1030279	COG0005F	gbs1253	punA	Metabolism and transport	Purines, pyrimidines, nucleosides, and nucleotides	Purine nucleoside phosphorylase (EC 2.4.2.1)	0,511	0,000	1,272	0,017	0,510	0,000	1,619	0,000
1030278	COG1393P	gbs1254	arsC	Metabolism and transport	Inorganic ion transport and metabolism	Arsenate reductase (EC 1.20.4.1)	0,601	0,000	1,491	0,003	0,590	0,000	1,726	0,000
1030280	COG1015G	gbs1255		Metabolism and transport	Carbohydrates, organic alcohols, and acids	Phosphopentomutase (EC 5.4.2.7)	0,629	0,000	1,312	0,002	0,640	0,000	1,748	0,000
1030277	COG0120G	gbs1256	rpiA	Metabolism and transport	Carbohydrates, organic alcohols, and acids	Ribose 5-phosphate isomerase (EC 5.3.1.6)	0,729	0,001	1,303	0,012	0,693	0,000	1,428	0,000
1030282	COG5549O	gbs1257		Cellular processess	Posttranslational modification, protein turnover, chaperones	Endopeptidase, M10 family (EC 3.4.24.-)	0,435	0,000	0,768	0,011	0,453	0,000	0,885	0,295
1030281	COG0627R	gbs1258	estA	General function predicted only		Acetyl esterase (EC 3.1.1.-)	0,524	0,000	0,671	0,007	0,499	0,000	0,819	0,018
1030283	COG0595R	gbs1259		General function predicted only		Zn-dependent hydrolase (EC 3.-.-.-)	0,533	0,000	0,894	0,057	0,543	0,000	1,085	0,221
1030287	COG1101R	gbs1260		Transport and binding proteins	Unknown substrate	ABC transporter ATP-binding protein	0,837	0,323	0,973	0,848	0,821	0,248	0,967	0,809
1030288	COG4120R	gbs1261		Transport and binding proteins	Unknown substrate	ABC transporter permease protein	0,552	0,011	0,508	0,008	0,432	0,003	0,301	0,001
1030290	COG2984R	gbs1262		Transport and binding proteins	Unknown substrate	ABC transporter substrate-binding protein	0,940	0,662	1,302	0,080	1,122	0,486	1,332	0,049
1030285	COG1293K	gbs1263	fbp	Cell Envelope	Other	Fibronectin-binding protein Fibrinogen-binding protein	0,673	0,000	0,397	0,000	0,667	0,000	0,485	0,000
1030284	COG3527Q	gbs1264		Metabolism and transport	Secondary metabolites	Alpha-acetolactate decarboxylase (EC 4.1.1.5)	0,443	0,000	0,211	0,000	0,402	0,000	0,225	0,000
1030291	COG0028EH	gbs1265		Metabolism and transport	Amino acids, peptides, aminosugars and amines	Acetolactate synthase (EC 2.2.1.6)	0,817	0,008	0,438	0,000	0,755	0,000	0,351	0,000
1030293	-	gbs1266		Hypothetical		Tetratricopeptide repeat family protein	0,357	0,000	0,479	0,000	0,338	0,000	0,396	0,000

1030289	COG0628R	gbs1267		Hypothetical		Hypothetical membrane spanning protein	0,435	0,000	0,558	0,002	0,452	0,000	0,661	0,004
1030295	COG0494LR	gbs1268		Cellular processess	DNA replication, recombination and repair	Phosphohydrolase (MutT nudix family protein)	0,636	0,001	0,731	0,012	0,599	0,004	0,656	0,002
1030296	COG0494LR	gbs1269		General function predicted only		7,8-dihydro-8-oxoguanine-triphosphatase (EC 3.6.1.-)	1,126	0,231	0,545	0,000	0,951	0,574	0,530	0,000
1030294	-	gbs1270		General function predicted only		Hyaluronate lyase precursor (EC 4.2.2.1)	1,127	0,111	1,286	0,176	1,251	0,177	1,445	0,060
1030292	COG1088M	gbs1271	cpsFQ	Metabolism and transport	Carbohydrates, organic alcohols, and acids	dTDP-glucose 4,6-dehydratase (EC 4.2.1.46)	0,663	0,000	0,716	0,001	0,671	0,000	0,879	0,054
1030299	COG1898M	gbs1272	rmlC	Metabolism and transport	Carbohydrates, organic alcohols, and acids	dTDP-4-dehydrorhamnose 3,5-epimerase (EC 5.1.3.13)	0,666	0,000	0,726	0,000	0,650	0,000	0,964	0,491
1030298	COG1209M	gbs1273	rmlA	Metabolism and transport	Carbohydrates, organic alcohols, and acids	Glucose-1-phosphate thymidylyltransferase (EC 2.7.7.24)	1,037	0,578	0,779	0,013	0,993	0,913	0,971	0,578
1030300	COG0665E	gbs1274		Metabolism and transport	Amino acids, peptides, aminosugars and amines	Glycine D-amino acid oxidases family	0,382	0,000	0,604	0,005	0,449	0,000	0,648	0,000
1030297	COG0327S	gbs1275		General function predicted only		NIF3-related protein	0,709	0,007	0,450	0,000	0,595	0,000	0,453	0,000
1030303	COG2384R	gbs1276		Hypothetical		Hypothetical cytosolic protein	0,457	0,000	0,477	0,000	0,386	0,000	0,463	0,000
1030302	COG3935L	gbs1277	dnaD	Cellular processess	DNA replication, recombination and repair	DNA replication protein dnaD	0,098	0,000	0,255	0,000	0,068	0,000	0,158	0,000
1030304	COG0503F	gbs1278	apt	Metabolism and transport	Purines, pyrimidines, nucleosides, and nucleotides	Adenine phosphoribosyltransferase (EC 2.4.2.7)	0,424	0,000	1,036	0,501	0,458	0,000	0,940	0,399
1030301	-	gbs1279		Cell Envelope	Other	M protein	1,111	0,181	1,100	0,323	1,384	0,002	1,472	0,001
1030307	COG0608L	gbs1280	recJ	Cellular processess	DNA replication, recombination and repair	Single-stranded-DNA-specific exonuclease recJ (EC 3.1.-.-)	0,406	0,000	0,844	0,227	0,293	0,000	0,913	0,453
1030308	COG0300R	gbs1281		Metabolism and transport	Central intermediary metabolism	Short chain dehydrogenase	0,443	0,000	0,885	0,190	0,492	0,000	1,018	0,813
1030306	COG1234R	gbs1282	elaC	Cellular processess	Transcription	Ribonuclease Z (EC 3.1.26.11)	0,446	0,000	0,896	0,318	0,456	0,000	0,981	0,795
1030305	-	gbs1283		Metabolism and transport	Carbohydrates, organic alcohols, and acids	galactose 1-phosphate uridylyltransferase	0,317	0,000	0,715	0,070	0,330	0,000	0,711	0,002
1030311	COG2262R	gbs1284	hflX	General function predicted only		GTP-binding protein hflX	0,661	0,001	1,207	0,254	0,855	0,131	1,194	0,039
1030309	COG0324J	gbs1285	miaA	Cellular processess	Translation, ribosomal structure and biogenesis	tRNA delta(2)-isopentenylpyrophosphate transferase (EC 2.5.1.8)	1,870	0,001	2,049	0,005	1,931	0,032	1,805	0,004
1030310	-	gbs1286		Hypothetical		Hypothetical protein	3,482	0,000	1,453	0,018	3,015	0,000	0,875	0,310
1030314	COG1275P	gbs1287	exfA	Hypothetical		Hypothetical membrane-spanning protein	0,848	0,455	1,800	0,008	0,832	0,397	1,875	0,012
1030315	COG1523G	gbs1288		Metabolism and transport	Carbohydrates, organic alcohols, and acids	LPXTG Pullulanase (EC 3.2.1.41)	3,638	0,049	15,691	0,001	3,582	0,073	21,908	0,000
1030313	COG1387ER	gbs1289		Metabolism and transport	Amino acids, peptides, aminosugars and amines	Histidinol-phosphatase (EC 3.1.3.15)	0,567	0,001	0,337	0,000	0,616	0,000	0,506	0,001
1030312	COG1573L	gbs1290		Cellular processess	DNA replication, recombination and repair	Uracil DNA glycosylase superfamily protein	0,614	0,003	0,336	0,000	0,566	0,000	0,487	0,000
1030317	COG0624E	gbs1291		Metabolism and transport	Amino acids, peptides, aminosugars and amines	Xaa-His dipeptidase (EC 3.4.13.3)	1,359	0,001	0,876	0,088	1,303	0,003	0,999	0,988
1030316	COG0778C	gbs1292		Metabolism and transport	Energy production and conversion	NAD(P)H-dependent quinone reductase (EC 1.-.-.-)	1,235	0,060	0,510	0,000	1,128	0,123	0,662	0,000
1030319	COG0584C, (	gbs1293		Metabolism and transport	Energy production and conversion	glycerophosphoryl diester phosphodiesterase	0,872	0,203	1,570	0,017	1,141	0,409	1,404	0,002
1030322	COG0322L	gbs1294	uvrC	Cellular processess	DNA replication, recombination and repair	Excinuclease ABC subunit C	0,342	0,000	0,503	0,002	0,288	0,000	0,587	0,005
1030320	COG1636S	gbs1295		Hypothetical		Hypothetical cytosolic protein	0,510	0,000	0,583	0,006	0,454	0,000	0,640	0,031
1030321	COG0534V	gbs1296		Cellular processess	Toxin production and resistance	Na+ driven multidrug efflux pump	0,650	0,056	0,441	0,000	0,680	0,060	0,545	0,006
1030318	COG2764S	gbs1297		Metabolism and transport	Energy production and conversion	PhnB protein	0,828	0,005	0,629	0,000	0,798	0,008	0,505	0,000
1030327	-	gbs1298		Hypothetical		hypothetical protein	0,405	0,000	0,529	0,000	0,377	0,000	0,437	0,000
1030325	-	gbs1299		Mobile and extrachromosomal elements		PUTATIVE HOST CELL SURFACE-EXPOSED LIPOPROTEIN	0,499	0,000	0,339	0,000	0,440	0,000	0,354	0,000
1030323	COG2801L	gbs1301		Mobile and extrachromosomal elements		Transposase	1,495	0,154	1,355	0,136	0,964	0,900	2,181	0,006
1030324	-	gbs1302		Mobile and extrachromosomal elements		Transposase	1,624	0,355	4,928	0,049	2,416	0,259	3,537	0,066
1030329	-	gbs1306		Mobile and extrachromosomal elements		Streptococcal histidine triad protein	0,311	0,005	0,776	0,331	0,459	0,014	1,347	0,397
1030333	COG0803P	gbs1307	lmb	Metabolism and transport	Inorganic ion transport and metabolism	Laminin-binding surface protein	1,783	0,025	1,547	0,139	1,559	0,007	1,996	0,007
1030336	COG1404O	gbs1308	scpB	Cellular processess	Posttranslational modification, protein turnover, chaperones	LPXTN C5A peptidase precursor (EC 3.4.21.-)	0,767	0,045	1,073	0,590	0,795	0,049	1,257	0,137
1030332	COG2176L	gbs1312		Hypothetical		Hypothetical protein	0,194	0,000	0,377	0,002	0,215	0,000	0,310	0,000
1030342	-	gbs1313		Hypothetical		Hypothetical protein	0,290	0,000	0,621	0,004	0,372	0,000	0,424	0,000
1030339	COG0582L	gbs1314		Mobile and extrachromosomal elements		DNA integration recombination inversion protein	0,832	0,295	0,782	0,014	0,783	0,060	1,214	0,180
1030338	-	gbs1315		Hypothetical		Hypothetical protein	1,057	0,623	1,414	0,004	1,177	0,120	1,757	0,001
1030351	-	gbs1316		Mobile and extrachromosomal elements		Replication protein	4,635	0,007	6,721	0,006	8,104	0,009	8,393	0,001
1030348	-	gbs1318		Hypothetical		Hypothetical protein	2,254	0,288	11,686	0,206	3,671	0,155	2,599	0,030
1030343	COG1674D	gbs1320		Cellular processess	Cell division	FtsK SpoIIIE family	2,346	0,157	2,817	0,116	2,529	0,143	5,429	0,044
1030357	-	gbs1321		Hypothetical		Hypothetical protein	4,322	0,002	10,924	0,054	4,032	0,011	5,876	0,003
1030354	-	gbs1322		Hypothetical		Hypothetical protein	0,532	0,102	0,850	0,538	0,659	0,202	1,557	0,212
1030361	-	gbs1323		Hypothetical		Hypothetical protein	0,725	0,375	1,275	0,434	0,656	0,133	1,394	0,422
1030353	-	gbs1324	hsdM	Mobile and extrachromosomal elements		TYPE II RESTRICTION-MODIFICATION SYSTEM MODIFICATION SUBUNIT	0,831	0,481	1,853	0,147	1,003	0,993	1,392	0,181
1030364	COG0582L	gbs1325		Mobile and extrachromosomal elements		DNA integration recombination inversion protein	1,003	0,981	1,537	0,003	0,915	0,476	2,143	0,000
1030363	-	gbs1326		Hypothetical		Hypothetical protein	2,039	0,000	2,431	0,048	1,769	0,013	2,779	0,000
1030360	COG1396K	gbs1327		Cellular processess	Transcription	Transcriptional regulator, Cro CI family	6,154	0,084	11,191	0,008	3,735	0,014	10,392	0,066
1030368	COG2017G	gbs1328		Metabolism and transport	Carbohydrates, organic alcohols, and acids	Aldose 1-epimerase family protein	5,535	0,004	8,976	0,000	5,446	0,003	7,610	0,001
1030367	-	gbs1329	lacG	Metabolism and transport	Carbohydrates, organic alcohols, and acids	6-phospho-beta-galactosidase (EC 3.2.1.85)	1,579	0,221	5,579	0,011	2,494	0,045	4,136	0,004
1030369	COG1455G	gbs1330	lacE	Metabolism and transport	Carbohydrates, organic alcohols, and acids	PTS system, lactose-specific IIBC component (EC 2.7.1.69)	3,259	0,000	6,175	0,000	2,988	0,001	4,999	0,000
1030366	-	gbs1331	lacF	Metabolism and transport	Carbohydrates, organic alcohols, and acids	PTS system, lactose-specific IIA component (EC 2.7.1.69)	4,299	0,000	10,066	0,003	5,661	0,003	7,907	0,002
1030373	COG3711K	gbs1332		Cellular processess	Transcription	Transcription antiterminator	1,857	0,112	6,167	0,047	3,352	0,033	2,738	0,057
1030371	COG3684G	gbs1333	lacD.2	Metabolism and transport	Carbohydrates, organic alcohols, and acids	Tagatose-bisphosphate aldolase (EC 4.1.2.40)	3,118	0,000	5,198	0,000	3,650	0,001	6,544	0,000
1030372	COG1105G	gbs1334	lacC.2	Metabolism and transport	Carbohydrates, organic alcohols, and acids	Tagatose-6-phosphate kinase (EC 2.7.1.144)	3,963	0,025	7,163	0,011	3,153	0,000	8,167	0,013
1030370	COG0698G	gbs1335	lacB.1	Metabolism and transport	Carbohydrates, organic alcohols, and acids	Galactose-6-phosphate isomerase lacB subunit (EC 5.3.1.26)	2,959	0,002	4,636	0,010	3,327	0,036	4,600	0,006
1030376	COG0698G	gbs1336	lacA.2	Metabolism and transport	Carbohydrates, organic alcohols, and acids	Galactose-6-phosphate isomerase lacA subunit (EC 5.3.1.26)	3,181	0,000	3,606	0,004	3,343	0,000	5,086	0,000
1030375	COG1349KG	gbs1337		Cellular processess	Transcription	Lactose phosphotransferase system repressor	0,845	0,144	1,193	0,061	0,898	0,201	1,327	0,006
1030378	COG3843U	gbs1338	srtI	General function predicted only		Relaxase	2,667	0,004	2,603	0,039	2,202	0,062	3,139	0,010
1030374	-	gbs1339		Mobile and extrachromosomal elements		Mobilisation protein	1,221	0,662	7,121	0,016	1,434	0,484	2,893	0,103
1030381	-	gbs1340		Hypothetical		Hypothetical protein	3,222	0,008	6,993	0,003	3,707	0,006	4,095	0,000
1030379	COG0210L	gbs1341		Cellular processess	DNA replication, recombination and repair	DNA helicase II (EC 3.6.1.-)	0,676	0,000	1,095	0,414	0,710	0,015	1,309	0,001
1030380	COG3593L	gbs1342		Hypothetical		Hypothetical protein	0,896	0,352	1,082	0,503	1,032	0,766	1,214	0,129
1030377	-	gbs1343		Cellular processess	Toxin production and resistance	Hypothetical protein	2,260	0,004	1,653	0,035	2,485	0,004	1,573	0,020
1030384	COG1396K	gbs1344		Cellular processess	Transcription	Transcriptional regulator, Cro CI family	4,279	0,000	2,069	0,004	4,554	0,000	2,136	0,023
1030383	-	gbs1345		Hypothetical		Hypothetical protein	5,430	0,009	6,454	0,005	6,074	0,019	7,615	0,019
1030382	-	gbs1346		Hypothetical		Hypothetical protein	0,784	0,368	1,344	0,467	0,666	0,063	1,128	0,651
1030388	-	gbs1347		Hypothetical		Hypothetical protein	1,035	0,928	3,175	0,153	1,468	0,452	2,859	0,145
1030387	-	gbs1348		General function predicted only		LtrC-like protein	2,052	0,008	3,307	0,006	2,304	0,011	4,399	0,001
1030386	-	gbs1349		Hypothetical		Hypothetical protein	1,035	0,831	2,246	0,012	1,498	0,017	2,204	0,005
1030391	-	gbs1350		Hypothetical		Hypothetical protein	1,316	0,028	1,421	0,176	1,085	0,640	1,552	0,019
1030390	-	gbs1351		Hypothetical		Hypothetical protein	1,918	0,002	0,796	0,424	1,939	0,000	0,875	0,636
1030392	COG0553KL,	gbs1352		General function predicted only		Superfamily II DNA and RNA helicase (SNF2 family)	3,941	0,009	7,691	0,003	5,893	0,004	6,342	0,001
1030389	-	gbs1353		General function predicted only		Superfamily II DNA and RNA helicase (SNF2 family)	3,675	0,002	10,397	0,000	4,135	0,004	7,762	0,003
1030397	-	gbs1354		Hypothetical		Hypothetical protein	1,795	0,285	10,376	0,076	3,131	0,095	4,302	0,022
1030395	-	gbs1355		Hypothetical		Hypothetical protein	4,634	0,002	7,625	0,002	4,885	0,001	5,789	0,003
1030396	-	gbs1356		Cellular processess	Toxin production and resistance	LPXTG Cell surface protein	3,321	0,001	7,227	0,002	5,661	0,000	5,588	0,015
1030393	COG5340K	gbs1357		Mobile and extrachromosomal elements		Abortive infection protein AbiEi	0,577	0,004	0,847	0,307	0,540	0,002	0,945	0,706
1030401	COG2253S	gbs1358		Mobile and extrachromosomal elements		Abortive infection protein AbiEii	0,691	0,014	1,010	0,940	0,687	0,016	1,088	0,558
1030400	COG3942R	gbs1359		Hypothetical		Hypothetical membrane associated protein	4,907	0,025	12,499	0,050	5,226	0,004	10,771	0,004
1030398	COG3451U	gbs1360		Hypothetical		TRSE PROTEIN	3,365	0,041	2,466	0,063	2,190	0,063	1,790	0,058
1030399	-	gbs1361		Hypothetical		Hypothetical protein	2,686	0,035	6,751	0,009	3,729	0,011	5,738	0,006
1030405	-	gbs1362		Hypothetical		Hypothetical protein	3,326	0,008	8,086	0,001	3,532	0,003	5,301	0,001
1030403	-	gbs1363		Hypothetical		Hypothetical protein	4,348	0,013	6,965	0,000	3,958	0,003	4,879	0,005

1030406	COG3505U	gbs1364		Mobile and extrachromosomal elements		TraG TraD family	4,165	0,050	10,613	0,028	3,316	0,029	10,075	0,002
1030402	-	gbs1365		Hypothetical		Hypothetical protein	5,132	0,001	6,980	0,005	7,211	0,021	9,549	0,010
1030411	-	gbs1366		Cellular processess	Posttranslational modification, protein turnover, chaperones	CAAX amino terminal protease family	2,477	0,008	7,499	0,051	2,998	0,051	4,938	0,051
1030407	-	gbs1367		Hypothetical		Hypothetical protein	1,815	0,165	7,672	0,118	7,043	0,010	2,350	0,010
1030409	COG1393P	gbs1368		Metabolism and transport	Inorganic ion transport and metabolism	Arsenate reductase (EC 1.20.4.1)	10,625	0,111	47,968	0,117	28,553	0,050	14,797	0,049
1030408	-	gbs1369		Hypothetical		Hypothetical protein	4,450	0,014	6,133	0,010	5,009	0,004	4,133	0,013
1030414	COG0270L	gbs1370		Cellular processess	DNA replication, recombination and repair	DNA-cytosine methyltransferase (EC 2.1.1.37)	7,453	0,005	9,823	0,027	5,193	0,001	11,592	0,003
1030413	-	gbs1371		Cellular processess	DNA replication, recombination and repair	DNA-cytosine methyltransferase (EC 2.1.1.37)	3,026	0,106	7,083	0,013	3,408	0,027	5,279	0,055
1030415	-	gbs1372		Hypothetical		IFN-response binding factor 1	2,307	0,001	4,017	0,002	2,676	0,021	4,796	0,004
1030412	-	gbs1373		Hypothetical		Hypothetical protein	3,261	0,013	8,850	0,019	3,816	0,015	7,506	0,005
1030417	COG0222J	gbs1374	rplL	Cellular processess	Translation, ribosomal structure and biogenesis	LSU ribosomal protein L12P (L7 L12)	1,749	0,003	1,236	0,113	1,841	0,000	1,085	0,471
1030420	COG0244J	gbs1375	rplJ	Cellular processess	Translation, ribosomal structure and biogenesis	LSU ribosomal protein L10P	1,602	0,000	0,814	0,096	1,593	0,000	0,717	0,014
1030418	COG0542O	gbs1376	clpL	Cellular processess	Posttranslational modification, protein turnover, chaperones	ATP-dependent endopeptidase clp ATP-binding subunit clpL	0,711	0,135	2,287	0,000	1,111	0,613	10,378	0,000
1030421	COG2040E	gbs1377	mmuM	Metabolism and transport	Amino acids, peptides, aminosugars and amines	Homocysteine S-methyltransferase (EC 2.1.1.10)	0,575	0,043	0,822	0,451	0,757	0,227	1,411	0,084
1030419	COG0833E	gbs1378		Metabolism and transport	Amino acids, peptides, aminosugars and amines	S-methylmethionine permease	0,969	0,803	1,291	0,119	0,903	0,611	1,490	0,043
1030425	-	gbs1379		Hypothetical		Hypothetical protein	1,488	0,060	2,716	0,000	1,753	0,020	2,128	0,002
1030422	-	gbs1380		Hypothetical		Hypothetical protein	1,687	0,010	2,858	0,037	2,102	0,037	2,267	0,003
1030423	COG1309K	gbs1381		Cellular processess	Transcription	Transcriptional regulator, TetR family	0,813	0,086	0,823	0,332	0,725	0,071	0,744	0,037
1030428	COG0218R	gbs1382		General function predicted only		GTP-binding protein YihA	0,233	0,000	0,389	0,000	0,215	0,000	0,386	0,000
1030429	COG1219O	gbs1383	clpX	Cellular processess	Posttranslational modification, protein turnover, chaperones	ATP-dependent endopeptidase clp ATP-binding subunit clpX	0,401	0,000	0,497	0,000	0,316	0,000	0,535	0,000
1030427	COG0262H	gbs1384	dfrA	Metabolism and transport	Cofactors, prosthetic groups, and carriers	Dihydrofolate reductase (EC 1.5.1.3)	0,395	0,000	0,338	0,000	0,361	0,000	0,364	0,000
1030432	-	gbs1385	ThyA	Metabolism and transport	Purines, pyrimidines, nucleosides, and nucleotides	Thymidylate synthase (EC 2.1.1.45)	0,525	0,000	0,434	0,000	0,470	0,000	0,476	0,000
1030426	COG3425I	gbs1386	mvaS.1	Metabolism and transport	Fatty acid and phospholipids	Hydroxymethylglutaryl-CoA synthase (EC 2.3.3.10)	0,362	0,000	0,360	0,000	0,248	0,000	0,547	0,001
1030434	COG1257I	gbs1387		Metabolism and transport	Fatty acid and phospholipids	3-hydroxy-3-methylglutaryl-coenzyme A reductase (EC 1.1.1.34) 3-hydroxy-3-meth	0,299	0,000	0,274	0,000	0,240	0,000	0,419	0,000
1030433	-	gbs1388		Hypothetical		Hypothetical cytosolic protein	0,465	0,000	0,314	0,000	0,461	0,000	0,275	0,000
1030436	COG1272R	gbs1389	hemIII	General function predicted only		Conserved membrane protein (hemolysin III homolog)	1,028	0,763	0,558	0,001	0,982	0,878	0,437	0,000
1030431	COG1597IR	gbs1390		Hypothetical		Hypothetical protein	0,573	0,000	0,485	0,000	0,526	0,000	0,548	0,000
1030437	COG0435O	gbs1391		Metabolism and transport	Central intermediary metabolism	Glutathione S-transferase family protein	2,113	0,000	1,236	0,050	2,011	0,000	1,237	0,036
1030435	-	gbs1392		Hypothetical		Hypothetical protein	0,248	0,000	0,489	0,000	0,246	0,000	0,487	0,000
1030443	COG1304C	gbs1393		Metabolism and transport	Energy production and conversion	Isopentenyl-diphosphate delta-isomerase (EC 5.3.3.2)	0,382	0,000	0,604	0,002	0,400	0,000	0,828	0,081
1030440	COG1577I	gbs1394	mvaK2	Metabolism and transport	Fatty acid and phospholipids	Phosphomevalonate kinase (EC 2.7.4.2)	0,214	0,000	0,359	0,000	0,163	0,000	0,459	0,000
1030441	COG3407I	gbs1395	mvaD	Metabolism and transport	Fatty acid and phospholipids	Diphosphomevalonate decarboxylase (EC 4.1.1.33)	0,318	0,000	0,418	0,001	0,194	0,000	0,649	0,017
1030439	COG1577I	gbs1396	mvaK1	Metabolism and transport	Fatty acid and phospholipids	Mevalonate kinase (EC 2.7.1.36)	0,560	0,000	0,430	0,000	0,353	0,000	0,642	0,001
1030438	COG0642T	gbs1397		Cellular processess	Signal transduction	Two component system histidine kinase (EC 2.7.3.-)	0,511	0,000	0,862	0,317	0,512	0,000	0,850	0,211
1030446	COG0745TK	gbs1398		Cellular processess	Signal transduction	Two-component response regulator	0,762	0,007	0,907	0,414	0,680	0,003	0,607	0,001
1030447	COG2357S	gbs1399		Metabolism and transport	Purines, pyrimidines, nucleosides, and nucleotides	GTP pyrophosphokinase (EC 2.7.6.5)	0,558	0,000	0,581	0,000	0,498	0,000	0,640	0,000
1030445	COG1132V	gbs1400		Cellular processess	Toxin production and resistance	Multidrug protein lipid ABC transporter family, ATP-binding and permease protein	0,717	0,301	1,317	0,313	0,662	0,214	1,508	0,132
1030448	COG1132V	gbs1401		Cellular processess	Toxin production and resistance	Multidrug resistance ABC transporter ATP-binding and permease protein	0,840	0,308	1,313	0,278	0,807	0,194	1,521	0,013
1030444	COG1846K	gbs1402		Cellular processess	Transcription	Transcriptional regulator, MarR family	0,764	0,372	1,879	0,236	0,448	0,022	0,968	0,920
1030452	COG0737F	gbs1403		Metabolism and transport	Purines, pyrimidines, nucleosides, and nucleotides	LPXTN 5 -nucleotidase (EC 3.1.3.5)	0,693	0,110	0,640	0,067	0,777	0,236	0,694	0,102
1030451	-	gbs1404	fms	Cellular processess	Posttranslational modification, protein turnover, chaperones	Peptide deformylase (EC 3.5.1.88)	0,464	0,000	0,310	0,000	0,428	0,000	0,384	0,000
1030449	COG0334E	gbs1405		Metabolism and transport	Amino acids, peptides, aminosugars and amines	NADP-specific glutamate dehydrogenase (EC 1.4.1.4)	0,790	0,021	0,654	0,002	0,871	0,147	0,574	0,000
1030456	COG3247S	gbs1406		Hypothetical		Hypothetical membrane spanning protein	1,082	0,462	0,470	0,001	0,885	0,352	0,394	0,000
1030453	COG1132V	gbs1407		Cellular processess	Toxin production and resistance	Multidrug protein lipid ABC transporter family, ATP-binding and permease protein	0,342	0,000	0,614	0,029	0,390	0,000	1,112	0,209
1030454	COG1132V	gbs1408		Cellular processess	Toxin production and resistance	Multidrug resistance ABC transporter ATP-binding and permease protein	0,450	0,000	0,772	0,138	0,415	0,000	0,961	0,537
1030461	-	gbs1409		Metabolism and transport	Central intermediary metabolism	Acetyltransferase yjgM (EC 2.3.1.-)	0,258	0,000	0,457	0,000	0,241	0,000	0,613	0,002
1030463	COG0488R	gbs1410		Transport and binding proteins	Unknown substrate	ABC transporter ATP-binding protein uup	0,322	0,000	0,501	0,000	0,343	0,000	0,716	0,003
1030458	COG0617J	gbs1411	papS	Cellular processess	Translation, ribosomal structure and biogenesis	tRNA nucleotidyltransferase (EC 2.7.7.25)	0,177	0,000	0,327	0,000	0,223	0,000	0,480	0,000
1030459	COG1307S	gbs1412		Metabolism and transport	Fatty acid and phospholipids	Fatty acid-binding protein, DegV family	0,341	0,000	0,650	0,000	0,354	0,000	0,787	0,002
1030462	-	gbs1413		Hypothetical		Hypothetical cytosolic protein	0,388	0,000	0,648	0,000	0,338	0,000	0,723	0,002
1030457	-	gbs1414		Hypothetical		Hypothetical protein	0,483	0,000	0,515	0,003	0,494	0,001	0,622	0,012
1030468	COG4331S	gbs1415		General function predicted only		CPRD14 PROTEIN	1,476	0,031	0,976	0,908	1,261	0,164	1,141	0,438
1030467	COG1299G, (	gbs1416	fruA	Metabolism and transport	Carbohydrates, organic alcohols, and acids	PTS system, fructose-specific IIABC component (EC 2.7.1.69)	0,638	0,177	0,558	0,083	0,550	0,075	0,672	0,168
1030466	COG1105G	gbs1417	fruB	Metabolism and transport	Carbohydrates, organic alcohols, and acids	1-phosphofructokinase (EC 2.7.1.56)	0,394	0,022	0,448	0,031	0,449	0,028	0,430	0,024
1030465	COG1349KG	gbs1418	fruR	Cellular processess	Transcription	Fructose repressor	0,233	0,023	0,268	0,027	0,204	0,019	0,359	0,044
1030472	COG2348V	gbs1419		Cellular processess	Toxin production and resistance	Factor essential for expression of methicillin resistance	0,610	0,002	1,293	0,080	0,787	0,156	1,792	0,000
1030471	-	gbs1420		General function predicted only		LPXTG Choline-binding protein	3,398	0,001	6,051	0,000	4,492	0,001	9,493	0,000
1030469	COG1893H	gbs1421	apbA	Metabolism and transport	Cofactors, prosthetic groups, and carriers	Ketopantoate reductase PanE ApbA family protein	0,436	0,000	0,729	0,014	0,394	0,000	0,934	0,525
1030475	COG3641R	gbs1422		Hypothetical		Hypothetical membrane spanning protein	0,257	0,000	0,254	0,000	0,228	0,000	0,552	0,002
1030473	COG0492O	gbs1423	trxB	Metabolism and transport	Energy production and conversion	Thioredoxin reductase (EC 1.8.1.9)	0,238	0,000	0,388	0,000	0,121	0,000	0,574	0,013
1030474	-	gbs1424	trmD	Cellular processess	Translation, ribosomal structure and biogenesis	tRNA (Guanine-N(1)-)-methyltransferase (EC 2.1.1.31)	0,335	0,000	0,528	0,016	0,344	0,000	0,986	0,935
1030477	COG0806J	gbs1425	rimM	Cellular processess	Translation, ribosomal structure and biogenesis	16S rRNA processing protein rimM	0,595	0,001	1,301	0,095	0,645	0,001	1,275	0,013
1030481	-	gbs1426		Cellular processess	Transcription	Transcriptional regulator	0,540	0,000	0,665	0,001	0,579	0,000	0,739	0,006
1030480	COG1837R	gbs1427		General function predicted only		RNA binding protein	0,424	0,000	0,477	0,000	0,341	0,000	0,616	0,001
1030482	-	gbs1428	rpsP	Cellular processess	Translation, ribosomal structure and biogenesis	SSU ribosomal protein S16P	0,389	0,000	0,472	0,000	0,346	0,000	0,618	0,000
1030476	COG0577V	gbs1429		Transport and binding proteins	Unknown substrate	ABC transporter permease protein	0,374	0,000	0,251	0,000	0,412	0,000	0,370	0,000
1030484	COG1136V	gbs1430		Transport and binding proteins	Unknown substrate	ABC transporter ATP-binding protein	0,490	0,000	0,389	0,000	0,403	0,000	0,282	0,000
1030486	COG0845M	gbs1431		Cell Envelope	Other	HlyD family secretion protein	0,756	0,015	0,594	0,003	0,692	0,017	0,570	0,000
1030483	COG0458EF	gbs1432		Metabolism and transport	Purines, pyrimidines, nucleosides, and nucleotides	Carbamoyl-phosphate synthase large chain (EC 6.3.5.5)	0,618	0,001	0,751	0,036	0,646	0,001	1,094	0,171
1030490	COG0505EF	gbs1433		Metabolism and transport	Purines, pyrimidines, nucleosides, and nucleotides	Carbamoyl-phosphate synthase small chain (EC 6.3.5.5)	1,003	0,966	1,015	0,913	1,040	0,712	1,353	0,002
1030487	COG2065F	gbs1434	pyrR	Metabolism and transport	Purines, pyrimidines, nucleosides, and nucleotides	Uracil phosphoribosyltransferase (EC 2.4.2.9) Pyrimidine operon regulatory protein	1,242	0,084	1,327	0,012	1,342	0,014	1,362	0,014
1030488	COG0564J	gbs1435		Cellular processess	Translation, ribosomal structure and biogenesis	Ribosomal large subunit pseudouridine synthase D (EC 4.2.1.70)	1,105	0,268	1,303	0,003	1,088	0,417	1,492	0,000
1030494	COG0597MU	gbs1436	lsp	Cellular processess	Posttranslational modification, protein turnover, chaperones	Lipoprotein signal peptidase (EC 3.4.23.36)	0,502	0,000	0,550	0,000	0,428	0,000	0,442	0,000
1030495	COG0583K	gbs1437		Cellular processess	Transcription	Transcriptional regulators, LysR family	0,874	0,271	0,609	0,001	0,714	0,022	0,500	0,001
1030493	-	gbs1438	rpmA	Cellular processess	Translation, ribosomal structure and biogenesis	LSU ribosomal protein L27P	1,257	0,010	0,782	0,021	1,225	0,032	0,787	0,013
1030499	COG2868J	gbs1439		General function predicted only		hypothetical ribosome-associated protein	1,199	0,041	0,687	0,000	1,074	0,307	0,694	0,000
1030492	-	gbs1440	rpl21	Cellular processess	Translation, ribosomal structure and biogenesis	LSU ribosomal protein L21P	1,271	0,029	0,699	0,000	1,196	0,030	0,751	0,002
1030504	COG2843M	gbs1441	capA	Cell Envelope	Biosynthesis and degradation of surface polysaccharides and lipopo	Capsule biosynthesis protein capA	1,728	0,000	1,323	0,011	1,688	0,004	1,130	0,277
1030505	COG0301H	gbs1442	thiI	Metabolism and transport	Cofactors, prosthetic groups, and carriers	Thiamine biosynthesis protein thiI	0,369	0,000	0,776	0,011	0,411	0,000	0,894	0,190
1030502	COG1104E	gbs1443	nifS1	Metabolism and transport	Amino acids, peptides, aminosugars and amines	Cysteine desulfurase (EC 2.8.1.7) Selenocysteine lyase (EC 4.4.1.16)	0,101	0,000	0,293	0,000	0,072	0,000	0,430	0,000
1030501	-	gbs1444		Hypothetical		Hypothetical protein	0,294	0,000	0,224	0,000	0,253	0,000	0,234	0,000
1030500	COG1249C	gbs1445	gor	Metabolism and transport	Energy production and conversion	Glutathione reductase (EC 1.8.1.7)	0,568	0,000	0,458	0,000	0,546	0,000	0,349	0,000
1030506	COG3679S	gbs1446		Hypothetical		Hypothetical cytosolic protein	0,593	0,000	0,542	0,000	0,507	0,000	0,537	0,000
1030507	-	gbs1447	aroF	Metabolism and transport	Amino acids, peptides, aminosugars and amines	Chorismate synthase (EC 4.2.3.5)	0,243	0,000	0,578	0,006	0,358	0,000	0,913	0,619
1030510	COG0337E	gbs1448	aroB	Metabolism and transport	Amino acids, peptides, aminosugars and amines	3-dehydroquinate synthase (EC 4.2.3.4)	0,162	0,000	0,389	0,002	0,349	0,000	0,335	0,000
1030512	-	gbs1449	aroD	Metabolism and transport	Amino acids, peptides, aminosugars and amines	3-dehydroquinate dehydratase (EC 4.2.1.10)	0,288	0,000	0,518	0,000	0,265	0,000	0,614	0,001
1030508	COG1092R	gbs1450		Metabolism and transport	Central intermediary metabolism	Methyltransferase (EC 2.1.1.-)	0,297	0,000	0,547	0,000	0,270	0,000	0,614	0,000
1030514	COG1368M	gbs1451		Metabolism and transport	Central intermediary metabolism	Sulfatase family protein	0,266	0,000	0,317	0,000	0,245	0,000	0,397	0,000

1030516	-	gbs1452	rplT	Cellular processess	Translation, ribosomal structure and biogenesis	LSU ribosomal protein L20P	0,906	0,215	0,536	0,002	0,853	0,027	0,432	0,000
1030515	COG0291J	gbs1453	rpmI	Cellular processess	Translation, ribosomal structure and biogenesis	LSU ribosomal protein L35P	1,238	0,001	0,603	0,000	1,025	0,582	0,560	0,000
1030513	COG0290J	gbs1454	infC	Cellular processess	Translation, ribosomal structure and biogenesis	Bacterial Protein Translation Initiation Factor 3 (IF-3)	1,118	0,058	0,726	0,000	0,970	0,673	0,763	0,003
1030511	COG0283F	gbs1455	cmk	Metabolism and transport	Purines, pyrimidines, nucleosides, and nucleotides	Cytidylate kinase (EC 2.7.4.14)	0,378	0,000	0,559	0,000	0,366	0,000	0,633	0,000
1030522	-	gbs1456		Hypothetical		Hypothetical membrane associated protein	0,626	0,000	0,940	0,367	0,619	0,000	1,051	0,289
1030519	COG1141C	gbs1457		Metabolism and transport	Energy production and conversion	Ferredoxin	0,596	0,004	0,749	0,032	0,491	0,000	0,569	0,000
1030518	-	gbs1458	ebsA	General function predicted only		Pore forming protein ebsA	0,810	0,027	0,857	0,115	0,734	0,011	0,934	0,193
1030517	COG2195E	gbs1459	pepT	Metabolism and transport	Amino acids, peptides, aminosugars and amines	Tripeptidase T (EC 3.4.11.4)	0,958	0,252	1,063	0,355	0,913	0,173	1,120	0,224
1030526	COG2244R	gbs1460		Cell Envelope	Biosynthesis and degradation of surface polysaccharides and lipopo	Export protein for polysaccharides and teichoic acids	0,618	0,002	1,001	0,995	0,635	0,002	0,918	0,422
1030524	COG0769M	gbs1461	murE	Cell Envelope	Biosynthesis and degradation of murein sacculus and peptidoglycar	UDP-N-acetylmuramoyl-L-alanyl-D-glutamate--lysine ligase (EC 6.3.2.7)	0,207	0,000	0,413	0,000	0,216	0,000	0,363	0,000
1030523	COG1120PH	gbs1462	fhuA	Metabolism and transport	Inorganic ion transport and metabolism	Ferrichrome transport ATP-binding protein fhuC	0,172	0,000	0,444	0,001	0,199	0,000	0,617	0,007
1030532	COG0614P	gbs1463	fhuD	Metabolism and transport	Inorganic ion transport and metabolism	Ferrichrome-binding protein	0,573	0,000	0,999	0,996	0,496	0,000	0,867	0,192
1030528	COG0609P	gbs1464	fhuB	Metabolism and transport	Inorganic ion transport and metabolism	Ferrichrome transport system permease protein fhuB	0,851	0,195	1,388	0,195	0,783	0,213	0,986	0,941
1030533	COG0609P	gbs1465	fhuG	Metabolism and transport	Inorganic ion transport and metabolism	Ferrichrome transport system permease protein fhuG	0,171	0,000	0,454	0,011	0,150	0,000	0,302	0,002
1030525	-	gbs1466		Hypothetical		Hypothetical cytosolic protein	0,356	0,000	0,629	0,003	0,477	0,000	0,523	0,000
1030534	COG1227C	gbs1467	ppaC	Metabolism and transport	Energy production and conversion	Inorganic pyrophosphatase (EC 3.6.1.1)	0,847	0,030	0,764	0,002	0,895	0,072	0,876	0,030
1030531	COG1180O	gbs1468	pflC	Metabolism and transport	Energy production and conversion	Pyruvate formate-lyase activating enzyme (EC 1.97.1.4)	1,110	0,728	1,940	0,020	1,181	0,580	1,909	0,025
1030539	COG1253R	gbs1469	hlyX	General function predicted only		Magnesium and cobalt efflux protein corC	0,505	0,000	0,700	0,002	0,503	0,000	0,807	0,006
1030537	-	gbs1470		Metabolism and transport	Central intermediary metabolism	SAM-dependent methyltransferase (EC 2.1.-.-)	0,604	0,000	0,669	0,001	0,505	0,000	0,689	0,003
1030543	COG1242R	gbs1471		General function predicted only		Radical SAM superfamily protein	0,571	0,000	0,503	0,000	0,472	0,000	0,581	0,000
1030536	COG0671I	gbs1472		Metabolism and transport	Fatty acid and phospholipids	Membrane-associated phospholipid phosphatase	1,291	0,327	0,388	0,002	1,211	0,441	0,279	0,001
1030535	COG3601S	gbs1473		Transport and binding proteins	Unknown substrate	Riboflavin transporter	1,934	0,021	0,726	0,038	1,667	0,063	0,488	0,001
1030548	COG4932M	gbs1474	pilC	hypothetical		LPXTG Hypothetical protein	0,581	0,007	0,612	0,010	0,635	0,012	0,412	0,001
1030545	COG3764M	gbs1475		Cellular processess	Posttranslational modification, protein turnover, chaperones	Sortase	0,286	0,000	0,439	0,002	0,315	0,000	0,181	0,000
1030542	COG3764M	gbs1476		Cellular processess	Posttranslational modification, protein turnover, chaperones	Sortase	0,483	0,003	0,805	0,261	0,497	0,004	0,416	0,002
1030552	-	gbs1477	pilB	Hypothetical		IPXTG Cell wall surface anchor family protein	1,225	0,032	0,401	0,000	1,104	0,345	0,161	0,000
1030551	COG4932M	gbs1478	pilA	Cell Envelope	Other	IPXTG Collagen adhesion protein	0,343	0,000	0,266	0,000	0,239	0,000	0,130	0,000
1030555	-	gbs1479	rogB	Cellular processess	Transcription	Transcriptional regulator RogB	0,516	0,002	0,326	0,000	0,467	0,001	0,422	0,001
1030549	COG0438M	gbs1480		Cell Envelope	Biosynthesis and degradation of surface polysaccharides and lipopo	N-acetylglucosaminyl-phosphatidylinositol biosynthetic protein	0,235	0,000	0,326	0,000	0,200	0,000	0,375	0,000
1030556	COG1216R	gbs1481		General function predicted only		dTDP-rhamnosyl transferase rfbF (EC 2.-.-.-)	0,147	0,000	0,269	0,000	0,149	0,000	0,342	0,000
1030557	COG2244R	gbs1482		General function predicted only		Oligosaccharide translocase (flippase)	0,202	0,000	0,478	0,000	0,208	0,000	0,408	0,000
1030553	-	gbs1483		Hypothetical		Hypothetical protein	0,216	0,000	0,560	0,003	0,239	0,000	0,467	0,000
1030563	COG0463M	gbs1484		Metabolism and transport	Central intermediary metabolism	Glycosyltransferase involved in cell wall biogenesis (EC 2.4.-.-)	0,268	0,000	0,494	0,000	0,243	0,000	0,505	0,001
1030560	COG0463M	gbs1485		Metabolism and transport	Central intermediary metabolism	Glycosyltransferase involved in cell wall biogenesis (EC 2.4.-.-)	0,274	0,000	0,530	0,001	0,287	0,000	0,491	0,000
1030566	COG0451MG	gbs1486		Hypothetical		UDP-D-glucuronate carboxy-lyase (EC 4.1.1.35)	0,312	0,000	0,508	0,000	0,311	0,000	0,544	0,000
1030559	COG1211I	gbs1487		Metabolism and transport	Fatty acid and phospholipids	2-C-methyl-D-erythritol 4-phosphate cytidylyltransferase (EC 2.7.7.60)	0,425	0,000	0,451	0,000	0,361	0,000	0,615	0,001
1030567	COG3475M	gbs1488		Cell Envelope	Biosynthesis and degradation of surface polysaccharides and lipopo	Lipooligosaccharide cholinephosphotransferase (EC 2.7.8.-)	0,372	0,000	0,794	0,016	0,384	0,000	0,660	0,001
1030565	-	gbs1489		Hypothetical		Hypothetical protein	0,196	0,000	0,277	0,000	0,160	0,000	0,360	0,000
1030564	COG2456S	gbs1490		Hypothetical		Hypothetical membrane spanning protein	0,176	0,000	0,332	0,000	0,184	0,000	0,352	0,000
1030570	COG0463M	gbs1491		Metabolism and transport	Central intermediary metabolism	Glycosyltransferase involved in cell wall biogenesis (EC 2.4.-.-)	0,354	0,000	0,526	0,000	0,338	0,000	0,541	0,000
1030568	COG0463M	gbs1492	rgpBc	Cell Envelope	Biosynthesis and degradation of murein sacculus and peptidoglycar	alpha-L-Rha alpha-1,3-L-rhamnosyltransferase (EC 2.4.1.-)	0,278	0,000	0,404	0,000	0,257	0,000	0,556	0,002
1030571	COG0438M	gbs1493	rgpAc	Cell Envelope	Biosynthesis and degradation of murein sacculus and peptidoglycar	alpha-D-GlcNAc alpha-1,2-L-rhamnosyltransferase (EC 2.4.1.-)	0,369	0,000	0,391	0,000	0,329	0,000	0,614	0,001
1030575	COG1091M	gbs1494	rmlD	Metabolism and transport	Carbohydrates, organic alcohols, and acids	dTDP-4-dehydrorhamnose reductase (EC 1.1.1.133)	0,845	0,158	0,548	0,000	0,760	0,021	0,515	0,000
1030577	-	gbs1495		Hypothetical		Hypothetical cytosolic protein	0,486	0,000	0,772	0,002	0,569	0,000	0,858	0,130
1030574	COG0568K	gbs1496	rpoD	Cellular processess	Transcription	RNA polymerase sigma factor rpoD	0,355	0,000	0,568	0,000	0,368	0,000	0,639	0,000
1030576	COG0358L	gbs1497	dnaG	Cellular processess	DNA replication, recombination and repair	DNA primase (EC 2.7.7.-)	0,286	0,000	0,607	0,000	0,197	0,000	0,762	0,004
1030572	-	gbs1498	mscL	Transport and binding proteins	Unknown substrate	Large-conductance mechanosensitive channel	2,014	0,000	1,524	0,003	2,045	0,000	1,196	0,024
1030581	COG0828J	gbs1499	rpsU	Cellular processess	Translation, ribosomal structure and biogenesis	SSU ribosomal protein S21P	1,950	0,000	1,628	0,001	2,323	0,000	1,699	0,000
1030579	COG0727R	gbs1500		Hypothetical		Hypothetical protein	0,741	0,357	0,393	0,008	0,685	0,243	0,438	0,029
1030580	COG0834ET	gbs1501		Metabolism and transport	Amino acids, peptides, aminosugars and amines	Arginine-binding protein	0,569	0,134	0,431	0,038	0,537	0,098	0,451	0,043
1030578	-	gbs1502		Metabolism and transport	Amino acids, peptides, aminosugars and amines	Ammonium transporter	3,732	0,000	6,020	0,002	4,401	0,001	5,698	0,000
1030584	COG0492O	gbs1503		Cellular processess	Posttranslational modification, protein turnover, chaperones	Oxidoreductase (EC 1.1.1.-)	1,203	0,681	1,786	0,224	1,580	0,196	1,400	0,329
1030583	COG1054R	gbs1504		Metabolism and transport	Central intermediary metabolism	Rhodanese-related sulfurtransferases	0,489	0,000	0,541	0,000	0,489	0,000	0,595	0,000
1030582	-	gbs1505		Hypothetical		Hypothetical protein	1,023	0,888	0,480	0,001	0,921	0,600	0,340	0,000
1030586	COG2271G	gbs1506		Metabolism and transport	Carbohydrates, organic alcohols, and acids	Transporter	0,753	0,338	0,560	0,044	1,008	0,974	1,040	0,851
1030592	COG0058G	gbs1507	glgP	Metabolism and transport	Carbohydrates, organic alcohols, and acids	Maltodextrin phosphorylase (EC 2.4.1.1)	1,790	0,001	3,301	0,000	1,944	0,019	4,216	0,000
1030595	-	gbs1508	malM	Metabolism and transport	Carbohydrates, organic alcohols, and acids	4-alpha-glucanotransferase (EC 2.4.1.25)	3,775	0,001	4,975	0,000	3,474	0,000	5,132	0,000
1030593	COG1609K	gbs1509	malR	Cellular processess	Transcription	Transcriptional regulator, LacI family	0,413	0,000	0,504	0,001	0,354	0,000	0,353	0,000
1030597	COG2182G	gbs1510	malE	Metabolism and transport	Carbohydrates, organic alcohols, and acids	Maltose maltodextrin-binding protein	5,885	0,000	8,400	0,000	6,737	0,000	9,489	0,000
1030591	COG1175G	gbs1511	malF	Metabolism and transport	Carbohydrates, organic alcohols, and acids	Maltodextrin transport system permease protein malC	2,050	0,005	4,713	0,000	2,385	0,001	5,222	0,000
1030599	COG3833G	gbs1512	malG	Metabolism and transport	Carbohydrates, organic alcohols, and acids	Maltose transport system permease protein malG	1,411	0,110	5,532	0,000	1,766	0,030	6,039	0,000
1030600	COG3104E	gbs1513		Metabolism and transport	Amino acids, peptides, aminosugars and amines	Di- tripeptide transporter	0,322	0,000	0,444	0,000	0,301	0,000	0,422	0,000
1030598	COG0494LR	gbs1514		Cellular processess	DNA replication, recombination and repair	Phosphohydrolase (MutT nudix family protein)	0,169	0,000	0,390	0,001	0,211	0,000	0,613	0,071
1030603	-	gbs1515		Hypothetical		Hypothetical protein	0,127	0,000	0,377	0,001	0,122	0,000	0,476	0,003
1030602	-	gbs1516		Hypothetical		surface protein Pls	0,399	0,001	0,849	0,300	0,354	0,001	1,023	0,854
1030607	COG0438M	gbs1517		Metabolism and transport	Central intermediary metabolism	Probable poly(Glycerol-phosphate) alpha-glucosyltransferase (EC 2.4.1.52)	0,273	0,000	0,434	0,000	0,239	0,000	0,529	0,001
1030610	COG0653U	gbs1518		Cellular processess	Posttranslational modification, protein turnover, chaperones	Protein translocase subunit secA	0,383	0,000	1,158	0,426	0,415	0,000	0,971	0,842
1030609	-	gbs1519		Hypothetical		Hypothetical protein	0,217	0,000	0,607	0,021	0,268	0,000	0,783	0,136
1030608	-	gbs1520		Hypothetical		Hypothetical protein	0,326	0,001	0,885	0,393	0,348	0,001	0,944	0,711
1030605	-	gbs1521		Hypothetical		Hypothetical protein	0,035	0,000	0,379	0,000	0,056	0,000	0,509	0,001
1030604	COG0201U	gbs1522		Cellular processess	Posttranslational modification, protein turnover, chaperones	Protein translocase subunit secY	0,274	0,000	0,736	0,034	0,336	0,000	1,037	0,793
1030612	-	gbs1523		Hypothetical		Hypothetical protein	0,389	0,000	1,036	0,832	0,341	0,000	1,047	0,645
1030613	COG0463M	gbs1524		Metabolism and transport	Central intermediary metabolism	Glycosyltransferase (EC 2.4.1.-)	0,292	0,000	0,694	0,092	0,242	0,000	0,859	0,173
1030614	COG1442M	gbs1525		Metabolism and transport	Central intermediary metabolism	Glycosyl transferase, family 8	0,163	0,000	0,632	0,006	0,152	0,000	0,803	0,138
1030611	COG1442M	gbs1526		Metabolism and transport	Central intermediary metabolism	Glycosyl transferase, family 8	0,102	0,000	0,475	0,005	0,137	0,000	0,773	0,023
1030617	COG1442M	gbs1527		Metabolism and transport	Central intermediary metabolism	Glycosyl transferase, family 8	0,600	0,002	1,424	0,028	0,835	0,162	1,372	0,015
1030616	-	gbs1528		Metabolism and transport	Carbohydrates, organic alcohols, and acids	Putative galactofuranosyltransferase (EC 2.4.1.-)	0,513	0,005	0,329	0,000	0,335	0,000	0,497	0,002
1030619	-	gbs1529		Cell Envelope	Other	LPXTG Fibronectin-binding protein	3,030	0,007	6,014	0,001	2,885	0,018	4,639	0,005
1030615	-	gbs1530	rofA	Cellular processess	Transcription	Transcriptional regulator RogB	0,837	0,458	0,695	0,208	0,705	0,194	0,957	0,836
1030621	COG0556L	gbs1531	UvrB	Cellular processess	DNA replication, recombination and repair	Excinuclease ABC subunit B	0,540	0,000	0,421	0,000	0,509	0,000	0,443	0,000
1030620	-	gbs1532		Cellular processess	Posttranslational modification, protein turnover, chaperones	CAAX amino terminal protease family	0,582	0,002	0,549	0,002	0,520	0,001	0,428	0,000
1030618	COG0765E, C	gbs1533	glnP	Metabolism and transport	Amino acids, peptides, aminosugars and amines	Glutamine-binding protein Glutamine transport system permease protein glnP	0,257	0,000	0,145	0,000	0,236	0,000	0,182	0,000
1030623	COG1126E	gbs1534	glnQ	Metabolism and transport	Amino acids, peptides, aminosugars and amines	Glutamine transport ATP-binding protein glnQ	0,420	0,000	0,303	0,000	0,403	0,000	0,344	0,000
1030622	-	gbs1535		Hypothetical		Hypothetical protein	0,275	0,000	0,543	0,000	0,237	0,000	0,669	0,000
1030625	-	gbs1536		Hypothetical		Hypothetical cytosolic protein	0,794	0,097	0,421	0,000	0,697	0,001	0,289	0,000
1030627	COG0536R	gbs1537	obg	General function predicted only		GTP-binding protein CgtA (probably involved in DNA repair)	0,568	0,000	0,564	0,000	0,593	0,000	0,548	0,000
1030626	COG2309E	gbs1538		Metabolism and transport	Amino acids, peptides, aminosugars and amines	Aminopeptidase S (EC 3.4.11.-)	1,634	0,001	1,311	0,114	1,759	0,001	1,809	0,000
1030624	-	gbs1539		Hypothetical		LPXTG Hypothetical protein	0,456	0,000	0,752	0,008	0,489	0,000	0,754	0,002

1030630	-	gbs1540		Metabolism and transport	Carbohydrates, organic alcohols, and acids	LPXTG Amidase family protein	0,816	0,039	0,980	0,871	0,717	0,005	1,056	0,544
1030632	COG1187J	gbs1541	rsuA	Cellular processess	Translation, ribosomal structure and biogenesis	Ribosomal small subunit pseudouridine synthase A (EC 4.2.1.70)	2,047	0,000	1,084	0,715	1,453	0,012	0,816	0,256
1030628	COG0656R	gbs1542		General function predicted only		Aldo keto reductase family	3,560	0,000	1,397	0,001	3,335	0,000	0,957	0,605
1030633	COG0778C	gbs1543		Metabolism and transport	Energy production and conversion	NAD(P)H-dependent quinone reductase (EC 1.-.-.-)	1,534	0,002	0,739	0,002	1,503	0,000	0,660	0,003
1030634	COG0346E	gbs1544	gloA	Metabolism and transport	Amino acids, peptides, aminosugars and amines	Lactoylglutathione lyase (EC 4.4.1.5)	1,218	0,035	0,744	0,009	1,187	0,038	0,615	0,000
1030631	COG0463M	gbs1545		Metabolism and transport	Central intermediary metabolism	Bactoprenol glucosyl transferase (EC 2.4.1.-)	0,615	0,037	0,267	0,000	0,517	0,004	0,564	0,009
1030636	COG1113E	gbs1546		Metabolism and transport	Amino acids, peptides, aminosugars and amines	Amino acid permease	0,288	0,000	0,371	0,000	0,286	0,000	0,374	0,000
1030639	COG0691O	gbs1547		Cellular processess	Posttranslational modification, protein turnover, chaperones	SsrA-binding protein	0,622	0,001	0,624	0,022	0,717	0,004	0,760	0,043
1030635	COG0557K	gbs1548		Cellular processess	DNA replication, recombination and repair	Exoribonuclease II (EC 3.1.13.1)	1,463	0,013	1,601	0,001	1,345	0,053	1,559	0,002
1030640	COG1314U	gbs1549	secG	Cellular processess	Posttranslational modification, protein turnover, chaperones	Protein translocase subunit secG	0,541	0,000	0,632	0,000	0,525	0,000	0,692	0,000
1030641	-	gbs1550		Cellular processess	Toxin production and resistance	Multidrug resistance efflux pump	0,700	0,025	0,726	0,012	0,675	0,005	0,820	0,077
1030637	-	gbs1551		Hypothetical		Hypothetical protein	0,578	0,000	0,834	0,155	0,755	0,068	0,599	0,002
1030638	COG1131V	gbs1552		Transport and binding proteins	Unknown substrate	ABC transporter ATP-binding protein	0,931	0,571	0,456	0,000	0,898	0,370	0,514	0,010
1030643	COG0237H	gbs1553	coaE	Metabolism and transport	Cofactors, prosthetic groups, and carriers	Dephospho-CoA kinase (EC 2.7.1.24)	0,399	0,000	0,849	0,357	0,376	0,000	1,064	0,638
1030644	COG0266L	gbs1554	fpg	Cellular processess	DNA replication, recombination and repair	Formamidopyrimidine-DNA glycosylase (EC 3.2.2.23)	0,423	0,000	0,669	0,108	0,443	0,000	0,826	0,125
1030646	-	gbs1555	mutR	Cellular processess	Transcription	Transcriptional regulator	0,417	0,000	0,695	0,064	0,228	0,000	0,916	0,417
1030642	COG5279D	gbs1556		Cell Envelope	Biosynthesis and degradation of murein sacculus and peptidoglycan	S-layer homology domain Putative murein endopeptidase	10,035	0,000	2,950	0,001	7,884	0,000	1,846	0,004
1030650	-	gbs1558		Cellular processess	Posttranslational modification, protein turnover, chaperones	CAAX amino terminal protease family	0,392	0,001	1,135	0,780	0,409	0,002	0,630	0,012
1030648	-	gbs1559		Hypothetical		Hypothetical protein	0,447	0,000	0,477	0,000	0,418	0,000	0,433	0,000
1030645	COG1159R	gbs1560	era	General function predicted only		GTP-binding protein era	0,464	0,000	0,530	0,000	0,369	0,000	0,530	0,000
1030652	-	gbs1561	dgk	Metabolism and transport	Fatty acid and phospholipids	Diacylglycerol kinase (EC 2.7.1.107)	0,521	0,000	0,599	0,000	0,425	0,000	0,568	0,000
1030653	COG0319R	gbs1562		Hypothetical		Hypothetical metal-binding protein	0,791	0,003	0,725	0,000	0,642	0,001	0,784	0,002
1030651	COG3315Q	gbs1563		Metabolism and transport	Secondary metabolites	Polyketide synthase O-methyltransferase (EC 2.1.1.-)	1,229	0,158	0,369	0,000	0,791	0,296	0,572	0,020
1030655	COG0494LR	gbs1564		Cellular processess	DNA replication, recombination and repair	Phosphohydrolase (MutT nudix family protein)	0,172	0,000	0,270	0,000	0,169	0,000	0,426	0,000
1030649	-	gbs1565		Hypothetical		Hypothetical protein	0,250	0,000	0,267	0,000	0,149	0,000	0,274	0,000
1030656	COG1702T	gbs1566	phoH	Cellular processess	Signal transduction	PhoH protein	0,220	0,000	0,362	0,000	0,225	0,000	0,314	0,000
1030654	COG4716S	gbs1567		General function predicted only		Myosin-crossreactive antigen	0,584	0,012	0,718	0,088	0,688	0,026	0,571	0,021
1030661	-	gbs1568		Hypothetical		Hypothetical cytosolic protein	0,484	0,001	0,377	0,000	0,489	0,001	0,450	0,001
1030660	-	gbs1569	msrA.2	Cellular processess	Posttranslational modification, protein turnover, chaperones	Peptide methionine sulfoxide reductase msrA (EC 1.8.4.6)	0,744	0,071	0,675	0,030	0,755	0,072	0,608	0,010
1030658	COG2996S	gbs1570		General function predicted only		S1 RNA binding domain	0,423	0,000	0,704	0,000	0,446	0,000	0,611	0,000
1030674	-	gbs1571	frr	Cellular processess	Translation, ribosomal structure and biogenesis	Ribosome Recycling Factor (RRF)	0,795	0,024	0,893	0,228	0,776	0,001	1,053	0,401
1031936	COG0528F	gbs1572	pyrH	Metabolism and transport	Purines, pyrimidines, nucleosides, and nucleotides	Uridylate kinase (EC 2.7.4.-)	0,587	0,000	0,667	0,000	0,580	0,000	0,701	0,000
1031802	COG1124EP	gbs1573		Metabolism and transport	Amino acids, peptides, aminosugars and amines	Nickel transport ATP-binding protein nikE	1,836	0,020	4,500	0,007	1,810	0,072	3,534	0,000
1030662	COG0444EP	gbs1574		Metabolism and transport	Amino acids, peptides, aminosugars and amines	Nickel transport ATP-binding protein nikD	1,790	0,021	4,032	0,000	2,111	0,104	4,580	0,000
1031803	COG1173EP	gbs1575		Metabolism and transport	Amino acids, peptides, aminosugars and amines	Nickel transport system permease protein nikC	0,819	0,372	3,369	0,031	1,092	0,860	5,084	0,075
1031800	COG0601EP	gbs1576		Metabolism and transport	Amino acids, peptides, aminosugars and amines	Nickel transport system permease protein nikB	1,464	0,510	2,480	0,027	0,891	0,708	4,995	0,107
1031804	COG0747E	gbs1577		Metabolism and transport	Amino acids, peptides, aminosugars and amines	Nickel-binding protein	1,543	0,177	3,008	0,013	2,208	0,007	3,423	0,000
1031398	-	gbs1578 (	rplA	Cellular processess	Translation, ribosomal structure and biogenesis	LSU ribosomal protein L1P	0,453	0,000	0,681	0,003	0,490	0,000	0,713	0,006
1031801	-	gbs1579 (	rplK	Cellular processess	Translation, ribosomal structure and biogenesis	LSU ribosomal protein L11P	0,332	0,000	0,528	0,000	0,347	0,000	0,559	0,000
1031806	-	gbs1580		Hypothetical		Hypothetical protein	0,861	0,310	0,723	0,243	0,746	0,090	0,424	0,001
1031805	COG1473R	gbs1581		General function predicted only		Carboxypeptidase, M20(D) family	0,904	0,575	0,456	0,000	1,032	0,765	0,313	0,000
1031811	COG0583K	gbs1582		Cellular processess	Transcription	Transcriptional regulators, LysR family	0,441	0,000	0,558	0,015	0,269	0,000	0,608	0,019
1031808	-	gbs1583		Hypothetical		Integral membrane protein	0,550	0,001	0,991	0,942	0,460	0,000	0,894	0,367
1031809	COG0115EH,	gbs1584		Metabolism and transport	Amino acids, peptides, aminosugars and amines	Para-aminobenzoate synthetase component I (EC 6.3.5.8) 4-amino-4-deoxychorisr	0,836	0,116	0,670	0,048	0,697	0,021	0,662	0,017
1031807	COG1674D	gbs1585	ftsK	Cellular processess	Cell division	Cell division protein ftsK	0,419	0,000	0,589	0,001	0,466	0,000	0,733	0,025
1031813	COG0652O	gbs1586		Cellular processess	Posttranslational modification, protein turnover, chaperones	Peptidyl-prolyl cis-trans isomerase (EC 5.2.1.8)	0,526	0,000	0,589	0,000	0,459	0,000	0,668	0,000
1031812	COG1108P	gbs1587	mtsC	Metabolism and transport	Inorganic ion transport and metabolism	Manganese transport system membrane protein	0,247	0,000	1,097	0,545	0,295	0,000	1,234	0,256
1031810	COG1121P	gbs1588	mtsB	Metabolism and transport	Inorganic ion transport and metabolism	Manganese transport system ATP-binding protein	0,455	0,001	1,134	0,482	0,409	0,000	1,471	0,044
1031817	COG0803P	gbs1589	mtsA	Metabolism and transport	Inorganic ion transport and metabolism	Manganese-binding protein	2,231	0,000	1,837	0,000	2,451	0,000	2,464	0,000
1031815	COG1321K	gbs1590	scaR	Cellular processess	Transcription	Iron-dependent repressor	1,507	0,106	1,450	0,002	1,557	0,024	1,847	0,001
1031814	COG0775F	gbs1591	pfs	Metabolism and transport	Purines, pyrimidines, nucleosides, and nucleotides	5 -methylthioadenosine nucleosidase (EC 3.2.2.16) S-adenosylhomocysteine nucle	0,609	0,000	0,479	0,000	0,583	0,000	0,602	0,000
1031818	-	gbs1592		Hypothetical		Hypothetical membrane associated protein	0,531	0,000	0,516	0,000	0,460	0,000	0,583	0,000
1031816	COG0494LR	gbs1593		Metabolism and transport	Purines, pyrimidines, nucleosides, and nucleotides	ADP-ribose pyrophosphatase (EC 3.6.1.13)	0,665	0,004	0,461	0,000	0,627	0,000	0,570	0,000
1031822	COG1207M	gbs1594	gcaD	Metabolism and transport	Amino acids, peptides, aminosugars and amines	Glucosamine-1-phosphate acetyltransferase (EC 2.3.1.-) UDP-N-acetylglucosamin	0,956	0,352	0,945	0,389	0,894	0,031	0,932	0,123
1031821	COG2764S	gbs1595		Metabolism and transport	Energy production and conversion	PhnB protein	3,176	0,015	1,278	0,379	2,795	0,029	0,891	0,500
1031819	COG0346E	gbs1596		Metabolism and transport	Amino acids, peptides, aminosugars and amines	Lactoylglutathione lyase (EC 4.4.1.5)	2,693	0,044	0,797	0,370	2,109	0,072	0,674	0,019
1031820	COG0673R	gbs1597		Metabolism and transport	Central intermediary metabolism	NAD-dependent oxidoreductase	0,607	0,000	0,702	0,007	0,607	0,001	0,648	0,001
1031823	-	gbs1598		Hypothetical		Hypothetical protein	0,518	0,000	0,379	0,000	0,427	0,000	0,383	0,000
1031823	-	gbs1598		Hypothetical		Hypothetical protein	0,694	0,002	0,683	0,002	0,608	0,000	0,662	0,001
1031837	-	gbs1599		Hypothetical		Hypothetical protein	0,661	0,001	0,534	0,000	0,571	0,000	0,613	0,000
1031834	COG1028IQR	gbs1600		Metabolism and transport	Fatty acid and phospholipids	Short chain dehydrogenase	0,771	0,032	0,633	0,023	0,593	0,001	0,602	0,016
1031833	-	gbs1601		Hypothetical		Hypothetical protein	1,577	0,001	0,825	0,061	1,221	0,019	0,988	0,927
1031830	-	gbs1602		General function predicted only		DNA-binding protein	0,672	0,008	0,607	0,009	0,535	0,002	0,672	0,008
1031824	-	gbs1603		Hypothetical		Hypothetical protein	0,975	0,772	0,780	0,107	0,784	0,027	0,713	0,007
1031831	COG1215M	gbs1605		Metabolism and transport	Central intermediary metabolism	Glycosyltransferase (EC 2.4.1.-)	1,744	0,017	2,502	0,007	2,032	0,002	3,008	0,001
1031839	-	gbs1606		Hypothetical		Hypothetical protein	0,556	0,105	1,599	0,103	0,715	0,288	1,136	0,729
1031836	-	gbs1607		Hypothetical		Hypothetical protein	1,479	0,055	1,636	0,168	1,173	0,661	2,480	0,012
1031835	COG1285S	gbs1608		Hypothetical		Hypothetical protein	2,262	0,000	5,679	0,025	3,042	0,004	1,734	0,219
1031844	-	gbs1609		Hypothetical		Hypothetical protein	1,789	0,342	3,409	0,012	1,658	0,430	2,633	0,084
1031843	COG1114E	gbs1610		Metabolism and transport	Amino acids, peptides, aminosugars and amines	Branched-chain amino acid transport system carrier protein	0,460	0,001	0,398	0,000	0,486	0,002	0,366	0,000
1031842	COG0073R, (	gbs1611	metG	Cellular processess	Translation, ribosomal structure and biogenesis	Methionyl-tRNA synthetase (EC 6.1.1.10) Protein secretion chaperonin CsaA	0,679	0,000	0,643	0,001	0,690	0,000	0,808	0,006
1031845	COG3615P	gbs1612		Metabolism and transport	Inorganic ion transport and metabolism	Tellurite resistance protein tehB	1,267	0,098	1,208	0,168	1,186	0,102	1,346	0,048
1031841	-	gbs1613		Hypothetical		Hypothetical membrane spanning protein	0,408	0,000	1,127	0,487	0,619	0,040	1,155	0,245
1031846	COG1455G	gbs1614		Metabolism and transport	Carbohydrates, organic alcohols, and acids	PTS system, cellobiose-specific IIC component	0,539	0,002	0,324	0,000	0,520	0,001	0,391	0,000
1031840	COG4814R	gbs1615		General function predicted only		putative acyltransferases and hydrolases with the alpha beta hydrolase fold	0,331	0,000	0,312	0,000	0,335	0,000	0,308	0,000
1031848	COG0708L	gbs1616	exoA	Cellular processess	DNA replication, recombination and repair	Exodeoxyribonuclease III (EC 3.1.11.2)	0,364	0,000	0,418	0,000	0,416	0,000	0,419	0,000
1031847	COG1393P	gbs1617		Metabolism and transport	Inorganic ion transport and metabolism	Arsenate reductase family protein	2,056	0,000	0,902	0,249	1,701	0,001	0,843	0,049
1031851	COG0350L	gbs1618		Metabolism and transport	Energy production and conversion	O6-methylguanine-DNA methyltransferase (EC 2.1.1.63)	1,198	0,037	0,727	0,002	0,810	0,027	0,674	0,016
1031849	COG0111HE	gbs1619		Metabolism and transport	Carbohydrates, organic alcohols, and acids	D-3-phosphoglycerate dehydrogenase (EC 1.1.1.95)	0,467	0,000	0,222	0,000	0,454	0,000	0,187	0,000
1031850	-	gbs1620		Metabolism and transport	Central intermediary metabolism	Acetyltransferase (EC 2.3.1.-)	0,377	0,002	0,142	0,000	0,356	0,001	0,161	0,000
1031852	COG1932HE	gbs1621	serC	Metabolism and transport	Amino acids, peptides, aminosugars and amines	Phosphoserine aminotransferase (EC 2.6.1.52)	0,908	0,546	0,413	0,002	0,874	0,403	0,288	0,001
1031856	COG3142P	gbs1622	cutC	Metabolism and transport	Inorganic ion transport and metabolism	Copper homeostasis protein cutC	0,962	0,772	0,785	0,167	0,778	0,121	1,025	0,843
1031854	COG0313R	gbs1623		General function predicted only		Tetrapyrrole (Corrin Porphyrin) methylase family protein	0,377	0,000	0,522	0,000	0,328	0,000	0,507	0,000
1031853	COG4467S	gbs1624		Hypothetical		Initiation-control protein	0,495	0,000	0,498	0,000	0,432	0,000	0,407	0,000
1031855	COG0470L	gbs1625	dnaX	Cellular processess	DNA replication, recombination and repair	DNA polymerase III, delta subunit (EC 2.7.7.7)	0,529	0,000	0,573	0,000	0,451	0,000	0,552	0,000
1031858	COG0125F	gbs1626	tmk	Metabolism and transport	Purines, pyrimidines, nucleosides, and nucleotides	Thymidylate kinase (EC 2.7.4.9)	0,953	0,708	0,809	0,019	0,891	0,302	0,862	0,080
1031863	COG0517R	gbs1627		Metabolism and transport	Energy production and conversion	Acetoin utilization acuB protein	0,481	0,108	0,237	0,027	0,448	0,089	0,183	0,020
1031860	COG0410E	gbs1628		Metabolism and transport	Amino acids, peptides, aminosugars and amines	Branched-chain amino acid transport ATP-binding protein livF	0,434	0,077	0,156	0,017	0,378	0,056	0,135	0,015

1031861	COG0411E	gbs1629		Metabolism and transport	Amino acids, peptides, aminosugars and amines	Branched-chain amino acid transport ATP-binding protein livG	0,337	0,039	0,150	0,014	0,293	0,031	0,109	0,012
1031864	COG4177E	gbs1630		Metabolism and transport	Amino acids, peptides, aminosugars and amines	Branched-chain amino acid transport system permease protein livM	0,403	0,059	0,179	0,016	0,369	0,046	0,137	0,013
1031862	COG0559E	gbs1631		Metabolism and transport	Amino acids, peptides, aminosugars and amines	Branched-chain amino acid transport system permease protein livH	0,418	0,054	0,299	0,026	0,445	0,065	0,254	0,020
1031859	COG0683E	gbs1632		Metabolism and transport	Amino acids, peptides, aminosugars and amines	Leucine-, isoleucine-, valine-, threonine-, and alanine-binding protein	0,532	0,026	0,333	0,003	0,519	0,024	0,301	0,003
1031857	-	gbs1633		Hypothetical		Hypothetical cytosolic protein	0,328	0,000	0,391	0,000	0,317	0,000	0,371	0,000
1031870	COG0740OU	gbs1634	clpP	Cellular processess	Posttranslational modification, protein turnover, chaperones	ATP-dependent endopeptidase clp proteolytic subunit clpP (EC 3.4.21.92)	1,448	0,000	0,723	0,018	1,400	0,003	0,658	0,002
1031867	COG0035F	gbs1635	upp	Metabolism and transport	Purines, pyrimidines, nucleosides, and nucleotides	Uracil phosphoribosyltransferase (EC 2.4.2.9)	1,601	0,002	0,753	0,002	1,356	0,001	0,594	0,000
1031865	COG1168E	gbs1636		Metabolism and transport	Amino acids, peptides, aminosugars and amines	Cystathionine beta-lyase (EC 4.4.1.8)	0,649	0,003	0,527	0,003	0,588	0,011	0,403	0,000
1031866	COG0219J	gbs1637		Cellular processess	Translation, ribosomal structure and biogenesis	23S rRNA methyltransferase (EC 2.1.1.-)	0,752	0,457	0,445	0,014	0,654	0,074	0,368	0,003
1031868	COG0531E	gbs1638		Metabolism and transport	Amino acids, peptides, aminosugars and amines	Amino acid permease	0,257	0,000	0,526	0,000	0,222	0,000	0,482	0,000
1031872	COG0569P	gbs1639		Metabolism and transport	Inorganic ion transport and metabolism	Trk system potassium uptake protein trkA	0,432	0,000	0,681	0,056	0,427	0,000	0,477	0,000
1031871	COG0168P	gbs1640		Metabolism and transport	Inorganic ion transport and metabolism	Trk system potassium uptake protein trkH	0,190	0,000	0,221	0,000	0,245	0,000	0,193	0,000
1031874	-	gbs1641		Hypothetical		Hypothetical cytosolic protein	0,482	0,000	0,841	0,053	0,482	0,000	1,041	0,742
1031876	COG1187J	gbs1642		Cellular processess	Translation, ribosomal structure and biogenesis	Ribosomal large subunit pseudouridine synthase B (EC 4.2.1.70)	0,307	0,000	0,716	0,015	0,264	0,000	0,889	0,233
1031869	-	gbs1643		Cellular processess	Cell division	Segregation and condensation protein ScpB	0,383	0,000	1,030	0,811	0,405	0,000	1,235	0,022
1031877	COG1354S	gbs1644	scpA	Cellular processess	Cell division	Segregation and condensation protein ScpA	0,180	0,000	0,542	0,001	0,137	0,000	0,806	0,087
1031878	-	gbs1645		Mobile and extrachromosomal elements		DNA integration recombination inversion protein	0,247	0,000	1,034	0,744	0,283	0,000	1,135	0,155
1031875	COG0517R	gbs1646		General function predicted only		CBS domain containing protein	0,260	0,000	0,670	0,032	0,226	0,000	0,956	0,634
1031882	COG0622R	gbs1647		General function predicted only		putative phosphoesterase	0,311	0,000	0,677	0,000	0,296	0,000	0,862	0,051
1031879	COG0127F	gbs1648		Metabolism and transport	Purines, pyrimidines, nucleosides, and nucleotides	Xanthosine triphosphate pyrophosphatase (EC 3.6.1.-)	0,490	0,000	0,994	0,958	0,447	0,000	1,192	0,007
1031880	COG0796M	gbs1649	glr	Cell Envelope	Biosynthesis and degradation of murein sacculus and peptidoglycar	Glutamate racemase (EC 5.1.1.3)	0,337	0,000	0,660	0,001	0,290	0,000	0,790	0,041
1031873	COG3763S	gbs1650		Hypothetical		Hypothetical exported protein	0,524	0,000	0,723	0,000	0,500	0,000	0,908	0,051
1031883	-	gbs1651		Hypothetical		Hypothetical protein	6,616	0,000	2,948	0,022	4,729	0,003	4,260	0,007
1031884	-	gbs1652		Cellular processess	Transcription	Transcriptional regulator, biotin repressor family	0,911	0,363	0,527	0,000	0,886	0,235	0,467	0,000
1031881	COG0670R	gbs1653		Hypothetical		Integral membrane protein	1,105	0,076	0,835	0,016	1,056	0,346	0,840	0,008
1031889	COG1418R	gbs1654		Metabolism and transport	Central intermediary metabolism	Hydrolase (HAD superfamily)	1,304	0,002	0,741	0,001	1,233	0,006	0,669	0,000
1031886	COG0566J	gbs1655		Cellular processess	Translation, ribosomal structure and biogenesis	23S rRNA methyltransferase (EC 2.1.1.-)	0,355	0,000	0,588	0,000	0,329	0,000	0,632	0,000
1031887	COG1254C	gbs1656		Metabolism and transport	Energy production and conversion	Acylphosphatase (EC 3.6.1.7)	1,326	0,087	0,678	0,045	1,488	0,044	0,575	0,012
1031885	COG0706U	gbs1657		Cell Envelope	Other	60 kDa inner membrane protein YIDC	0,472	0,000	0,670	0,001	0,470	0,000	0,780	0,007
1031891	COG0765E	gbs1658		Metabolism and transport	Amino acids, peptides, aminosugars and amines	Probable amino-acid ABC transporter permease protein yckA	0,785	0,024	0,841	0,091	0,781	0,022	0,762	0,014
1030732	COG0834ET	gbs1659		Metabolism and transport	Amino acids, peptides, aminosugars and amines	Cystine-binding protein	0,904	0,311	0,933	0,436	0,840	0,089	0,860	0,140
1029816	-	gbs1660	amiC	Metabolism and transport	Amino acids, peptides, aminosugars and amines	Amidase family protein	1,253	0,129	0,532	0,003	1,095	0,553	0,460	0,001
1031179	COG0782K	gbs1661	greA	Cellular processess	Transcription	Transcription elongation factor greA	0,350	0,000	0,348	0,000	0,343	0,000	0,412	0,000
1031229	-	gbs1662		Hypothetical		Hypothetical membrane associated protein	0,517	0,000	0,653	0,000	0,477	0,000	0,741	0,001
1031232	-	gbs1663		Metabolism and transport	Central intermediary metabolism	Acetyltransferase, GNAT family	0,148	0,000	0,189	0,000	0,149	0,000	0,344	0,000
1030666	COG0773M	gbs1664	murC	Cell Envelope	Biosynthesis and degradation of murein sacculus and peptidoglycar	UDP-N-acetylmuramate--alanine ligase (EC 6.3.2.8)	0,328	0,000	0,476	0,000	0,278	0,000	0,435	0,000
1030737	-	gbs1665		Hypothetical		Hypothetical cytosolic protein	0,450	0,000	0,544	0,000	0,444	0,000	0,567	0,000
1031231	COG0553KL	gbs1666	snf	Hypothetical		SWF SNF family helicase	0,339	0,000	0,339	0,000	0,291	0,000	0,453	0,000
1031116	COG1160R	gbs1667	pgdA	General function predicted only		GTP-binding protein	0,462	0,000	0,670	0,000	0,488	0,000	0,657	0,000
1030812	COG1484L	gbs1668	dnal	Cellular processess	DNA replication, recombination and repair	Primosomal protein dnaI	0,498	0,000	0,797	0,037	0,480	0,000	0,761	0,026
1030665	COG3611L	gbs1669	dnaB	Cellular processess	DNA replication, recombination and repair	Replicative DNA helicase (EC 3.6.1.-)	0,386	0,000	0,622	0,001	0,400	0,000	0,695	0,009
1030833	-	gbs1670		Cellular processess	Transcription	Putative regulatory protein	0,649	0,000	0,789	0,000	0,719	0,000	0,895	0,108
1030601	COG0642T	gbs1671	covS	Cellular processess	Signal transduction	Transmembrane histidine kinase CsrS	0,344	0,000	0,679	0,001	0,329	0,000	0,810	0,031
1031230	COG0745TK	gbs1672	covR	Cellular processess	Signal transduction	Response regulator CsrR	0,490	0,000	0,690	0,000	0,483	0,000	0,715	0,000
1031227	COG1399R	gbs1673		Hypothetical		Hypothetical cytosolic protein	0,452	0,000	0,631	0,002	0,429	0,000	0,589	0,001
1030606	COG0501O	gbs1674		Cellular processess	Posttranslational modification, protein turnover, chaperones	Endopeptidase htpX (EC 3.4.24.-)	2,135	0,000	0,661	0,001	2,013	0,000	0,697	0,000
1030706	-	gbs1675	lemA	General function predicted only		LemA protein	4,089	0,000	1,631	0,013	4,177	0,000	1,296	0,044
1030734	COG0357M	gbs1676	gidB	Metabolism and transport	Central intermediary metabolism	Methyltransferase gidB (EC 2.1.-.-)	0,227	0,000	0,332	0,000	0,238	0,000	0,297	0,000
1030714	COG0168P	gbs1677		Metabolism and transport	Inorganic ion transport and metabolism	Potassium uptake protein ktrB	0,359	0,000	0,459	0,001	0,394	0,000	0,445	0,000
1031228	COG0569P	gbs1678		Metabolism and transport	Inorganic ion transport and metabolism	Potassium uptake protein ktrA	0,493	0,000	0,547	0,000	0,546	0,000	0,675	0,001
1030689	COG0619P	gbs1679		Metabolism and transport	Inorganic ion transport and metabolism	Cobalt transport protein cbiQ	0,268	0,000	0,390	0,000	0,293	0,000	0,471	0,000
1030735	COG1122P	gbs1680		Metabolism and transport	Inorganic ion transport and metabolism	Cobalt transport ATP-binding protein cbiO	0,116	0,000	0,252	0,000	0,139	0,000	0,280	0,000
1030541	COG4720S	gbs1681		Hypothetical		Hypothetical protein	0,396	0,000	0,454	0,000	0,358	0,000	0,516	0,000
1031530	COG3633E	gbs1682		Metabolism and transport	Amino acids, peptides, aminosugars and amines	Serine threonine sodium symporter	0,292	0,000	0,169	0,000	0,275	0,001	0,093	0,000
1030673	COG1114E	gbs1683	braB	Metabolism and transport	Amino acids, peptides, aminosugars and amines	Branched-chain amino acid transport system carrier protein	0,270	0,000	0,265	0,000	0,289	0,000	0,219	0,000
1031574	COG1063ER	gbs1684		Metabolism and transport	Carbohydrates, organic alcohols, and acids	Zn-dependent alcohol dehydrogenases and related dehydrogenases	18,572	0,001	14,440	0,001	21,482	0,001	14,983	0,008
1031563	COG2011P	gbs1685		Transport and binding proteins	Unknown substrate	ABC transporter permease protein	0,425	0,009	0,457	0,012	0,458	0,012	0,448	0,011
1031562	COG1135P	gbs1686		Transport and binding proteins	Unknown substrate	ABC transporter ATP-binding protein	0,319	0,006	0,319	0,006	0,306	0,006	0,369	0,008
1031570	COG0624E	gbs1687		Metabolism and transport	Amino acids, peptides, aminosugars and amines	Acetylornithine deacetylase Succinyl-diaminopimelate desuccinylase and related de	0,269	0,012	0,134	0,006	0,150	0,006	0,254	0,011
1031051	COG1464P	gbs1688		Transport and binding proteins	Unknown substrate	ABC transporter substrate-binding protein	0,321	0,000	0,216	0,000	0,330	0,000	0,271	0,000
1030774	COG0834ET	gbs1689		Transport and binding proteins	Unknown substrate	ABC transporter substrate-binding protein	2,677	0,001	2,396	0,000	2,394	0,001	1,807	0,004
1031549	COG2071R	gbs1690		General function predicted only		Glutamine amidotransferase, class I	2,691	0,000	1,825	0,072	2,052	0,023	1,424	0,273
1031325	-	gbs1691		Hypothetical		Hypothetical cytosolic protein	0,746	0,004	0,715	0,000	0,697	0,000	0,713	0,000
1031277	COG2376G	gbs1692		Metabolism and transport	Carbohydrates, organic alcohols, and acids	Dihydroxyacetone kinase (EC 2.7.1.29)	1,591	0,001	1,501	0,233	1,382	0,058	1,390	0,132
1031198	COG1309K	gbs1693		Cellular processess	Transcription	Transcriptional regulator, TetR family	1,462	0,173	0,977	0,910	1,330	0,474	1,398	0,318
1030356	COG2376G	gbs1694		Metabolism and transport	Carbohydrates, organic alcohols, and acids	Dihydroxyacetone kinase (EC 2.7.1.29)	2,157	0,019	6,320	0,001	2,592	0,005	7,567	0,001
1030362	COG2376G	gbs1695		Metabolism and transport	Carbohydrates, organic alcohols, and acids	Dihydroxyacetone kinase (EC 2.7.1.29)	2,509	0,000	7,917	0,004	2,761	0,002	10,284	0,001
1030340	COG3412S	gbs1696		Metabolism and transport	Fatty acid and phospholipids	Dihydroxyacetone kinase phosphotransfer protein	0,718	0,445	7,673	0,001	1,731	0,219	10,260	0,008
1030843	-	gbs1697		Metabolism and transport	Carbohydrates, organic alcohols, and acids	Glycerol uptake facilitator protein	1,867	0,002	7,383	0,003	1,712	0,076	8,461	0,001
1029835	COG0662G	gbs1698		Hypothetical		Hypothetical cytosolic protein	3,303	0,000	1,501	0,000	3,458	0,000	1,139	0,124
1030365	COG0789K	gbs1699		Cellular processess	Transcription	Transcriptional activator tipA	0,616	0,014	0,443	0,011	0,305	0,000	0,152	0,000
1031422	COG1323R	gbs1700		Hypothetical		Hypothetical cytosolic protein	0,563	0,020	0,841	0,385	0,479	0,004	0,902	0,558
1030792	-	gbs1701		Hypothetical		Hypothetical protein	0,361	0,000	0,478	0,015	0,398	0,001	0,451	0,002
1031378	COG0500QR	gbs1702		Metabolism and transport	Secondary metabolites	Methyltransferase (EC 2.1.1.-)	0,339	0,000	0,293	0,000	0,371	0,000	0,359	0,000
1031376	-	gbs1703		Hypothetical		iojap protein family	0,280	0,000	0,429	0,000	0,296	0,000	0,354	0,000
1030344	COG1335Q	gbs1704		Metabolism and transport	Secondary metabolites	Isochorismatase family protein	0,206	0,000	0,393	0,000	0,176	0,000	0,334	0,000
1030352	COG1713H	gbs1705		Metabolism and transport	Cofactors, prosthetic groups, and carriers	Hydrolase (HAD superfamily)	0,185	0,000	0,305	0,000	0,200	0,000	0,321	0,000
1031292	COG1057H	gbs1706	nadD	Metabolism and transport	Cofactors, prosthetic groups, and carriers	Nicotinate-nucleotide adenylyltransferase (EC 2.7.7.18)	0,221	0,000	0,348	0,000	0,200	0,000	0,369	0,000
1030078	-	gbs1707		Hypothetical		hypothetical RNA binding protein	0,149	0,000	0,209	0,000	0,140	0,000	0,265	0,000
1031389	COG1161R	gbs1708		General function predicted only		GTP-binding protein	0,354	0,000	0,654	0,003	0,349	0,000	0,641	0,003
1031668	COG2179R	gbs1709		General function predicted only		Putative lipase	0,131	0,000	0,193	0,000	0,143	0,000	0,179	0,000
1031355	COG0697GEI	gbs1710		Transport and binding proteins	Unknown substrate	Transporter, drug metabolite exporter family	0,625	0,048	1,649	0,103	0,759	0,273	1,380	0,092
1030455	COG0064J	gbs1711	gatB	Cellular processess	Translation, ribosomal structure and biogenesis	Aspartyl glutamyl-tRNA(Asn Gln) amidotransferase subunit B (EC 6.3.5.-)	0,509	0,000	0,694	0,000	0,513	0,000	0,850	0,049
1030183	-	gbs1712	gatA	Cellular processess	Translation, ribosomal structure and biogenesis	Aspartyl glutamyl-tRNA(Asn Gln) amidotransferase subunit A (EC 6.3.5.-)	0,228	0,000	0,373	0,000	0,237	0,000	0,455	0,000
1029862	COG0721J	gbs1713	gatC	Cellular processess	Translation, ribosomal structure and biogenesis	Aspartyl glutamyl-tRNA(Asn Gln) amidotransferase subunit C (EC 6.3.5.-)	0,297	0,000	0,497	0,000	0,255	0,000	0,653	0,000
1030268	COG0574G	gbs1714		Metabolism and transport	Carbohydrates, organic alcohols, and acids	Pyruvate,phosphate dikinase (EC 2.7.9.1)	0,452	0,010	10,602	0,020	0,858	0,644	14,004	0,011
1031461	-	gbs1715		General function predicted only		ATP GTP-binding protein	0,502	0,082	6,961	0,054	0,306	0,012	9,019	0,042
1031400	COG0517R	gbs1716		General function predicted only		CBS domain containing protein	1,036	0,863	6,726	0,017	0,884	0,604	8,684	0,009

1030863	COG1250I	gbs1717		Metabolism and transport	Fatty acid and phospholipids	3-hydroxybutyryl-CoA dehydrogenase (EC 1.1.1.157)	1,391	0,113	2,778	0,002	1,298	0,253	2,599	0,002
1030063	COG1335Q	gbs1718		Metabolism and transport	Secondary metabolites	Pyrazinamidase (EC 3.5.1.-) Nicotinamidase (EC 3.5.1.19)	0,659	0,001	0,388	0,000	0,575	0,000	0,461	0,000
1031455	-	gbs1719	codY	Cellular processess	Transcription	Transcription pleiotropic repressor codY	0,288	0,000	0,500	0,000	0,284	0,000	0,524	0,000
1030746	COG0436E	gbs1720		Metabolism and transport	Amino acids, peptides, aminosugars and amines	Aspartate aminotransferase (EC 2.6.1.1)	0,552	0,000	0,488	0,000	0,530	0,000	0,527	0,000
1030629	COG0589T	gbs1721		Cellular processess	Posttranslational modification, protein turnover, chaperones	Universal stress protein family	8,390	0,000	8,964	0,000	10,052	0,000	6,531	0,001
1031541	COG0561R, (	gbs1722		Metabolism and transport	Central intermediary metabolism	Hydrolase (HAD superfamily)	0,444	0,000	1,036	0,824	0,333	0,000	0,885	0,345
1030529	-	gbs1723	asnB	Metabolism and transport	Amino acids, peptides, aminosugars and amines	L-asparaginase (EC 3.5.1.1)	0,581	0,000	0,496	0,002	0,503	0,000	0,640	0,005
1030023	COG0169E	gbs1724	aroE	Metabolism and transport	Amino acids, peptides, aminosugars and amines	Shikimate 5-dehydrogenase (EC 1.1.1.25)	0,947	0,666	0,604	0,041	0,974	0,910	0,740	0,138
1030846	COG4989R	gbs1725		Metabolism and transport	Central intermediary metabolism	Oxidoreductase (EC 1.1.1.-)	2,170	0,000	1,404	0,120	2,319	0,000	1,424	0,051
1030858	COG1200LK	gbs1726	recG	Cellular processess	DNA replication, recombination and repair	ATP-dependent DNA helicase recG (EC 3.6.1.-)	0,714	0,004	0,993	0,961	0,804	0,072	0,966	0,744
1030170	COG3942R	gbs1727	isp2	General function predicted only		Immunogenic secreted protein	0,449	0,000	0,516	0,000	0,437	0,000	0,583	0,000
1029940	COG0787M	gbs1728	alr	Cell Envelope	Biosynthesis and degradation of murein sacculus and peptidoglycar	Alanine racemase (EC 5.1.1.1)	0,452	0,000	0,342	0,000	0,325	0,000	0,360	0,000
1030345	COG0736I	gbs1729	acpS	Metabolism and transport	Fatty acid and phospholipids	Holo-[acyl-carrier protein] synthase (EC 2.7.8.7)	0,442	0,000	0,515	0,008	0,270	0,000	0,724	0,081
1031536	COG0722E	gbs1730		Metabolism and transport	Amino acids, peptides, aminosugars and amines	3-deoxy-7-phosphoheptulonate synthase (EC 2.5.1.54)	0,401	0,000	0,550	0,000	0,303	0,000	0,646	0,001
1030346	COG0653U	gbs1731	secA	Cellular processess	Posttranslational modification, protein turnover, chaperones	Protein translocase subunit secA	0,566	0,000	0,657	0,000	0,553	0,000	0,702	0,000
1030410	COG1482G	gbs1732	pmi	Metabolism and transport	Carbohydrates, organic alcohols, and acids	Mannose-6-phosphate isomerase (EC 5.3.1.8)	0,368	0,000	0,460	0,001	0,339	0,000	0,543	0,000
1030394	COG1940KG	gbs1733	scrK	Metabolism and transport	Carbohydrates, organic alcohols, and acids	Fructokinase (EC 2.7.1.4)	2,012	0,038	2,504	0,041	1,750	0,207	3,514	0,019
1030249	COG1263G	gbs1734	scrA	Metabolism and transport	Carbohydrates, organic alcohols, and acids	PTS system, sucrose-specific IIABC component (EC 2.7.1.69)	1,430	0,108	2,533	0,121	1,316	0,263	3,659	0,002
1030678	-	gbs1735	scrB	Metabolism and transport	Carbohydrates, organic alcohols, and acids	Sucrose-6-phosphate hydrolase (EC 3.2.1.26)	0,480	0,005	1,168	0,719	0,609	0,065	1,606	0,013
1030460	COG1609K	gbs1736	scrR	Cellular processess	Transcription	Sucrose operon repressor	0,803	0,262	1,004	0,980	0,416	0,000	1,598	0,009
1030497	COG0781K	gbs1737	nusB	Cellular processess	Transcription	N utilization substance protein B	0,559	0,000	0,587	0,000	0,539	0,000	0,751	0,000
1030404	-	gbs1738		Cellular processess	Posttranslational modification, protein turnover, chaperones	General stress protein, Gls24 family	0,794	0,015	0,629	0,000	0,658	0,000	0,608	0,000
1030682	COG0231J	gbs1739	efp	Cellular processess	Translation, ribosomal structure and biogenesis	Protein Translation Elongation Factor P (EF-P)	0,832	0,025	0,803	0,000	0,694	0,000	0,813	0,000
1030540	COG1132V	gbs1740		Transport and binding proteins	Unknown substrate	ABC transporter ATP-binding and permease protein	1,236	0,434	8,388	0,000	1,548	0,300	12,934	0,000
1030681	COG1132V	gbs1741		Transport and binding proteins	Unknown substrate	ABC transporter, ATP-binding/permease protein	1,664	0,039	6,332	0,000	2,084	0,014	8,271	0,000
1030676	COG1122P	gbs1742		Metabolism and transport	Inorganic ion transport and metabolism	Cobalt transport ATP-binding protein cbiO	1,019	0,949	7,193	0,000	1,037	0,876	9,942	0,000
1030686	COG0619P	gbs1743		Metabolism and transport	Inorganic ion transport and metabolism	Cobalt permease and related transporters	1,209	0,354	8,250	0,000	1,190	0,562	10,729	0,000
1030679	-	gbs1744		Hypothetical		membrane-bound protein	0,764	0,399	8,469	0,000	0,597	0,045	12,364	0,000
1030680	-	gbs1745		Hypothetical		Hypothetical protein	1,231	0,440	7,073	0,001	1,148	0,648	10,542	0,000
1030677	-	gbs1746		Hypothetical		Hypothetical protein	1,019	0,933	8,581	0,000	1,546	0,222	12,212	0,000
1030684	COG1021Q	gbs1748		Metabolism and transport	Secondary metabolites	2,3-dihydroxybenzoate-AMP ligase (EC 2.7.7.58)	1,954	0,053	9,709	0,000	1,755	0,082	10,880	0,000
1030683	COG1321K	gbs1749		Cellular processess	Transcription	Iron-dependent repressor	0,253	0,000	0,393	0,000	0,256	0,000	0,525	0,000
1030685	COG2131F	gbs1750	comEB	Metabolism and transport	Purines, pyrimidines, nucleosides, and nucleotides	ComE operon protein 2	0,402	0,000	0,332	0,000	0,349	0,000	0,510	0,000
1030715	COG0006E	gbs1751		Metabolism and transport	Amino acids, peptides, aminosugars and amines	Xaa-Pro dipeptidase (EC 3.4.13.9)	0,718	0,004	0,619	0,006	0,684	0,004	0,475	0,000
1030708	-	gbs1752		Hypothetical		Hypothetical protein	0,826	0,214	1,135	0,516	0,866	0,491	1,564	0,001
1030687	-	gbs1753		Cellular processess	Toxin production and resistance	Multidrug resistance protein B	1,285	0,203	1,996	0,079	1,532	0,062	2,177	0,003
1030705	COG0178L	gbs1754	uvrA	Cellular processess	DNA replication, recombination and repair	Excinuclease ABC subunit A	0,722	0,057	0,707	0,114	0,779	0,157	0,576	0,015
1030719	-	gbs1755		Hypothetical		Hypothetical membrane spanning protein	0,423	0,000	0,492	0,000	0,399	0,000	0,543	0,001
1030723	COG0598P	gbs1756	corA	Metabolism and transport	Inorganic ion transport and metabolism	Magnesium and cobalt transport protein corA	0,431	0,000	0,462	0,000	0,449	0,001	0,565	0,002
1030704	-	gbs1757	rpsR	Cellular processess	Translation, ribosomal structure and biogenesis	SSU ribosomal protein S18P	1,281	0,003	1,193	0,123	1,380	0,014	0,983	0,786
1030713	COG0629L	gbs1758	ssb3	Cellular processess	DNA replication, recombination and repair	Single strand binding protein	0,986	0,834	1,070	0,243	1,039	0,691	1,036	0,657
1030730	COG0360J	gbs1759	rpsF	Cellular processess	Translation, ribosomal structure and biogenesis	SSU ribosomal protein S6P	1,256	0,004	1,093	0,208	1,318	0,007	0,925	0,242
1030720	COG1194L	gbs1760	mutY	Cellular processess	DNA replication, recombination and repair	A G-specific adenine DNA glycosylase (EC 3.2.2.-)	0,555	0,000	0,670	0,003	0,561	0,000	0,706	0,000
1030726	COG1396K	gbs1761		Cellular processess	Transcription	Transcriptional regulator	1,361	0,022	1,041	0,861	1,263	0,059	1,622	0,008
1030744	COG0526OC	gbs1762	trx	Metabolism and transport	Energy production and conversion	Thioredoxin	0,471	0,000	0,350	0,000	0,454	0,000	0,346	0,000
1031017	-	gbs1763		Metabolism and transport	Fatty acid and phospholipids	Phosphatidylglycerophosphatase B (EC 3.1.3.27)	0,248	0,000	0,524	0,000	0,257	0,000	0,393	0,000
1030736	COG1193L	gbs1764	mutS2	Cellular processess	DNA replication, recombination and repair	DNA mismatch repair protein mutS	0,222	0,000	0,352	0,000	0,170	0,000	0,404	0,000
1031828	-	gbs1765		General function predicted only		CvpA family membrane protein	0,626	0,001	0,752	0,007	0,527	0,000	0,644	0,014
1030758	-	gbs1766		Hypothetical		Hypothetical cytosolic protein	0,384	0,000	0,391	0,000	0,384	0,000	0,447	0,000
1031367	COG1039L	gbs1767		Cellular processess	Transcription	Ribonuclease HIII (EC 3.1.26.4)	0,167	0,000	0,281	0,000	0,158	0,000	0,301	0,000
1030794	COG0681U	gbs1768	spi	Cellular processess	Posttranslational modification, protein turnover, chaperones	Signal peptidase I (EC 3.4.21.89)	0,525	0,000	0,811	0,187	0,543	0,000	0,652	0,000
1030802	COG0507L	gbs1769	recD	Cellular processess	DNA replication, recombination and repair	Exodeoxyribonuclease V alpha chain (EC 3.1.11.5)	0,557	0,002	0,786	0,132	0,387	0,000	1,004	0,975
1030764	-	gbs1770		Hypothetical		Hypothetical protein	1,734	0,000	1,349	0,066	1,598	0,004	1,230	0,034
1031309	COG0389L	gbs1771	dinP	Cellular processess	DNA replication, recombination and repair	DNA polymerase IV (EC 2.7.7.7)	1,325	0,003	1,391	0,007	1,317	0,015	1,215	0,065
1030768	COG1882C	gbs1772	pfl	Metabolism and transport	Energy production and conversion	Formate acetyltransferase (EC 2.3.1.54)	2,683	0,000	9,861	0,000	2,885	0,000	12,010	0,000
1030831	-	gbs1773		General function predicted only		FMN-binding protein	1,722	0,054	10,260	0,000	2,129	0,021	9,979	0,000
1030769	COG1680V	gbs1774		Cellular processess	Toxin production and resistance	Beta-lactamase family protein	0,460	0,001	0,469	0,001	0,522	0,001	0,359	0,000
1030770	-	gbs1775		Hypothetical		Hypothetical cytosolic protein	1,006	0,957	0,591	0,004	0,919	0,504	0,556	0,001
1030773	-	gbs1776		Hypothetical		Hypothetical membrane spanning protein	0,459	0,000	0,448	0,001	0,384	0,000	0,334	0,000
1030776	-	gbs1777	glpF.2	Metabolism and transport	Carbohydrates, organic alcohols, and acids	Aquaporin Glycerol uptake facilitator protein	0,789	0,030	0,535	0,000	0,717	0,011	0,403	0,000
1030777	COG0589T	gbs1778		Cellular processess	Posttranslational modification, protein turnover, chaperones	Universal stress protein family	0,542	0,023	1,243	0,668	1,555	0,269	1,044	0,907
1031468	-	gbs1779	norA	Transport and binding proteins	Unknown substrate	Transporter, MFS superfamily	1,773	0,066	1,995	0,191	2,061	0,014	0,993	0,984
1030778	COG0664T	gbs1780	srv	Cellular processess	Transcription	Transcription regulator, crp family	0,813	0,095	0,423	0,000	0,651	0,000	0,558	0,000
1030789	COG2936R	gbs1781	pepXP	Metabolism and transport	Amino acids, peptides, aminosugars and amines	Xaa-Pro dipeptidyl-peptidase (EC 3.4.14.11)	0,560	0,000	0,637	0,000	0,543	0,000	0,771	0,004
1031410	-	gbs1782		Hypothetical		Hypothetical protein	0,190	0,000	0,237	0,000	0,182	0,000	0,408	0,000
1030790	COG0142H	gbs1783		Metabolism and transport	Cofactors, prosthetic groups, and carriers	Farnesyl pyrophosphate synthetase (EC 2.5.1.1) Geranyltranstransferase (EC 2.5.	0,413	0,000	0,496	0,000	0,392	0,000	0,569	0,000
1030803	COG4987CO	gbs1784		Metabolism and transport	Energy production and conversion	Transport ATP-binding protein cydC	0,060	0,000	0,418	0,000	0,064	0,000	0,630	0,025
1030795	COG4988CO	gbs1785		Metabolism and transport	Energy production and conversion	Transport ATP-binding protein cydD	0,292	0,000	0,508	0,015	0,184	0,000	0,896	0,349
1030801	COG1294C	gbs1786		Metabolism and transport	Energy production and conversion	Cytochrome d ubiquinol oxidase subunit II (EC 1.10.3.-)	0,368	0,000	0,708	0,001	0,367	0,000	0,809	0,025
1030701	COG1271C	gbs1787		Metabolism and transport	Energy production and conversion	Cytochrome d ubiquinol oxidase subunit I (EC 1.10.3.-)	0,452	0,000	0,894	0,142	0,455	0,000	0,985	0,835
1030807	COG1252C	gbs1788		Metabolism and transport	Energy production and conversion	NADH dehydrogenase family	0,525	0,000	0,778	0,017	0,445	0,000	0,838	0,063
1030761	COG1575H	gbs1789		Metabolism and transport	Cofactors, prosthetic groups, and carriers	1,4-dihydroxy-2-naphthoate polyprenyltransferase (EC 2.5.1.-)	0,504	0,000	0,886	0,225	0,456	0,000	0,995	0,957
1030847	-	gbs1790		Hypothetical		Hypothetical protein	10,459	0,000	9,566	0,000	12,332	0,000	7,127	0,002
1030848	COG0718S	gbs1791		Hypothetical		Hypothetical cytosolic protein	0,282	0,000	0,300	0,000	0,221	0,000	0,276	0,000
1030850	COG2230M	gbs1792		Cell Envelope	Biosynthesis and degradation of surface polysaccharides and lipopo	Cyclopropane-fatty-acyl-phospholipid synthase (EC 2.1.1.79)	0,888	0,280	0,652	0,016	0,967	0,752	0,499	0,001
1030851	COG0789K	gbs1793		Cellular processess	Transcription	Transcriptional regulator, MerR family	6,654	0,000	2,096	0,001	5,785	0,000	1,816	0,000
1030859	COG0847L	gbs1794	dnaQ	Cellular processess	DNA replication, recombination and repair	DNA polymerase III, epsilon chain (EC 2.7.7.7)	2,978	0,000	1,044	0,831	2,333	0,016	0,994	0,979
1030852	-	gbs1795		Hypothetical		Hypothetical cytosolic protein	0,438	0,000	0,501	0,000	0,376	0,000	0,620	0,000
1030856	COG2081R	gbs1796		General function predicted only		NAD(FAD)-utilizing dehydrogenases	0,761	0,095	0,698	0,030	0,681	0,009	0,658	0,010
1030860	COG1085C	gbs1797		Metabolism and transport	Energy production and conversion	Galactose-1-phosphate uridylyltransferase (EC 2.7.7.10)	0,885	0,402	1,228	0,197	0,775	0,224	0,958	0,651
1030855	COG0199J	gbs1798	rpsN2	Cellular processess	Translation, ribosomal structure and biogenesis	SSU ribosomal protein S14P	3,232	0,000	1,118	0,403	2,695	0,001	1,186	0,191
1030854	COG2008E	gbs1799		Metabolism and transport	Amino acids, peptides, aminosugars and amines	Low-specificity L-threonine aldolase (EC 4.1.2.5)	0,341	0,000	0,278	0,000	0,346	0,000	0,341	0,000
1030853	COG0533O	gbs1800		Cellular processess	Posttranslational modification, protein turnover, chaperones	O-sialoglycoprotein endopeptidase (EC 3.4.24.57)	0,410	0,000	0,483	0,003	0,354	0,000	0,586	0,000
1030862	COG0456R	gbs1801		Cellular processess	Translation, ribosomal structure and biogenesis	Ribosomal-protein-S18-alanine acetyltransferase (EC 2.3.1.128)	0,309	0,000	0,442	0,001	0,234	0,000	0,537	0,002
1030865	COG1214O	gbs1802		Cellular processess	Posttranslational modification, protein turnover, chaperones	Non-proteolytic protein, peptidase family M22	0,412	0,000	0,409	0,000	0,413	0,000	0,556	0,000
1030868	-	gbs1803		General function predicted only		Putative transcriptional regulator	0,682	0,002	0,600	0,000	0,500	0,000	0,790	0,006
1030867	COG0595R	gbs1804		General function predicted only		Zn-dependent hydrolase (EC 3.-.-.-)	0,678	0,002	0,583	0,000	0,548	0,000	0,790	0,029
1030861	COG3942R	gbs1805		General function predicted only		Secreted protein	0,155	0,000	0,057	0,000	0,198	0,000	0,085	0,000

1030872	-	gbs1806	glnA	Metabolism and transport	Amino acids, peptides, aminosugars and amines	Glutamine synthetase (EC 6.3.1.2)	1,313	0,051	1,301	0,001	1,405	0,053	1,883	0,000
1030864	COG0789K	gbs1807		Cellular processess	Transcription	Transcriptional regulator, MerR family	4,045	0,007	2,845	0,000	4,207	0,002	3,524	0,000
1030866	COG4129S	gbs1808		Hypothetical		Hypothetical protein	0,738	0,013	0,863	0,230	0,914	0,461	1,125	0,567
1030870	-	gbs1809	pgk	Metabolism and transport	Carbohydrates, organic alcohols, and acids	Phosphoglycerate kinase (EC 2.7.2.3)	1,642	0,000	1,700	0,000	1,954	0,000	1,874	0,000
1030873	COG2503R	gbs1810	lppC	Metabolism and transport	Cofactors, prosthetic groups, and carriers	Acid phosphatase (EC 3.1.3.2)	2,464	0,000	3,485	0,000	3,660	0,000	3,750	0,000
1030869	COG0057G	gbs1811	gapC	Metabolism and transport	Carbohydrates, organic alcohols, and acids	Glyceraldehyde 3-phosphate dehydrogenase (EC 1.2.1.12) GAPDH	3,129	0,000	3,062	0,000	3,830	0,000	3,143	0,000
1030871	COG0480J	gbs1812	fus	Cellular processess	Translation, ribosomal structure and biogenesis	Protein Translation Elongation Factor G (EF-G)	0,977	0,658	0,930	0,139	0,986	0,839	0,898	0,065
1030883	COG0049J	gbs1813	rpsG	Cellular processess	Translation, ribosomal structure and biogenesis	SSU ribosomal protein S7P	0,539	0,000	0,691	0,000	0,590	0,000	0,663	0,000
1030884	-	gbs1814	rpsL	Cellular processess	Translation, ribosomal structure and biogenesis	SSU ribosomal protein S12P	0,654	0,000	0,949	0,564	0,624	0,000	0,986	0,887
1030890	COG0503F	gbs1815	purR	Metabolism and transport	Purines, pyrimidines, nucleosides, and nucleotides	Pur operon repressor	1,368	0,002	0,694	0,000	1,251	0,016	0,722	0,000
1030876	COG3481R	gbs1816	cbf	General function predicted only		CMP-binding factor	0,398	0,000	0,475	0,000	0,368	0,000	0,519	0,000
1030896	COG1322S	gbs1817		General function predicted only		RmuC family protein	0,231	0,000	0,378	0,000	0,218	0,000	0,479	0,000
1030891	COG1564H	gbs1818		Metabolism and transport	Cofactors, prosthetic groups, and carriers	Thiamin pyrophosphokinase (EC 2.7.6.2)	0,220	0,000	0,443	0,000	0,237	0,000	0,470	0,000
1030894	COG0036G	gbs1819	rpe	Metabolism and transport	Carbohydrates, organic alcohols, and acids	Ribulose-phosphate 3-epimerase (EC 5.1.3.1)	0,383	0,000	0,554	0,000	0,360	0,000	0,722	0,006
1030900	COG1162R	gbs1820		General function predicted only		GTPase (EC 3.6.1.-)	0,228	0,000	0,333	0,000	0,223	0,000	0,415	0,000
1030895	-	gbs1821		Cellular processess	Translation, ribosomal structure and biogenesis	23S rRNA m(1)G 745 methyltransferase (EC 2.1.1.51)	0,152	0,000	0,268	0,000	0,145	0,000	0,251	0,000
1030893	COG0030J	gbs1822	ksgA	Cellular processess	Translation, ribosomal structure and biogenesis	Dimethyladenosine transferase (EC 2.1.1.-)	0,300	0,000	0,426	0,000	0,276	0,000	0,409	0,000
1030892	COG0537FGI	gbs1823		General function predicted only		HIT family hydrolase	0,303	0,000	0,438	0,000	0,314	0,000	0,475	0,000
1030897	COG1658L	gbs1824		Cellular processess	Transcription	Ribonuclease M5 (EC 3.1.26.8)	0,579	0,000	0,753	0,038	0,537	0,000	0,780	0,010
1030898	COG0084L	gbs1825	tatD	Cellular processess	DNA replication, recombination and repair	DNase, TatD family (EC 3.1.-.-)	0,383	0,000	0,502	0,000	0,382	0,000	0,545	0,000
1030899	-	gbs1826		Hypothetical		Hypothetical protein	0,426	0,000	0,287	0,000	0,374	0,000	0,356	0,000
1030909	-	gbs1827		Hypothetical		Hypothetical protein	0,385	0,000	0,228	0,000	0,302	0,000	0,257	0,000
1030903	-	gbs1828		Hypothetical		Hypothetical cytosolic protein	0,608	0,000	0,272	0,000	0,572	0,000	0,369	0,000
1030902	-	gbs1829		Hypothetical		Hypothetical secreted protein	1,048	0,699	0,307	0,000	0,864	0,288	0,388	0,000
1030904	COG3966M	gbs1830	dltD	General function predicted only		Protein dltD precursor	0,491	0,000	0,409	0,000	0,430	0,000	0,539	0,000
1030910	COG0236IQ	gbs1831		Metabolism and transport	Fatty acid and phospholipids	D-alanyl carrier protein	0,653	0,001	0,372	0,000	0,595	0,000	0,491	0,000
1030913	COG1696M	gbs1832		General function predicted only		Protein dltB	0,539	0,000	0,382	0,000	0,490	0,000	0,466	0,000
1030911	COG1020Q	gbs1833	dltA	Metabolism and transport	Secondary metabolites	D-alanine-activating enzyme (EC 6.3.2.-)	0,644	0,000	0,457	0,000	0,596	0,000	0,599	0,000
1030917	COG0642T	gbs1834		Cellular processess	Signal transduction	Sensory transduction protein kinase (EC 2.7.3.-)	0,437	0,000	0,367	0,000	0,405	0,000	0,496	0,000
1030914	COG0745TK	gbs1835		Cellular processess	Transcription	Transcriptional regulatory protein	0,570	0,000	0,394	0,000	0,542	0,000	0,508	0,000
1030915	-	gbs1836	rpmH	Cellular processess	Translation, ribosomal structure and biogenesis	LSU ribosomal protein L34P	1,178	0,136	0,799	0,127	0,990	0,930	0,696	0,001
1030912	COG3314S	gbs1837		Hypothetical		Hypothetical protein	0,297	0,000	0,287	0,000	0,276	0,000	0,279	0,000
1030922	COG2113E, C	gbs1838	opuABC	Metabolism and transport	Amino acids, peptides, aminosugars and amines	Glycine betaine-binding protein Glycine betaine transport system permease protein	0,392	0,002	0,338	0,001	0,362	0,002	0,433	0,003
1030926	COG4175E	gbs1839	opuAA	Metabolism and transport	Amino acids, peptides, aminosugars and amines	Glycine betaine transport ATP-binding protein	0,425	0,007	0,379	0,004	0,390	0,005	0,509	0,014
1030923	COG3957G	gbs1840		Metabolism and transport	Carbohydrates, organic alcohols, and acids	Xylulose-5-phosphate (EC 4.1.2.9) Fructose-6-phosphate phosphoketolase (EC 4.1	2,346	0,043	20,152	0,005	3,823	0,008	12,075	0,007
1030916	COG2220R	gbs1841		Cellular processess	Toxin production and resistance	Metal-dependent hydrolase (EC 3.-.-.-)	4,712	0,003	29,024	0,015	6,548	0,010	24,279	0,023
1030928	COG3711K	gbs1842		Cellular processess	Transcription	Transcription antiterminator, BglG family	0,727	0,008	1,249	0,071	0,683	0,002	1,266	0,055
1030929	-	gbs1843		General function predicted only		protein with aldose 1-epimerase motif involved in 2-ketogluconate utilization	4,934	0,039	24,112	0,018	8,439	0,008	11,236	0,010
1030924	COG1070G	gbs1844		Metabolism and transport	Carbohydrates, organic alcohols, and acids	L-xylulokinase (EC 2.7.1.53)	2,548	0,124	12,724	0,030	2,897	0,001	12,328	0,011
1030919	COG2159R	gbs1845		Metabolism and transport	Purines, pyrimidines, nucleosides, and nucleotides	Amidohydrolase	2,041	0,303	3,724	0,003	2,274	0,102	4,999	0,001
1030921	COG3775G	gbs1846		Metabolism and transport	Carbohydrates, organic alcohols, and acids	PTS system, galactitol-specific IIC component (EC 2.7.1.69)	1,800	0,077	5,976	0,009	2,853	0,161	6,418	0,104
1030932	COG1052CHI	gbs1847		Metabolism and transport	Central intermediary metabolism	Glyoxylate reductase (NADP+) (EC 1.1.1.79) Glyoxylate reductase (NAD+) (EC 1.1	1,333	0,204	1,801	0,125	1,183	0,483	1,981	0,031
1030931	-	gbs1848		Hypothetical		Hypothetical protein	1,217	0,331	1,897	0,079	0,881	0,608	2,024	0,002
1030925	COG1609K	gbs1849		Cellular processess	Transcription	Kdg operon repressor	0,824	0,275	0,852	0,561	0,704	0,109	0,436	0,004
1030936	-	gbs1850		Metabolism and transport	Energy production and conversion	Transaldolase (EC 2.2.1.2)	2,047	0,015	16,807	0,011	2,544	0,039	11,639	0,038
1030939	COG0235G	gbs1851	araD	Metabolism and transport	Carbohydrates, organic alcohols, and acids	L-ribulose-5-phosphate 4-epimerase (EC 5.1.3.4)	1,778	0,034	15,974	0,009	3,310	0,008	11,109	0,040
1030935	COG3623G	gbs1852		Metabolism and transport	Carbohydrates, organic alcohols, and acids	L-xylulose 5-phosphate 3-epimerase (EC 5.3.1.-)	2,369	0,008	14,573	0,008	3,142	0,003	10,564	0,024
1030930	COG0269G	gbs1853		Metabolism and transport	Carbohydrates, organic alcohols, and acids	3-keto-L-gulonate-6-phosphate decarboxylase (EC 4.1.1.-)	1,808	0,022	12,834	0,014	2,525	0,009	9,459	0,032
1030937	COG1762GT	gbs1854		Metabolism and transport	Carbohydrates, organic alcohols, and acids	PTS system, 3-keto-L-gulonate specific IIA component (EC 2.7.1.69)	1,600	0,197	12,614	0,016	2,796	0,023	8,189	0,042
1030933	COG3414G	gbs1855		Metabolism and transport	Carbohydrates, organic alcohols, and acids	PTS system, 3-keto-L-gulonate specific IIB component (EC 2.7.1.69)	3,490	0,010	18,016	0,013	5,672	0,011	11,141	0,050
1030941	-	gbs1856	ulaA	Metabolism and transport	Carbohydrates, organic alcohols, and acids	PTS system, 3-keto-L-gulonate specific IIC component (EC 2.7.1.69)	3,566	0,008	18,021	0,023	6,193	0,005	11,617	0,070
1030938	-	gbs1857		Hypothetical		Hypothetical membrane associated protein	0,799	0,051	0,647	0,002	0,668	0,002	0,772	0,024
1030943	-	gbs1858		Hypothetical		Hypothetical protein	0,438	0,001	0,822	0,237	0,633	0,078	0,496	0,000
1030945	-	gbs1859	purA	Metabolism and transport	Purines, pyrimidines, nucleosides, and nucleotides	Adenylosuccinate synthetase (EC 6.3.4.4)	1,078	0,336	0,624	0,000	0,980	0,809	0,621	0,000
1030942	COG1299G	gbs1860	sloR	Cellular processess	Transcription	Transcriptional regulator pfoR	0,549	0,000	0,418	0,002	0,502	0,004	0,508	0,000
1030940	COG2964S	gbs1861		General function predicted only		Putative DNA-binding protein	1,091	0,510	1,229	0,205	1,275	0,064	1,055	0,669
1030947	COG1181M, (	gbs1862		Metabolism and transport	Cofactors, prosthetic groups, and carriers	Amino acid ligase family protein (putative polyamide biosynthesis enzyme)	0,303	0,000	0,099	0,000	0,268	0,000	0,091	0,000
1030950	-	gbs1863		Hypothetical		Hypothetical membrane associated protein	0,456	0,000	0,209	0,000	0,375	0,000	0,420	0,000
1030944	COG3853P	gbs1864		Metabolism and transport	Inorganic ion transport and metabolism	Tellurite resistance protein	0,717	0,012	0,576	0,010	0,623	0,001	0,844	0,048
1030949	-	gbs1865	hslO	Cellular processess	Posttranslational modification, protein turnover, chaperones	33 kDa chaperonin	0,206	0,000	0,220	0,000	0,217	0,000	0,270	0,000
1030948	COG0042J	gbs1866		Cellular processess	Translation, ribosomal structure and biogenesis	tRNA-dihydrouridine synthase	0,517	0,001	0,595	0,006	0,487	0,001	0,633	0,005
1030951	COG1428F	gbs1867		Metabolism and transport	Purines, pyrimidines, nucleosides, and nucleotides	Deoxyadenosine kinase (EC 2.7.1.76) Deoxyguanosine kinase (EC 2.7.1.113)	0,328	0,000	0,637	0,040	0,248	0,000	0,398	0,001
1030946	COG1247M	gbs1868		Metabolism and transport	Fatty acid and phospholipids	Phosphinothricin N-acetyltransferase (EC 2.3.1.-)	0,214	0,000	0,348	0,000	0,211	0,000	0,250	0,000
1030952	COG0542O	gbs1869		Cellular processess	Transcription	Negative regulator of genetic competence clpC mecB	0,511	0,000	0,554	0,000	0,455	0,000	0,682	0,002
1030958	-	gbs1870	ctsR	Cellular processess	Transcription	Transcriptional regulator ctsR	0,326	0,001	0,407	0,001	0,319	0,001	0,515	0,005
1030955	-	gbs1871		Cellular processess	Toxin production and resistance	Small multidrug export protein	0,507	0,000	0,250	0,000	0,427	0,000	0,280	0,000
1030953	-	gbs1872	tsf	Cellular processess	Translation, ribosomal structure and biogenesis	Protein Translation Elongation Factor Ts (EF-Ts)	0,596	0,000	0,739	0,021	0,692	0,000	0,767	0,001
1030963	-	gbs1873	rpsB	Cellular processess	Translation, ribosomal structure and biogenesis	SSU ribosomal protein S2P	0,529	0,000	0,576	0,000	0,517	0,000	0,584	0,000
1030961	COG0450O	gbs1874	ahpC	Metabolism and transport	Energy production and conversion	Peroxiredoxin (EC 1.11.1.15)	1,476	0,003	1,968	0,000	1,552	0,001	1,830	0,000
1030959	COG3634O	gbs1875	ahpF	Metabolism and transport	Energy production and conversion	Peroxiredoxin reductase (NAD(P)H) (EC 1.6.4.-) NADH oxidase H2O2-forming (EC	1,132	0,296	1,696	0,000	1,153	0,186	1,740	0,000
1030954	COG0025P	gbs1876		Metabolism and transport	Inorganic ion transport and metabolism	Na+ H+ antiporter nhaP	0,269	0,000	0,471	0,020	0,320	0,000	0,398	0,006
1030965	-	gbs1877		Hypothetical		Hypothetical protein	0,298	0,000	0,356	0,000	0,262	0,000	0,339	0,000
1030964	COG1619V	gbs1878	mccF	Cell Envelope	Biosynthesis and degradation of murein sacculus and peptidoglycar	Muramoyltetrapeptide carboxypeptidase (EC 3.4.17.13)	4,188	0,000	1,178	0,286	3,951	0,000	0,811	0,257
1030971	COG3590O	gbs1879	pepO	Cellular processess	Posttranslational modification, protein turnover, chaperones	Oligoendopeptidase O (EC 3.4.24.-)	0,750	0,026	0,494	0,000	0,609	0,000	0,443	0,000
1030970	COG0673R	gbs1880		Metabolism and transport	Central intermediary metabolism	NAD-dependent oxidoreductase	1,272	0,035	0,841	0,265	1,179	0,269	0,652	0,009
1030967	-	gbs1881		Transport and binding proteins	Unknown substrate	Transporter, MFS superfamily	0,840	0,269	1,856	0,008	0,709	0,133	2,026	0,000
1030962	COG0664T	gbs1882		Cellular processess	Signal transduction	Catabolite gene activator	0,529	0,004	0,498	0,002	0,539	0,011	0,513	0,003
1030973	-	gbs1883	def	Cellular processess	Posttranslational modification, protein turnover, chaperones	Peptide deformylase (EC 3.5.1.88)	0,703	0,001	0,533	0,000	0,635	0,000	0,625	0,000
1030969	COG1609K	gbs1884	regR	Cellular processess	Transcription	Transcriptional regulator, LacI family	0,646	0,000	0,751	0,066	0,606	0,001	1,004	0,974
1030968	-	gbs1885		Hypothetical		Oligohyaluronate lyase (EC 4.2.2.-)	3,211	0,000	5,214	0,001	2,728	0,149	4,196	0,018
1030976	-	gbs1886	agaD	Metabolism and transport	Carbohydrates, organic alcohols, and acids	PTS system, N-acetylgalactosamine-specific IID component (EC 2.7.1.69)	1,874	0,000	4,763	0,016	2,407	0,028	5,796	0,000
1030974	-	gbs1887		Metabolism and transport	Carbohydrates, organic alcohols, and acids	PTS system, N-acetylgalactosamine-specific IIC component (EC 2.7.1.69)	2,470	0,011	5,942	0,001	2,906	0,018	10,582	0,000
1030980	-	gbs1888	agaV	Metabolism and transport	Carbohydrates, organic alcohols, and acids	PTS system, N-acetylgalactosamine-specific IIB component (EC 2.7.1.69)	2,052	0,004	3,480	0,001	3,690	0,011	7,987	0,008
1030972	COG4225R	gbs1889		General function predicted only		Unsaturated glucuronyl hydrolase (EC 3.2.1.-)	2,901	0,010	11,198	0,002	2,887	0,007	10,718	0,009
1030988	COG2893G	gbs1890		Metabolism and transport	Carbohydrates, organic alcohols, and acids	PTS system, N-acetylgalactosamine-specific IIA component (EC 2.7.1.69)	2,111	0,061	5,194	0,030	4,192	0,003	6,938	0,001
1030993	COG1028IQR	gbs1891	idnO	Metabolism and transport	Fatty acid and phospholipids	2-deoxy-D-gluconate 3-dehydrogenase (EC 1.1.1.125)	3,645	0,078	6,147	0,004	4,154	0,015	6,369	0,000
1030995	COG0698G	gbs1892		Metabolism and transport	Carbohydrates, organic alcohols, and acids	Galactose-6-phosphate isomerase LacB subunit (EC 5.3.1.26)	4,672	0,028	9,100	0,020	7,607	0,011	16,076	0,000
1030975	COG0524G	gbs1893		Metabolism and transport	Carbohydrates, organic alcohols, and acids	2-dehydro-3-deoxygluconokinase (EC 2.7.1.45)	6,351	0,009	12,000	0,016	7,534	0,000	18,057	0,001

1031009	COG0800G	gbs1894	kgdA	Metabolism and transport	Carbohydrates, organic alcohols, and acids	4-Hydroxy-2-oxoglutarate aldolase (EC 4.1.3.16) 2-dehydro-3-deoxyphosphogluco	2,897	0,087	8,620	0,002	4,549	0,056	7,078	0,022
1031001	-	gbs1895		Hypothetical		Hypothetical protein	2,493	0,000	2,047	0,025	2,416	0,015	1,905	0,000
1031005	COG3560R	gbs1896		General function predicted only		Nitroreductase family protein	2,538	0,258	1,109	0,726	2,150	0,267	1,000	1,000
1031010	COG1846K	gbs1897		Cellular processess	Transcription	Transcriptional regulator, MarR family	0,308	0,000	0,410	0,002	0,364	0,000	0,484	0,001
1031006	COG2176L	gbs1898	polC	Cellular processess	DNA replication, recombination and repair	DNA polymerase III alpha subunit (EC 2.7.7.7)	0,551	0,000	0,768	0,046	0,519	0,000	0,919	0,415
1031003	COG1705NU	gbs1899	mur1.2	Cell Envelope	Biosynthesis and degradation of murein sacculus and peptidoglycan	N-acetylmuramidase (EC 3.2.1.17)	0,927	0,661	0,560	0,054	0,731	0,062	0,357	0,001
1031002	COG0442J	gbs1900	proS	Cellular processess	Translation, ribosomal structure and biogenesis	Prolyl-tRNA synthetase (EC 6.1.1.15)	0,425	0,000	0,665	0,003	0,412	0,000	0,670	0,001
1031007	COG0750M	gbs1901		Cellular processess	Posttranslational modification, protein turnover, chaperones	Membrane endopeptidase, M50 family	0,460	0,000	0,539	0,001	0,455	0,000	0,746	0,018
1031014	COG0575I	gbs1902	cdsA	Metabolism and transport	Fatty acid and phospholipids	Phosphatidate cytidylyltransferase (EC 2.7.7.41)	0,329	0,000	0,477	0,000	0,323	0,000	0,515	0,000
1031008	COG0020I	gbs1903	uppS	Metabolism and transport	Fatty acid and phospholipids	Undecaprenyl pyrophosphate synthetase (EC 2.5.1.31)	0,464	0,000	0,433	0,000	0,440	0,000	0,469	0,000
1031015	COG1862U	gbs1904	yajC	Cellular processess	Posttranslational modification, protein turnover, chaperones	Protein translocase subunit YajC	1,510	0,000	0,640	0,000	1,354	0,002	0,608	0,000
1031019	-	gbs1905		Metabolism and transport	Energy production and conversion	Thioredoxin	0,610	0,000	0,367	0,000	0,482	0,000	0,333	0,000
1031021	COG0281C	gbs1906		Metabolism and transport	Energy production and conversion	NAD-dependent malic enzyme (EC 1.1.1.39)	3,890	0,000	7,846	0,021	4,456	0,001	5,615	0,000
1031011	-	gbs1907	malP	Metabolism and transport	Carbohydrates, organic alcohols, and acids	Malate-sodium symport	5,503	0,076	7,365	0,211	2,663	0,053	2,712	0,026
1031022	COG3290T	gbs1908	dpiB	Cellular processess	Signal transduction	Sensor kinase dpiB (EC 2.7.3.-)	0,822	0,345	0,877	0,457	0,582	0,013	1,184	0,127
1031023	COG4565KT	gbs1909	dpiA	Cellular processess	Transcription	Transcriptional regulatory protein	0,873	0,214	1,155	0,602	0,846	0,265	1,556	0,040
1031020	COG1087M	gbs1910		Metabolism and transport	Energy production and conversion	UDP-glucose 4-epimerase (EC 5.1.3.2)	1,087	0,290	1,067	0,464	1,139	0,137	1,369	0,014
1031027	-	gbs1911	dexB	Metabolism and transport	Carbohydrates, organic alcohols, and acids	Glucan 1,6-alpha-glucosidase (EC 3.2.1.70)	2,229	0,000	2,318	0,000	1,868	0,006	3,224	0,000
1031029	COG3839G	gbs1912	msmK	Metabolism and transport	Carbohydrates, organic alcohols, and acids	Multiple sugar transport ATP-binding protein msmK	1,377	0,083	2,325	0,002	1,561	0,012	2,945	0,002
1031025	COG2508TQ	gbs1913	lrp	Cellular processess	Signal transduction	Leucine rich protein	0,550	0,000	0,698	0,023	0,688	0,016	0,803	0,029
1031024	COG2017G	gbs1914		Metabolism and transport	Carbohydrates, organic alcohols, and acids	Aldose 1-epimerase family protein	1,259	0,293	2,802	0,031	1,861	0,083	1,687	0,157
1031035	COG3684G	gbs1915		Metabolism and transport	Carbohydrates, organic alcohols, and acids	Tagatose-bisphosphate aldolase (EC 4.1.2.40)	3,231	0,001	6,162	0,000	3,596	0,000	5,436	0,001
1031028	COG1105G	gbs1916		Metabolism and transport	Carbohydrates, organic alcohols, and acids	Tagatose-6-phosphate kinase (EC 2.7.1.144)	0,946	0,841	3,398	0,175	0,971	0,918	1,778	0,094
1031032	COG0698G	gbs1917		Metabolism and transport	Carbohydrates, organic alcohols, and acids	Galactose-6-phosphate isomerase lacB subunit (EC 5.3.1.26)	2,157	0,012	3,994	0,005	2,234	0,032	3,761	0,005
1031048	COG0698G	gbs1918	lacA.1	Metabolism and transport	Carbohydrates, organic alcohols, and acids	Galactose-6-phosphate isomerase lacA subunit (EC 5.3.1.26)	1,158	0,704	1,206	0,570	1,008	0,977	0,926	0,803
1031043	COG4409G	gbs1919		Metabolism and transport	Carbohydrates, organic alcohols, and acids	Sialidase A precursor (EC 3.2.1.18)	0,704	0,025	1,256	0,280	0,757	0,125	1,532	0,032
1031030	COG3775G	gbs1920		Metabolism and transport	Carbohydrates, organic alcohols, and acids	PTS system, galactose-specific IIC component (EC 2.7.1.69)	1,947	0,017	5,104	0,020	2,474	0,039	5,905	0,000
1031054	COG3414G	gbs1921		Metabolism and transport	Carbohydrates, organic alcohols, and acids	PTS system, galactose-specific IIB component (EC 2.7.1.69)	2,381	0,003	3,980	0,287	2,743	0,034	3,628	0,047
1031044	COG1762GT	gbs1922		Metabolism and transport	Carbohydrates, organic alcohols, and acids	PTS system, galactose-specific IIA component (EC 2.7.1.69)	3,055	0,000	6,813	0,061	3,689	0,000	5,481	0,002
1031037	COG1349KG	gbs1923	lacR.1	Cellular processess	Transcription	Lactose phosphotransferase system repressor	1,758	0,000	1,225	0,248	1,625	0,001	0,992	0,950
1031057	-	gbs1925		Mobile and extrachromosomal elements		Streptococcal histidine triad protein	1,053	0,801	2,024	0,194	1,477	0,079	1,855	0,045
1031045	COG0803P	gbs1926		Metabolism and transport	Inorganic ion transport and metabolism	Laminin-binding surface protein	1,519	0,334	1,396	0,267	1,036	0,914	1,404	0,300
1031062	-	gbs1927		Cellular processess	Translation, ribosomal structure and biogenesis	D-tyrosyl-tRNA(Tyr) deacylase (EC 3.1.-.-)	0,354	0,000	0,526	0,001	0,402	0,000	0,402	0,000
1031064	COG0317TK	gbs1928	relA	Cellular processess	Signal transduction	GTP pyrophosphokinase (EC 2.7.6.5) Guanosine-3 ,5 -bis(Diphosphate) 3 -pyroph	0,539	0,000	0,577	0,004	0,398	0,000	0,754	0,035
1031058	COG0737F	gbs1929		Metabolism and transport	Purines, pyrimidines, nucleosides, and nucleotides	LPXTG 2 ,3 -cyclic-nucleotide 2 -phosphodiesterase (EC 3.1.4.16) 3 -nucleotidase	0,722	0,125	0,503	0,015	0,815	0,286	0,516	0,017
1031061	COG1780F	gbs1930		Metabolism and transport	Purines, pyrimidines, nucleosides, and nucleotides	NrdI protein	0,854	0,102	0,385	0,000	0,725	0,008	0,479	0,000
1031076	COG1363G	gbs1931		Metabolism and transport	Carbohydrates, organic alcohols, and acids	Deblocking aminopeptidase (EC 3.4.11.-)	0,484	0,000	0,315	0,000	0,504	0,001	0,326	0,000
1031063	-	gbs1932		General function predicted only		Putative kinase	0,467	0,000	0,380	0,000	0,491	0,000	0,323	0,000
1031060	COG1840P	gbs1933		Metabolism and transport	Inorganic ion transport and metabolism	Iron(III)-binding protein	0,914	0,541	3,445	0,004	0,709	0,029	2,484	0,002
1031078	COG4753T	gbs1934		Cellular processess	Signal transduction	Two-component response regulator yesN	0,122	0,000	3,253	0,004	0,194	0,000	1,821	0,059
1031079	COG2972T	gbs1935		Cellular processess	Signal transduction	Two-component sensor kinase yesM (EC 2.7.3.-)	0,391	0,001	4,135	0,002	0,458	0,004	3,110	0,001
1031074	-	gbs1936	ptsD	Metabolism and transport	Carbohydrates, organic alcohols, and acids	PTS system, mannose fructose family IID component	2,895	0,004	48,342	0,001	3,312	0,002	29,910	0,000
1031065	-	gbs1937	ptsC	Metabolism and transport	Carbohydrates, organic alcohols, and acids	PTS system, mannose fructose family IIC component	4,262	0,040	133,435	0,000	5,514	0,032	84,117	0,001
1031081	-	gbs1938	ptsB	Metabolism and transport	Carbohydrates, organic alcohols, and acids	PTS system, mannose fructose family IIB component	8,976	0,003	138,515	0,000	11,740	0,000	90,729	0,000
1031080	COG2893G	gbs1939		Metabolism and transport	Carbohydrates, organic alcohols, and acids	PTS system, mannose fructose family IIA component	10,461	0,000	99,897	0,000	8,965	0,000	65,303	0,000
1031082	-	gbs1940		Hypothetical		Hypothetical membrane spanning protein	0,419	0,040	1,309	0,591	0,428	0,037	0,425	0,038
1031075	-	gbs1941		Transport and binding proteins	Unknown substrate	Transporter	0,526	0,001	0,917	0,435	0,521	0,001	0,915	0,497
1031084	COG1131V	gbs1942		Transport and binding proteins	Unknown substrate	ABC transporter ATP-binding protein	1,149	0,535	0,487	0,024	1,032	0,884	0,790	0,070
1031085	COG2972T	gbs1943	fasB	hypothetical		Hypothetical protein	0,503	0,002	0,523	0,006	0,554	0,002	0,450	0,001
1031083	COG3279KT	gbs1944	fasA	Cellular processess	Transcription	Response regulator FasA	0,597	0,003	0,863	0,497	0,611	0,000	0,454	0,000
1031089	COG3568R	gbs1945		General function predicted only		Endonuclease Exonuclease phosphatase family protein	2,060	0,102	7,293	0,002	2,618	0,053	9,461	0,000
1031087	COG1263G	gbs1946		Metabolism and transport	Carbohydrates, organic alcohols, and acids	PTS system, glucose-specific IIABC component (EC 2.7.1.69)	3,697	0,000	5,903	0,000	3,758	0,001	8,466	0,000
1031091	COG0642T	gbs1947		Cellular processess	Signal transduction	Phosphate regulon sensor protein phoR (EC 2.7.3.-)	0,703	0,004	0,693	0,043	0,724	0,008	0,968	0,761
1031090	COG0745TK	gbs1948		Cellular processess	Signal transduction	Alkaline phosphatase synthesis two-component response regulator phoP	1,096	0,492	0,989	0,957	0,951	0,706	0,982	0,850
1031086	COG0704P	gbs1949		Metabolism and transport	Inorganic ion transport and metabolism	Phosphate transport system protein phoU	0,672	0,059	0,758	0,132	0,912	0,864	0,917	0,799
1031103	COG1117P	gbs1950		Metabolism and transport	Inorganic ion transport and metabolism	Phosphate transport ATP-binding protein pstB	1,043	0,921	1,802	0,062	0,997	0,993	1,602	0,078
1031094	COG0581P	gbs1951		Metabolism and transport	Inorganic ion transport and metabolism	Phosphate transport system permease protein pstA	1,191	0,511	4,880	0,024	1,601	0,074	2,451	0,029
1031092	COG0573P	gbs1952		Metabolism and transport	Inorganic ion transport and metabolism	Phosphate transport system permease protein pstC	1,479	0,293	7,531	0,065	2,744	0,109	3,852	0,013
1031095	COG0226P	gbs1953		Metabolism and transport	Inorganic ion transport and metabolism	probable hemolysin precursor	2,773	0,006	2,825	0,050	3,466	0,002	4,347	0,021
1031108	-	gbs1954		Hypothetical		Hypothetical protein	0,412	0,000	0,774	0,091	0,474	0,000	0,790	0,132
1031100	COG1385S	gbs1955		Hypothetical		Hypothetical cytosolic protein	0,214	0,000	0,220	0,000	0,321	0,000	0,536	0,000
1031096	COG2264J	gbs1956		Cellular processess	Translation, ribosomal structure and biogenesis	Ribosomal protein L11 methyltransferase (EC 2.1.1.-)	1,032	0,809	1,482	0,008	1,225	0,126	1,457	0,094
1031099	COG4815S	gbs1957		Hypothetical		Hypothetical cytosolic protein	0,693	0,006	1,036	0,715	0,645	0,014	0,855	0,069
1031111	COG0789K	gbs1958		Cellular processess	Transcription	Transcriptional activator tipA	5,018	0,000	1,960	0,008	4,882	0,000	1,382	0,005
1031101	-	gbs1959		Metabolism and transport	Central intermediary metabolism	Acetyltransferase (EC 2.3.1.-)	1,353	0,040	0,920	0,423	1,316	0,024	1,077	0,377
1031110	COG0494LR	gbs1960		Cellular processess	DNA replication, recombination and repair	Phosphohydrolase (MutT nudix family protein)	1,533	0,002	1,102	0,322	1,650	0,025	1,289	0,006
1031106	-	gbs1961		Hypothetical		Hypothetical protein	0,817	0,077	0,562	0,000	0,739	0,024	0,620	0,000
1031114	-	gbs1962		Hypothetical		Hypothetical protein	0,863	0,080	0,697	0,070	0,780	0,002	0,912	0,434
1031107	-	gbs1963		Metabolism and transport	Central intermediary metabolism	Acetyltransferase, GNAT family	0,934	0,416	1,177	0,245	0,880	0,285	1,024	0,806
1031118	COG2256L	gbs1964		Cellular processess	DNA replication, recombination and repair	ATPase, AAA family	0,345	0,000	0,328	0,000	0,258	0,000	0,350	0,000
1031109	-	gbs1965		Hypothetical		Hypothetical protein	0,976	0,955	0,923	0,808	0,623	0,229	0,984	0,960
1031119	-	gbs1966		Hypothetical		Hypothetical protein	1,096	0,760	0,766	0,457	0,904	0,742	0,634	0,237
1031129	-	gbs1967		Hypothetical		Hypothetical cytosolic protein	2,080	0,001	1,575	0,017	1,928	0,003	1,403	0,066
1031122	-	gbs1968		Hypothetical		Hypothetical protein	3,811	0,000	1,033	0,919	3,253	0,000	1,249	0,430
1031130	-	gbs1969		Mobile and extrachromosomal elements		DNA integration recombination inversion protein	1,834	0,005	1,949	0,082	0,791	0,456	1,384	0,444
1031133	-	gbs1970		Hypothetical		Hypothetical protein	1,002	0,998	1,415	0,599	0,944	0,927	1,106	0,840
1031135	-	gbs1971		Hypothetical		Hypothetical protein	2,000	0,114	1,504	0,449	2,471	0,054	2,319	0,092
1031136	COG1396K	gbs1972		Cellular processess	Transcription	Transcriptional regulator, Cro CI family	1,345	0,023	0,508	0,001	1,081	0,600	0,434	0,000
1031128	-	gbs1973		Hypothetical		Hypothetical protein	0,500	0,000	1,073	0,734	0,553	0,000	0,975	0,816
1031142	-	gbs1974		Hypothetical		Hypothetical protein	0,687	0,032	0,823	0,507	0,818	0,353	0,374	0,001
1031140	COG2378K	gbs1975		Cellular processess	Transcription	Transcriptional regulator, DeoR family	1,286	0,340	0,908	0,779	1,025	0,929	1,497	0,133
1031143	-	gbs1976		Hypothetical		Hypothetical protein	6,356	0,000	1,580	0,164	4,813	0,000	1,588	0,086
1031137	-	gbs1977		Hypothetical		Hypothetical protein	0,935	0,735	1,160	0,534	0,990	0,963	1,194	0,700
1031131	-	gbs1978		Hypothetical		Hypothetical protein	1,845	0,003	1,786	0,118	1,803	0,022	1,007	0,973
1031134	COG4842S	gbs1979		Cellular processess	Posttranslational modification, protein turnover, chaperones	a small heat shock protein of Clostridium acetobutylicum homolog lmo0056	3,380	0,118	4,676	0,044	6,441	0,075	3,279	0,249
1031144	-	gbs1980		Hypothetical		Hypothetical secreted protein	1,628	0,004	0,835	0,565	1,587	0,050	1,023	0,937
1031178	-	gbs1981		Hypothetical		Hypothetical protein	2,313	0,003	1,712	0,005	2,087	0,024	2,124	0,000
1031222	COG3077L	gbs1982		Cellular processess	DNA replication, recombination and repair	DNA-damage-inducible protein J	4,036	0,024	5,272	0,021	1,898	0,080	6,327	0,069

1031139	-	gbs1983		Hypothetical		Hypothetical protein	0,418	0,000	0,434	0,011	0,363	0,000	0,401	0,021
1031221	-	gbs1984		Hypothetical		Hypothetical protein	0,506	0,000	0,628	0,004	0,450	0,000	0,517	0,000
1031226	-	gbs1985		Hypothetical		Hypothetical membrane spanning protein	0,367	0,000	0,492	0,000	0,296	0,000	0,437	0,000
1031224	COG0488R, (	gbs1986		Transport and binding proteins	Unknown substrate	ABC transporter ATP-binding protein	0,458	0,000	0,565	0,000	0,389	0,000	0,497	0,000
1031252	-	gbs1987		Cellular processess	Toxin production and resistance	Streptomycin adenylyltransferase (Aminoglycoside 6- adenylyltransferase) (Aminoglyc	0,720	0,088	0,279	0,000	0,583	0,000	0,454	0,005
1031273	-	gbs1988		Hypothetical		Hypothetical protein	0,541	0,001	0,994	0,987	0,608	0,001	0,783	0,118
1031271	-	gbs1989		Hypothetical		Hypothetical protein	0,639	0,003	1,024	0,923	0,495	0,001	1,091	0,471
1031272	COG1695K	gbs1990		Cellular processess	Transcription	Transcriptional regulator, PadR family	0,780	0,053	1,646	0,178	1,245	0,249	1,748	0,001
1031274	-	gbs1991		Metabolism and transport	Central intermediary metabolism	Acetyltransferase, GNAT family	0,837	0,699	2,485	0,246	0,661	0,272	1,159	0,601
1031282	-	gbs1992		Transport and binding proteins	Unknown substrate	ABC transporter permease protein	2,028	0,075	2,514	0,102	2,265	0,039	3,123	0,026
1031275	COG1131V	gbs1993		Transport and binding proteins	Unknown substrate	ABC transporter ATP-binding protein	2,360	0,014	3,540	0,034	2,421	0,040	2,810	0,003
1031279	COG1396K	gbs1994		Cellular processess	Transcription	Transcriptional regulator, Cro CI family	2,298	0,021	2,358	0,007	1,098	0,750	2,396	0,012
1031276	-	gbs1995		Metabolism and transport	Fatty acid and phospholipids	Phosphatidylglycerophosphatase B homolog	1,488	0,133	1,778	0,325	1,118	0,782	0,792	0,496
1031286	COG1694R	gbs1996		Hypothetical		Hypothetical protein	1,168	0,195	0,389	0,000	0,849	0,170	0,349	0,000
1031281	COG1611R	gbs1997		General function predicted only		Lysine decarboxylase family	1,035	0,759	0,429	0,000	0,947	0,622	0,336	0,000
1031284	COG1266R	gbs1998		Cellular processess	Posttranslational modification, protein turnover, chaperones	CAAX amino terminal protease family	0,962	0,718	0,492	0,007	0,593	0,029	0,647	0,024
1031280	COG0607P	gbs1999		Metabolism and transport	Inorganic ion transport and metabolism	Rhodanese-related sulfurtransferases	0,953	0,790	0,458	0,000	0,921	0,673	0,751	0,072
1031278	-	gbs2000	cfb	Cellular processess	Transcription	CAMP factor	0,943	0,693	0,359	0,000	0,919	0,633	0,624	0,005
1031283	COG0563F	gbs2001	flaR	Metabolism and transport	Purines, pyrimidines, nucleosides, and nucleotides	DNA topology modulation protein flar-related protein	1,213	0,060	1,715	0,062	1,522	0,013	1,464	0,047
1031285	COG0371C	gbs2002		Metabolism and transport	Energy production and conversion	Glycerol dehydrogenase (EC 1.1.1.6)	1,459	0,027	0,628	0,011	1,488	0,013	0,505	0,001
1031288	COG4420S	gbs2003		Hypothetical		Hypothetical membrane spanning protein	0,948	0,882	0,664	0,353	1,021	0,956	1,038	0,928
1031287	COG0646E, (	gbs2004		Metabolism and transport	Amino acids, peptides, aminosugars and amines	5-methyltetrahydrofolate--homocysteine methyltransferase (EC 2.1.1.13) homocyste	0,073	0,101	0,118	0,116	0,096	0,108	0,109	0,113
1031289	COG0620E	gbs2005		Metabolism and transport	Amino acids, peptides, aminosugars and amines	5-methyltetrahydropteroyltriglutamate--homocysteine methyltransferase (EC 2.1.1.1	0,211	0,151	0,153	0,127	0,304	0,196	0,214	0,153
1031291	COG4392S	gbs2006		Metabolism and transport	Amino acids, peptides, aminosugars and amines	Branched-chain amino acid transport protein azlD	0,234	0,000	0,214	0,000	0,169	0,000	0,281	0,000
1031296	COG1296E	gbs2007		Metabolism and transport	Amino acids, peptides, aminosugars and amines	Branched-chain amino acid transport protein azlC	0,346	0,000	0,378	0,000	0,356	0,000	0,265	0,000
1031304	COG1404O	gbs2008		Cellular processess	Posttranslational modification, protein turnover, chaperones	LPXTG Endopeptidase lactocepin (EC 3.4.21.96)	1,159	0,280	0,489	0,005	1,221	0,160	0,403	0,002
1031388	COG0745TK	gbs2009		Cellular processess	Transcription	Transcriptional regulatory protein	0,936	0,562	0,579	0,015	0,795	0,082	0,560	0,005
1031308	COG0642T	gbs2010		Cellular processess	Signal transduction	Two component system histidine kinase (EC 2.7.3.-)	0,815	0,096	0,604	0,030	0,696	0,081	0,947	0,810
1031392	-	gbs2011		Hypothetical		Hypothetical protein	1,574	0,000	0,920	0,502	1,441	0,002	0,901	0,463
1031290	COG0495J	gbs2012	leuS	Cellular processess	Translation, ribosomal structure and biogenesis	Leucyl-tRNA synthetase (EC 6.1.1.4)	0,602	0,000	0,666	0,001	0,552	0,000	0,734	0,000
1031390	-	gbs2013		Transport and binding proteins	Unknown substrate	Transporter, MFS superfamily	12,869	0,000	5,692	0,001	13,587	0,000	4,282	0,001
1031391	COG4667R	gbs2014		Cellular processess	Posttranslational modification, protein turnover, chaperones	Phospholipase (EC 3.1.-.-)	0,887	0,286	0,397	0,002	0,723	0,017	0,409	0,000
1031395	COG1442M	gbs2015		Metabolism and transport	Central intermediary metabolism	Glycosyl transferase, family 8	0,424	0,000	0,289	0,000	0,371	0,000	0,294	0,000
1031328	COG1442M	gbs2016		Metabolism and transport	Central intermediary metabolism	Glycosyl transferase, family 8	0,399	0,000	0,259	0,000	0,402	0,000	0,298	0,000
1031405	COG0250K	gbs2017	nusG	Cellular processess	Transcription	Transcription antitermination protein nusG	1,951	0,000	0,839	0,171	1,652	0,005	0,743	0,011
1031394	-	gbs2018	bibA	Hypothetical		LPXTG Hypothetical protein	6,906	0,002	2,360	0,000	5,540	0,001	2,028	0,000
1031393	COG0690U	gbs2019	secE	Cellular processess	Posttranslational modification, protein turnover, chaperones	Protein translocase subunit secE	0,374	0,000	0,398	0,000	0,341	0,000	0,361	0,000
1031409	COG0744M	gbs2020	pbp2A	Cell Envelope	Biosynthesis and degradation of murein sacculus and peptidoglycar	Multimodular transpeptidase-transglycosylase PBP 2A	0,458	0,000	0,614	0,001	0,513	0,000	0,640	0,001
1031404	COG0564J	gbs2021		Cellular processess	Translation, ribosomal structure and biogenesis	Ribosomal large subunit pseudouridine synthase D (EC 4.2.1.70)	0,360	0,000	0,358	0,000	0,380	0,000	0,384	0,000
1031397	COG4640S	gbs2022		Hypothetical		Hypothetical protein	1,269	0,028	1,720	0,000	1,127	0,198	1,644	0,000
1031407	COG0274F	gbs2024	deoC	Metabolism and transport	Purines, pyrimidines, nucleosides, and nucleotides	Deoxyribose-phosphate aldolase (EC 4.1.2.4)	0,738	0,005	1,821	0,000	0,839	0,046	5,025	0,000
1031408	-	gbs2025	nupC	Metabolism and transport	Purines, pyrimidines, nucleosides, and nucleotides	Nucleoside permease nupC	0,836	0,087	2,320	0,000	0,948	0,596	5,588	0,000
1031412	COG2820F	gbs2026	udp	Metabolism and transport	Purines, pyrimidines, nucleosides, and nucleotides	Uridine phosphorylase (EC 2.4.2.3)	1,040	0,583	2,766	0,000	1,365	0,003	6,709	0,000
1031415	COG2188K	gbs2027	crgR	Cellular processess	Transcription	Transcriptional regulator, GntR family	0,750	0,033	0,945	0,720	0,715	0,026	1,307	0,048
1031414	-	gbs2028		Hypothetical		Hypothetical protein	1,033	0,774	1,074	0,763	1,146	0,230	1,476	0,015
1031419	COG0459O	gbs2029	groEL	Cellular processess	Posttranslational modification, protein turnover, chaperones	60 kDa chaperonin GROEL	0,533	0,019	0,343	0,004	0,624	0,044	0,414	0,007
1031421	COG0234O	gbs2030	groES	Cellular processess	Posttranslational modification, protein turnover, chaperones	10 kDa chaperonin GROES	0,797	0,313	0,511	0,024	0,961	0,850	0,576	0,042
1031427	COG1101R	gbs2031		Transport and binding proteins	Unknown substrate	ABC transporter ATP-binding protein	0,442	0,002	0,249	0,000	0,329	0,001	0,279	0,000
1031423	COG4120R	gbs2032		Transport and binding proteins	Unknown substrate	ABC transporter permease protein	0,596	0,044	0,243	0,000	0,328	0,001	0,237	0,000
1031435	COG2984R	gbs2033		Transport and binding proteins	Unknown substrate	ABC transporter substrate-binding protein	0,273	0,001	0,189	0,000	0,462	0,016	0,168	0,000
1031459	COG0561R	gbs2034		Metabolism and transport	Central intermediary metabolism	Hydrolase (HAD superfamily)	1,097	0,586	1,344	0,199	0,910	0,637	1,577	0,027
1031451	COG2514R	gbs2035		Metabolism and transport	Energy production and conversion	Glyoxalase family protein	2,591	0,287	0,954	0,920	2,573	0,224	1,104	0,827
1031452	COG3022S	gbs2036	yaaA	Hypothetical		Hypothetical cytosolic protein	0,467	0,011	0,405	0,000	0,455	0,011	0,385	0,000
1031438	COG0602O	gbs2037	nrdG	Cellular processess	Posttranslational modification, protein turnover, chaperones	Anaerobic ribonucleoside-triphosphate reductase activating protein	0,806	0,110	1,108	0,590	0,968	0,882	1,282	0,005
1031463	COG3981R	gbs2038		Metabolism and transport	Central intermediary metabolism	Acetyltransferase (EC 2.3.1.-)	0,331	0,000	0,315	0,000	0,346	0,000	0,489	0,000
1031457	COG0673R	gbs2039		Metabolism and transport	Central intermediary metabolism	NAD-dependent oxidoreductase	0,547	0,000	0,433	0,000	0,402	0,000	0,567	0,000
1031458	-	gbs2040		Hypothetical		Hypothetical protein	0,906	0,116	0,433	0,000	0,746	0,000	0,540	0,000
1031480	COG1328F	gbs2041	nrdD	Metabolism and transport	Purines, pyrimidines, nucleosides, and nucleotides	Anaerobic ribonucleoside-triphosphate reductase (EC 1.17.4.2)	1,820	0,010	2,973	0,003	1,621	0,020	2,907	0,000
1031460	-	gbs2042		Hypothetical		Hypothetical membrane spanning protein	0,360	0,000	0,465	0,000	0,262	0,000	0,529	0,000
1031467	COG3906S	gbs2043		Hypothetical		Hypothetical cytosolic protein	1,813	0,000	0,585	0,000	1,517	0,000	0,528	0,000
1031487	-	gbs2044		Cellular processess	DNA replication, recombination and repair	Endonuclease involved in recombination	1,968	0,000	0,684	0,000	1,640	0,000	0,609	0,000
1031478	-	gbs2045		Hypothetical		Hypothetical cytosolic protein	3,063	0,000	0,849	0,034	2,528	0,000	0,716	0,001
1031465	COG1393P	gbs2046	spxA	Metabolism and transport	Inorganic ion transport and metabolism	Arsenate reductase family protein	1,635	0,000	1,210	0,106	1,609	0,001	1,218	0,210
1031471	COG0468L	gbs2047	recA	Cellular processess	DNA replication, recombination and repair	RecA protein	0,450	0,000	0,527	0,000	0,507	0,000	0,612	0,000
1031601	COG1058R, (	gbs2048	cinA	General function predicted only		Colligrin	0,574	0,001	0,848	0,242	0,522	0,000	0,707	0,017
1031543	-	gbs2049	tag	Cellular processess	DNA replication, recombination and repair	DNA-3-methyladenine glycosylase (EC 3.2.2.20)	0,355	0,000	0,567	0,001	0,411	0,000	0,693	0,005
1031545	COG0632L	gbs2050	ruvA	Cellular processess	DNA replication, recombination and repair	Holliday junction DNA helicase ruvA	0,186	0,000	0,308	0,000	0,183	0,000	0,586	0,001
1031501	-	gbs2051	lmrP	Cellular processess	Toxin production and resistance	Multidrug resistance protein lmrP	0,187	0,000	0,243	0,000	0,141	0,000	0,369	0,000
1031648	COG0323L	gbs2052	mutL	Cellular processess	DNA replication, recombination and repair	DNA mismatch repair protein mutL	0,570	0,000	0,766	0,029	0,500	0,000	1,003	0,976
1031494	-	gbs2053	csp	Cellular processess	Posttranslational modification, protein turnover, chaperones	Cold shock protein	0,503	0,010	0,465	0,008	0,371	0,003	0,222	0,001
1031681	COG0249L	gbs2054	mutS	Cellular processess	DNA replication, recombination and repair	DNA mismatch repair protein mutS	0,699	0,002	0,907	0,604	0,618	0,005	1,027	0,754
1031544	COG1438K	gbs2055	argR2	Cellular processess	Transcription	Arginine repressor, argR	0,652	0,000	0,450	0,000	0,634	0,000	0,576	0,000
1031682	COG0018J	gbs2056	argS	Cellular processess	Translation, ribosomal structure and biogenesis	Arginyl-tRNA synthetase (EC 6.1.1.19)	0,695	0,000	0,816	0,004	0,733	0,000	0,856	0,019
1031685	-	gbs2057	uviB			Bacteriocin uviB	0,387	0,000	0,172	0,000	0,329	0,000	0,238	0,000
1031680	COG1284S	gbs2058		Hypothetical		Hypothetical membrane spanning protein	0,517	0,002	0,876	0,407	0,539	0,002	0,752	0,075
1030780	COG1284S	gbs2059		Hypothetical		Hypothetical membrane spanning protein	0,215	0,000	0,418	0,000	0,224	0,000	0,434	0,000
1031827	COG0173J	gbs2060	aspS	Cellular processess	Translation, ribosomal structure and biogenesis	Aspartyl-tRNA synthetase (EC 6.1.1.12)	0,324	0,000	0,328	0,000	0,245	0,000	0,399	0,000
1031683	COG0124J	gbs2061	hisS	Cellular processess	Translation, ribosomal structure and biogenesis	Histidyl-tRNA synthetase (EC 6.1.1.21)	0,499	0,000	0,326	0,000	0,468	0,000	0,248	0,000
1031684	COG0333J	gbs2062	rpmF	Cellular processess	Translation, ribosomal structure and biogenesis	LSU ribosomal protein L32P	0,968	0,611	0,539	0,000	0,792	0,006	0,628	0,001
1030811	COG0267J	gbs2063	rpmG	Cellular processess	Translation, ribosomal structure and biogenesis	LSU ribosomal protein L33P	1,333	0,008	0,549	0,000	1,262	0,010	0,563	0,000
1030716	-	gbs2064	cadD	Cellular processess	Toxin production and resistance	Cadmium resistance protein	0,657	0,002	0,504	0,000	0,674	0,003	0,435	0,003
1030808	COG0640K	gbs2065	cadC	Cellular processess	Toxin production and resistance	Cadmium efflux system accessory protein	0,699	0,006	0,576	0,009	0,695	0,043	0,577	0,005
1030814	-	gbs2066		Hypothetical		Hypothetical protein	0,730	0,001	0,502	0,009	0,630	0,007	0,628	0,000
1030813	-	gbs2067		Hypothetical		Hypothetical protein	2,008	0,019	4,986	0,000	4,456	0,077	3,878	0,011
1030815	-	gbs2068		Hypothetical		Hypothetical protein	4,301	0,000	5,997	0,001	5,635	0,000	5,666	0,000
1030830	COG1674D	gbs2069		Cellular processess	Cell division	FtsK SpoIIIE family	3,556	0,002	4,705	0,018	1,784	0,119	3,753	0,097
1031433	-	gbs2070		Hypothetical		Hypothetical protein	3,558	0,008	2,306	0,014	2,501	0,055	2,671	0,080
1030817	-	gbs2071		Mobile and extrachromosomal elements		Replication protein	2,450	0,010	6,975	0,016	3,327	0,001	3,069	0,006

1030783	-	gbs2072		Mobile and extrachromosomal elements		Phage protein	3,116	0,237	2,512	0,145	2,397	0,288	1,617	0,377
1030747	COG0582L	gbs2073		Mobile and extrachromosomal elements		DNA integration recombination inversion protein	0,362	0,000	0,424	0,000	0,330	0,000	0,430	0,000
1030842	-	gbs2074		Hypothetical		Hypothetical cytosolic protein	0,249	0,000	0,553	0,000	0,256	0,000	0,767	0,048
1030781	-	gbs2076		Hypothetical		Hypothetical protein	0,578	0,005	0,417	0,001	0,642	0,035	0,506	0,002
1030820	-	gbs2077		Hypothetical		Hypothetical protein	0,354	0,000	0,419	0,001	0,236	0,000	0,365	0,000
1031055	COG3212S	gbs2080		Hypothetical		Hypothetical protein	3,880	0,017	2,624	0,011	3,653	0,125	2,803	0,210
1030819	COG0745TK	gbs2081		Cellular processess	Transcription	Transcriptional regulatory protein	0,895	0,347	0,801	0,211	1,064	0,570	0,666	0,001
1030759	COG0642T	gbs2082		Cellular processess	Signal transduction	Two-component sensor kinase czcS (EC 2.7.3.-)	0,566	0,000	0,483	0,000	0,572	0,000	0,630	0,001
1030703	COG1288S	gbs2083		Metabolism and transport	Amino acids, peptides, aminosugars and amines	Arginine ornithine antiporter	2,888	0,001	8,309	0,017	2,702	0,006	4,465	0,000
1030750	COG0549E	gbs2084		Metabolism and transport	Amino acids, peptides, aminosugars and amines	Carbamate kinase (EC 2.7.2.2)	1,542	0,205	5,188	0,056	1,751	0,163	3,509	0,008
1031013	COG0078E	gbs2085		Metabolism and transport	Amino acids, peptides, aminosugars and amines	Ornithine carbamoyltransferase (EC 2.1.3.3)	2,392	0,006	6,422	0,011	2,501	0,007	3,441	0,000
1031066	COG0642T	gbs2086		Cellular processess	Signal transduction	Sporulation kinase A (EC 2.7.3.-)	0,578	0,045	0,667	0,117	0,396	0,003	1,092	0,728
1030729	COG4753T	gbs2087		Cellular processess	Signal transduction	Two-component response regulator	0,564	0,015	0,827	0,525	0,266	0,000	1,421	0,011
1031098	COG1125E	gbs2088	proV	Metabolism and transport	Amino acids, peptides, aminosugars and amines	Glycine betaine transport ATP-binding protein	0,576	0,003	0,260	0,000	0,511	0,001	0,222	0,000
1030749	COG1174E, C	gbs2089		Metabolism and transport	Amino acids, peptides, aminosugars and amines	Glycine betaine transport system permease protein Glycine betaine-binding protein	0,526	0,000	0,407	0,000	0,504	0,000	0,342	0,000
1031102	COG0392S, C	gbs2090		General function predicted only		Lysylcardiolipin synthase (EC 2.3.2.-) Lysyltransferase (EC 2.3.2.3)	0,408	0,000	0,597	0,000	0,358	0,000	0,690	0,001
1031088	COG0596R	gbs2091		General function predicted only		Non-heme chloroperoxidase (EC 1.11.1.10)	0,383	0,000	0,522	0,001	0,330	0,000	0,535	0,000
1030688	COG3759S	gbs2092		Hypothetical		Hypothetical membrane spanning protein	1,020	0,963	1,968	0,411	1,374	0,546	1,646	0,260
1030562	COG1511S	gbs2093		Mobile and extrachromosomal elements		Phage infection protein	0,430	0,008	1,406	0,477	0,464	0,001	0,399	0,000
1030957	COG1309K	gbs2094		Cellular processess	Transcription	Transcriptional regulator, TetR family	0,940	0,813	1,117	0,731	0,504	0,034	0,751	0,307
1030235	-	gbs2095		Hypothetical		Hypothetical protein	0,923	0,779	1,213	0,486	0,822	0,530	0,446	0,008
1029947	COG0522J	gbs2096	rpsD	Cellular processess	Translation, ribosomal structure and biogenesis	SSU ribosomal protein S4P	0,529	0,000	0,749	0,007	0,529	0,000	0,750	0,012
1031112	-	gbs2097		Hypothetical		Hypothetical protein	0,417	0,000	0,433	0,000	0,345	0,000	0,431	0,000
1031046	COG0305L	gbs2098	holB	Cellular processess	DNA replication, recombination and repair	Replicative DNA helicase (EC 3.6.1.-)	0,423	0,000	0,619	0,000	0,347	0,000	0,637	0,000
1030821	COG0359J	gbs2099	rplI	Cellular processess	Translation, ribosomal structure and biogenesis	LSU ribosomal protein L9P	0,572	0,000	0,618	0,000	0,487	0,000	0,636	0,000
1030960	COG3887T	gbs2100		Metabolism and transport	Central intermediary metabolism	Phosphoesterase, DHH family protein	0,444	0,000	0,561	0,000	0,335	0,000	0,595	0,000
1030341	COG0445D	gbs2101	gidA	General function predicted only		Putative tRNA (5-carboxymethylaminomethyl-2-thiouridylate) synthase subunit GidA	0,584	0,000	0,783	0,050	0,670	0,002	1,104	0,529
1031036	COG2095U	gbs2102		Cell Envelope	Other	MarC family integral membrane protein	0,347	0,000	0,516	0,001	0,377	0,000	0,561	0,001
1030810	COG0482J	gbs2103	trmU	Cellular processess	Translation, ribosomal structure and biogenesis	tRNA (5-methylaminomethyl-2-thiouridylate)-methyltransferase (EC 2.1.1.61)	0,384	0,000	0,583	0,003	0,380	0,000	0,651	0,012
1031056	COG1760E	gbs2104	sdhB	Metabolism and transport	Amino acids, peptides, aminosugars and amines	L-serine dehydratase beta subunit (EC 4.3.1.17)	0,238	0,000	0,222	0,000	0,230	0,000	0,444	0,001
1030711	COG1760E	gbs2105	sdhA	Metabolism and transport	Amino acids, peptides, aminosugars and amines	L-serine dehydratase alpha subunit (EC 4.3.1.17)	0,930	0,379	1,273	0,146	0,941	0,442	1,483	0,006
1031113	-	gbs2106		General function predicted only		Transglycosylase SLT domain family protein	0,299	0,000	0,278	0,000	0,290	0,000	0,336	0,000
1031059	-	gbs2107		Cell Envelope	Biosynthesis and degradation of surface polysaccharides and lipopo	peptidoglycan binding protein (LysM domain)	0,341	0,001	0,156	0,000	0,316	0,000	0,141	0,000
1030956	COG0619P	gbs2108	cbiQ	Metabolism and transport	Inorganic ion transport and metabolism	Hyaluronan ABC exporter permease protein	0,463	0,000	0,543	0,000	0,418	0,000	0,466	0,000
1030712	COG1122P	gbs2109	cbiO2	Metabolism and transport	Inorganic ion transport and metabolism	Hyaluronan ABC exporter ATP-binding protein	0,282	0,000	0,471	0,000	0,283	0,000	0,511	0,000
1030739	COG1122P	gbs2110	cbiO1	Metabolism and transport	Inorganic ion transport and metabolism	Hyaluronan ABC exporter ATP-binding protein	0,417	0,000	0,495	0,000	0,363	0,000	0,506	0,000
1030710	COG0558I	gbs2111	pgsA	Metabolism and transport	Fatty acid and phospholipids	CDP-diacylglycerol--glycerol-3-phosphate 3-phosphatidyltransferase (EC 2.7.8.5)	0,414	0,000	0,481	0,000	0,401	0,000	0,481	0,000
1030721	COG0612R	gbs2112		Cellular processess	Posttranslational modification, protein turnover, chaperones	Peptidase, M16 family	0,231	0,000	0,445	0,002	0,191	0,000	0,401	0,000
1030765	COG0612R	gbs2113		General function predicted only		Non-proteolytic protein, peptidase family M16	0,204	0,000	0,476	0,005	0,248	0,000	0,305	0,000
1030822	COG2501S	gbs2114		Hypothetical		Hypothetical cytosolic protein	0,441	0,001	0,426	0,001	0,351	0,000	0,386	0,000
1030733	COG1195L	gbs2115	recF	Cellular processess	DNA replication, recombination and repair	DNA replication and repair protein recF	0,466	0,001	0,578	0,010	0,463	0,001	0,493	0,002
1031093	COG4975G	gbs2116		Metabolism and transport	Carbohydrates, organic alcohols, and acids	Glucose uptake family protein	0,463	0,000	0,397	0,000	0,360	0,000	0,441	0,000
1031097	-	gbs2117		Cellular processess	Transcription	Transcriptional regulator, Cro CI family	0,202	0,000	0,227	0,000	0,157	0,000	0,168	0,000
1030752	COG0516F, C	gbs2118	guaB	Metabolism and transport	Purines, pyrimidines, nucleosides, and nucleotides	Inosine-5 -monophosphate dehydrogenase (EC 1.1.1.205)	0,468	0,000	0,320	0,000	0,516	0,000	0,263	0,000
1030751	COG1438K	gbs2119	ahrC.2	Cellular processess	Transcription	Arginine repressor, argR	0,811	0,010	1,009	0,882	0,699	0,009	0,847	0,051
1031069	COG0664T	gbs2120		Cellular processess	Transcription	Transcriptional regulatory protein	0,656	0,000	1,008	0,917	0,616	0,000	0,963	0,514
1030823	COG3382S	gbs2121		Hypothetical		Hypothetical protein	0,749	0,104	0,788	0,213	0,600	0,032	0,909	0,543
1031077	COG2235E	gbs2122	arcA	Metabolism and transport	Amino acids, peptides, aminosugars and amines	Arginine deiminase (EC 3.5.3.6)	8,800	0,006	8,033	0,001	15,443	0,002	5,434	0,000
1031073	-	gbs2123		Metabolism and transport	Central intermediary metabolism	Acetyltransferase (EC 2.3.1.-)	5,448	0,029	8,621	0,003	10,394	0,005	4,900	0,000
1031070	COG0078E	gbs2124	arcB	Metabolism and transport	Amino acids, peptides, aminosugars and amines	Ornithine carbamoyltransferase (EC 2.1.3.3)	3,337	0,021	8,171	0,002	7,140	0,006	5,394	0,000
1030825	COG0531E	gbs2125		Metabolism and transport	Amino acids, peptides, aminosugars and amines	Arginine ornithine antiporter	2,220	0,085	7,440	0,001	4,544	0,018	4,471	0,000
1030767	COG0549E	gbs2126	arcC	Metabolism and transport	Amino acids, peptides, aminosugars and amines	Carbamate kinase (EC 2.7.2.2)	1,166	0,637	5,487	0,001	2,322	0,101	2,903	0,000
1030760	COG0180J	gbs2127	trpS	Cellular processess	Translation, ribosomal structure and biogenesis	Tryptophanyl-tRNA synthetase (EC 6.1.1.2)	0,515	0,000	0,636	0,000	0,476	0,000	0,840	0,008
1030806	-	gbs2128		Hypothetical		Hypothetical membrane spanning protein	0,618	0,051	0,658	0,088	0,611	0,050	0,440	0,007
1030763	COG1284S	gbs2129		Hypothetical		Hypothetical membrane spanning protein	0,169	0,000	0,322	0,000	0,182	0,000	0,320	0,000
1031072	COG0488R	gbs2130		Transport and binding proteins	Unknown substrate	ABC transporter ATP-binding protein	0,494	0,000	0,496	0,000	0,469	0,000	0,538	0,000
1031067	-	gbs2131		Transport and binding proteins	Unknown substrate	ABC transporter, permease protein	0,420	0,000	0,639	0,019	0,484	0,001	0,592	0,001
1031071	COG1576S	gbs2132		Hypothetical		Hypothetical cytosolic protein	0,712	0,020	0,498	0,000	0,596	0,003	0,546	0,000
1030829	COG0265O	gbs2133	htrA	Cellular processess	Posttranslational modification, protein turnover, chaperones	Endopeptidase degP (EC 3.4.21.-)	1,622	0,000	0,607	0,002	1,631	0,004	0,422	0,000
1031012	COG1475K	gbs2134	parB	Cellular processess	Transcription	Chromosome partitioning protein parB	0,661	0,001	0,468	0,000	0,676	0,003	0,475	0,000
